# Executable network of SARS-CoV-2-host interaction predicts drug combination treatments

**DOI:** 10.1038/s41746-022-00561-5

**Published:** 2022-02-14

**Authors:** Rowan Howell, Matthew A. Clarke, Ann-Kathrin Reuschl, Tianyi Chen, Sean Abbott-Imboden, Mervyn Singer, David M. Lowe, Clare L. Bennett, Benjamin Chain, Clare Jolly, Jasmin Fisher

**Affiliations:** 1 UCL Cancer Institute, University College London, 72 Huntley Street, London, WC1E 6DD UK; 2 Division of Infection and Immunity, University College London, London, WC1E 6BT UK; 3 Bloomsbury Institute of Intensive Care Medicine, Division of Medicine, University College London, London, WC1E 6BT UK; 4 Institute of Immunity and Transplantation, University College London, London, NW3 2PF UK; 5 Department of Computer Science, Gower Street, University College London, London, WC1E 6BT UK

**Keywords:** Virtual drug screening, Cellular signalling networks, Dynamic networks, Regulatory networks

## Abstract

The COVID-19 pandemic has pushed healthcare systems globally to a breaking point. The urgent need for effective and affordable COVID-19 treatments calls for repurposing combinations of approved drugs. The challenge is to identify which combinations are likely to be most effective and at what stages of the disease. Here, we present the first disease-stage executable signalling network model of SARS-CoV-2-host interactions used to predict effective repurposed drug combinations for treating early- and late stage severe disease. Using our executable model, we performed in silico screening of 9870 pairs of 140 potential targets and have identified nine new drug combinations. Camostat and Apilimod were predicted to be the most promising combination in effectively supressing viral replication in the early stages of severe disease and were validated experimentally in human Caco-2 cells. Our study further demonstrates the power of executable mechanistic modelling to enable rapid pre-clinical evaluation of combination therapies tailored to disease progression. It also presents a novel resource and expandable model system that can respond to further needs in the pandemic.

## Introduction

COVID-19 is a complex disease in both dynamics and severity^1–4^. While most infections only result in mild symptomatic presentation, a proportion suffers more severe disease, for example, the CDC estimate 4.9% of SARS-CoV-2 infections in the USA resulted in hospitalisation in the period up to March 2021^5,6^. Mild cases see an innate immune and interferon (IFN-I/III) response to SARS-CoV-2 infection, as typically observed in other viral infections, likely supporting clearance of the virus and the onset of adaptive immunity^1,7,8^. By contrast, a delayed or absent IFN-I/III response to infection may contribute to the presentation of severe disease^7,9–15^. This is especially of concern as emerging variants have adapted to allow potent host immune antagonism^16^, allowing the virus to replicate with little opposition in the early stages of infection^1,10^. This can then be followed by a maladaptively strong and persistent inflammatory response only after the majority of viral replication has occurred^9,17^, leading to life-threatening conditions such as acute respiratory distress syndrome (ARDS)^18^.

There is an urgent need for new effective drugs to manage COVID-19 infection to lower morbidity, mortality and reduce the strain on healthcare systems^19,20^. While several vaccines have been approved^21–23^, many millions more patients will need treatment during the years it will take to deliver vaccination worldwide^24,25^. The path for de novo drug discovery is long and complex; the best hope for rapid development of new therapies is, therefore, to combine readily available drugs^26^, with a focus on affordable and well-tested treatments, such as Dexamethasone, which will be necessary to treat COVID-19 in low-income countries that are struggling to obtain sufficient doses of vaccines. Given the large range of potentially suitable compounds, several challenges arise: which drugs are most effective and at what stage of the infection? Are there combinations of drugs that allow effective treatment at lower doses and reduced toxicity?

Driven by the different characteristics of early and late stages of severe COVID-19, and the need to find therapies appropriate to each stage of the disease without interfering with the effective immune response in mild cases, we have developed a computational model that can reproduce these different phases of COVID-19. This approach builds upon our previous success at finding novel drug targets and effective drug combinations for cancer therapy^27–30^. We created a detailed network map of the interaction between SARS-CoV-2 and lung epithelial cells, as pulmonary involvement, in particular, is a hallmark of severe disease. We use this model to screen thousands of drug combinations to find treatments that block either key viral–host interactions important to viral replication, or the pathogenic dysregulation of the immune response. We focus especially on host-directed therapies, which may be more robust to future variants of the virus^31^. We identify several combinations of repurposed drugs that are predicted to act together to target viral replication in the early stages of the disease or inflammation in the late stage. Moreover, we propose that the model and screen results represent a powerful resource for the community to generate hypotheses about the potential effects of targeting host and viral targets in combination.

## Results

### Executable network map of the interaction between SARS-CoV-2 and lung epithelium

**Fig. 1:**
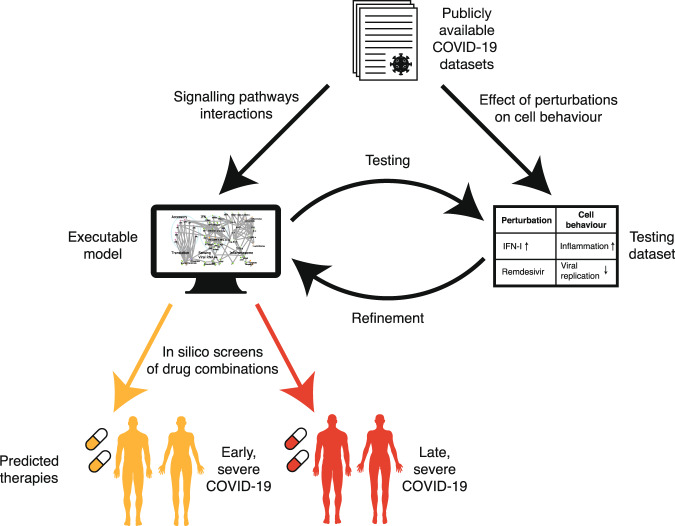
Schematic workflow of SARS-CoV-2 infection modelling. Publicly available COVID-19 datasets are collated into two subsets. Data on the point-to-point signalling pathway interactions of the virus with the host cell, and of the host cell in response to infection, are used to build a network model. Data from experiments showing how the overall behaviour of the infected cell changes under perturbations, such as a potential treatment, are used as a testing dataset to validate the model. The readouts of the model are compared to the testing dataset and the model is refined iteratively until it reproduces all the experimentally observed behaviours. We then screen the effects of potential drug treatments, either singly or in combination, on the model, to find the best-predicted therapies for early and late stage severe COVID-19.

**Fig. 2:**
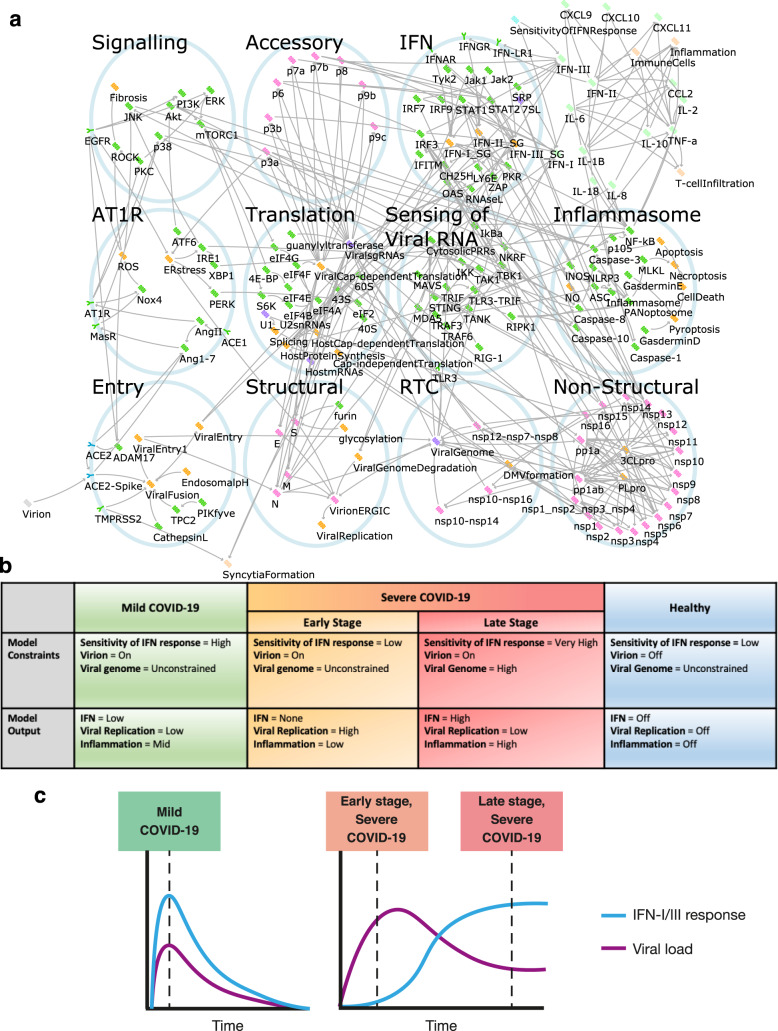
Computational modelling of host–virus interaction in COVID-19. **a** The SARS-CoV-2 infection signalling network as modelled in the BioModelAnalyzer tool. Viral proteins depicted in pink, host proteins depicted in green, cellular processes depicted in orange, RNAs depicted in purple. Activating interactions are represented by arrows (→), inactivating by bars (⊣). **b** Description of the model constraints and outputs for the three states of the disease and the control healthy state. **c** Trajectories of IFN –I/III response and viral load in mild and severe COVID-19 (as described in Park and Iwasaki, *Cell Host and Microbe*, 2020) were used to inform the model.

We model the interaction map between SARS-CoV-2 and lung epithelial cells as an executable Qualitative Network^32^ model (Fig. 1) using the BioModelAnalyzer (BMA) tool (https://biomodelanalyzer.org). This model is an executable computer program in graphical form characterising the signalling regulatory network (Fig. 2a), made up of 175 nodes, representing viral and host proteins, as well as cellular processes affected by these proteins such as viral replication and inflammation. There are 387 edges joining these nodes representing activating or inhibiting interactions (Supplementary Table 1). At any point, each node has a discrete level that represents its activity that is determined by the input of activations and inhibitions that the node receives and the node’s target function (Methods, Supplementary Table 2).

Given the importance of the degree of type I/III IFN response in disease progression^1,7,10,12,15,33^, we use it in our modelling to reproduce the mild case and both the early and late stages of severe cases of COVID-19, as well as an uninfected control, through setting key input nodes in the model (see Fig. 2b and Supplementary Table 3). The mild case is characterised by modest symptoms from presentation to resolution, in part due to a rapid IFN response. In contrast, severe disease may present with mild symptoms initially, but in these patients, the IFN-I/III response is evaded and suppressed^1,7,15,33^. This presents an opportunity for intervention to prevent progression to the late stage of severe disease through treatments targeting viral replication. Conversely, in the late stage of severe disease, IFN-I/III response is increased at the site of infection^34^ and most viral reproduction has already occurred. This leaves behind viral RNA and proteins that trigger further maladaptive immune responses, so anti-inflammatory treatments may be more appropriate to control severe symptoms.

These different treatment goals require modelling of these stages of severe disease separately. To accomplish this, we use the node “Sensitivity of IFN Response” to convey both the response to and magnitude of, IFN-I/III production and set this node in the model to a low level in the early stage of severe disease. In the late stage, we set this to high, and specify a high burden of viral RNA and protein by the Viral Genome node, assuming replication has peaked (Fig. 2b, c and Supplementary Table 3). Finally, our model also represents mild disease and uninfected individuals, in order to investigate whether treatments are predicted to have adverse effects beyond known side effects. For example, treatments that reduce maladaptive inflammation in the late severe case may inhibit beneficial inflammatory response if given improperly at the early stages of infection or in mild disease.

### Network model validation by comparison to known effects of SARS-CoV-2 infection and treatment

**Fig. 3:**
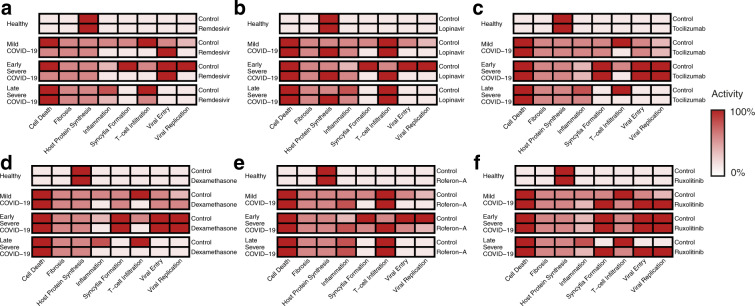
Model predictions of the effects of known drug treatments for COVID-19. The predicted behaviour of **a** Remdesivir, **b** Lopinavir, **c** Tocilizumab, **d** Dexamethasone, **e** Roferon-A and **f** Ruxolitinib. Colour strength represents the strength of symptoms predicted by the model following drug intervention. All nodes normalised to the maximal level of respective nodes, and range between 0–100%. Comparison to known experimental results in Supplementary Table 4.

Having generated a network model of viral infection, we first verified that it reproduced key results from experiments that have defined SARS-CoV-2 interaction with lung epithelial cells, as well as known clinical results establishing the utility of different treatments at different stages of the disease (Supplementary Table 4). In particular, we tested the model responses to monotherapies that have been used as COVID-19 treatments, such as the antivirals Remdesivir^35^ and Lopinavir^36^; and the immune modulators Tocilizumab^37^, Dexamethasone^38^, Interferon α-2a (Roferon-A)^39^ and Ruxolitinib^40^. Drugs were modelled by setting the affected node in the signalling network to either zero, to represent an inhibitory drug, or to the maximum value, to represent an agonist. The effect of the treatment was then assessed by comparing the resulting level of the nodes representing cellular processes such as viral replication and inflammation to known experimental results (Supplemental Table 4). We then predicted the response of all phenotypes to these drugs (Fig. 3).

### Inhibiting protein translation is predicted to suppress viral replication in the early stages of severe COVID-19

Our approach to find new and improved treatments is to test the response of our computational model to an exhaustive search across combinations of many different drugs, either clinically approved or in phase II/III trials, to determine optimal treatments for each stage of development of the disease (Table 1). We implemented the screening by fixing the activity of nodes in the network to a constant value to represent the effect of mono- or combination treatments over all drug combinations and disease stages (Fig. 2b and Supplementary Tables 3, 5). This *in silico* screen predicted the most effective drug treatments against each stage of COVID-19 and checked for additional adverse effects beyond known drug side effects in mild cases of the disease compared to the severe cases.

**Table 1:** List of approved drugs used for in silico screening and their targets in the network model.

Drug	Pathway	Targeted node	References
4-PBA	AT1R	ER stress	Hsu, A. C.-Y., 2020; Kolb, P. S., 2015
ACEi	AT1R	ACE1	Vaduganathan, M., 2020
Actimmune	IFN	IFN-II	Razaghi, A., 2016
Aliskiren	AT1R	AngII, Ang1-7	Sriram, K., 2020
Anifrolumab	IFN	IFNAR	Riggs, J. M., 2018
Apilimod	Entry	PIKfyve	Ou, X., 2020
ARB	AT1R	AT1R	Vaduganathan, M., 2020
Baricitinib	IFN	Jak1, Jak2, ViralFusion	Richardson, P., 2020
Basiliximab	Cytokines & Chemokines	IL-2	Onrust, S., 1999
Belnacasan	Inflammasome	Caspase-1	Kudelova, J., 2015
Camostat	Entry	TMPRSS2	Hoffmann, M., 2020
Canakinumab	Cytokines and chemokines	IL-1B	Ucciferri, C., 2020
Carlumab	Cytokines and chemokines	CCL2	Lim, S. Y., 2016
Cetuximab	Signalling	EGFR	Drugbank 2021
Chloroquine	Structural	EndosomalpH, glycosylation, TNF-a, IL-6	Wang, M., 2020; Savarino, A., 2003
Colchicine	Inflammasome	Inflammasome	Martinez, G. J., 2017
Copanlisib	Signalling	PI3K	Yang, J., 2019
Dexamethasone	Inflammasome	IL-10, TNF-a, NF-kB, JNK, ERK, p38, IL-1B, IL-8	Selvaraj, V., 2020; Smoak, K. A., 2004; RECOVERY 2021
Disulfiram	Inflammasome	GasderminD	Hu, J. J., 2020
Emricasan	Inflammasome	Caspase-1, Caspase-3, Caspase-8, Caspase-10	Kudelova, J., 2015
Fedratinib	IFN	Jak2	Szelag, M., 2016
Homoharringtonine	Translation	60S	Choy, K.-T., 2020; Winer, E. S., 2018
Infliximab	Cytokines and chemokines	TNF-a	Robinson, P. C., 2020
Lopinavir	Non-structural	3CLpro	Choy, K.-T., 2020; Ma, C., 2020; Cao, B., 2020
Losmapimod	Signalling	p38	Drugbank 2021
Miltefosine	Signalling	Akt	Nitulescu, G. M., 2015
N-acetylcysteine	AT1R	ROS	Meng, Y., 2015
Nivocasan	Inflammasome	Caspase-1, Caspase-8	Kudelova, J., 2015
Rapamycin	Signalling	mTORC1	Ballou, L. M., 2008
Recombinant IL-10	Cytokines and chemokines	IL-10	Asadullah, K., 2003
Remdesivir	Non-structural	nsp12-nsp7-nsp8	Wang, M., 2020; Beigel, J. H., 2020
Roferon-A	IFN	IFN-I	Mantlo, E., 2020
Ruxolitinib	IFN	Jak1, Jak2	Szelag, M., 2016; Cao, Y., 2020; Thorne, L. G., 2021
SAR113945	Sensing of Viral RNA	IKK	Herrington, F. D., 2015
Tetrandrine	Entry	TPC2	Ou, X., 2020
Tocilizumab	Cytokines and chemokines	IL-6	Mihara, M., 2011; RECOVERY 2021
Ulixertinib	Signalling	ERK	Hsu, A. C.-Y., 2020
Umifenovir	Entry	ACE2-Spike, S	Wang, X., 2020; Kim, S. Y., 2020

The network model was used to evaluate the best single and combinations of 38 drugs, either clinically approved or in phase II/III trials at the time of writing. Drugs that had not passed phase II/III clinical trials at the time of writing are included in Supplementary Table 5 and the network model predictions for these drugs are shown in Supplementary Figs. 8, 9.

**Fig. 4:**
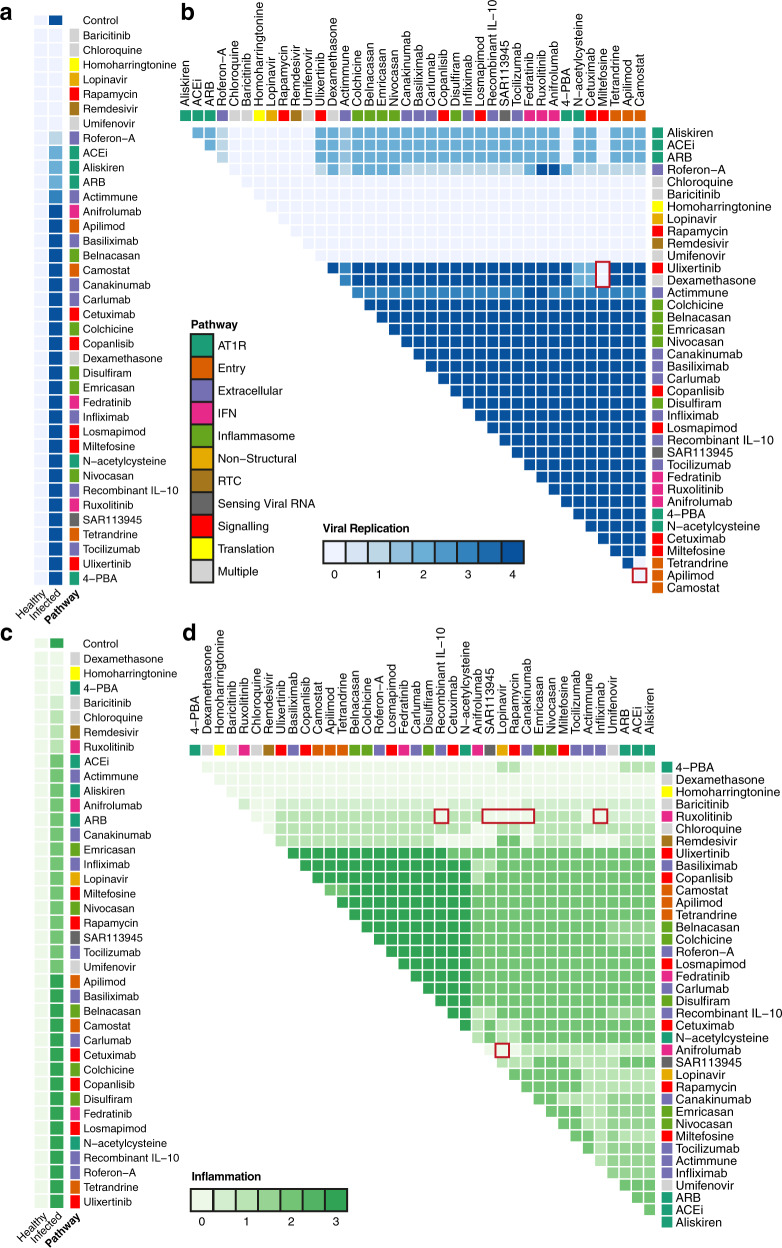
In silico screens for optimised drug combinations to treat COVID-19. **a** Effect of single drugs (rows) on viral replication in infected cells in the early stage of severe disease and in control healthy cells. **b** The effects of drug combinations on viral replication in an early stage of severe COVID-19. **c** The effect of single drugs (rows) on inflammation in infected cells in a late stage of severe disease and in control healthy cells. **d** Effect of drug combinations on inflammation in the late stage of severe COVID-19. Colour of the cell corresponds to the level of viral replication (blue) and inflammation (green). Red boxes indicate therapies discussed in detail. The annotations show the pathway affected by each drug. Pathway categories for each node are shown in Supplementary Table 6.

**Fig. 5:**
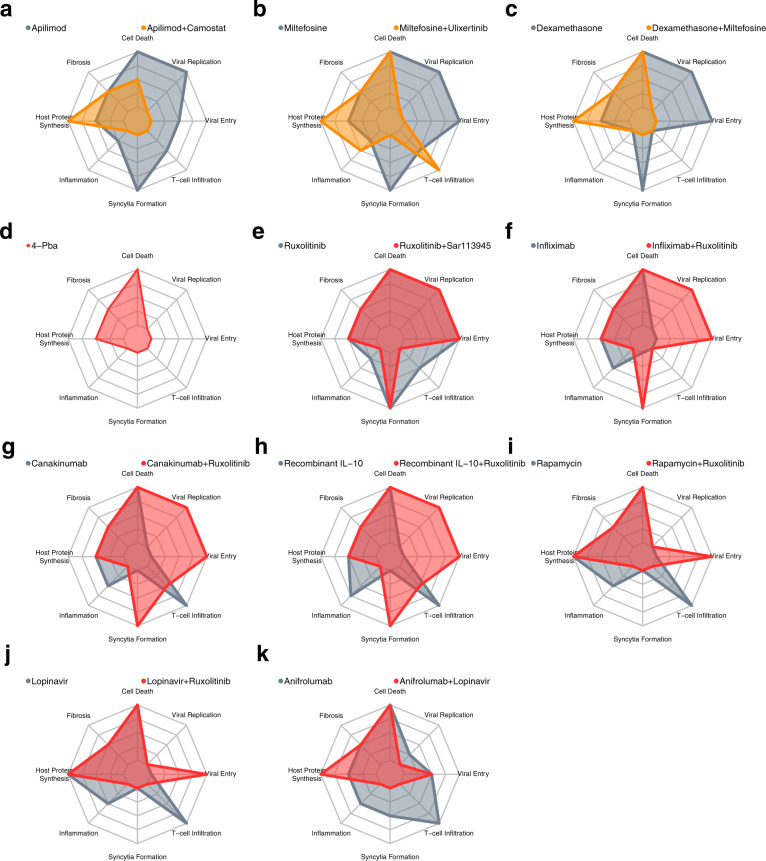
Predicted effective drug treatments for severe COVID-19. **a**–**c** The effect of treatments predicted to reduce viral replication in the early stage of severe COVID-19. Recommended therapies are shown in orange, with a representative control monotherapy shown in grey, with the strength of the biological process denoted by radial distance. **d**–**k** The effect of treatments predicted to reduce viral replication in the late stage of severe COVID-19. Recommended therapies are shown in red, with a representative control monotherapy shown in grey. All nodes normalised to the maximal level of respective nodes, and range between 0–100%.

At the presentation stage of severe COVID-19, characterised by a weak IFN-I/III response (Fig. 2b and Supplementary Table 3), treatments that are able to arrest viral replication before it can trigger an excessive inflammatory response are needed. As this stage occurs mainly prior to hospitalisation, these treatments must be taken as anticipatory therapy; at or close to the time of suspected exposure or infection. It is therefore critical that they do not adversely affect mild COVID-19 patients, as they will likely be administered before it is possible to stratify patients by prognosis. Our model predicts that, for example, consistent with known treatments in clinical use and trials, Remdesivir^35^ and recombinant Interferon α-2a (Roferon-A)^39^ are effective at reducing viral replication at the early stage of severe disease (Fig. 4a). In addition, several host translation inhibitors drawn from anti-cancer therapies were also shown to be effective by our model, including Homoharringtonine or Rapamycin (Fig. 4a). Homoharringtonine has previously been identified in a profile of potential drugs targeting SARS-CoV-2^41^, believed to act through inhibition of the eukaryotic small ribosomal subunit (60S)^42^, suppressing viral replication as the virus depends on host translation machinery for the production of viral proteins^43^. Homoharringtonine has cleared Phase II trials for chronic myeloid leukaemia^42^ and so is a good candidate to be fast-tracked for use. Similarly, Rapamycin and other mTOR inhibitors, which block translation, are in trials as post-exposure prophylaxis for COVID-19, to prevent the early stage of severe disease from progressing^44,45^. Our model predicts that Rapamycin is only effective against cap-dependent rather than cap-independent protein synthesis. However, as SARS-CoV-2 is dependent upon hijacking the cap-dependent form of protein synthesis^46^ this allows equal effectiveness in suppressing viral replication as with other translation inhibitors, with less effect on host protein synthesis and possibly fewer side effects (Fig. 5i). Other translation inhibitors drawn from anti-cancer therapies further demonstrate the potential of host-directed treatments^47,48^.

### Combination of Camostat and Apilimod suppress viral replication in early severe COVID-19

Our analysis further reveals options to combine different treatments, focusing on cases where there is an improvement in other pathological processes, in addition to decreasing viral replication. Viral entry inhibitors Camostat (TMPRSS2 inhibitor) and Apilimod (PIKfyve inhibitor) were shown to reduce viral entry but not viral replication in the model, whereas in combination they showed additional ability to prevent viral replication (Fig. 4a, b). This combination also showed improved effects on other key aspects of pathology compared to monotherapy (the effect of mono- vs combination therapy is shown in Fig. 5a for all simulated symptoms), recapitulating the effects of Apilimod seen in TMPRSS2-negative cell lines^49^. Miltefosine (AKT inhibitor) was shown in the model to be ineffective against viral replication alone but exhibits several promising combinations. A combination of Miltefosine with Ulixertinib (MAPK inhibitor) predicted effects across the redundant pathways controlling translation, leading to similar efficacy as Rapamycin (Fig. 4a, b). However, increased inflammation is also predicted as a side effect (Fig. 5b). Conversely, a combination of Miltefosine with the approved anti-inflammatory Dexamethasone was predicted by the model to target both viral replication and inflammation (Figs. 4b, 5c), if the ERK-inhibitory effects of Dexamethasone are sufficient to replace a dedicated inhibitor like Ulixertinib^50^.

### Reduction of ER stress using 4-PBA predicted to reduce excessive inflammation in late stage severe COVID-19

We next considered the late stage of the severe disease, as defined by an inappropriate inflammatory response that must be curbed (Fig. 2b and Supplementary Table 3). These treatments must be used with care, lest they reduce the useful antiviral immune response in the early severe or mild cases. As seen in the landmark RECOVERY trial^38^, Dexamethasone alone is a partially effective anti-inflammatory treatment for this stage of COVID-19 infection (Fig. 4c). Our model also predicts that sodium phenylbutyrate (4-PBA) may have some efficacy at this stage. Endoplasmic reticulum (ER) stress is dysregulated by the SARS-CoV spike protein (S)^51,52^. It has been suggested that this pathway plays a pro-inflammatory role, via activation of NFκB, in coronavirus infections in general^53,54^; preliminary results show similar effects in SARS-CoV-2^55,56^. 4-PBA is a chemical chaperone that helps reduce protein misfolding and so reduces ER stress (Figs. 4c, 5d). 4-PBA is already FDA approved for urea cycle disorders and is under consideration for use in cystic fibrosis, glioma and acute myeloid leukaemia^57,58^. However, this therapy is predicted by the model to slightly worsen viral replication in the mild case by decreasing IFN-II activity, and antiviral immunity (Supplementary Fig. 2h). While this change is small, it may prevent this therapy from being used prophylactically, but it can be safely applied in the late stage of severe disease, once viral replication has peaked, and viral load is waning. Similarly, the IFN-I blocking agent Anifrolumab decreases inflammation in the late stage of severe disease, but this has a counterproductive effect on viral replication in mild disease (Supplementary Fig. 2h).

### Antiviral Lopinavir predicted to suppress inflammation when used in combination with Ruxolitinib in late stage severe COVID-19

Ruxolitinib is already predicted to be effective as a monotherapy and is currently being trialled for COVID-19^40^. However, Ruxolitinib alone may, through inhibition of JAK-STAT and IFN-I signalling, impair innate immune-mediated suppression of viral entry and replication in mild cases (Fig. 3f and Supplementary Fig. 2g, h), and has no benefit in early severe disease (Fig. 3f and Supplementary Fig. 3d). While our model does not suggest combination treatments that can ameliorate this, meaning that Ruxolitinib is likely too risky to use in mild disease, we predict several ways to enhance the effects of Ruxolitinib appropriate for the late stage of severe disease. These include drugs that are ineffective alone such as SAR113945 (IKK inhibitor), Infliximab (TNF-α inhibitor), Canukinumab (IL-1B inhibitor) and recombinant IL-10 (Figs. 4c, d, 5e–j).

The trade-off of these combinations suggests that they should be used only in the late stage of the disease. For example, a combination of Rapamycin and Ruxolitinib is predicted to be more effective against inflammation than either drug alone. However, together these drugs are also predicted to increase viral entry (Fig. 5i). This may suggest that Rapamycin alone could be a good early monotherapy and, if the disease progresses, could be combined with Ruxolitinib in the late stage when a viral entry has already peaked. However, secondary infection, especially bacterial, could be a concern given the potent immunosuppressive effects of some of these combinations.

Lopinavir, often administered with Ritonavir, an agent that increases the half-life of Lopinavir in plasma, acts against the virus directly by blocking viral proteases, but has not demonstrated success in treating hospitalised COVID-19 patients^36,59^. This is consistent with our model’s prediction, which suggests that while Lopinavir may be effective at reducing viral replication in the early stages of severe COVID-19 (Fig. 4a), it is not predicted to provide additional benefit in the late stage of the disease (Supplementary Fig. 4g, h). However, while Lopinavir inhibits the 3CL protease of SARS-CoV-1^60^, it may only be effective against SARS-CoV-2 at toxic doses^41^ or not at all^61^ though dedicated 3CLPro inhibitors are in development^62^, including Paxlovid (PF-07321332)^63^. Lopinavir is one of the therapies currently being tested in the FLARE trial^64^, which may shed light on this.

Promisingly, targeting 3CL protease in our network model showed interesting anti-inflammatory effects, especially when used in combination with other drugs. 3CL protease is responsible for the proper processing of many viral proteins, including those involved in RNA-dependent RNA polymerase activity (RDRP). Without RDRP activity, the virus is unable to produce sgRNAs required for translation of structural and accessory proteins, including p3a and S, which have been shown to have a pro-inflammatory effect^55,56^. Direct targets of 3CLpro activity like viral nsp10 have also been shown to modulate the expression of pro-inflammatory cytokines^65^. This means that 3CL protease inhibition could potentially increase the effectiveness of anti-inflammatories such as Anifrolumab (IFNAR inhibitor) or Ruxolitinib (JAK1/2 inhibitor) (Figs. 4c, d, 5j, k). As such, anti-3CLpro drugs, such as Lopinavir or Paxlovid, may provide benefit even after viral replication has peaked by also playing a role in combination with other drugs to control dysregulated inflammation, and so can be beneficial in both phases of the disease. This contrasts with drugs such as Remdesivir, which blocks viral replication effectively by blocking RDRP but leaves other pro-inflammatory proteins intact. Consequently, our model predicts that Remdesivir will only be effective when applied early in the course of the disease. This is in line with clinical trials showing only modest effects when applied in patients who are already hospitalised^35^.

### Characteristic differences in cytokine level distinguish mild, early severe, and late severe COVID-19

As our model predictions show that the effect of drugs is dependent on the stage of the disease at which they are administered, we further searched using our network model for potential prognostic biomarkers for the different stages by comparing the steady-state levels of each node in mild, early, and late severe COVID-19. We observed that a characteristic signature of the early disease is a lower activity of cytokines, such as TNF-α, IL-6 and IL-10 and Interferons type I-III, compared to the mild case (Supplementary Fig. 5). These will subsequently rise in the late stage of the severe case, with some such as IL-6 exceeding the level seen in the mild case. This is in line with the evidence that severe disease arises, in part, due to an insufficient initial innate immune response, followed by a maladaptively strong and persistent inflammatory response and the prior identification of TNF-α, IL-6, IL-8, IL-10 and CXCL10 as prognostic markers for COVID-19 disease severity in hospitalised patients^33,66–69^. This suggests that measuring cytokine levels could help distinguish mild from severe cases in the early stages of the disease, but we also note the risk of an incorrect prognosis due to similarities between late severe and mild cases.

### Camostat and Apilimod combined have significantly greater effect suppressing viral entry and replication

**Fig. 6:**
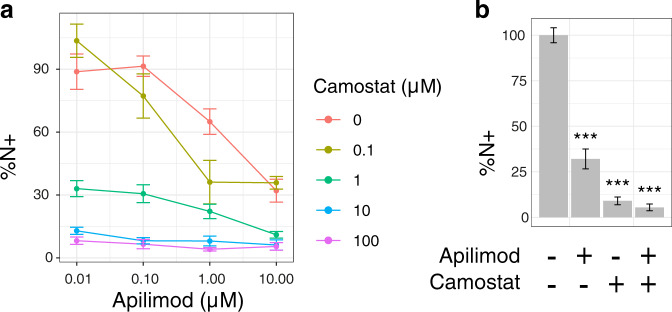
Combination of Camostat and Apilimod suppresses viral entry and replication more effectively than monotherapy. **a** Percentage cells expressing nucleocapsid protein relative to control for Apilimod and Camostat combinations. **b** Percentage cells expressing nucleocapsid protein for control, Apilimod (10 µM), Camostat (100 µM) or a combination of Apilimod (10 µM) and Camostat (100 µM). Data show the mean and standard error, from three independent experiments. *** indicates *p* < 0.001 by two-way ANOVA.

To validate our predictions, we selected the most promising combination treatments affecting viral replication identified from modelling early severe disease. Our model predicts that Camostat and Apilimod would have an enhanced effect on viral replication in combination compared to monotherapy (Supplementary Table 7). We tested this in Caco-2 cells and found that, as has been previously reported^70^, Camostat is effective at limiting viral entry leading to a reduction in SARS-CoV-2 nucleocapsid protein-positive cells in culture, but cannot completely suppress viral entry and replication (Fig. 6a). The addition of Apilimod significantly reduced SARS-CoV-2 infection further, even at the maximum dose of Camostat (Fig. 6b, *F* = 90.67, *p* = 2.8 × 10^−10^), in an additive manner (Supplementary Fig. 6). We further investigated whether the reported inhibitory effects of Dexamethasone on ERK activation^50^ were sufficient to synergise with Akt inhibition by Miltefosine (Supplementary Fig. 27) to exert direct antiviral effects, as this may provide extra benefit to Dexamethasone application early in disease progression. This builds on earlier findings that the combination of MAPK and PI3K pathway inhibitors is effective for MERS-CoV^71^, and inhibitors of the PI3K/Akt/mTOR pathway are effective against SARS-CoV-2^72,73^. However, this combination was ineffective except at doses of Miltefosine that showed toxicity (Supplementary Fig. 7d), potentially due to limited ERK inhibition by Dexamethasone in Caco-2 cells in vitro. This may be due to the slow nature of the effect of Dexamethasone on ERK, and so beneficial effects may not be evident in short-term cell-culture^50^.

## Discussion

Using our computational model, we can predict differential responses to therapy at different disease stages and explore the potential benefits of combination therapies over monotherapies for treating COVID-19 patients to identify the most effective combinations (Table 2). In this study, we have focussed on screening drugs that are clinically approved or in Phase II/III trials as these have the potential to immediately benefit patients in the current crisis and have had their efficacy validated in clinical practice. We further assume all drugs are maximally effective against all their putative targets to find the broadest array of potential therapies to guide experiments. A specific advantage of our computational approach is that we are able to screen a large number of potential drugs and combinations of drugs in a few hours. We have additionally screened for effective mono- and combination treatments for 36 more drugs of interest that are not yet approved or under trial (Supplementary Table 5 and Supplementary Figs. 8, 9). In addition, we have tested all possible hypothetical interventions against all the viral and host proteins in the network model – screening a total of over 9,000 combinations. These targets, if they can be made druggable, suggest potential novel viral or host-directed targets (Supplementary Figs. 10, 11). Together, the network model and the drug screening algorithm provide a valuable resource that can be leveraged by the scientific community to generate hypotheses about the effect of potential therapies on cellular processes and complement existing screens of monotherapies^74^.

**Table 2:** Summary of predicted effective treatments for early and late severe COVID-19.

	Predicted treatments to reduce viral replication in early stage severe COVID-19	Predicted treatments to reduce inflammation in advanced-stage severe COVID-19
Single drugs	Umifenovir Baricitinib Lopinavir Rapamycin Remdesivir Roferon-A	Dexamethasone (anti-inflammatory) 4-PBA (ER stress inhibitor)
Drug combinations	Camostat (TMPRSS2i) + Apilimod (PIKfyve inhibitor) Miltefosine (AKTi) + Ulixertinib (MAPKi)	Ruxolitinib (JAKi) + Recombinant IL-10 Ruxolitinib (JAKi) + Canakinumab (IL-1Bi) Ruxolitinib (JAKi) + Infliximab (TNF-α inhibitor) Ruxolitinib (JAKi) + SAR113945 (IKKi) Ruxolitinib (JAKi) + Rapamycin (mTORi) Ruxolitinib (JAKi) + Lopinavir (3CLpro inhibitor) Anifrolumab (IFNARi) + Lopinavir (3CLpro inhibitor)

Drug treatments predicted by the computational model to be effective in early or late stage severe COVID-19.

There are several computational approaches that have been applied to drug screening that are being applied to COVID-19, each with their own strengths and weaknesses. For example, as the structures of SARS-CoV-2 proteins have been determined, existing databases of small molecules have been leveraged to survey thousands of candidates to bind to these proteins^75^. Such studies helped identify 3CLpro as a common target, as well as the use of drugs such as Lopinavir and Remdesivir^26^. Another approach is the use of protein–protein interaction networks, which are particularly suited to identifying host-directed therapies^76,77^. These networks can be analysed for their structure alone and can cover a broad range of potential targets. These have been used to identify, for example, the potential for cancer drug repurposing^47,48^. However, static-network analyses rely on the assumption that certain features of the network structure can identify the best targets, e.g. proximity to disease-associated nodes, but they cannot predict specific effects. Moreover, they rely on pre-existing network databases or require additional curation^78,79^ or machine learning^80^ for new network types. Mathematical models such as Ordinary Differential Equations can interrogate the dynamics of the disease, but require precise data to fit model parameters, and so can only handle a smaller set of variables^81,82^.

By contrast, our approach combines scalable, executable modelling with transparent, biologically plausible explanations^30,83^ since each of our predictions is derived from biological interactions that can be explained in the context of the overall model, which itself is derived from, and consistent with, experimentally verified observations (Supplementary Table 1). The mechanistic explanations from our model advance the fight against the COVID-19 pandemic by increasing our understanding of why some treatments work and others fail. Our model and screening are readily accessible and are built using the BMA open-source and freely available toolset (https://biomodelanalyzer.org) and can be updated as new SARS-CoV-2 variants emerge that may exploit different mechanisms to enter cells and replicate or suppress host defence mechanisms^16^.

We deliberately chose to focus on the innate, rather than the adaptive immune response, specifically in lung epithelial cells. We further prioritised predicting the best targets for optimised drugs, rather than attempting to model the full pharmacodynamics of specific compounds. This allowed us to survey a broad selection of potential therapies for a critical cell type in the severe form of COVID-19. It also allowed us to focus specifically on two scenarios for the innate immune response: first, a low response seen in many patients^9–14^ that we believe characterises the early stage, and second, a persistent and excessive response in the late phase of the disease^17,84^.

We considered this broad scope appropriate at this stage of the pandemic, when there is a need for rapid development of new treatments, but without compromising safety. In the absence of computational screening of the kind we advocate, there has been a focus on only a small number of drugs^85,86^, many screened through expensive Phase III trials^86^ with high-profile safety concerns in the case of Hydroxychloroquine^87^, one of the most studied^85^. Our computational approach can help accelerate the process of screening more drugs and rejecting those with safety concerns, rapidly guiding clinicians to the most likely candidates.

Exemplifying this, amongst the most promising targets for early severe COVID-19 predicted by our model were Camostat and Apilimod, primarily targeting TMPRSS2 and PIKfyve respectively. We show that these drugs are significantly more effective together than alone in suppressing viral entry and replication in Caco-2 cells (Fig. 6). Combining these drugs effectively blocks the two key pathways for viral entry, via the cell membrane exploiting TMPRSS2 and via the endosomal route, without which the virus is severely limited in routes into the cell^70^. This novel combination builds on prior work blocking cathepsin directly^70,88^ and demonstrates its effectiveness with two phase II tested drugs. This combination also limits the range of host target cells the virus can infect. Camostat alone will only be effective in cells that allow TMPRSS2 entry, while the TMPRSS2-negative cell population also targeted by the virus remains permissive^89^. The addition of Apilimod may prevent this by targeting the endosomal entry pathway. While current variants of SARS-CoV-2 primarily exploit the TMPRSS2 entry route^90^, this may be beneficial if the virus evolves to exploit the endosomal route more aggressively. Host-directed therapies such as this and others suggested by the model may be more resistant to future mutations in SARS-CoV-2^31^.

Our computational model could be developed further to address patient stratification and correct dosing. Many treatments, including those we suggest, depend upon being administered at a specific stage of the disease, making it vital to accurately stratify patients, and necessarily includes factors outside the scope of our model, such as when it is practical to administer treatment. As an example, Remdesivir and Interferon α-2a (Roferon-A) have similar effects in the model, but Interferon α-2a may be significantly easier to administer as it is inhaled, and so may be more appropriate in the early case, compared to Remdesivir that requires intravenous administration. The likely side effects of treatments must also be considered, though it is hoped that treatment courses will be relatively short; Miltefosine with Ulixertinib is likely to produce diarrhoea and vomiting, which may be a severe burden in an unwell patient and pose aerosol risks to medical staff attending to them. Many of these adverse effects are driven by interaction outside the scope of the current model and this could be a fruitful area of expansion. Our network model helps address stratification by identifying potential biomarkers for different stages and severities of disease. It could also be extended to address dosing for both mono- and combination therapies. Finally, as we understand more about the progression of the disease and the set of traits that determine mild versus severe disease, the model could be expanded to better and, in more detail, explore the stages of severe COVID-19.

In this study, we have demonstrated that computational modelling has the ability to rapidly screen and predict new and improved therapies for previously unknown and life-threatening conditions. In particular, through this approach, we have listed several new combination therapies, based on existing and approved drugs, with the potential to improve outcomes for COVID-19 patients. The flexibility and transparency of mechanistic computational modelling will allow this work to be further developed as the virus evolves and demands further changes in therapeutic practice.

## Methods

### Qualitative networks

We model the viral–host interaction network as a discrete qualitative network^32^. Qualitative networks are an extension of Boolean networks^91^ in which each node may take multiple finite values in a fixed range (e.g. 0,1,2) rather than only ON and OFF. We build and analyse this network model using the open-source (MIT License) and freely available BioModelAnalyzer (BMA) tool^92^ https://biomodelanalyzer.org. The model is available at https://github.com/JFisherLab/COVID19.

The network consists of nodes representing viral proteins and RNA; host genes and proteins; processes such as viral entry; and phenotypes such as inflammation (see Supplementary Notes). The interactions between nodes are represented as edges (see Supplementary Table 1). The level of activity of a node is represented as a positive integer within a fixed range specific to that node. The level of activity changes in response to other nodes, as determined by a mathematical function associated with the node called a target function. The target function takes as input the level of neighbouring nodes that have an incoming edge to the node the target function controls. The default target function is *avg(pos)-avg(neg)*. This takes the mean of the level of activity of all the nodes with a positive edge (represented by an arrow (→)) and subtracts the mean of the level of activity of all the nodes with a negative edge (represented by a flat head (⊣)). More complex functions are needed to describe nodes with behaviour such as only activating when an input rises above a threshold. These target functions, and their rationale, are described in Supplementary Table 2. If node *X* has a granularity of *a-b* and is used in the target function of another node *X’* with range *a’-b’*, then it is scaled to the range of *X*′ using the equation:

1 $$\frac{{\left( {X - a} \right)\left( {b^{\prime} - a^{\prime}} \right)}}{{\left( {b - a} \right) + a^{\prime}}}$$

When presenting the values of nodes of differing ranges in the same plot (Figs. 3, 5 and Supplementary Figs. 1, 5, 12) we normalise the value of each node as a percentage of the maximum value of the node.

For a given set of initial values for all nodes, the network model will update all node values synchronously, and so there will be a single stable attractor the network will tend to. This attractor may be of any number of states, if it is one state we refer to it as a fixed-point attractor, if greater than one state we refer to it as a loop. We can test for whether the network reaches a fixed-point attractor for all possible initial states^93^. If there are different attractors for different initial states, we refer to this as a bifurcation. In the case of a fixed point, we take the level of every node in the attractor as the prediction of biological behaviour, in the case of a loop or a bifurcation we use the mid-point of the upper and lower bound that can be placed upon node behaviour using the algorithm described in Cook et al.^93^. Details of the procedure of network simulation can be found in Schaub et al.^32^ and the bounds and reported mid-point are given in Supplementary Data S1.

### Model testing

We built the network using a bottom-up iterative approach. We collated data from the literature describing interactions between and within SARS-CoV-2 and the host cell. The experimental evidence for each edge is described in Supplementary Table 1. We then collated a separate set of testing data used to evaluate the model. This was formed from published data on the effect of drugs on SARS-CoV-2 in culture and is summarised in Supplementary Table 4. These data describe experiments that do not define a point-to-point interaction between genes and/or proteins, but rather the overall behaviour of the system that emerges from the sum of the interactions and so provides a testing dataset for the model separate from the data used to build it.

In order to reproduce an experimental condition in the model, we set the target function of the relevant node to a constant value. For example, the presence of SARS-CoV-2 is represented by changing the target function of *Virion* to 1. We then find the stable states of the model in this case and compare the predicted stable value of nodes for which there are data (e.g. inflammation or level of IL-6) to that observed in the experiment. We iteratively tested the model and based on these tests, expanded and refined the model until it matched all the observed behaviours, as described in Supplementary Table 4.

### In silico drug combination screens

In order to find the most effective treatments, we inactivate (set target function to a minimum) or activate (set target function to maximum) all nodes, or all sets of nodes targeted by a corresponding drug (Supplementary Table 5) either singly or in a pair-wise combination, using the BMA Command Line tool BioCheckConsole. We consider all combinations of nodes and all combinations of drugs, where a single drug may target multiple nodes. This assumes that drugs are able to act on all their putative targets and does not account for other pharmacodynamic effects that may reduce their efficacy. For each node in the network, we manually curated from the literature a list of drugs that could target nodes within the network, finding 74 candidate drugs (Supplementary Table 5), of which 38 had passed phase II trials at the time of writing (Table 1). We also found all the reported targets for these drugs using the DrugBank database^94^ to ensure all targets included in the model are direct and clinically relevant. We evaluate these perturbations by the stable state of the network for the eight phenotypes of interest, or in the case that the steady-state could not be found, the mid-point of the upper and lower bounds BMA could place on the behaviour of that node using the algorithm described in Cook et al.^93^. We compare different stages of the disease by using different backgrounds; setting certain nodes to a constant level to reproduce the mild, early severe, and late severe forms of the disease (see Supplementary Notes, Supplementary Table 3 and Supplementary Data 1).

### Cell and virus cultures

Caco-2 cells were a kind gift to Dr. Dalan Bailey (Pirbright Institute). Cells were cultured in Dulbecco’s Modified Eagle Medium (DMEM) supplemented with 10% heat-inactivated FBS (Labtech), 100 U/ml penicillin/streptomycin. For infections, cells were seeded at 0.2 × 10^6^ cells/ml. Stocks of SARS-CoV-2 strain BetaCoV/Australia/VIC01/2020 (NIBSC) were generated by propagation on Caco-2 cells and virus titres determined by RT-qPCR for viral E RNA copies as described previously^17^.

### Inhibitor treatment and infections

Caco-2 cells were pre-treated with Camostat Mesilate (ApexBio, B2082), Apilimod (Selleckchem, S6414), Miltefosine (Cayman Chemicals, 63280), Dexamethasone (EMD Millipore, 265005) or DMSO (Sigma) vehicle control at 2x the final concentration for 2 h prior to infection in 50 μl culture medium. Cells were infected with 1000 E copies/cell SARS-CoV-2 in 50 μl, bringing the total culture volume to 100 μl and inhibitors to 1x final concentrations as indicated. Cytotoxicity of inhibitors was determined by tetrazolium salt (MTT) assay. About 10% MTT was added to culture media and cells were incubated for 24 h at 37 °C. Cells were lysed with 10% SDS, 0.01 M HCl and the formation of purple formazan was measured at 570 nm.

### Flow cytometry

Infection levels were measured at 24 h by flow cytometry. Caco-2 cells were trypsinised, stained with fixable Zombie UV Live/Dead dye (BioLegend) and fixed with 4% PFA before intracellular staining for nucleocapsid protein. For intracellular detection of SARS-CoV-2 nucleoprotein, cells were permeabilised for 15 min with Intracellular Staining Perm Wash Buffer (BioLegend). Cells were then incubated with 1 μg/ml CR3009 SARS-CoV-2 cross-reactive antibody (a kind gift from Dr. Laura McCoy) in permeabilisation buffer for 30 min at room temperature, washed once and incubated with secondary Alexa Fluor 488-Donkey-anti-Human IgG (Jackson Labs). All samples were acquired and analysed using a NovoCyte (Agilent) and NovoExpress 1.5.0 software (Agilent). Supplementary Fig. 13 shows the gating strategy applied to a representative sample of Caco-2 cells.

### Calculation of drug combination indices

The expression of intracellular SARS-CoV-2 nucleocapsid protein was measured by flow cytometry at 24 h post infection and used as a measure of drug effect on viral entry and replication. The data were normalised to the value of the control group and averaged across nine replicates. For each treatment, the combined effect of drug 1 at concentration *a* and drug 2 at concentration *b* was calculated as $E_{a,b} = (100 - N_{a,b})/100$, where $N_{a,b}$ is the average percentage of cells expressing nucleocapsid protein at this concentration. The combination index (CI) was then calculated according to the Bliss independence model^95,96^:

2 $${{CI}} = \frac{{E_{a,0} + E_{0,b} - (E_{a,0} \times E_{0,b})}}{{E_{a,b}}}$$

When ${\mathrm{CI}}\, <\, 1$, this indicates synergy between the drugs at this concentration, while ${\mathrm{CI}} \approx 1$ indicates additivity and ${\mathrm{CI}}\, >\, 1$ indicates antagonism.

Plots were generated using R^97^ and the pheatmap^98^, fmsb^99^, RColorBrewer^100^, ggplotify^101^ and the tidyverse packages^102^.

### Reporting Summary

Further information on research design is available in the Nature Research Reporting Summary linked to this article.

## Supplementary information

Supplementary Materials

Dataset 1

REPORTING SUMMARY

## Data Availability

Authors can confirm that all relevant data are included in the paper and/or its supplementary information files. The model is available at https://github.com/JFisherLab/COVID19.

## Code availability

Custom code used to analyse the model is available upon reasonable request.

## References

[1] Park A, Iwasaki A (2020). Type I and Type III interferons – Induction, signaling, evasion, and application to combat COVID-19. Cell Host Microbe.

[2] Ellul MA (2020). Neurological associations of COVID-19. Lancet Neurol..

[3] Jiang L (2020). COVID-19 and multisystem inflammatory syndrome in children and adolescents. Lancet Infect. Dis..

[4] Connors JM, Levy JH (2020). COVID-19 and its implications for thrombosis and anticoagulation. Blood.

[5] Estimated Disease Burden of COVID-19 | CDC. https://www.cdc.gov/coronavirus/2019-ncov/cases-updates/burden.html.

[6] Reese H (2021). Estimated incidence of coronavirus disease 2019 (COVID-19) illness and hospitalization—United States, February–September 2020. Clin. Infect. Dis..

[7] Liu C (2021). Time-resolved systems immunology reveals a late juncture linked to fatal COVID-19. Cell.

[8] Cheemarla NR (2021). Dynamic innate immune response determines susceptibility to SARS-CoV-2 infection and early replication kinetics. J. Exp. Med..

[9] Lucas C (2020). Longitudinal analyses reveal immunological misfiring in severe COVID-19. Nature.

[10] Zhang Q (2020). Inborn errors of type I IFN immunity in patients with life-threatening COVID-19. Science.

[11] Laing AG (2020). A dynamic COVID-19 immune signature includes associations with poor prognosis. Nat. Med..

[12] Bastard P (2020). Autoantibodies against type I IFNs in patients with life-threatening COVID-19. Science.

[13] Pairo-Castineira E (2021). Genetic mechanisms of critical illness in COVID-19. Nature.

[14] Combes AJ (2021). Global absence and targeting of protective immune states in severe COVID-19. Nature.

[15] Hadjadj J (2020). Impaired type I interferon activity and inflammatory responses in severe COVID-19 patients. Science.

[16] Thorne, L. G. et al. Evolution of enhanced innate immune evasion by SARS-CoV-2. *Nature.*10.1038/s41586-021-04352-y (2021). 10.1038/s41586-021-04352-y PMC8850198 34942634

[17] Thorne LG (2021). SARS-CoV-2 sensing by RIG-I and MDA5 links epithelial infection to macrophage inflammation. EMBO J..

[18] Lin S-H, Zhao Y-S, Zhou D-X, Zhou F-C, Xu F (2020). Coronavirus disease 2019 (COVID-19): cytokine storms, hyperinflammatory phenotypes, and acute respiratory distress syndrome. Genes Dis.

[19] Propper C, Stoye G, Zaranko B (2020). The wider impacts of the coronavirus pandemic on the NHS. Fisc. Stud..

[20] Greenberg N (2021). Mental health of staff working in intensive care during Covid-19. Occup. Med..

[21] Voysey M (2021). Safety and efficacy of the ChAdOx1 nCoV-19 vaccine (AZD1222) against SARS-CoV-2: an interim analysis of four randomised controlled trials in Brazil, South Africa, and the UK. Lancet.

[22] Polack FP (2020). Safety and efficacy of the BNT162b2 mRNA covid-19 vaccine. N. Engl. J. Med..

[23] Baden LR (2021). Efficacy and Safety of the mRNA-1273 SARS-CoV-2 Vaccine. N. Engl. J. Med..

[24] Anderson RM, Vegvari C, Truscott J, Collyer BS (2020). Challenges in creating herd immunity to SARS-CoV-2 infection by mass vaccination. Lancet.

[25] Jeyanathan M (2020). Immunological considerations for COVID-19 vaccine strategies. Nat. Rev. Immunol..

[26] Galindez G (2021). Lessons from the COVID-19 pandemic for advancing computational drug repurposing strategies. Nat. Comput. Sci..

[27] Chuang R (2015). Drug target optimization in chronic myeloid leukemia using innovative computational platform. Sci. Rep..

[28] Silverbush D (2017). Cell-specific computational modeling of the PIM pathway in acute myeloid leukemia. Cancer Res..

[29] Kreuzaler P (2019). Heterogeneity of Myc expression in breast cancer exposes pharmacological vulnerabilities revealed through executable mechanistic modeling. Proc. Natl Acad. Sci. USA.

[30] Clarke MA, Fisher J (2020). Executable cancer models: successes and challenges. Nat. Rev. Cancer.

[31] Kaufmann SHE, Dorhoi A, Hotchkiss RS, Bartenschlager R (2018). Host-directed therapies for bacterial and viral infections. Nat. Rev. Drug Discov..

[32] Schaub MA, Henzinger TA, Fisher J (2007). Qualitative networks: a symbolic approach to analyze biological signaling networks. BMC Syst. Biol..

[33] Arunachalam PS (2020). Systems biological assessment of immunity to mild versus severe COVID-19 infection in humans. Science.

[34] Broggi A (2020). Type III interferons disrupt the lung epithelial barrier upon viral recognition. Science.

[35] Beigel JH (2020). Remdesivir for the treatment of covid-19 — Final report. N. Engl. J. Med..

[36] Cao B (2020). A trial of lopinavir–ritonavir in adults hospitalized with severe covid-19. N. Engl. J. Med..

[37] RECOVERY Collaborative Group. (2021). Tocilizumab in patients admitted to hospital with COVID-19 (RECOVERY): a randomised, controlled, open-label, platform trial. Lancet.

[38] The RECOVERY Collaborative Group. (2021). Dexamethasone in hospitalized patients with covid-19. N. Engl. J. Med..

[39] ClinicalTrials.gov. US National Library of Medicine. https://clinicaltrials.gov/ct2/show/NCT04293887 (2020).

[40] Cao Y (2020). Ruxolitinib in treatment of severe coronavirus disease 2019 (COVID-19): a multicenter, single-blind, randomized controlled trial. J. Allergy Clin. Immunol..

[41] Choy KT (2020). Remdesivir, lopinavir, emetine, and homoharringtonine inhibit SARS-CoV-2 replication in vitro. Antivir. Res.

[42] Winer ES, DeAngelo DJ (2018). A review of omacetaxine: a chronic myeloid leukemia treatment resurrected. Oncol. Ther..

[43] de Breyne S (2020). Translational control of coronaviruses. Nucleic Acids Res..

[44] ClinicalTrials.gov. US National Library of Medicine. https://clinicaltrials.gov/ct2/show/NCT04461340.

[45] ClinicalTrials.gov. US National Library of Medicine. https://clinicaltrials.gov/ct2/show/NCT04584710.

[46] Romano M, Ruggiero A, Squeglia F, Maga G, Berisio R (2020). A structural view of SARS-CoV-2 RNA replication machinery: RNA synthesis, proofreading and final capping. Cells.

[47] White KM (2021). Plitidepsin has potent preclinical efficacy against SARS-CoV-2 by targeting the host protein eEF1A. Science.

[48] Varona JF (2022). Preclinical and randomized phase I studies of plitidepsin in adults hospitalized with COVID-19. Life Sci. Alliance.

[49] Ou X (2020). Characterization of spike glycoprotein of SARS-CoV-2 on virus entry and its immune cross-reactivity with SARS-CoV. Nat. Commun..

[50] Kassel O (2001). Glucocorticoids inhibit MAP kinase via increased expression and decreased degradation of MKP-1. EMBO J..

[51] Chan C-P (2006). Modulation of the unfolded protein response by the severe acute respiratory syndrome coronavirus spike protein. J. Virol..

[52] Versteeg GA, van de Nes PS, Bredenbeek PJ, Spaan WJM (2007). The coronavirus spike protein induces endoplasmic reticulum stress and upregulation of intracellular chemokine mRNA concentrations. J. Virol..

[53] Fung TS, Liu DX (2014). Coronavirus infection, ER stress, apoptosis and innate immunity. Front. Microbiol..

[54] Fung TS, Liu DX (2019). Human coronavirus: host-pathogen interaction. Annu. Rev. Microbiol..

[55] Hsu, A. C.-Y. et al. SARS-CoV-2 Spike protein promotes hyper-inflammatory response that can be ameliorated by Spike-antagonistic peptide and FDA-approved ER stress and MAP kinase inhibitors in vitro. Preprint at *bioRxiv*10.1101/2020.09.30.317818 (2020).

[56] Zhang, X. et al. Sequential ER stress and inflammatory responses are induced by SARS-CoV-2 ORF3 through ERphagy. Preprint at *bioRxiv*10.1101/2020.11.17.387902 (2020).

[57] Kolb PS (2015). The therapeutic effects of 4-phenylbutyric acid in maintaining proteostasis. Int. J. Biochem. Cell Biol..

[58] Iannitti T, Palmieri B (2011). Clinical and experimental applications of sodium phenylbutyrate. Drugs R. D..

[59] Horby PW (2020). Lopinavir–ritonavir in patients admitted to hospital with COVID-19 (RECOVERY): a randomised, controlled, open-label, platform trial. Lancet.

[60] Wu CY (2004). Small molecules targeting severe acute respiratory syndrome human coronavirus. Proc. Natl Acad. Sci. USA.

[61] Jang, M. et al. Lopinavir-ritonavir is not an effective inhibitor of the main protease activity of SARS-CoV-2 in vitro. Preprint at *bioRxiv*10.1101/2020.09.16.299800 (2020).

[62] Qiao J (2021). SARS-CoV-2 Mpro inhibitors with antiviral activity in a transgenic mouse model. Science.

[63] ClinicalTrials.gov. US National Library of Medicine. https://clinicaltrials.gov/ct2/show/NCT04960202 (2021).

[64] ClinicalTrials.gov. US National Library of Medicine. https://clinicaltrials.gov/ct2/show/NCT04499677 (2020).

[65] Li J (2021). Virus-host interactome and proteomic survey reveal potential virulence factors influencing SARS-CoV-2 pathogenesis. Med.

[66] Liu J (2020). Longitudinal characteristics of lymphocyte responses and cytokine profiles in the peripheral blood of SARS-CoV-2 infected patients. EBioMedicine.

[67] Tan M (2020). Immunopathological characteristics of coronavirus disease 2019 cases in Guangzhou, China. Immunology.

[68] Chen Y (2020). Blood molecular markers associated with COVID‐19 immunopathology and multi‐organ damage. EMBO J..

[69] Del Valle DM (2020). An inflammatory cytokine signature predicts COVID-19 severity and survival. Nat. Med..

[70] Hoffmann M (2020). SARS-CoV-2 cell entry depends on ACE2 and TMPRSS2 and is blocked by a clinically proven protease inhibitor. Cell.

[71] Kindrachuk J (2015). Antiviral potential of ERK/MAPK and PI3K/AKT/mTOR signaling modulation for Middle East respiratory syndrome coronavirus infection as identified by temporal kinome analysis. Antimicrob. Agents Chemother..

[72] Garcia G (2021). Antiviral drug screen identifies DNA-damage response inhibitor as potent blocker of SARS-CoV-2 replication. Cell Rep.

[73] Dittmar M (2021). Drug repurposing screens reveal cell-type-specific entry pathways and FDA-approved drugs active against SARS-Cov-2. Cell Rep..

[74] Bakowski MA (2021). Drug repurposing screens identify chemical entities for the development of COVID-19 interventions. Nat. Commun..

[75] Wu C (2020). Analysis of therapeutic targets for SARS-CoV-2 and discovery of potential drugs by computational methods. Acta Pharm. Sin. B.

[76] Gordon DE (2020). A SARS-CoV-2 protein interaction map reveals targets for drug repurposing. Nature.

[77] Bouhaddou M (2020). The global phosphorylation landscape of SARS-CoV-2 infection. Cell.

[78] Ostaszewski M (2020). COVID-19 disease map, building a computational repository of SARS-CoV-2 virus-host interaction mechanisms. Sci. Data.

[79] Verstraete N (2020). CovMulNet19, integrating proteins, diseases, drugs, and symptoms: a network medicine approach to COVID-19. Netw. Syst. Med.

[80] Richardson P (2020). Baricitinib as potential treatment for 2019-nCoV acute respiratory disease. Lancet.

[81] Jenner AL (2021). COVID-19 virtual patient cohort suggests immune mechanisms driving disease outcomes. PLOS Pathog..

[82] Dogra P (2021). Innate immunity plays a key role in controlling viral load in COVID-19: mechanistic insights from a whole-body infection dynamics model. ACS Pharmacol. Transl. Sci..

[83] Fisher J, Henzinger TA (2007). Executable cell biology. Nat. Biotechnol..

[84] Lucas C (2021). Delayed production of neutralizing antibodies correlates with fatal COVID-19. Nat. Med..

[85] Jones CW, Woodford AL, Platts-Mills TF (2020). Characteristics of COVID-19 clinical trials registered with ClinicalTrials.gov: cross-sectional analysis. BMJ Open.

[86] Fragkou PC (2020). Review of trials currently testing treatment and prevention of COVID-19. Clin. Microbiol. Infect..

[87] Ghazy RM (2020). A systematic review and meta-analysis on chloroquine and hydroxychloroquine as monotherapy or combined with azithromycin in COVID-19 treatment. Sci. Rep..

[88] Yousefi H, Mashouri L, Okpechi SC, Alahari N, Alahari SK (2021). Repurposing existing drugs for the treatment of COVID-19/SARS-CoV-2 infection: A review describing drug mechanisms of action. Biochem. Pharmacol..

[89] Jackson, C. B., Farzan, M., Chen, B. & Choe, H. Mechanisms of SARS-CoV-2 entry into cells. *Nat. Rev. Mol. Cell Biol*. 10.1038/s41580-021-00418-x (2021). 10.1038/s41580-021-00418-x PMC8491763 34611326

[90] Ozono S (2021). SARS-CoV-2 D614G spike mutation increases entry efficiency with enhanced ACE2-binding affinity. Nat. Commun..

[91] Bornholdt S (2008). Boolean network models of cellular regulation: prospects and limitations. J. R. Soc. Interface.

[92] Benque, D. et al. BMA: visual tool for modeling and analyzing biological networks. *Comput. Aided Verif*. 686–692 (2012).

[93] Cook B, Fisher J, Krepska E, Piterman N (2011). Proving stabilization of biological systems. Lect. Notes Comput. Sci. (including Subser. Lect. Notes Artif. Intell. Lect. Notes Bioinforma.).

[94] Wishart DS (2006). DrugBank: a comprehensive resource for in silico drug discovery and exploration. Nucleic Acids Res.

[95] Foucquier J, Guedj M (2015). Analysis of drug combinations: current methodological landscape. Pharmacol. Res. Perspect.

[96] Bliss CI (1939). The toxicity of poisons applied jointly. Ann. Appl. Biol..

[97] R Core Team. R: a language and environment for statistical computing. (2017).

[98] Kolde, R. pheatmap: pretty heatmaps. (2015).

[99] Nakazawa, M. fmsb: functions for medical statistics book with some demographic data. (2021).

[100] Neuwirth, E. RColorBrewer: ColorBrewer palettes. (2014).

[101] Yu, G. ggplotify: convert plot to ‘grob’ or ‘ggplot’ object. (2021).

[102] Wickham H (2019). Welcome to the Tidyverse. J. Open Source Softw.

