# Supplementary material for: Executable network of SARS-CoV-2-host interaction predicts drug combination treatments
Source: NPJ Digit Med. 2022 Feb 14;5:18. doi: 10.1038/s41746-022-00561-5 (PMC8844383; doi:10.1038/s41746-022-00561-5)
Supplement: Supplementary file 1 — Supplementary Materials [file 41746_2022_561_MOESM1_ESM.pdf]

Supplementary Materials

Executable Network of SARS-CoV-2-Host Interaction Predicts Drug Combination Treatments

Rowan Howell[1†], Matthew A. Clarke[1†], Ann-Kathrin Reuschl[2†], Tianyi Chen[1], Sean Abbott-Imboden[1], Mervyn Singer[3], David M. Lowe[4], Clare L. Bennett[1,4], Benjamin Chain[2,5], Clare Jolly[2*], Jasmin Fisher[1*]

[1]UCL Cancer Institute, University College London, 72 Huntley Street, London WC1E 6DD, UK
[2]Division of Infection and Immunity, University College London, London WC1E 6BT, UK
[3]Bloomsbury Institute of Intensive Care Medicine, Division of Medicine, University College London, London WC1E 6BT, UK
[4]Institute of Immunity and Transplantation, University College London, London NW3 2PF, UK
[5]Department of Computer Science, Gower Street, University College London, London WC1E 6BT, UK

[†] These authors contributed equally to this work

[*] Correspondence should be addressed to Jasmin Fisher jasmin.fisher@ucl.ac.uk or Clare Jolly c.jolly@ucl.ac.uk

Supplementary Materials Contents

Supplementary Notes………3

Supplementary Figure 1. Effect of drug combinations identified by in silico screening……………………..13

Supplementary Figure 2. Effects of single and combination therapies (38 drugs that have passed phase II trials) in mild COVID-19……………………………………………………………………………………………………..14

Supplementary Figure 3. Effects of single and combination therapies (38 drugs that have passed phase II trials) in early stage, severe COVID-19………………………………………………………………………………..23

Supplementary Figure 4. Effects of single and combination therapies (38 drugs that have passed phase II trials) in late stage, severe COVID-19………………………………………………………………………………...32

Supplementary Figure 5. Differential characteristics of mild, early severe and late severe COVID-19..41

Supplementary Figure 6. Camostat and Apilimod inhibit replication of SARS-COV-2 in Caco-2 cells…..42

Supplementary Figure 7. The impact of Dexamethasone and Miltefosine on replication of SARS-CoV-2 in Caco-2 cells……..43

Supplementary Figure 8. Effects of single and combination therapies (74 drugs, including those that have not passed phase II trials) in early stage, severe COVID-19……………………………………………………..44

Supplementary Figure 9. Effects of single and combination therapies (74 drugs, including those that have not passed phase II trials) in late stage, severe COVID-19………………………………………………………53

Supplementary Figure 10. Effects of single and combination therapies (all nodes) in early stage, severe COVID-19………..62

Supplementary Figure 11. Effects of single and combination therapies (all nodes) in late stage, severe COVID-19………..72

Supplementary Figure 12. Genetic risk factors associated with severe COVID-19……………………………..82

Supplementary Figure 13. Gating strategy used to identify expression of N protein and viability in Caco-2 cells………..83

Supplementary Table 1. Regulatory edges in the network model………………………………………………..84

Supplementary Table 2. Target functions used in the network model……………………………………………89

Supplementary Table 3. Constraints applied for each state of the network model……………………………91

Supplementary Table 4. Experiments from the literature used to validate the network model…………92

Supplementary Table 5. Drugs used for in silico drug repurposing screens………………………………………93

Supplementary Table 6. Classification of nodes in the network model…………………………………………..94

Supplementary Table 7. In silico simulation of Caco-2 cell drug treatments……………………………………98

Supplementary References…………………………………………………………………………………………………99

Supplementary Data 1. Results of in silico screens for the eight phenotypes in mild, early and late severe COVID-19 cases……………………………………………………………………See File: Supplementary_Data_1.xlsx

Supplementary Notes

We developed an executable model of SARS-CoV-2 infection in lung epithelial cells in order to explore combination therapies targeting the virus and host proteins. In this supplementary information we will begin by explaining how the model was constructed and the disease states it models. We will then describe in detail how the host and viral pathways were modelled. Next, we describe how the model was validated against *in vitro* experiments from the literature and clinical trials. Finally, we will discuss how, using the model, we performed *in silico* screening to identify drug combinations to treat SARS-CoV-2 infection.

Modelling the SARS-CoV-2 infection regulatory network

Reviewing 100 publications describing SARS-CoV-2, SARS-CoV-1 and pan-viral disease mechanisms, we constructed a Qualitative Network model[1] using the BioModelAnalyzer[2] tool (Figure 2a, Supplementary Table 1, Supplementary References). These publications were reviewed to ensure that they were: relevant to our tissue of interest, lung epithelium; relevant to our disease cases, mild, early and late severe; and to ensure that there were no contradictory findings from other studies. This computational network model represents the regulatory network underlying SARS-CoV-2 infection in lung epithelium. The network consists of 175 nodes representing 36 viral proteins and complexes, 102 host proteins and complexes, 31 cellular processes, 5 RNA species and a node controlling the strength of innate immune response (Supplementary Table 6). The nodes representing cellular processes calculate the combined action of the individual proteins that regulate and enact these processes, which have their own nodes. For example, the *ViralFusion* process node represents the activity of the *TMPRSS2*, *CathepsinL* and *TPC2* nodes. This allows us to model the effect on Viral Fusion of separate or combined inhibition of the proteins involved in this process. Of the 31 cellular processes, 8 are considered to be clinically relevant biological processes, which are used to monitor COVID-19 patients' response to treatment. These nodes are connected by edges that can be either activating (→) or inhibiting (⊣) derived from published experimental evidence (Supplementary Table 1). Separately, we collated a set of test data from the literature based on responses of SARS-CoV-2 infected cells to treatment (Supplementary Table 4). This allowed us to iteratively test, expand and refine the model by comparing the model predictions to this dataset of known responses. The testing process is described in more detail below.

Each node in the network holds an integer value between 0 and its maximum range, which is 2 for most nodes, and 4 for some symptom nodes and cytokines. The value of each node represents its level of activity, for example when *CCL2* takes a value of 0 it is inactive and when it is at 4 it is at its most active. The level of each node is determined by the target function, with the inputs being the activity of incoming nodes. The default target function gives a level of activity that is the average of the activators of the node minus the average of the inhibitors. More complex target functions can be specified, which can be more complex e.g., to model an interaction that is inhibiting in one context but activating at another (Supplementary Table 2).

There are eight phenotypic readout nodes in the model: *Viral Entry*, *Viral Replication*, *Inflammation*, *Fibrosis*, *Cell Death*, *T-cell Infiltration*, *Syncytia Formation* and *Host Protein Synthesis*. The *Viral Entry* node represents the ability of the virus to enter the cell, while *Viral Replication* represents the production of new virions which can go on to infect other cells. *Inflammation* represents the summed effect of pro-inflammatory cytokines, minus the anti-inflammatory cytokines. *Fibrosis* represents the production of α-collagen in the lungs, leading to lung scarring. *Cell Death* represents the level of death in a population of cells via apoptosis, necroptosis or pyroptosis. *T-cell Infiltration* represents the

movement of T-cells into the lungs, which is suspected to be the cause of T-cell lymphopenia seen in COVID-19 patients. *Syncytia Formation* represents the formation of cell-cell fusions seen in populations of infected cells. *Host Protein Synthesis* represents the ability of the cell to produce its own proteins.

There are three key input nodes to the model, which are used to describe the condition modelled. The infected state of the model is controlled by the *Virion* node, representing the presence of viral particles in the lungs. A node called *Immune Cells* is used to distinguish the presence of immune cells in tissue from epithelial cell lines, and so allows the comparison of the model to results from pure epithelial cell lines, but also *in vivo* data where immune cells and their reaction to the epithelial cells is recorded. The strength of the innate IFN-I and IFN-III response is thought to be critical to the progression and severity of COVID-19[3]. The *Sensitivity of IFN Response* node is used to convey both the response to, and magnitude of, IFN-I/III production in the range low, medium, high (0, 1, 2). All other unregulated nodes in the model represent constitutively active proteins.

States of the model

The SARS-CoV-2 infection model can stabilise in one of four states (Figure 2b):

- Uninfected
- Mild COVID-19 disease
- Early stage, severe COVID-19 disease
- Late stage, severe COVID-19 disease

Each of these states is specified by a combination of constraints applied to the model. We describe each of these states and their constraints below.

The **uninfected** state is controlled by setting the *Virion* node to be 0. In this state, all symptom nodes take value 0 (with the exception of *Host Protein Synthesis*, which is 2), representing the levels seen in healthy cells. This state is used as a control to compare with the infected states.

The **mild COVID-19** state represents a patient that is able to mount a robust innate immune response to SARS-CoV-2 infection, and as a result is able to limit Viral Replication in the lungs. This is controlled by setting the *Sensitivity of IFN Response* node to be 1, allowing a normal level of IFN induction in response to infection. In this state, both *Viral Replication* and *Viral Entry* are reduced relative to the early stage of the severe case, as some Interferon Stimulated Genes (ISGs)[4], such as ZAP and OAS, cause degradation of the viral genome[5], while others, such as CH25H[6] and LY6E[7] are capable of inhibiting entry of the virus into the cell. The *Inflammation* node reaches level 2, driven by both IFN-dependent and virus-dependent pathways. This is considered to be a control case, as such patients do not require medical intervention. However, it is not currently possible to distinguish between mild and severe cases in the early stages of the disease, so any treatment proposed to treat severe cases must be shown not to be detrimental to the mild response, such as by inhibiting beneficial IFN response. Those drugs used must also not have such severe side-effects that use in the mild case would be harmful, but predicting side-effects beyond the effects on the eight modelled phenotypes in lung epithelium is outside the scope of the model.

The **early stage, severe COVID-19** case represents patients who are unable to mount a robust IFN response and so are in the early stages of a disease progression that will become severe, but who may still have mild symptoms at presentation. In this state viral replication proceeds rapidly in the lungs. This state is controlled by setting the *Sensitivity of IFN Response* to 0. In this state *Viral Replication* and *Viral Entry* reach their maximum value of 4. *Inflammation* reaches level 1 in the absence of IFN-

dependent inflammation. In this case we search for treatments to limit the spread of infection by reducing the *Viral Replication* node.

There is a growing body of evidence that, although SARS-CoV-2 initially suppresses the IFN response, late stages of infection are characterised by high levels of IFN signalling[8], corresponding to the **late stage**, **severe COVID-19** case. This condition is modelled by setting the *Sensitivity of IFN Response* node and the *Viral Genome* node to 2. Setting *Sensitivity of IFN Response* to 2 leads to high levels of IFN induction, which totally dampen both *Viral Replication* and *Viral Entry* but also lead to Inflammation reaching 3. We set *Viral Genome* to 2 to represent that significant viral replication has already occurred by this stage of the disease and so there is a burden of viral RNA present even though replication has peaked. In this case we look for treatment to lower the *Inflammation* node, as this is the most dangerous aspect of this stage of the disease.

Key cellular processes and signalling represented in the model

Our model focuses on the following key aspects of SARS-CoV-2 replication and pathogenesis:

Viral entry: A cell becomes infected when a SARS-CoV-2 virion binds to the cell through interaction between membrane-bound ACE2 and the spike protein[9–11]. Once bound, the virus enters the cell using host factors TMPRSS2, cathepsinL and TPC2. Upon viral entry, the virus introduces the viral genome, wrapped in the nucleocapsid (N) protein, to the cell[12].

Viral translation: The viral proteins can be divided into 3 main categories: structural, non-structural and accessory[13]. There are 4 structural proteins: the spike (S), nucleocapsid (N), envelope (E) and membrane proteins (M), which together with the viral genome form the virion[12]. There are 16 non-structural proteins (nsps 1-16), which perform key tasks such as viral protein processing and replication of the viral genome[13]. There are also a number of accessory proteins, although they are less well understood[12]. Only a subset of the ORFs in this region of the genome are actually expressed as proteins. On the basis of transcriptomic and proteomic evidence we include p3a, p3b, p6, p7a, p7b, p8, p9b and p9c[14–16].

Two large ORFs: *ORF1a* and *ORF1ab* are translated directly from the viral (RNA) genome into poly-proteins pp1a and pp1ab[13]. Two proteases, 3CLpro (nsp5) and PLpro (nsp3) cleave these polyproteins into the constituent nsps. The structural and accessory proteins are translated from small genomic RNAs (sgRNAs) which are transcribed from the genome by RNA-Dependent RNA Polymerase (RDRP). The RDRP activity is performed by a complex formed of nsp7, nsp8 and nsp12[17]. The sgRNAs are then capped in a mechanism involving nsp10, nsp14 and nsp16, allowing their translation into proteins[18].

Viral replication: The virus reproduces by the formation of new virions in the ERGIC (ER-Golgi Intermediate Compartment)[19]. These particles are assembled from the four structural proteins surrounding the viral genome. After formation, the virions exit the cell through exocytosis.

Innate immune detection of the virus: Two innate virus sensing pathways are thought to be activated by SARS-CoV-2 in epithelial cells[20]. The first relies on cytosolic Pattern Recognition Receptors (PRRs), namely RIG-I and MDA5 which detect viral RNA, leading to activation of the kinase TBK1[21]. TBK1 phosphorylates the transcription factors IRF3 and IRF7, leading to expression of Type I and Type III IFNs. The second pathway involves the TLR3 receptor, which recognizes dsRNA species that form during replication of the viral genome[21]. This pathway also activates TBK1, as well as promoting inflammation by activating NF-κB, through IKK and IkBα[22].

IFN signalling pathway: Type I and Type III IFNs are expressed in lung epithelial cells[3] but Type II IFNs are only produced in some immune cells[23]. However, all types of IFNs are recognized by epithelial cells. There are multiple IFNs of each type as well as multiple receptors, but to simplify the model we

consider only the different types of IFN and a single receptor for each type. Upon activation of the receptor, a signal is passed through JAK-STAT signalling leading to expression of Interferon-Stimulated Genes (ISGs)[4]. In this model we include the ISGs CH25H and LY6E, which inhibit viral entry[6,7]; ZAP and OAS which lead to degradation of viral RNA[4,24]; IFITM which has been shown to inhibit spike-mediated fusion [25]; PKR which can inhibit translation[26]; and iNOS which is a cellular producer of Nitrous Oxide[27].

Cytokine secretion: Alongside the IFNs, the model includes a number of cytokines expressed both directly by epithelial cells and indirectly via immune cells. NF-κB is responsible for the expression of a wide range of cytokines and is a signalling hub on which multiple lines of signalling converge[22], including the Unfolded Protein Response (UPR)[28] and IFN signalling[29]. The cytokines represented in the model were chosen to represent those commonly observed in COVID-19 patients and correlated with worse outcomes[30,31].

Cell death: The model includes the pathways regulating apoptosis, pyroptosis and necroptosis. The model contains Caspases-1, 3, 8 and 10, with the activity of Caspase-3 controlling the level of apoptosis[32]. Pyroptosis is controlled by the activity of gasdermins D and E, which are regulated by the inflammasome and PANoptosome complexes respectively[33]. Necroptosis is regulated by MLKL which is also controlled by the PANoptosome[32].

Renin-angiotensin system: ACE2 is the key receptor used by SARS-CoV-2 to enter the cell, but it is also an enzyme which is important for regulation of angiotensin, a hormone that regulates blood pressure[34]. In healthy cells, ACE2 converts AngII into Ang1-7, helping to shift the balance in favour of Ang1-7[35]. Interaction of ACE2 with spike proteins is thought to prevent its catalytic activity, leading to an increase in AngII levels. AngII is detected by the AT1R, while Ang1-7 is detected by MasR. An imbalance between the activities of these two receptors leads to ER stress and activation of MAPK and ROCK kinases[36].

Translation of host proteins: Translation of host proteins can proceed via both cap-dependent and cap-independent pathways[37], while viral proteins rely on cap-dependent pathways[38]. Both pathways rely on ribosomal subunits, however they have different requirements in terms of eIFs. The viral protein nsp1 binds to the 40S ribosomal subunit and causes preferential translation of viral transcripts[39].

Endoplasmic Reticulum (ER) stress: ER stress is caused both by imbalance of the Ang1-7:AngII ratio and also by the S and p3a proteins[35,40,41]. ER stress leads to activation of the Unfolded Protein Response (UPR)[28]. The UPR has 3 pathways: ATF6 which acts to decrease protein translation; and IRE1 and XBP1 which cause inflammation.

Separation of initial infection and IFN response in the model

In order to distinguish between initial viral entry and viral entry after the response induced by the innate immune system, we use two separate nodes. The *Viral Entry1* node represents the initial entry of the virus into the cell and is uninhibited by the IFN response, while the *Viral Entry* node shows the effects of this IFN response. If the model stabilises in a state where *Viral Entry1* and *Viral Replication* are active but *Viral Entry* is fully inhibited, we interpret this as showing that a small population of cells was initially infected but that the subsequent IFN response prevented a spreading of infection throughout the wider population of cells. Similarly, we include a *Viral Genome Degradation* node to represent the effects of ISGs that restrict viral RNA. This node inhibits the *Viral Replication* node, representing the requirement of the virion for intact viral RNA, but not the *Viral Genome* node, which represents the initial proliferation of viral RNA.

Since our model runs from all possible initial states, IFN-I and NF-κB could self-activate each other[22,29]. To prevent this undesired behaviour, we model the induction of IFNs through the innate immune sensing machinery alone and discount positive feedback occurring through NF-κB, as the former interaction is more relevant in the early stage of the severe disease. Conversely, in the late stage, IFN activity is high due to many mechanisms, some of which remain unknown, driving a persistent and maladaptive inflammatory response. This is represented by the high level of the *Sensitivity of IFN Response* node. Taken together, these choices allow the model to represent ongoing feedback alongside the initial stimulus, giving greater insight into the underlying process.

Validating the model against publicly available data

In order to establish whether the model faithfully reproduces experimental data, we tested it against data from scientific literature. We collected 81 experiments derived from 21 published and pre-print studies using SARS-CoV-2, specific viral proteins, SARS-CoV-1 and relevant host factors. The full list of experimental observations we used to test the model is summarised in Supplementary Table 4. We reproduced the conditions described in each experiment as constraints on the model by fixing the nodes representing the experimental variables to a specific value. For example, the effect of an inhibitor was modelled by setting the relevant node to the constant value of 0. We then confirmed whether the resulting steady state of the model matched the experimental results. For example, Hoffmann, M et al.[42] used the protease inhibitor E-64d to inhibit cathepsinL in VeroE6 cells expressing exogenous TMPRSS2. To reproduce this, we set *TMPRSS2* to 1, and *cathepsinL* to 0. Hoffmann, M et al.[42] showed that this treatment reduced viral entry; indeed our model predicts that E-64d reduces the level of *Viral Entry* from 2 to 1, matching the experiment.

Our model reproduced 74 experimental observations out of the 81 tested. Zhang, X. et al.[41] shows that IFN-I is induced by expression of p3a, which the model cannot recapitulate, most likely as in our model NF-κB does not induce IFNs. Similarly, Thorne L. G. et al.[43] found that inhibition of NF-κB with the IKK inhibitor TPCA-1 reduced IFN expression, a finding that is also not recapitulated by the model. Furthermore, Zhang, X. et al.[41] also found that expression of p3a leads to production of TNF-α in A549. The model assumes that TNF-α is produced only by macrophages[44], meaning it will not be produced in an epithelial cell line. This is also the reason that the model does not reproduce the findings of Hsu, A. C.-Y. et al.[40], that TNF-α is induced by expression of S protein in a minimally immortalized epithelial cell line, BCi-NS1.1. The model is also unable to reproduce the findings of Hsu, A. C.-Y. et al.[40] that S-induced induction of various cytokines is suppressed by the ERK inhibitor, Ulixertinib. Banerjee, A. K. et al.[39] show that nsp8 and nsp9 inhibit IFN release by binding to the 7SL RNA component of the Signal Recognition Particle (SRP), which is represented in the model. They then show that nsp8 and nsp9 inhibit the ISG response of cells treated with IFN. Inhibition of the SRP by nsp8 and nsp9 is represented in the model but this mechanism is not sufficient to explain the experiment described so we cannot fully reproduce the experiment. Thorne L. G. et al.[43] found that IL-6 production is not affected by inhibition of MDA5 or Jak1/2, a disagreement with the model we cannot explain at this point. Furthermore, Thorne L. G. et al.[43] found that the IKK inhibitor TPCA-1 and the Jak1/2 inhibitor Ruxolitinib prevented virus-induced cell death in Calu3 cells, a finding not reproduced in the model. Finally, Thorne L. G. et al.[43] also found that Ruxolitinib had no effect on viral replication in their Calu3 cultures, while the model in fact predicts that inhibition of IFN signalling, such as through Jak1/2 inhibition, will increase viral replication. This is likely due to the distinction between viral challenge in cell culture, where all cells are all equally permissive and presented with the virus simultaneously, and the situation in a human lung, where the infection starts off localised in a permissive cell subset[45], allowing IFN signalling to be induced prior to infection in neighbouring regions. Certainly, there is evidence that a robust, timely IFN response is associated with mild disease[46,47] in humans and that loss-of-function mutations in the IFN response pathway are associated with more severe disease[48]. Therefore, while the model does not capture the behaviour of Ruxolitinib in cell culture, this may reflect the differences between cell culture and human disease.

Having tested the model against experimental data, we then checked the behaviour of commonly used drugs using the model. Remdesivir is a nucleoside analogue that blocks RDRP and has shown promise against SARS-CoV-2 in cell lines[49] and as a treatment for COVID-19[50]. We tested the effects of Remdesivir by setting the RDRP complex nsp12-nsp7-nsp8 to 0 and checked the steady state of the model in each of the clinically relevant conditions (Figure 3a). We specifically checked each of the eight biological processes nodes and compared their value to the untreated state. In this case, the model recapitulates the activity of Remdesivir, showing a total loss of Viral Replication. We repeated this analysis for Lopinavir, Tocilizumab, Dexamethasone, Roferon-A and Ruxolitinib.

Having established that the model captures the behaviour of commonly used drugs, we were interested to see if the model can predict the impact of genetic risk factors associated with increased risk of severe COVID-19. Zhang, Q. et al.[51] found that mutations in the IFN-I pathway, including IFNAR, lead to an increased risk of severe disease. Pairo-Castineira, E. et al.[48] also identified mutations in IFNAR, as well as OAS. We tested the impact of these genetic background in the same way that we tested the drugs effect (Supplementary Figure 12). Notably, the model predicts that loss of function mutations in IFNAR or OAS would increase viral replication in mild COVID-19 (Supplementary Figure 12a, b), suggesting that people with these mutations are at a higher risk of developing severe disease. We also tested the findings of Boudewijns, R. et al.[52], who used a hamster model of severe SARS-CoV-2 infection to show that in the late stages of disease STAT2-/- hamsters had increased viral load but reduced inflammation, which was in agreement with our model (Supplementary Figure 12c).

In silico **screens of new and improved COVID-19 drug combination treatments**

We used the model to simulate the effect of applying drugs individually and in all combinations of two drugs, in each of the four states of the model. The network includes many nodes which are the targets of existing selective therapies, such as Miltefosine, an Akt inhibitor[53]. For each node, we searched the literature for drugs targeting that node. This resulted in a table of 74 potential drugs (Supplementary Table 5). Of these 74, we further filtered to focus on those that had passed Phase II clinical trials (Table 1) For each treatment tested and condition we recorded the steady state value of the 8 symptom nodes. In the case that a fixed-point steady state could not be found, due to the steady state being a loop or there being multiple steady states and/or loops, we used the mid-point of the upper and lower bounds BMA could place on the behaviour of that node as representative of the predominant behaviour in these cases. This was taken across all steady states and calculated using the algorithm described in Cook et al[54]. The individual results for the eight phenotypes in the mild, early and late severe cases are reported in Supplementary Data 1. Here, the column "Number_of_perturbations" indicates whether a single or pairwise combination was applied; the column "Disease_State" indicates mild, early severe or late severe disease state; the column "Perturbation_A" indicates the first perturbation applied; the column "Level_A" indicates the level the first node is set to, or not applicable (NA) as the effect is defined in Supplementary Table 5; the column "Perturbation_B" indicates the second perturbation applied or not applicable (NA); the column “Level_B” indicates the level the second node is set to or not applicable (NA); the column "Lower bound" shows the lower bound for the phenotype measured determined by the algorithm; the column "Upper bound" shows the upper bound for the phenotype measured determined by the algorithm and the column "Midpoint" shows the midpoint between the upper and lower bound.

We focussed our analysis on two specific parameters and conditions: viral replication in the early stage of the severe case and inflammation in the late stage of the severe case. First, we considered the effect of each of the 38 drugs individually on viral replication in the early severe case (Figure 4a). We plotted the results as a heatmap, showing the level of *Viral Replication* after each treatment. This showed that many drugs in consideration as treatments for COVID-19, such as Baracitinib[55], were effective in our model. However, in order to effectively treat patients at this stage of disease, it may be necessary to

administer these drugs as post-exposure prophylaxis. We then turned our attention to the combination therapies (Figure 4b). Here we again plotted the results as a heatmap, with each square showing the value of *Viral Replication* upon treatment with the drugs shown on the x and y axis. We looked specifically for combinations that improved upon the single therapies, for example the combination of Dexamethasone and Ulixertinib. We repeated this analysis for Inflammation in the severe, late infection condition (Figures 4c, d).

We applied the above analysis to all eight phenotypic readout nodes in the mild, severe early and severe late conditions (Supplementary Figures 2-4). We also extended this analysis to the full list of 74 targeted drugs (Supplementary Figures 8, 9). Furthermore, we tested the impact of inhibiting all nodes individually and in combination, even where no drugs targeting these proteins currently exists (Supplementary Figures 10, 11).

In order to examine the effect of each drug combination on all the biological processes modelled, we plotted these values for each combination as a radar chart (Figure 5, Supplementary Figure 1). In these plots, the biological processes nodes are arranged in a circle and the value reached with a given treatment is plotted as point, which are then joined together between all biological processes. A representative monotherapy is plotted in grey with the combination therapy plotted in colour (orange for treatments identified for severe, early cases and red for treatments identified for severe, late cases). We do not model the dose relations of drug combinations and so cannot distinguish between synergistic and additive effects. However, we particularly focus on cases in which two drugs achieve an effect neither could as a monotherapy. For example, Ulixertinib and Miltefosine were predicted to have no effect on viral replication in the early stage of severe disease when applied as monotherapies but were predicted to have strong effect together (Fig 4a, b).

In silico simulation of Caco-2 cell drug treatments

We validated the model's predictions that Apilimod and Camostat; and Dexamethasone and Miltefosine act together to reduce viral replication. We test these predictions using Caco-2 cell cultures as they do not mount a significant IFN response to SARS-CoV-2 infection[56], providing a model of the severe, early COVID-19 condition. We modelled Caco-2 cells by setting the *Immune Cells* node to 0 as this is a clonal colorectal epithelial cell culture, and the *Sensitivity Of IFN Response* node to 0 to reflect the lack of significant IFN response. These simulations predict that combinations of Apilimod and Camostat, and Dexamethasone and Miltefosine, have a greater effect in combination than alone in Caco-2 cells (Supplementary Table 7).

Differential characteristics of different states of COVID-19

Individuals with COVID-19 display a wide range of symptoms and disease outcomes, with severity ranging from asymptomatic to fatal[57]. Therefore, there is a need for clinical biomarkers to aid in triage of COVID-19 patients. We investigated the steady states of our model representing the different states of disease to look for differences that could potentially distinguish between mild and severe cases. We applied hierarchical clustering to the level of all nodes in each state (Supplementary Figure 5a). The resulting heatmap reveals clusters of nodes that differ between states of the disease (Supplementary Figure 5b).

Hierarchical clustering identifies IFN-I, IFN-II, IFN-III, TNF-α, IL-2, IL-1B, IL-6, IL-8, IL-10, IL-18, CCL2, CXCL9, CXCL10 and CXCL11. All of these factors are raised in the late versus early severe stage of disease, in line with observed increases in IFN-I, IFN-II, IFN-III, IL-2, IL-10 and IL-18 reported in longitudinal studies[8,30]. The model also predicts that these factors are higher in the mild state compared to the early severe state of disease, reflecting the effective immune response in these

patients. Many of these factors, especially TNF-α, IL-6, IL-8, IL-10 and CXCL10 have been identified as prognostic markers for severity of COVID-19 in hospitalised patients[30,31,47,58,59].

References

1. Schaub, M. A., Henzinger, T. A. & Fisher, J. Qualitative networks: a symbolic approach to analyze biological signaling networks. *BMC Syst. Biol.* **1**, 4 (2007).
2. Benque, D. *et al.* BMA: Visual Tool for Modeling and Analyzing Biological Networks. *Comput. Aided Verif.* 686–692 (2012).
3. Park, A. & Iwasaki, A. Type I and Type III Interferons – Induction, Signaling, Evasion, and Application to Combat COVID-19. *Cell Host Microbe* **27**, 870–878 (2020).
4. Schoggins, J. W. & Rice, C. M. Interferon-stimulated genes and their antiviral effector functions. *Curr. Opin. Virol.* **1**, 519–525 (2011).
5. Nchioua, R. *et al.* The Zinc Finger Antiviral Protein restricts SARS-CoV-2. *bioRxiv* **130**, 2620–40.
6. Zang, R. *et al.* Cholesterol 25-hydroxylase suppresses SARS-CoV-2 replication by blocking membrane fusion. *Proc. Natl. Acad. Sci.* **117**, 32105–32113 (2020).
7. Pfaender, S. *et al.* LY6E impairs coronavirus fusion and confers immune control of viral disease. *Nat. Microbiol.* **5**, 1330–1339 (2020).
8. Lucas, C. *et al.* Longitudinal analyses reveal immunological misfiring in severe COVID-19. *Nature* **584**, 463–469 (2020).
9. Shang, J. *et al.* Cell entry mechanisms of SARS-CoV-2. *Proc. Natl. Acad. Sci. U. S. A.* **117**, 202003138 (2020).
10. Hoffmann, M. *et al.* SARS-CoV-2 Cell Entry Depends on ACE2 and TMPRSS2 and Is Blocked by a Clinically Proven Protease Inhibitor. *Cell* **181**, 271-280.e8 (2020).
11. Daniloski, Z. *et al.* Identification of Required Host Factors for SARS-CoV-2 Infection in Human Cells. *Cell* **184**, 92-105.e16 (2021).
12. Wang, Y., Grunewald, M. & Perlman, S. Coronaviruses: An Updated Overview of Their Replication and Pathogenesis. in *Methods in molecular biology (Clifton, N.J.)* vol. 2203 1–29 (2020).
13. Chen, Y., Liu, Q. & Guo, D. Emerging coronaviruses: Genome structure, replication, and pathogenesis. *J. Med. Virol.* **92**, 418–423 (2020).
14. Davidson, A. D. *et al.* Characterisation of the transcriptome and proteome of SARS-CoV-2 reveals a cell passage induced in-frame deletion of the furin-like cleavage site from the spike glycoprotein. *Genome Med.* **12**, 68 (2020).
15. Bouhaddou, M. *et al.* The Global Phosphorylation Landscape of SARS-CoV-2 Infection. *Cell* **182**, 685-712.e19 (2020).
16. Kim, D. *et al.* The Architecture of SARS-CoV-2 Transcriptome. *Cell* **181**, 914-921.e10 (2020).
17. Chen, J. *et al.* Structural Basis for Helicase-Polymerase Coupling in the SARS-CoV-2 Replication-Transcription Complex. *Cell* **182**, 1560-1573.e13 (2020).
18. Viswanathan, T. *et al.* Structural basis of RNA cap modification by SARS-CoV-2. *Nat. Commun.* **11**, 3718 (2020).
19. Fehr, A. R. & Perlman, S. Coronaviruses: an overview of their replication and pathogenesis. *Methods Mol. Biol.* **1282**, 1–23 (2015).
20. Vabret, N. *et al.* Immunology of COVID-19: Current State of the Science. *Immunity* **52**, 910–941 (2020).
21. Mogensen, T. H. Pathogen Recognition and Inflammatory Signaling in Innate Immune Defenses. *Clin. Microbiol. Rev.* **22**, 240–273 (2009).
22. Liu, T., Zhang, L., Joo, D. & Sun, S.-C. NF-κB signaling in inflammation. *Signal Transduct. Target. Ther.* **2**, 17023 (2017).
23. Ivashkiv, L. B. IFNγ: signalling, epigenetics and roles in immunity, metabolism, disease and

cancer immunotherapy. *Nat. Rev. Immunol.* **18**, 545–558 (2018).
24. Nchioua, R. *et al.* The Zinc Finger Antiviral Protein restricts SARS-CoV-2. *MBio* **130**, 2620–40 (2020).
25. Buchrieser, J. *et al.* Syncytia formation by SARS-CoV-2-infected cells. *EMBO J.* **39**, 1–12 (2020).
26. Gal-Ben-Ari, S., Barrera, I., Ehrlich, M. & Rosenblum, K. PKR: A Kinase to Remember. *Front. Mol. Neurosci.* **11**, 480 (2019).
27. Karki, R. *et al.* Synergism of TNF-α and IFN-γ Triggers Inflammatory Cell Death, Tissue Damage, and Mortality in SARS-CoV-2 Infection and Cytokine Shock Syndromes. *Cell* **184**, 149-168.e17 (2021).
28. Pathinayake, P. S. *et al.* Understanding the Unfolded Protein Response in the Pathogenesis of Asthma. *Front. Immunol.* **9**, 621–13 (2018).
29. Makris, S., Paulsen, M. & Johansson, C. Type I Interferons as Regulators of Lung Inflammation. *Front. Immunol.* **8**, 1–10 (2017).
30. Liu, J. *et al.* Longitudinal characteristics of lymphocyte responses and cytokine profiles in the peripheral blood of SARS-CoV-2 infected patients. *EBioMedicine* **55**, 102763 (2020).
31. Diao, B. *et al.* Reduction and Functional Exhaustion of T Cells in Patients With Coronavirus Disease 2019 (COVID-19). *Front. Immunol.* **11**, 1–7 (2020).
32. Lee, S., Channappanavar, R. & Kanneganti, T.-D. Coronaviruses: Innate Immunity, Inflammasome Activation, Inflammatory Cell Death, and Cytokines. *Trends Immunol.* **41**, 1083–1099 (2020).
33. van den Berg, D. F. & te Velde, A. A. Severe COVID-19: NLRP3 Inflammasome Dysregulated. *Front. Immunol.* **11**, 1708–6 (2020).
34. Sriram, K., Loomba, R. & Insel, P. A. Targeting the renin−angiotensin signaling pathway in COVID-19: Unanswered questions, opportunities, and challenges. *Proc. Natl. Acad. Sci.* **117**, 29274–29282 (2020).
35. Forrester, S. J. *et al.* Angiotensin II Signal Transduction: An Update on Mechanisms of Physiology and Pathophysiology. *Physiol. Rev.* **98**, 1627–1738 (2018).
36. Meng, Y. *et al.* The angiotensin-converting enzyme 2/angiotensin (1-7)/Mas axis protects against lung fibroblast migration and lung fibrosis by inhibiting the NOX4-derived ROS-mediated RhoA/Rho kinase pathway. *Antioxid. Redox Signal.* **22**, 241–258 (2015).
37. Sonenberg, N. & Hinnebusch, A. G. Regulation of Translation Initiation in Eukaryotes: Mechanisms and Biological Targets. *Cell* **136**, 731–745 (2009).
38. Nakagawa, K., Lokugamage, K. G. & Makino, S. Viral and Cellular mRNA Translation in Coronavirus-Infected Cells. *Adv. Virus Res.* **96**, 165–192 (2016).
39. Banerjee, A. K. *et al.* SARS-CoV-2 Disrupts Splicing, Translation, and Protein Trafficking to Suppress Host Defenses. *Cell* **183**, 1325-1339.e21 (2020).
40. Hsu, A. C.-Y. *et al.* SARS-CoV-2 Spike protein promotes hyper-inflammatory response that can be ameliorated by Spike-antagonistic peptide and FDA-approved ER stress and MAP kinase inhibitors in vitro. *bioRxiv* 2020.09.30.317818 (2020) doi:10.1101/2020.09.30.317818.
41. Zhang, X. *et al.* Sequential ER stress and inflammatory responses are induced by SARS-CoV-2 ORF3 through ERphagy. *bioRxiv* (2020) doi:10.1101/2020.11.17.387902.
42. Hoffmann, M. *et al.* SARS-CoV-2 Cell Entry Depends on ACE2 and TMPRSS2 and Is Blocked by a Clinically Proven Protease Inhibitor. *Cell* **181**, 271-280.e8 (2020).
43. Thorne, L. G. *et al.* SARS-CoV-2 sensing by RIG-I and MDA5 links epithelial infection to macrophage inflammation. *bioRxiv* 2020.12.23.424169 (2020) doi:10.1101/2020.12.23.424169.
44. Falvo, J. V., Tsytsykova, A. V. & Goldfeld, A. E. Transcriptional Control of the TNF Gene. in *TNF Pathophysiology* vol. 11 27–60 (KARGER, 2010).
45. Lee, I. T. *et al.* ACE2 localizes to the respiratory cilia and is not increased by ACE inhibitors or ARBs. *Nat. Commun.* **11**, 5453 (2020).
46. Hadjadj, J. *et al.* Impaired type I interferon activity and inflammatory responses in severe

COVID-19 patients. *Science (80-.).* **369**, 718–724 (2020).
47. Arunachalam, P. S. *et al.* Systems biological assessment of immunity to mild versus severe COVID-19 infection in humans. *Science (80-.).* **369**, 1210–1220 (2020).
48. Pairo-Castineira, E. *et al.* Genetic mechanisms of critical illness in COVID-19. *Nature* **591**, 92–98 (2021).
49. Wang, M. *et al.* Remdesivir and chloroquine effectively inhibit the recently emerged novel coronavirus (2019-nCoV) in vitro. *Cell Res.* **30**, 269–271 (2020).
50. Beigel, J. H. *et al.* Remdesivir for the Treatment of Covid-19 — Final Report. *N. Engl. J. Med.* **383**, 1813–1826 (2020).
51. Zhang, Q. *et al.* Inborn errors of type I IFN immunity in patients with life-threatening COVID-19. *Science (80-.).* **21**, eabd4570 (2020).
52. Boudewijns, R. *et al.* STAT2 signaling restricts viral dissemination but drives severe pneumonia in SARS-CoV-2 infected hamsters. *Nat. Commun.* **11**, 5838 (2020).
53. Nitulescu, G. M. *et al.* Akt inhibitors in cancer treatment: The long journey from drug discovery to clinical use (Review). *Int. J. Oncol.* **48**, 869–885 (2016).
54. Cook, B., Fisher, J., Krepska, E. & Piterman, N. Proving Stabilization of Biological Systems. in *VMCAI* vol. 11 134–149 (Springer, 2011).
55. Richardson, P. *et al.* Baricitinib as potential treatment for 2019-nCoV acute respiratory disease. *Lancet* **395**, e30–e31 (2020).
56. Stanifer, M. L. *et al.* Critical Role of Type III Interferon in Controlling SARS-CoV-2 Infection in Human Intestinal Epithelial Cells. *CellReports* **32**, 107863.
57. Guan, W. *et al.* Clinical Characteristics of Coronavirus Disease 2019 in China. *N. Engl. J. Med.* **382**, 1708–1720 (2020).
58. Chen, Y. *et al.* Blood molecular markers associated with COVID-19 immunopathology and multi-organ damage. *EMBO J.* **39**, 1–23 (2020).
59. Del Valle, D. M. *et al.* An inflammatory cytokine signature predicts COVID-19 severity and survival. *Nat. Med.* **26**, 1636–1643 (2020).

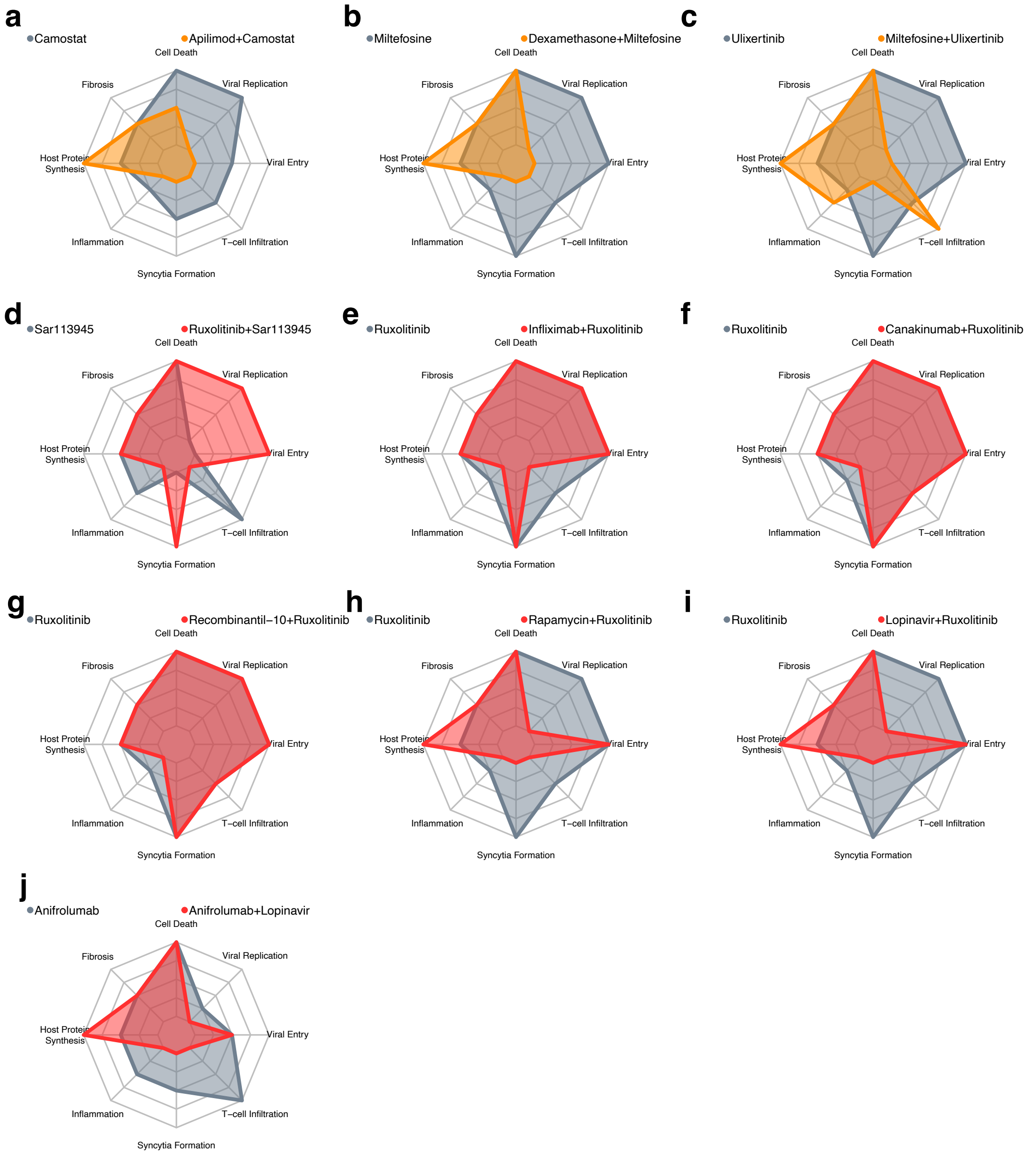

Supplementary Figure 1. Effect of drug combinations identified by in silico screening. (**a-c**) The effect of monotherapy vs drug combinations identified to limit viral replication under the early stage, severe Covid-19 condition. Monotherapy (grey), combination (orange), with the strength of the symptom denoted by radial distance. (**d-j**) The effect of monotherapy vs drug combinations identified to reduce inflammation under the late stage, severe Covid-19 condition. Monotherapy (grey), combination (Red). All nodes normalized to be between 0 and 100%.

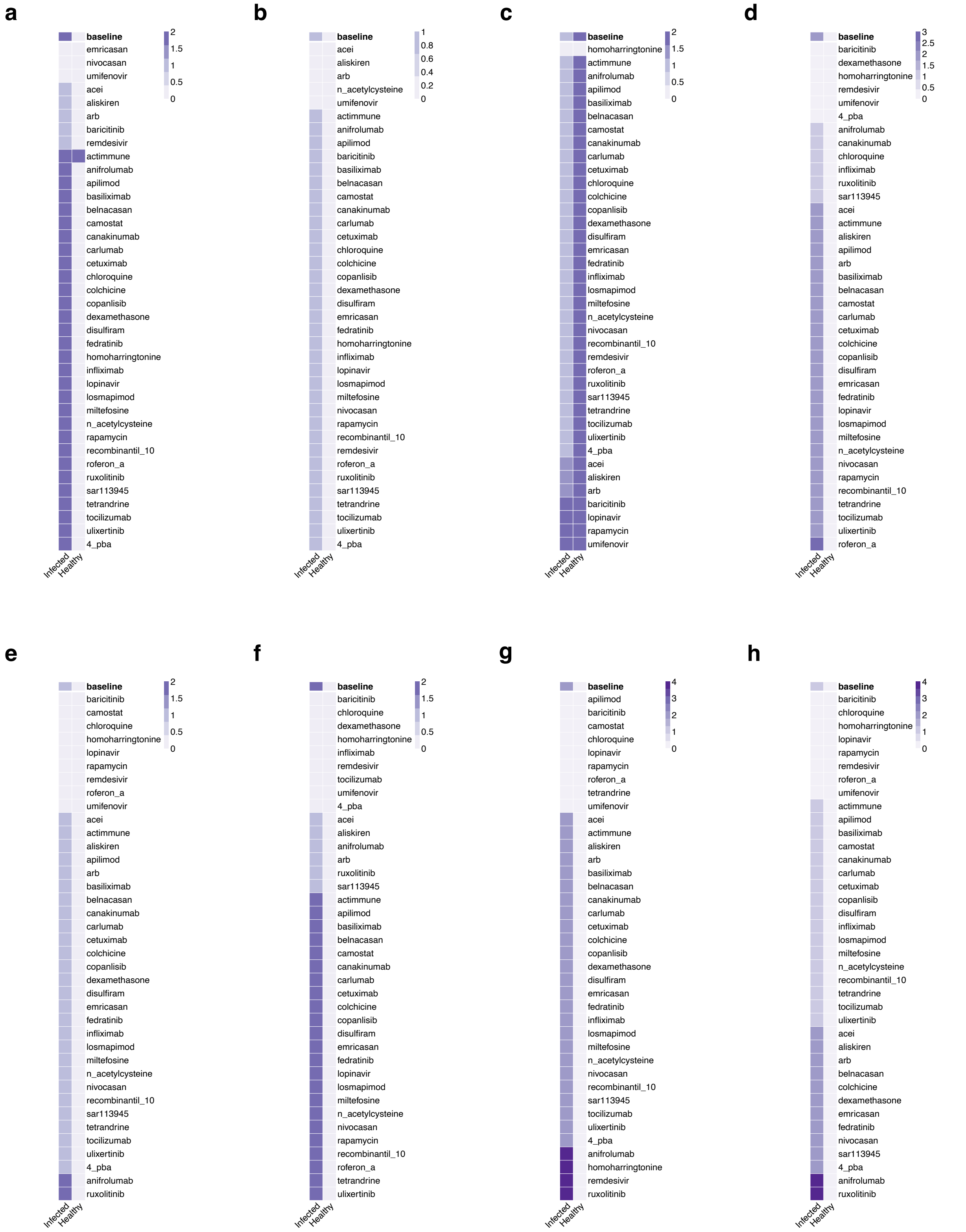

Supplementary Figure 2 a-h. Effects of single therapies (38 drugs that have passed phase II trials) in mild COVID-19. Columns represent infected and healthy conditions, rows represent drug tested including only drugs with known safety profiles, having completed phase II clinical trials (Supplementary Table 5). Showing the effect on: (a) Cell Death. (b) Fibrosis. (c) Host Protein Synthesis. (d) Inflammation. (e) Syncytia Formation. (f) T-cell Infiltration. (g) Viral Entry. (h) Viral Replication.

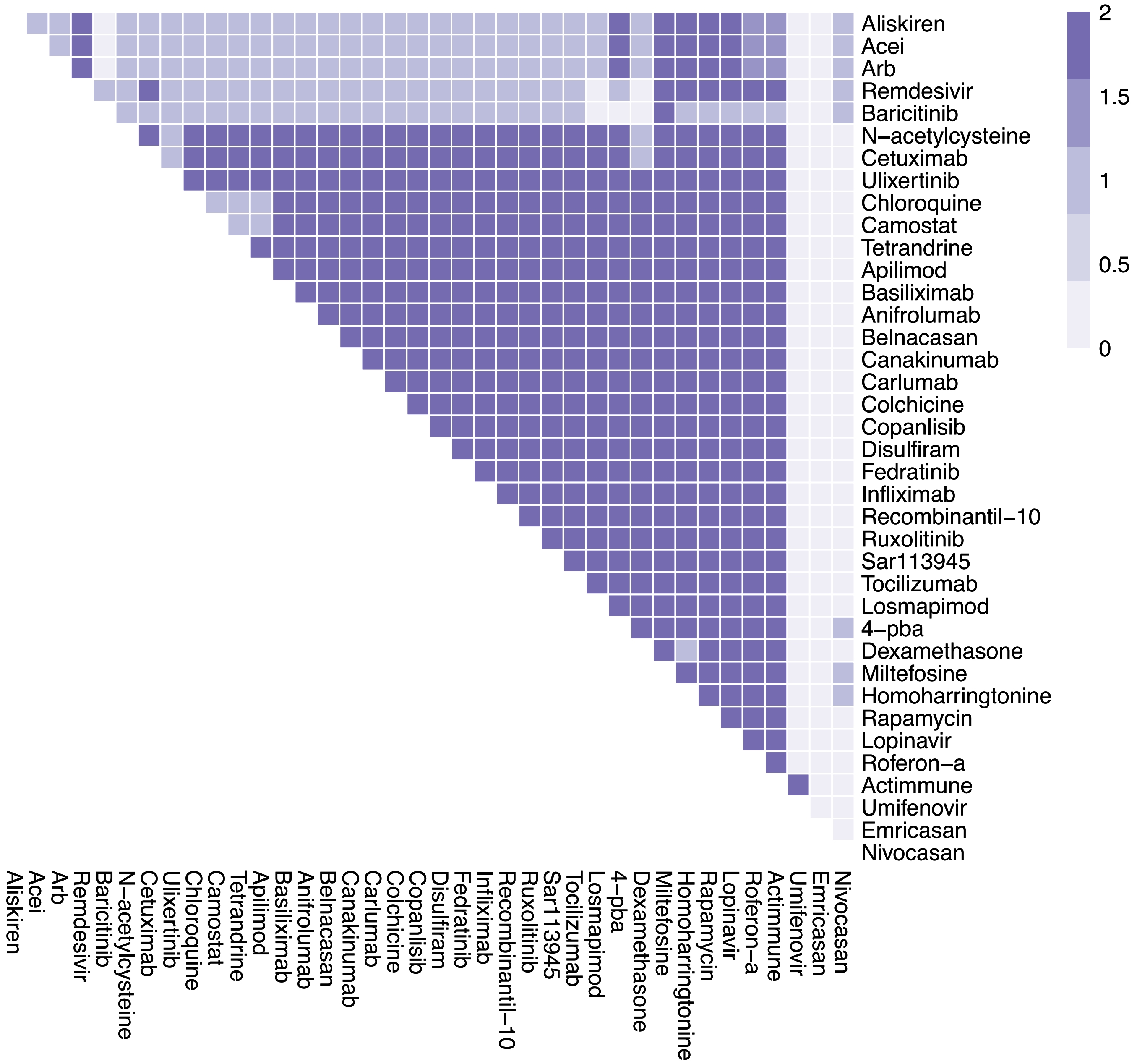

Supplementary Figure 2i. Effects of combination therapy (38 drugs that have passed phase II trials) on Cell Death in mild COVID-19. The colour of the square corresponds to the level of the *CellDeath* node at steady state under treatment by the drugs indicated on the x- and y-axis. The plot shows drugs with known safety profiles, having completed phase II clinical trials (Supplementary Table 5).

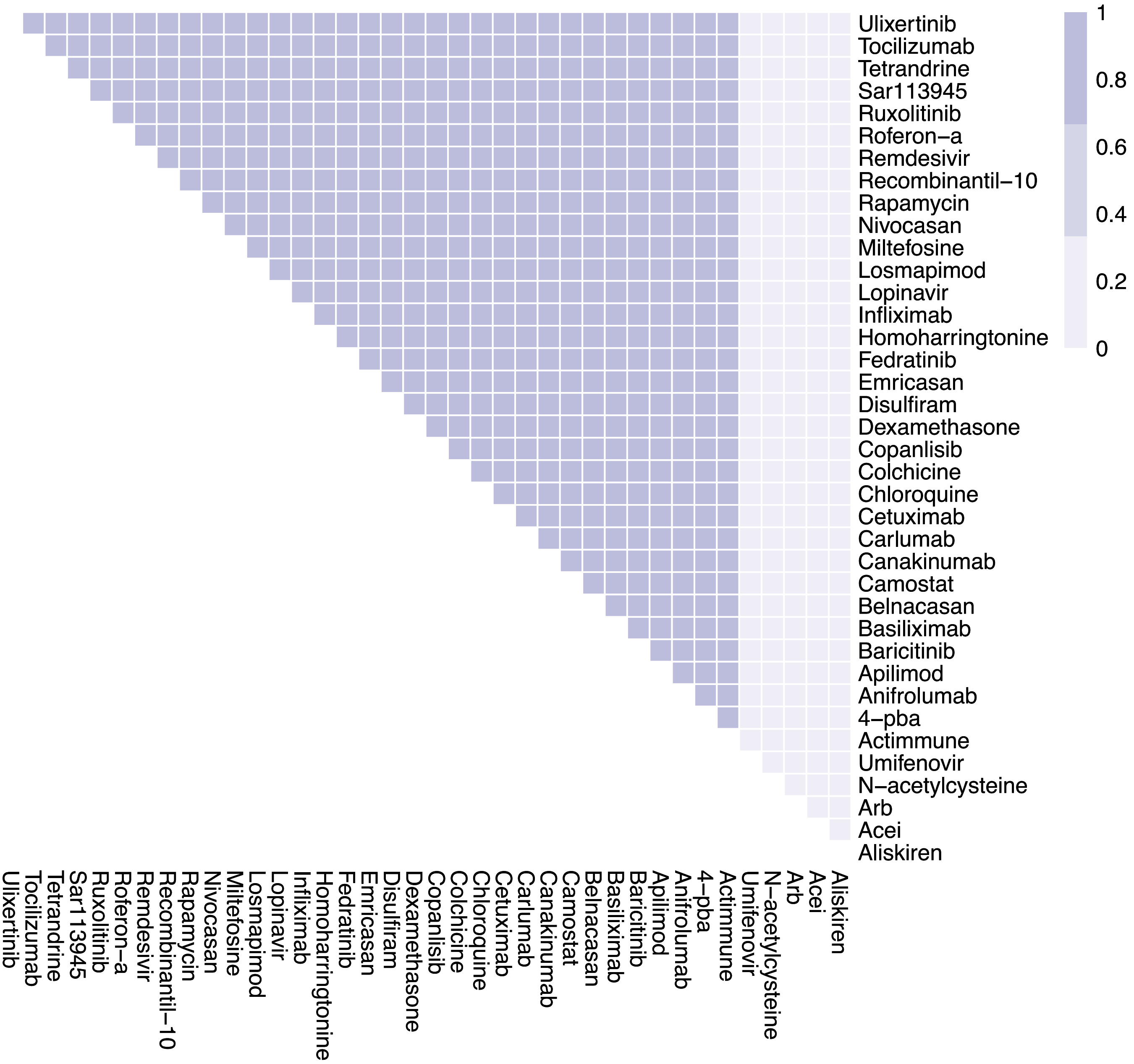

Supplementary Figure 2j. Effects of combination therapy (38 drugs that have passed phase II trials) on Fibrosis in mild COVID-19. The colour of the square corresponds to the level of the *Fibrosis* node at steady state under treatment by the drugs indicated on the x- and y-axis. The plot shows drugs with known safety profiles, having completed phase II clinical trials (Supplementary Table 5).

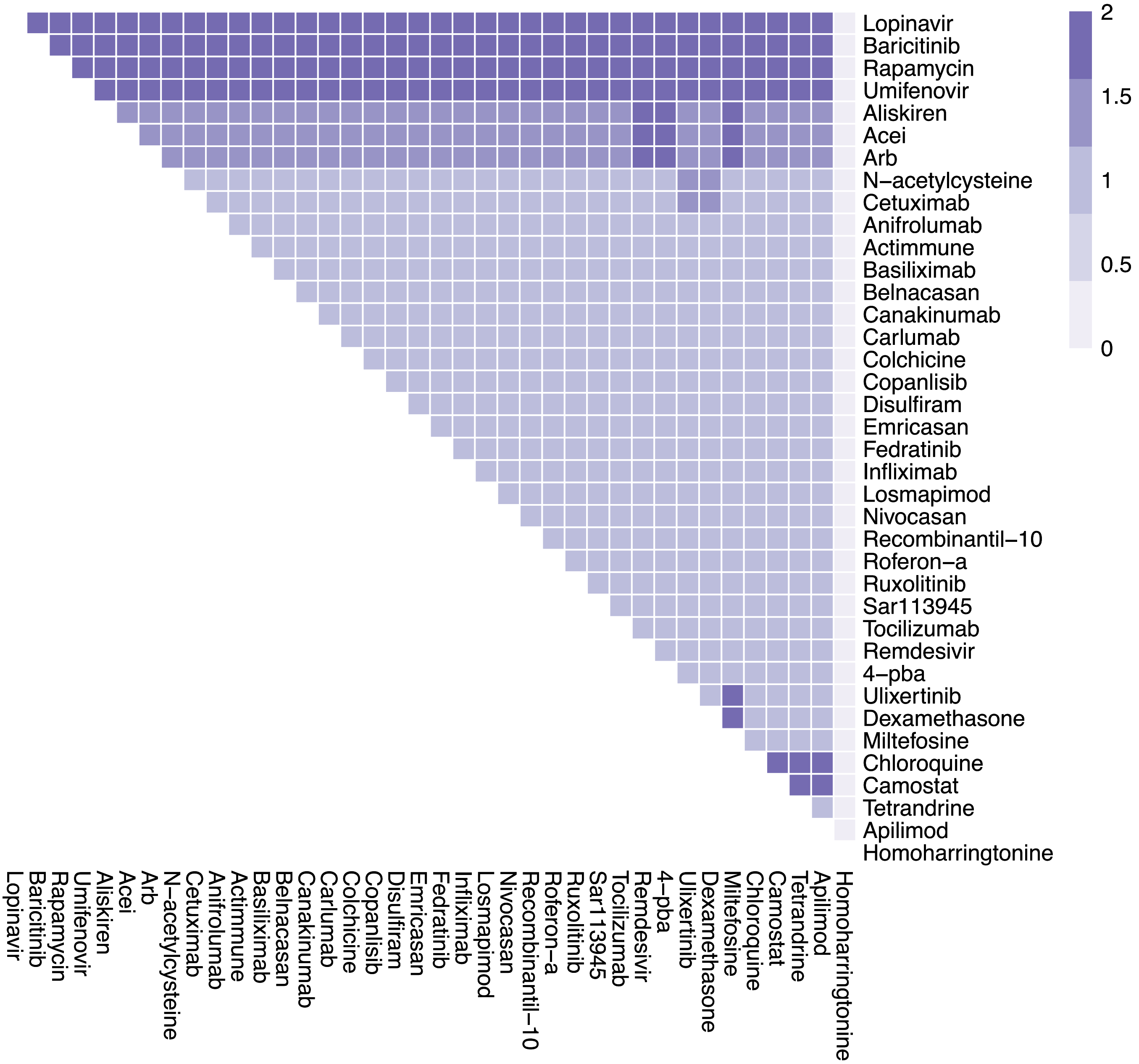

Supplementary Figure 2k. Effects of combination therapy (38 drugs that have passed phase II trials) on Host Protein Synthesis in mild COVID-19. The colour of the square corresponds to the level of the *HostProteinSynthesis* node at steady state under treatment by the drugs indicated on the x- and y-axis. The plot shows drugs with known safety profiles, having completed phase II clinical trials (Supplementary Table 5).

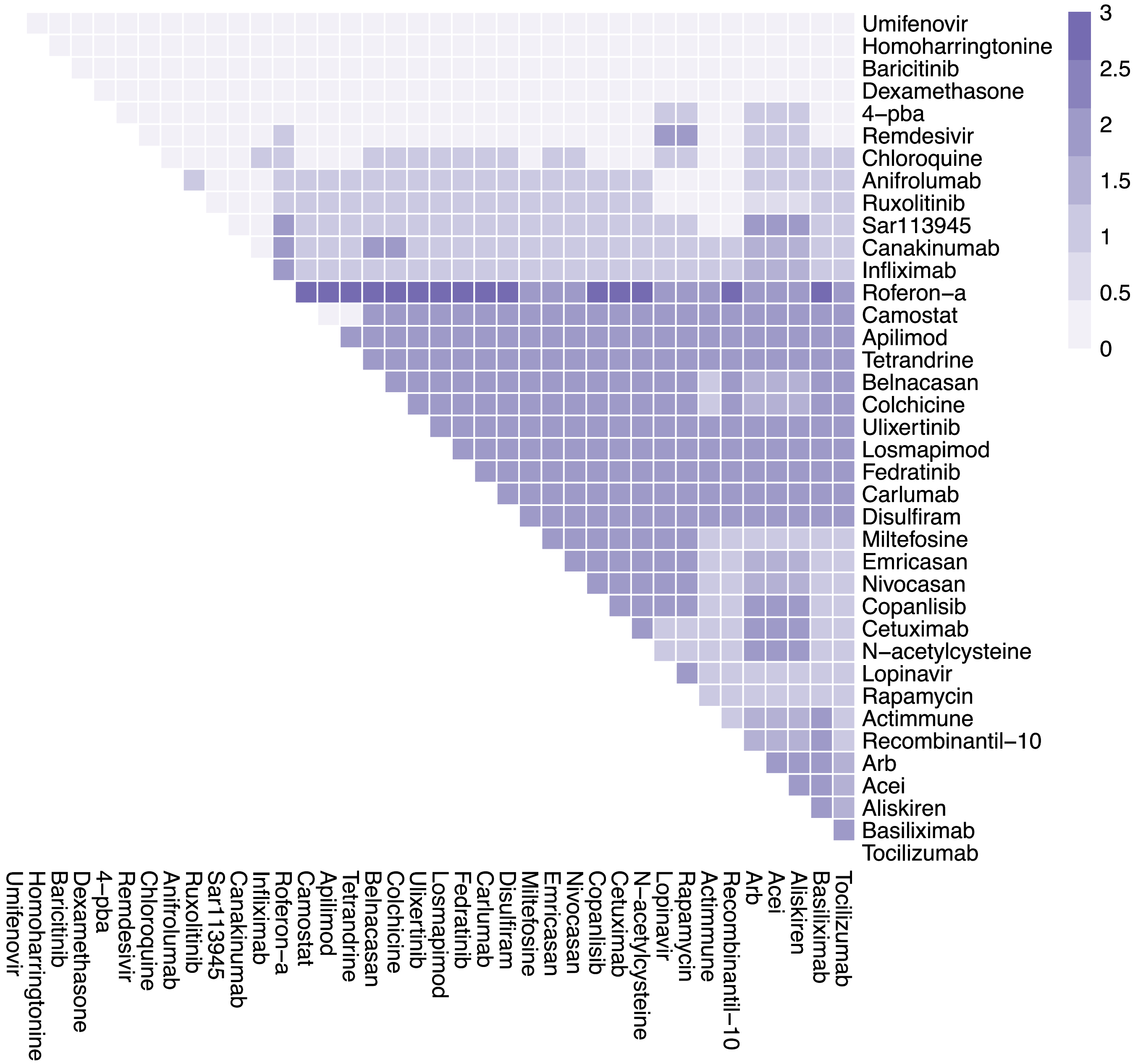

Supplementary Figure 2I. Effects of combination therapy (38 drugs that have passed phase II trials) on Inflammation in mild COVID-19. The colour of the square corresponds to the level of the *Inflammation* node at steady state under treatment by the drugs indicated on the x- and y-axis. The plot shows drugs with known safety profiles, having completed phase II clinical trials (Supplementary Table 5).

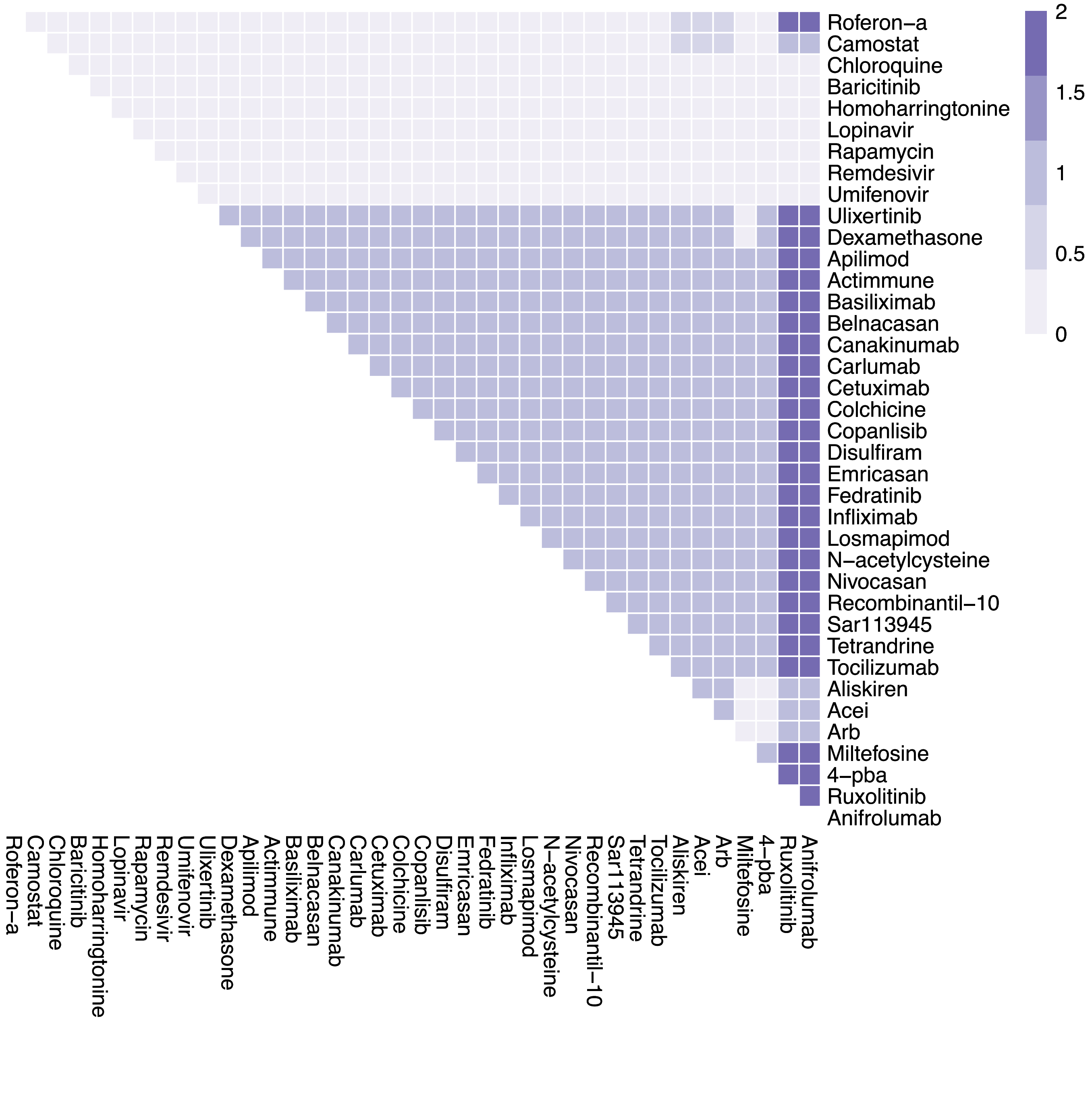

Supplementary Figure 2m. Effects of combination therapy (38 drugs that have passed phase II trials) on Syncytia Formation in mild COVID-19. The colour of the square corresponds to the level of the *SyncytiaFormation* node at steady state under treatment by the drugs indicated on the x- and y-axis. The plot shows drugs with known safety profiles, having completed phase II clinical trials (Supplementary Table 5).

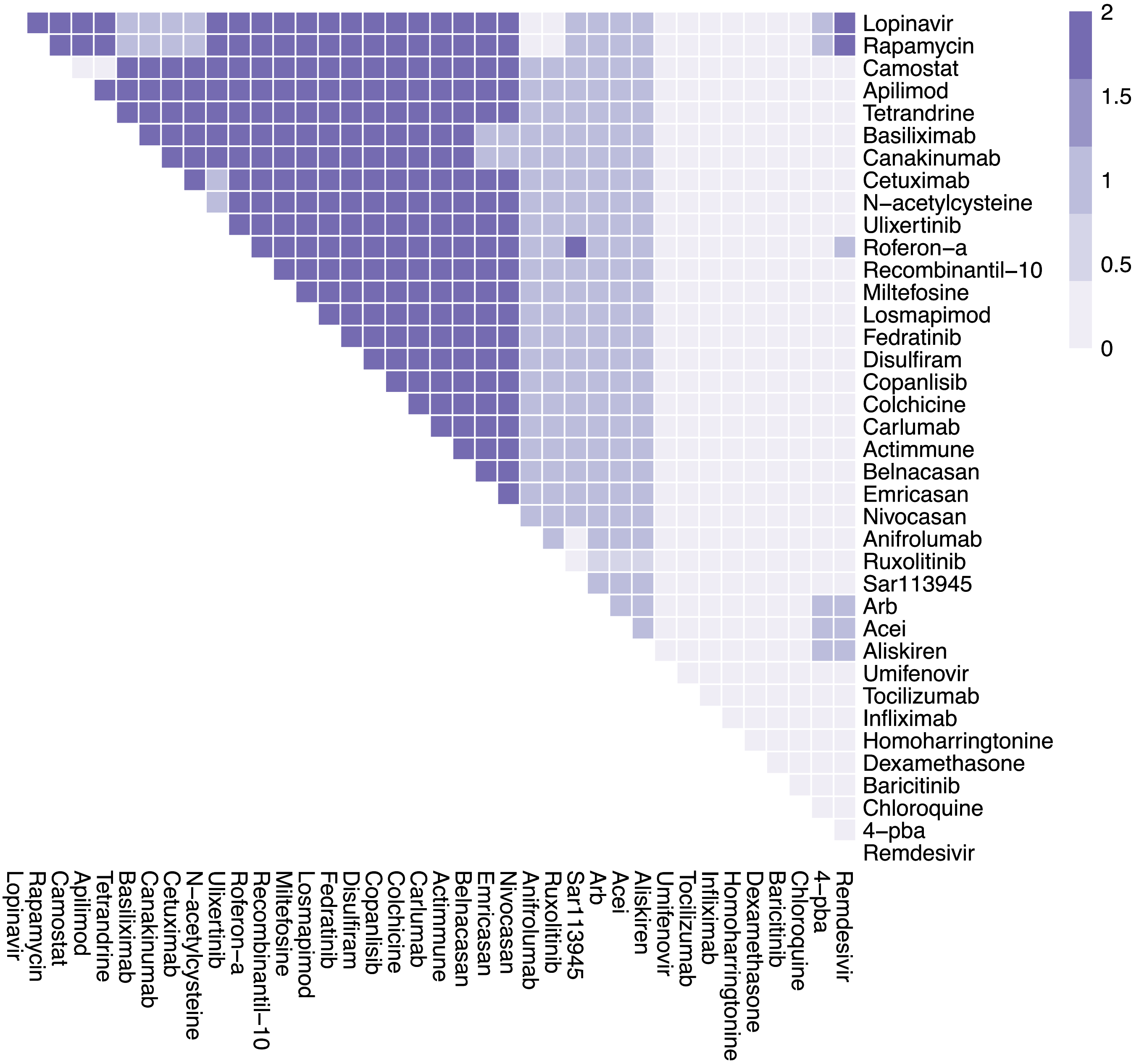

Supplementary Figure 2n. Effects of combination therapy (38 drugs that have passed phase II trials) on T-cell Infiltration in mild COVID-19. The colour of the square corresponds to the level of the *T-cellInfiltration* node at steady state under treatment by the drugs indicated on the x- and y-axis. The plot shows drugs with known safety profiles, having completed phase II clinical trials (Supplementary Table 5).

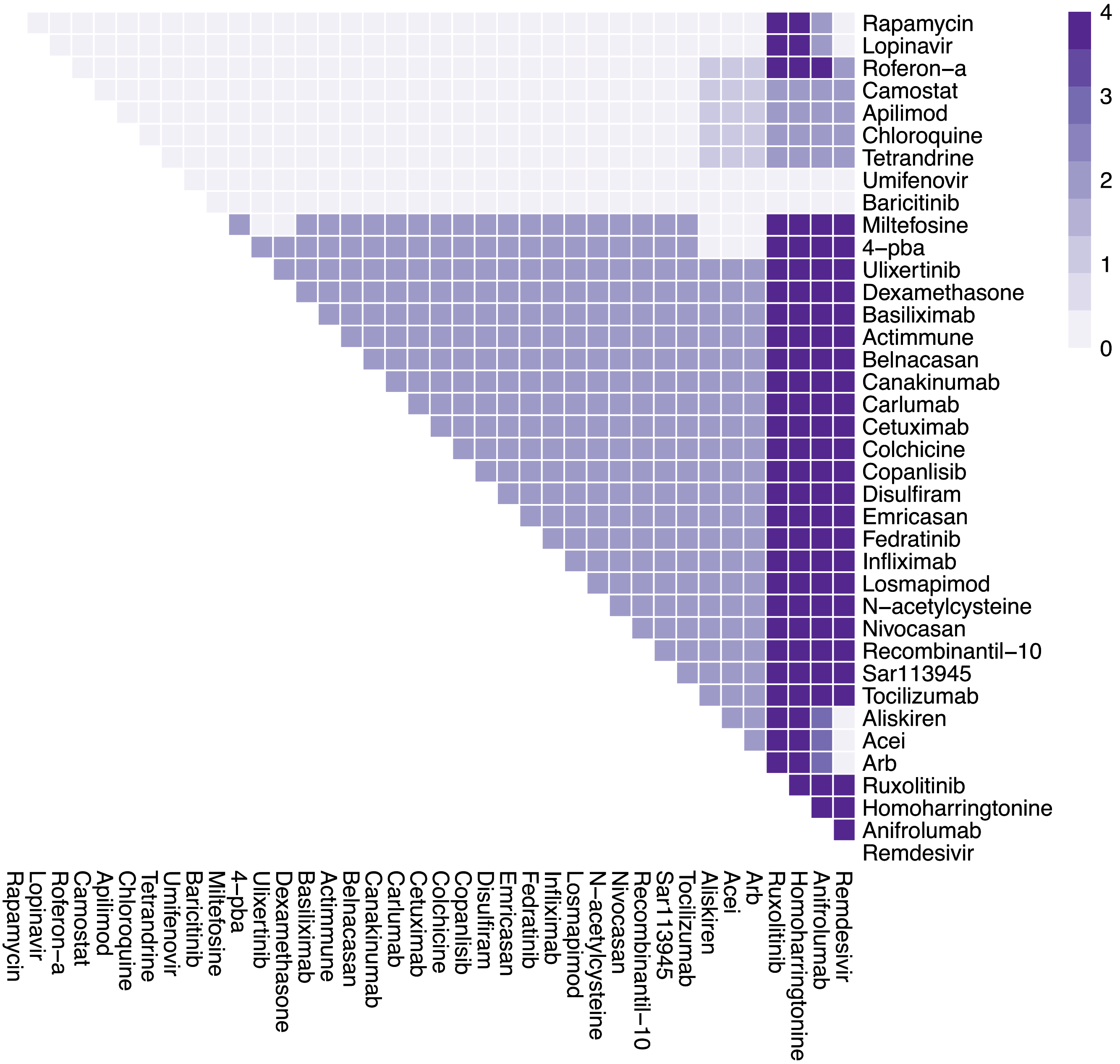

Supplementary Figure 2o. Effects of combination therapy (38 drugs that have passed phase II trials) on Viral Entry in mild COVID-19. The colour of the square corresponds to the level of the *ViralEntry* node at steady state under treatment by the drugs indicated on the x- and y-axis. The plot shows drugs with known safety profiles, having completed phase II clinical trials (Supplementary Table 5).

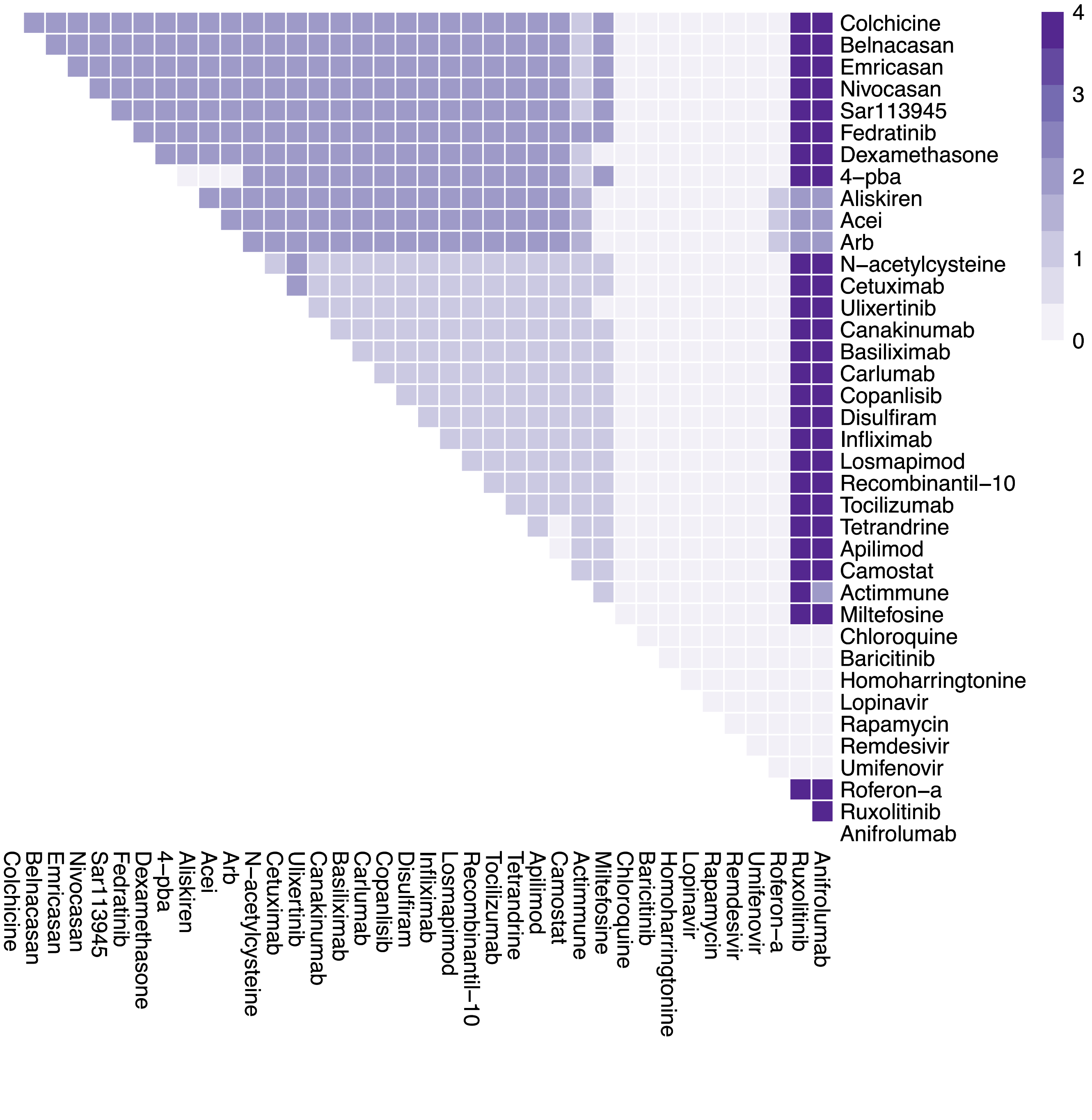

Supplementary Figure 2p. Effects of combination therapy (38 drugs that have passed phase II trials) on Viral Replication in mild COVID-19. The colour of the square corresponds to the level of the *ViralReplication* node at steady state under treatment by the drugs indicated on the x- and y-axis. The plot shows drugs with known safety profiles, having completed phase II clinical trials (Supplementary Table 5).

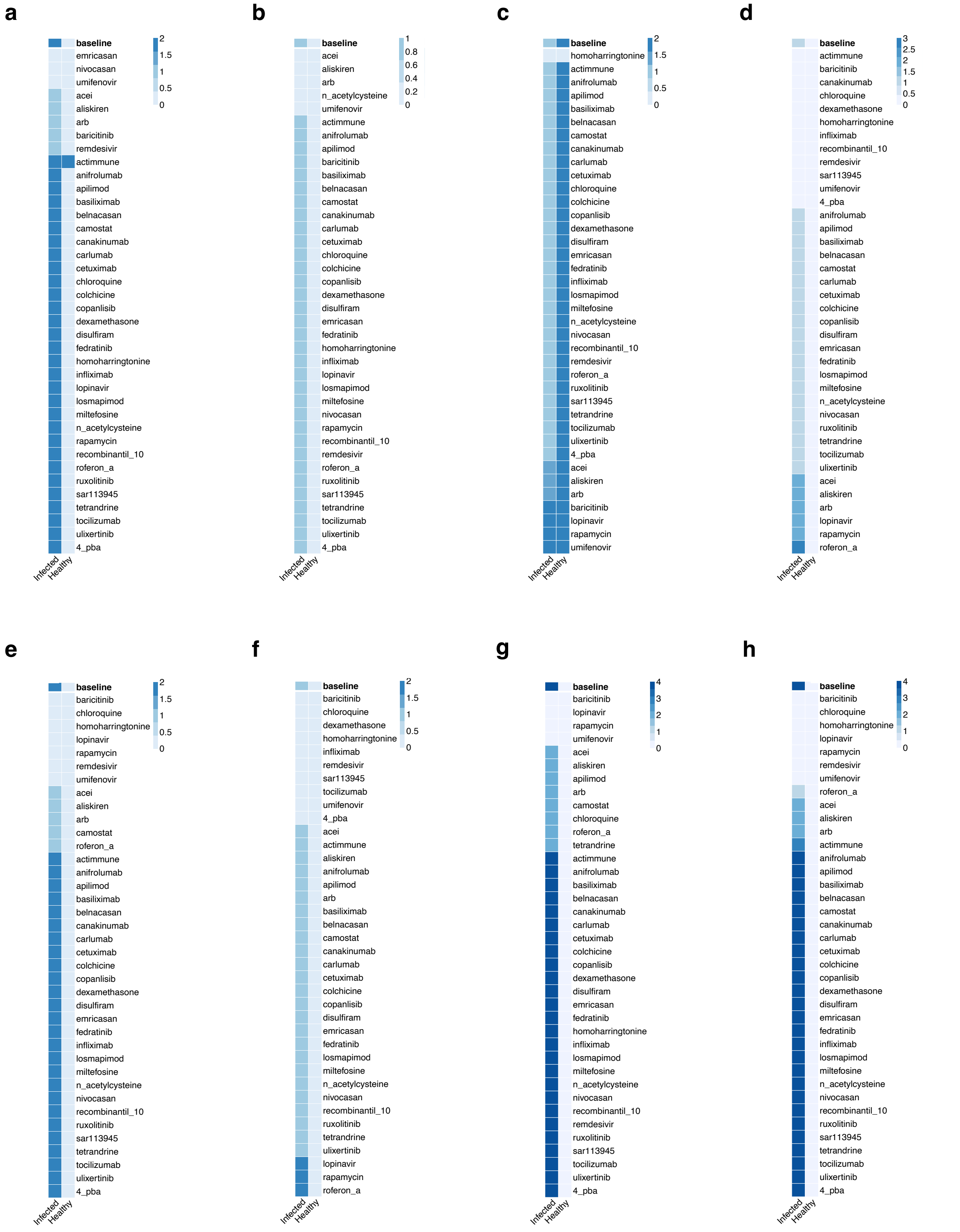

Supplementary Figure 3 a-h. Effects of single therapies (38 drugs that have passed phase II trials) in early stage severe COVID-19. Columns represent infected and healthy conditions, rows represent drug tested including only drugs with known safety profiles, having completed phase II clinical trials (Supplementary Table 5). Showing the effect on: (**a**) Cell Death. (**b**) Fibrosis. (**c**) Host Protein Synthesis. (**d**) Inflammation. (**e**) Syncytia Formation. (**f**) T-cell Infiltration. (**g**) Viral Entry. (**h**) Viral Replication.

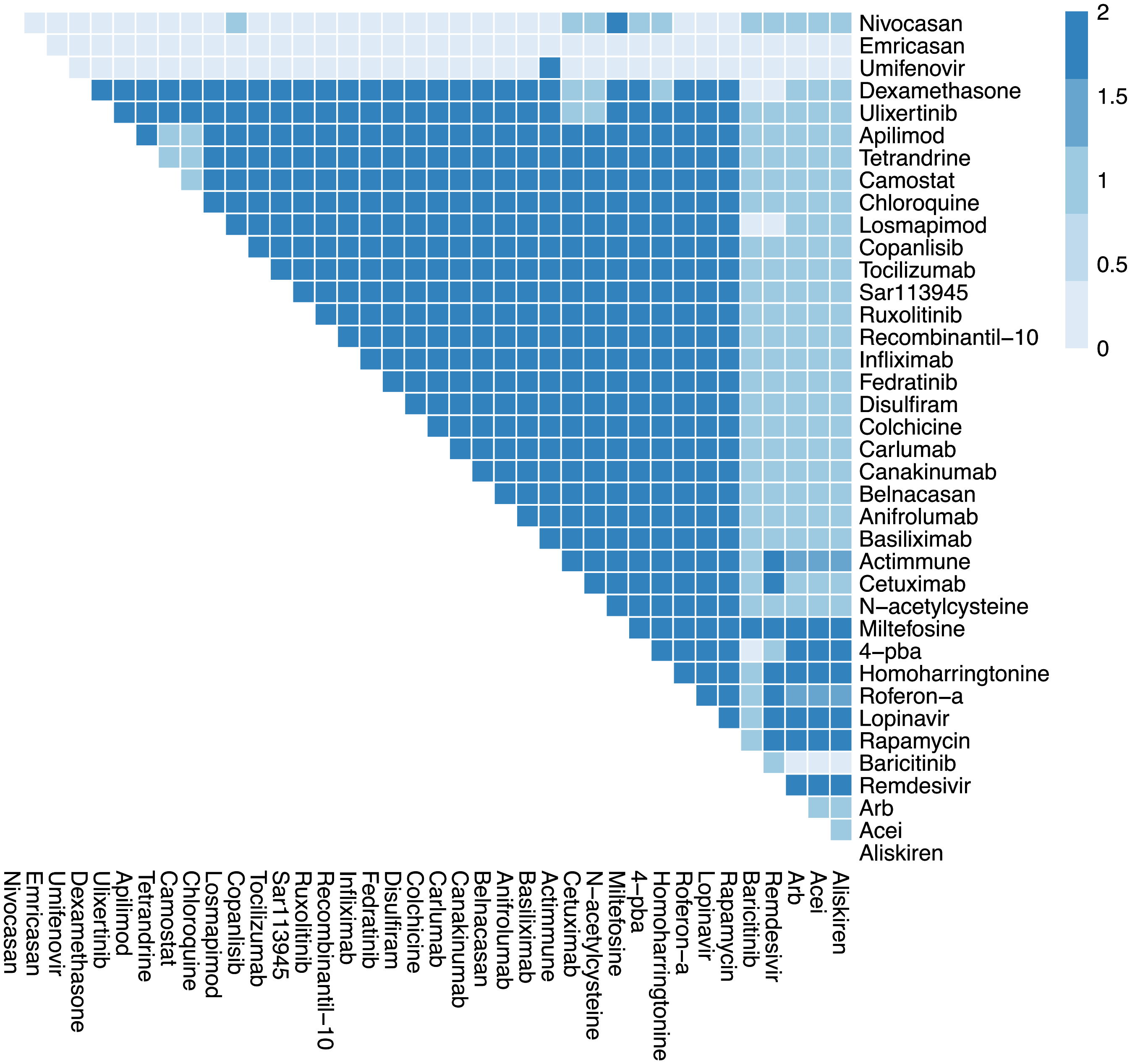

Supplementary Figure 3i. Effects of combination therapy (38 drugs that have passed phase II trials) on Cell Death in early stage, severe COVID-19. The colour of the square corresponds to the level of the *CellDeath* node at steady state under treatment by the drugs indicated on the x- and y-axis. The plot shows drugs with known safety profiles, having completed phase II clinical trials (Supplementary Table 5).

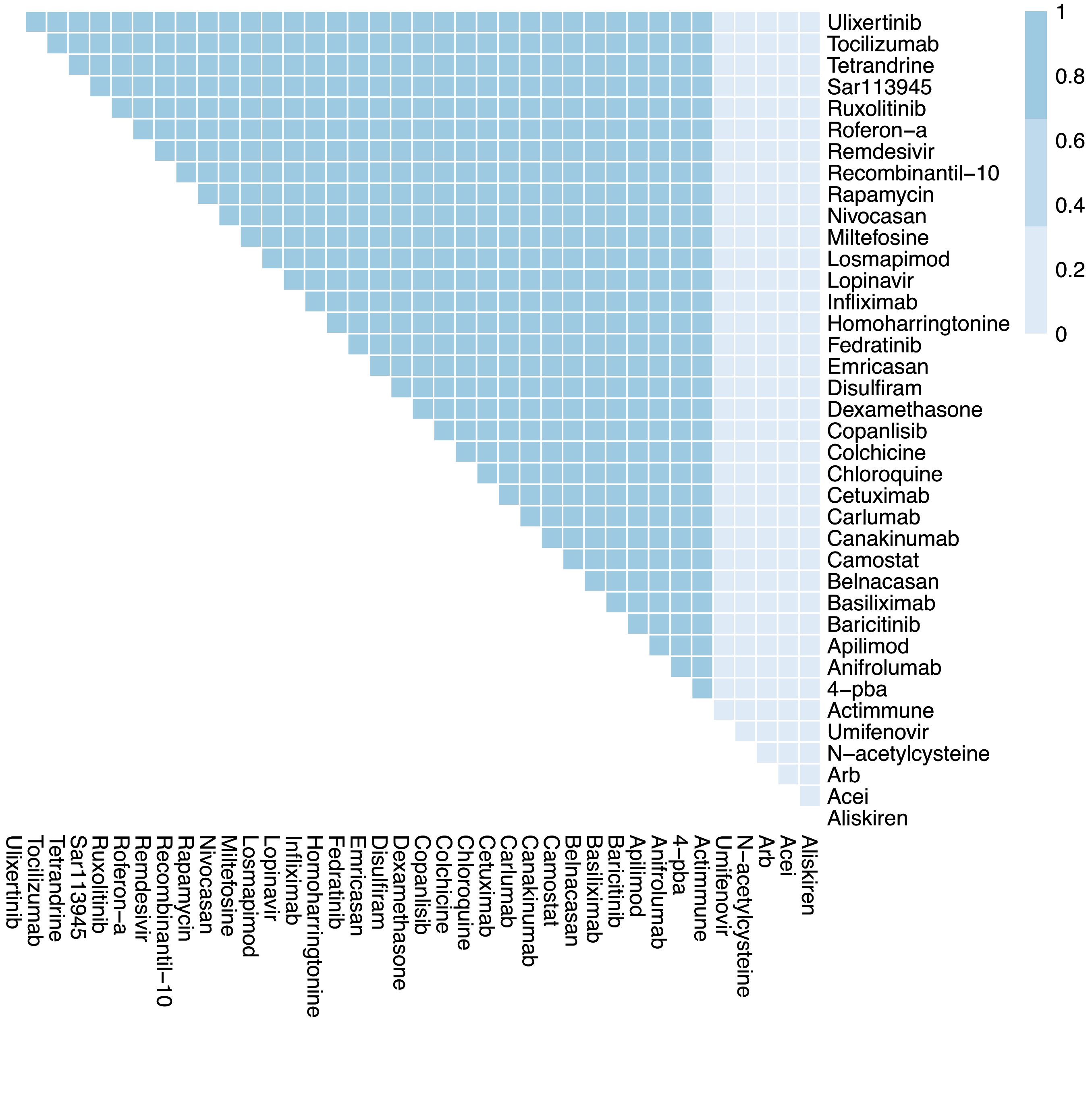

Supplementary Figure 3j. Effects of combination therapy (38 drugs that have passed phase II trials) on Fibrosis in early stage, severe COVID-19. The colour of the square corresponds to the level of the *Fibrosis* node at steady state under treatment by the drugs indicated on the x- and y-axis. The plot shows drugs with known safety profiles, having completed phase II clinical trials (Supplementary Table 5).

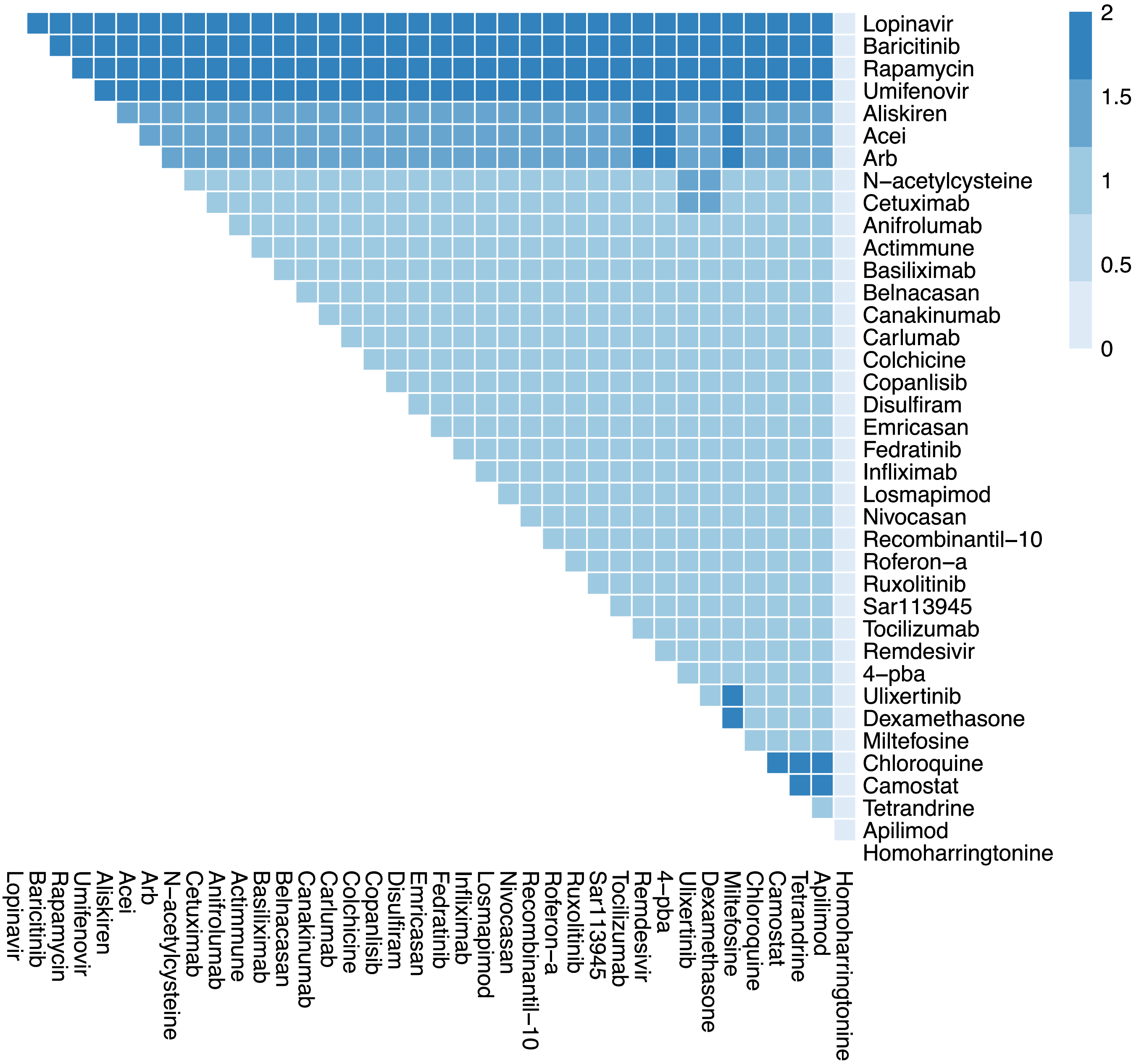

Supplementary Figure 3k. Effects of combination therapy (38 drugs that have passed phase II trials) on Host Protein Synthesis in early stage, severe COVID-19. The colour of the square corresponds to the level of the *HostProteinSynthesis* node at steady state under treatment by the drugs indicated on the x- and y-axis. The plot shows drugs with known safety profiles, having completed phase II clinical trials (Supplementary Table 5).

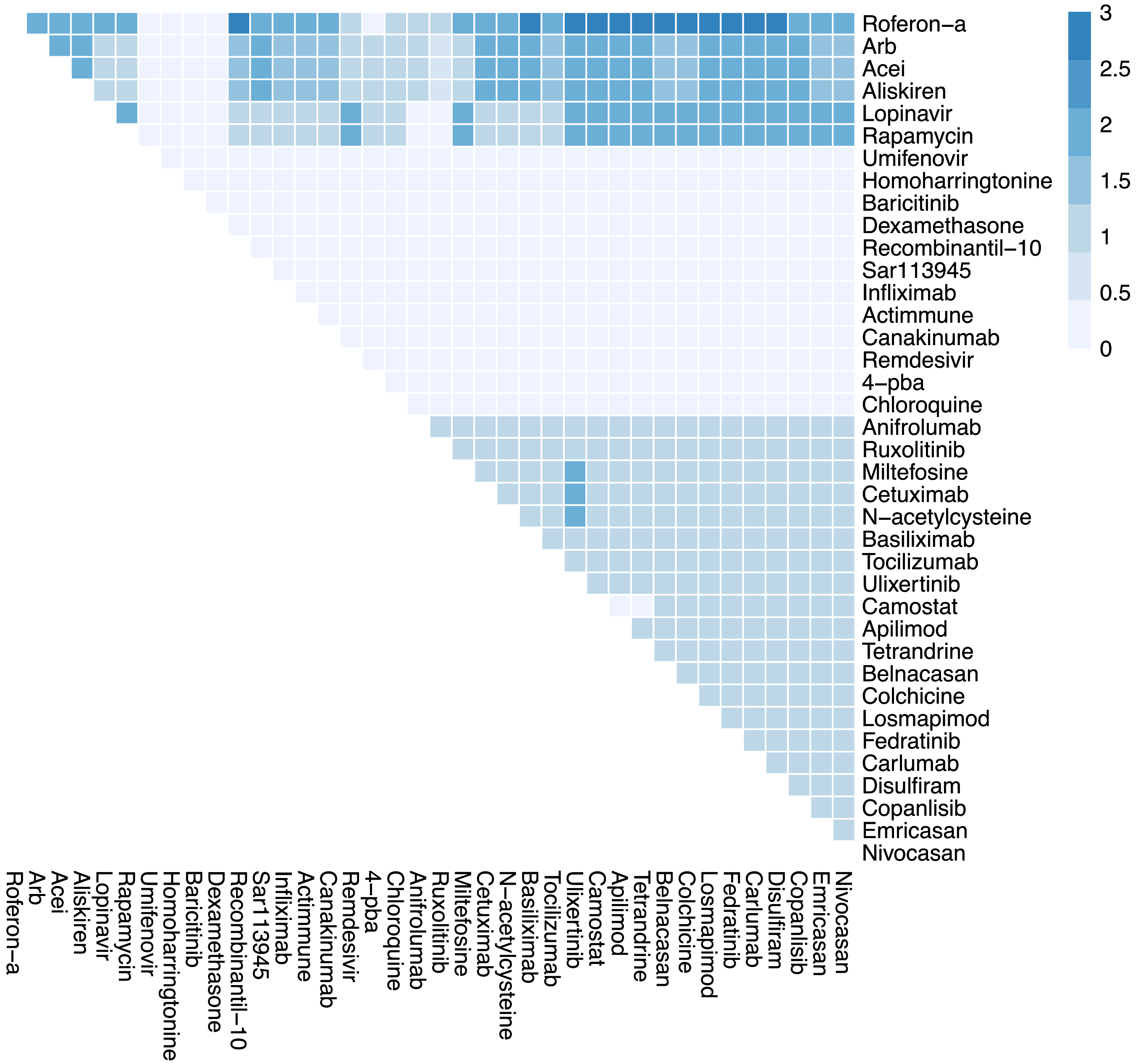

Supplementary Figure 3I. Effects of combination therapy (38 drugs that have passed phase II trials) on Inflammation in early stage, severe COVID-19. The colour of the square corresponds to the level of the *Inflammation* node at steady state under treatment by the drugs indicated on the x- and y-axis. The plot shows drugs with known safety profiles, having completed phase II clinical trials (Supplementary Table 5).

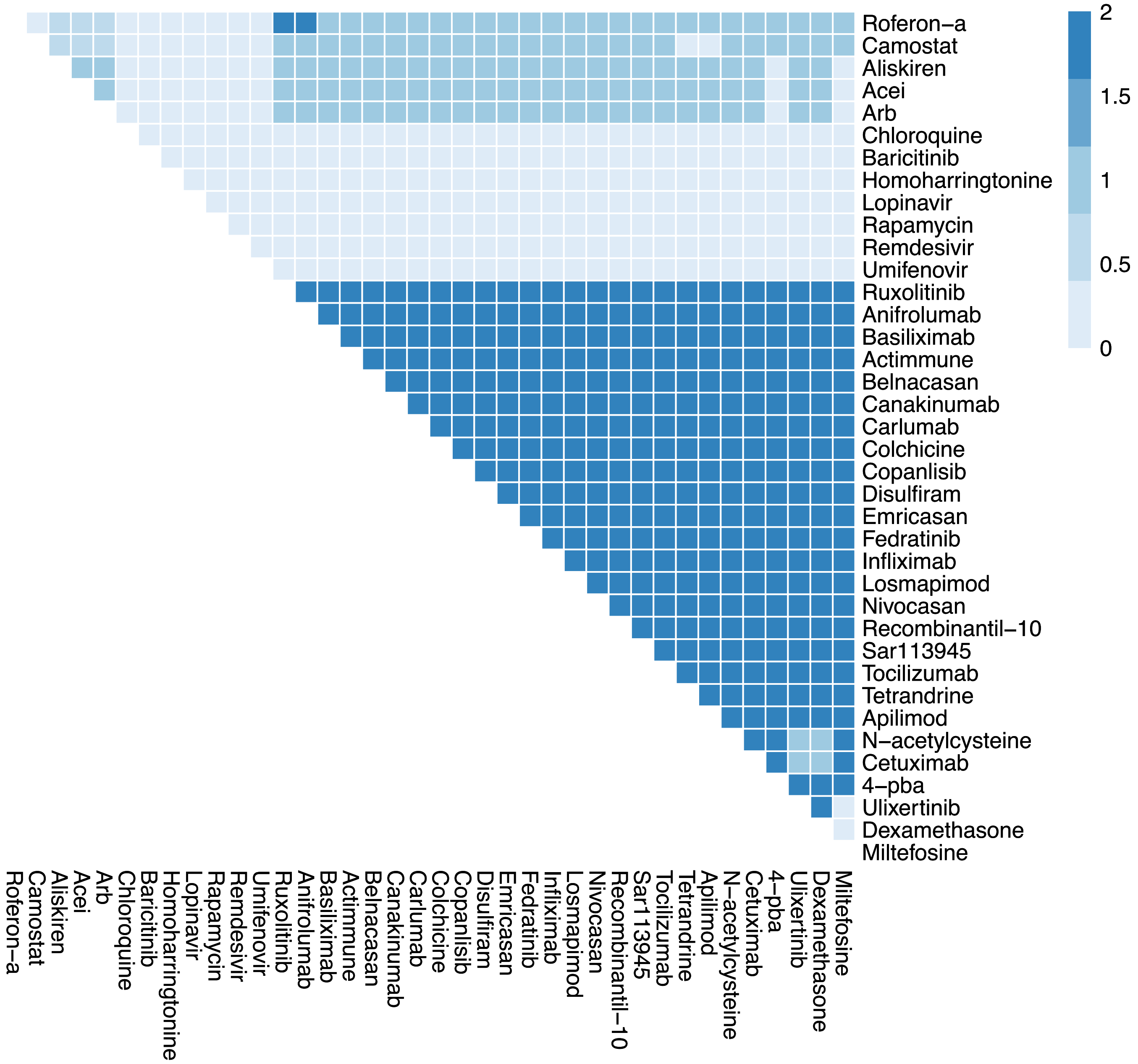

Supplementary Figure 3m. Effects of combination therapy (38 drugs that have passed phase II trials) on Syncytia Formation in early stage, severe COVID-19. The colour of the square corresponds to the level of the *SyncytiaFormation* node at steady state under treatment by the drugs indicated on the x- and y-axis. The plot shows drugs with known safety profiles, having completed phase II clinical trials (Supplementary Table 5).

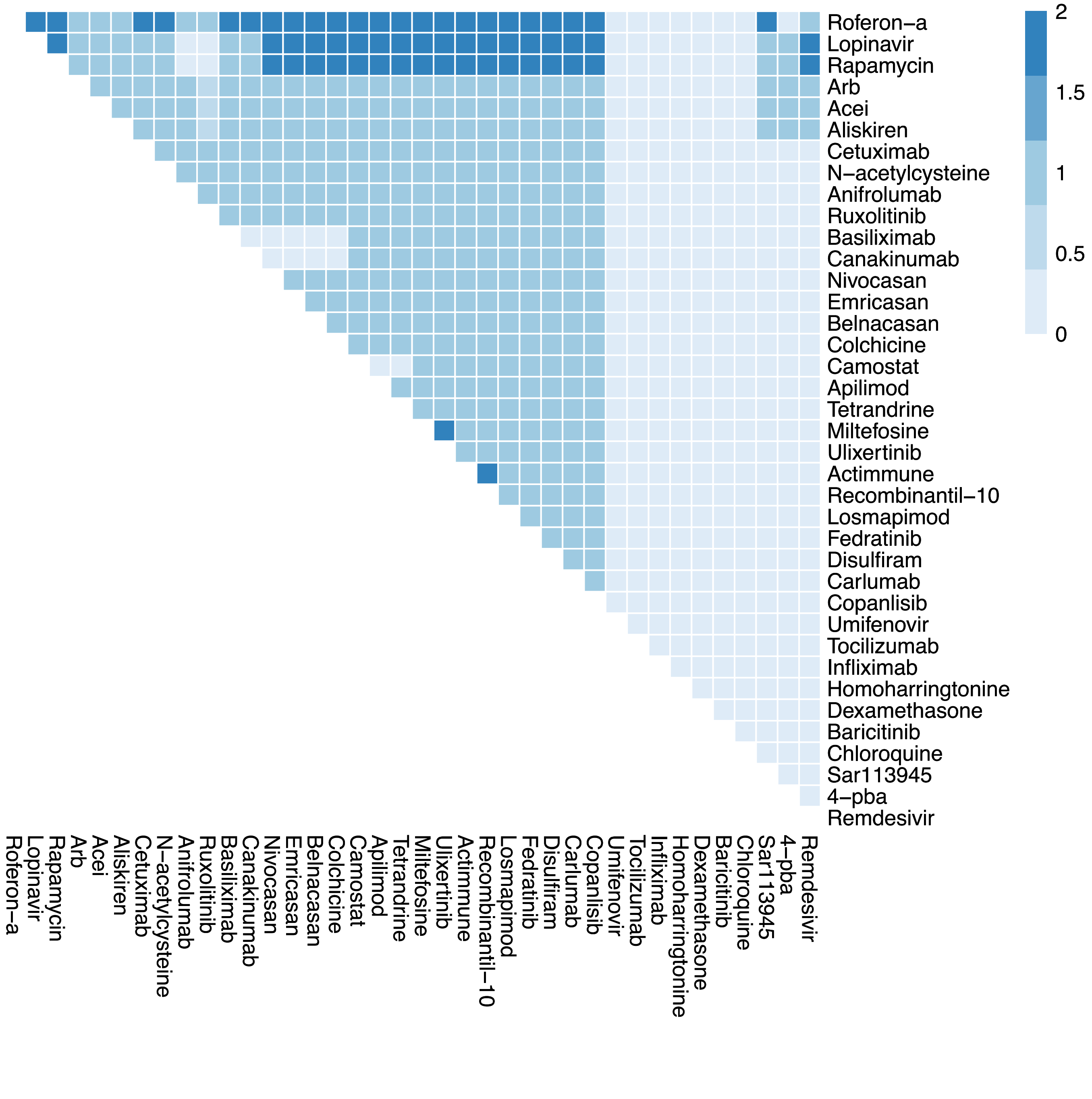

Supplementary Figure 3n. Effects of combination therapy (38 drugs that have passed phase II trials) on T-cell Infiltration in early stage, severe COVID-19. The colour of the square corresponds to the level of the *T-cellInfiltration* node at steady state under treatment by the drugs indicated on the x- and y-axis. The plot shows drugs with known safety profiles, having completed phase II clinical trials (Supplementary Table 5).

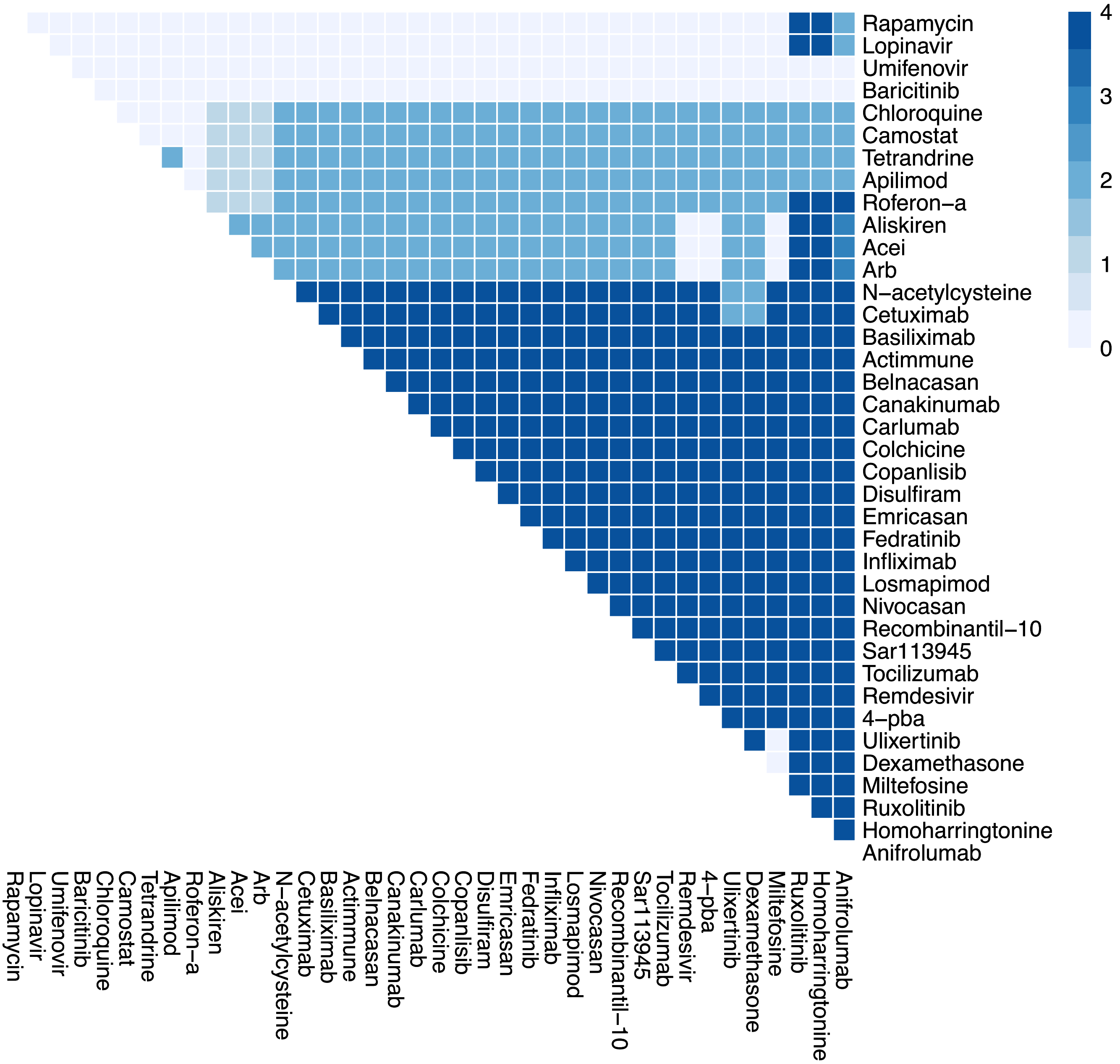

Supplementary Figure 3o. Effects of combination therapy (38 drugs that have passed phase II trials) on Viral Entry in early stage, severe COVID-19. The colour of the square corresponds to the level of the *ViralEntry* node at steady state under treatment by the drugs indicated on the x- and y-axis. The plot shows drugs with known safety profiles, having completed phase II clinical trials (Supplementary Table 5).

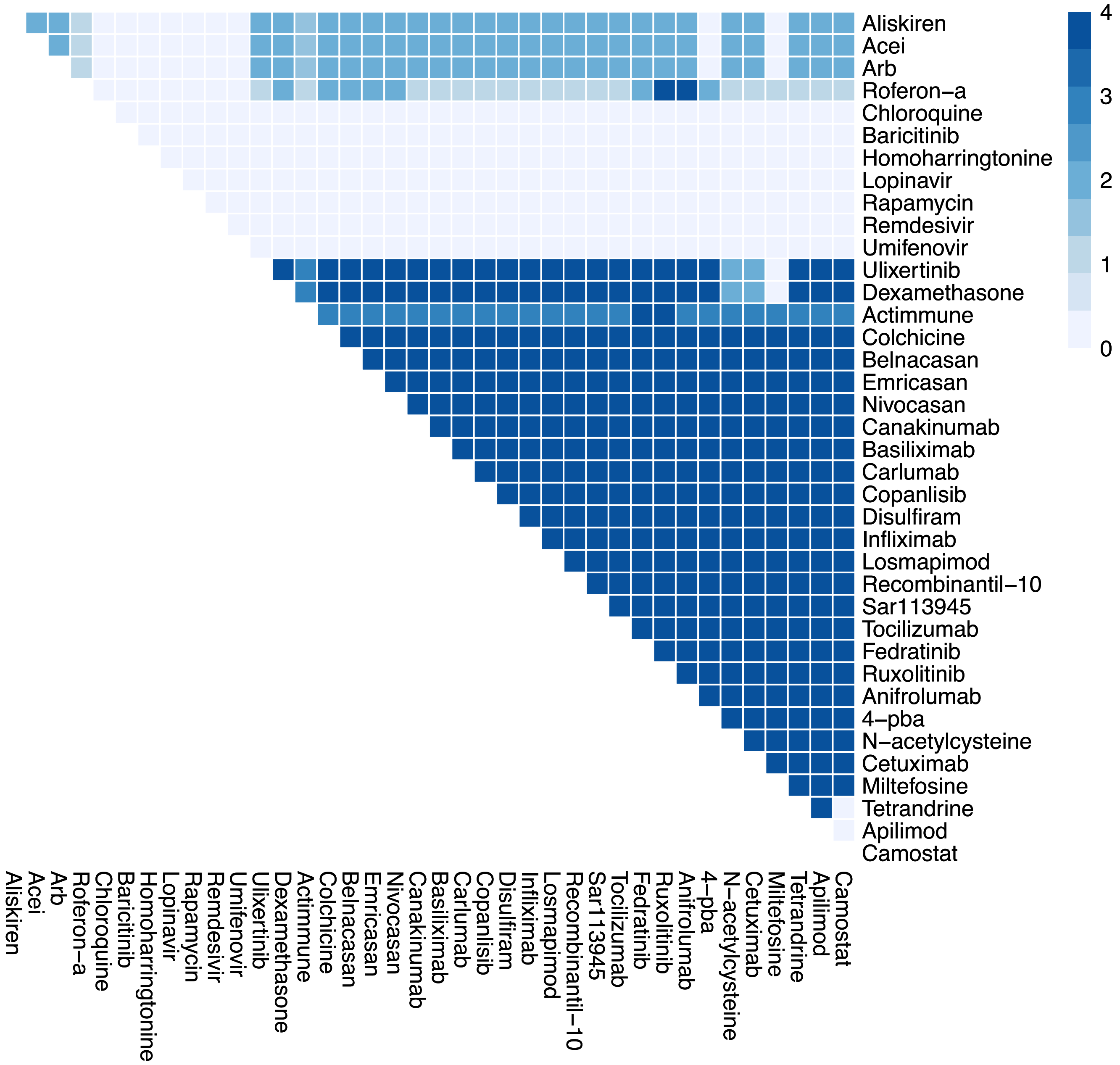

Supplementary Figure 3p. Effects of combination therapy (38 drugs that have passed phase II trials) on Viral Replication in early stage, severe COVID-19. The colour of the square corresponds to the level of the *ViralReplication* node at steady state under treatment by the drugs indicated on the x- and y-axis. The plot shows drugs with known safety profiles, having completed phase II clinical trials (Supplementary Table 5).

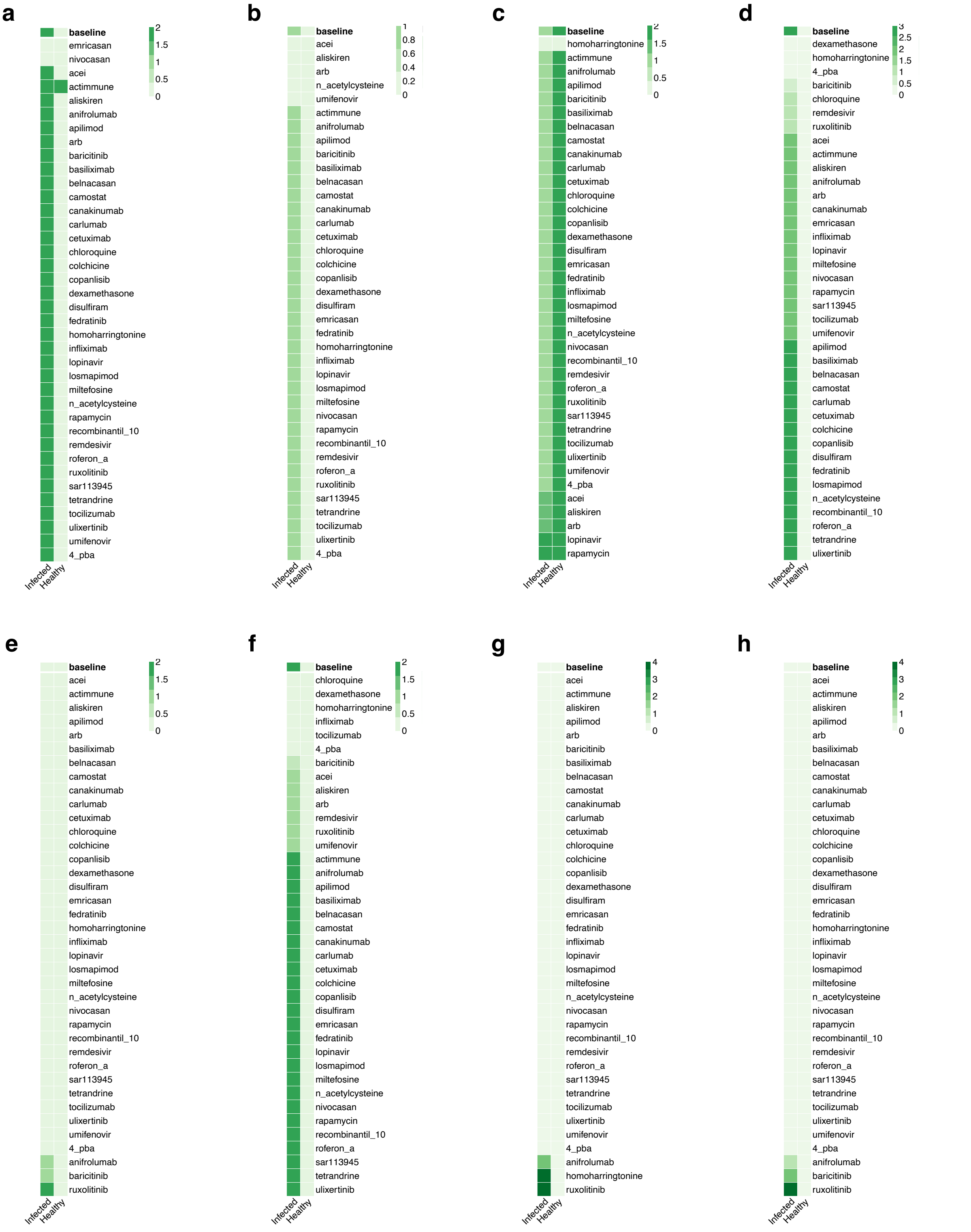

Supplementary Figure 4 a-h. Effects of single therapies (38 drugs that have passed phase II trials) in late stage, severe COVID-19. Columns represent infected and healthy conditions, rows represent drugs tested including only drugs with known safety profiles, having completed phase II clinical trials (Supplementary Table 5). Showing the effect on: (**a**) Cell Death. (**b**) Fibrosis. (c) Host Protein Synthesis. (**d**) Inflammation. (**e**) Syncytia Formation. (**f**) T-cell Infiltration. (**g**) Viral Entry. (**h**) Viral Replication.

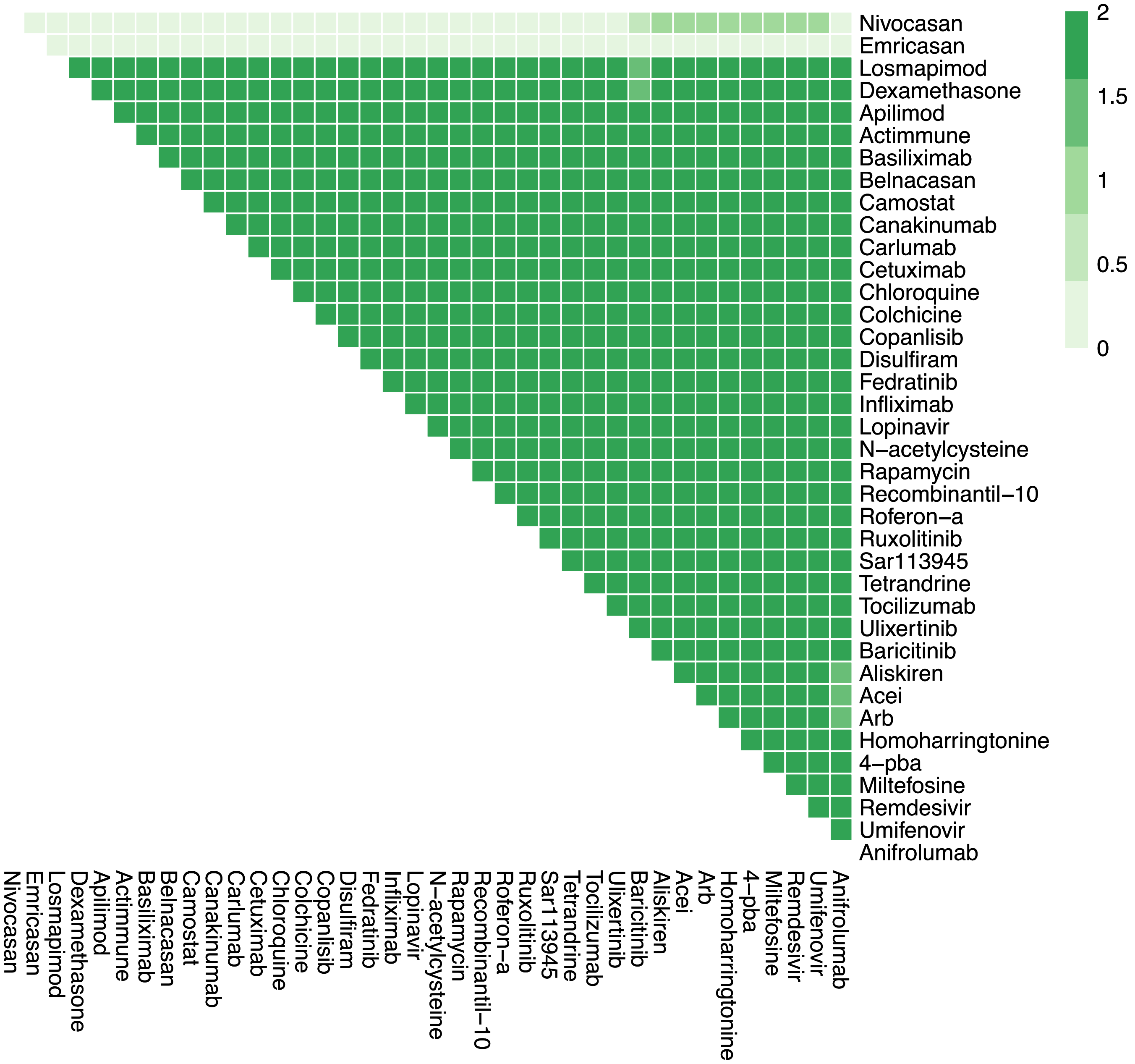

Supplementary Figure 4i. Effects of combination therapy (38 drugs that have passed phase II trials) on Cell Death in late stage, severe COVID-19. The colour of the square corresponds to the level of the *CellDeath* node at steady state under treatment by the drugs indicated on the x- and y-axis. The plot shows drugs with known safety profiles, having completed phase II clinical trials (Supplementary Table 5).

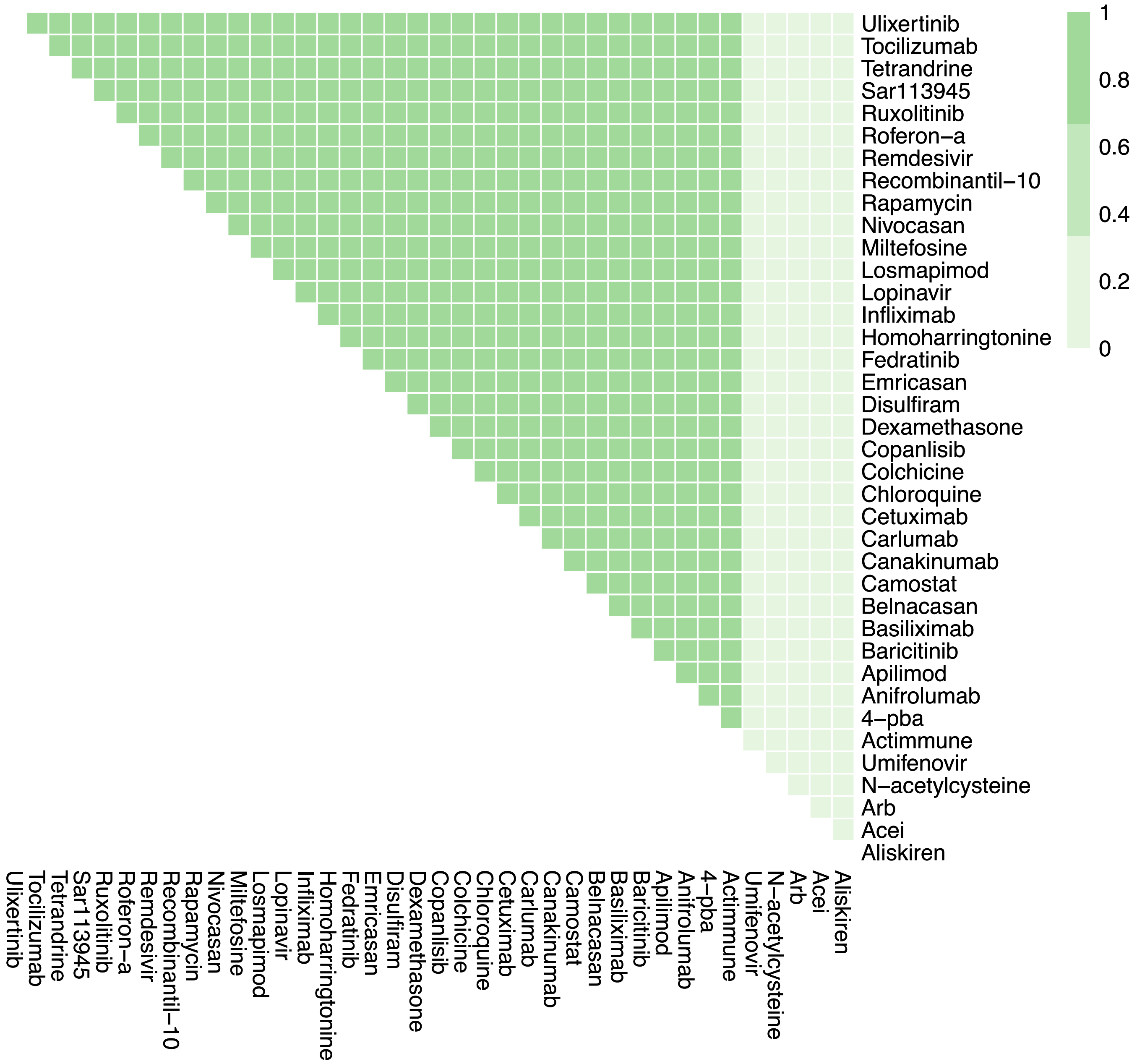

Supplementary Figure 4j. Effects of combination therapy (38 drugs that have passed phase II trials) on Fibrosis in late stage, severe COVID-19. The colour of the square corresponds to the level of the *Fibrosis* node at steady state under treatment by the drugs indicated on the x- and y-axis. The plot shows drugs with known safety profiles, having completed phase II clinical trials (Supplementary Table 5).

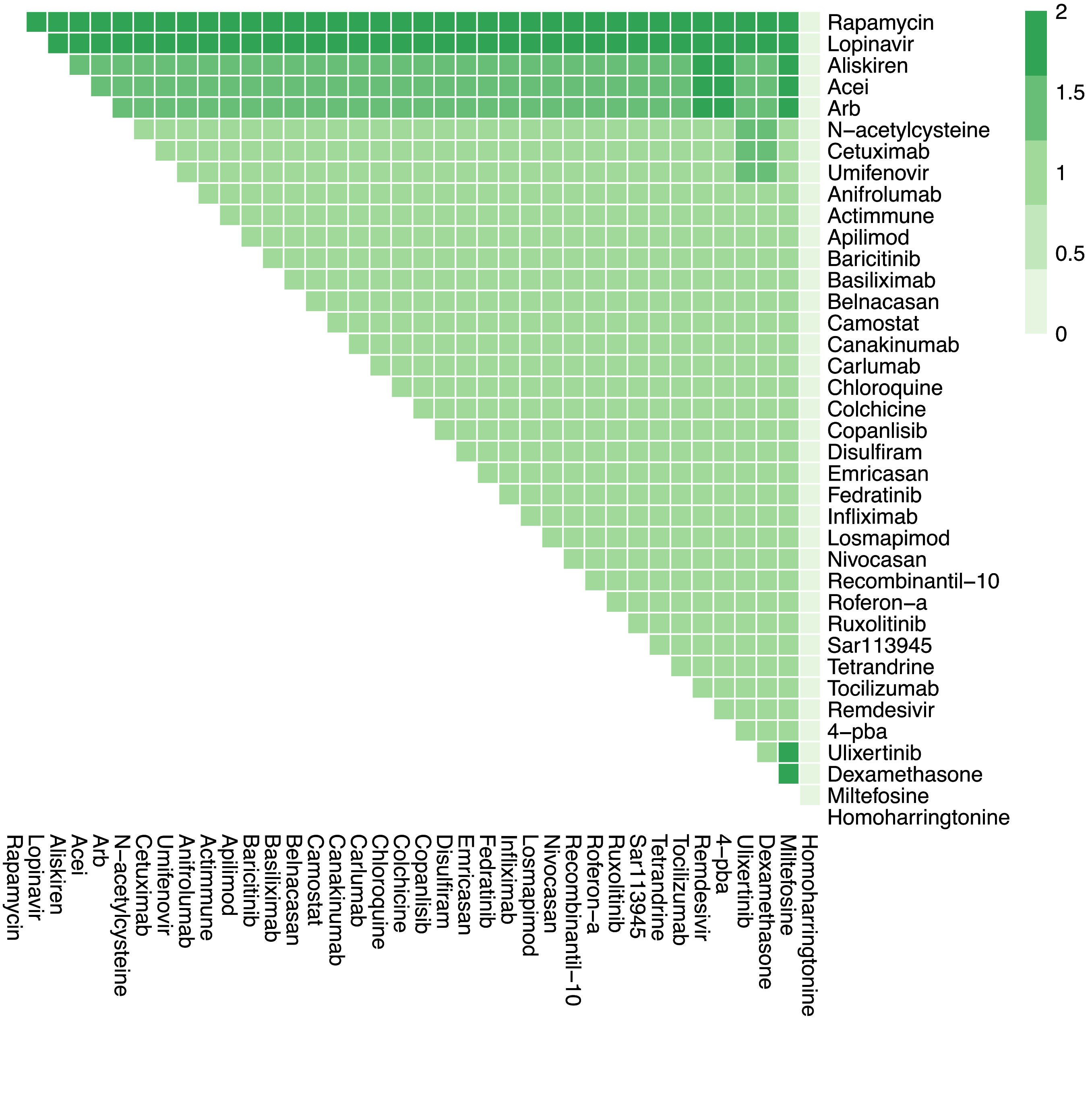

Supplementary Figure 4k. Effects of combination therapy (38 drugs that have passed phase II trials) on Host Protein Synthesis in late stage, severe COVID-19. The colour of the square corresponds to the level of the *HostProteinSynthesis* node at steady state under treatment by the drugs indicated on the x- and y-axis. The plot shows drugs with known safety profiles, having completed phase II clinical trials (Supplementary Table 5).

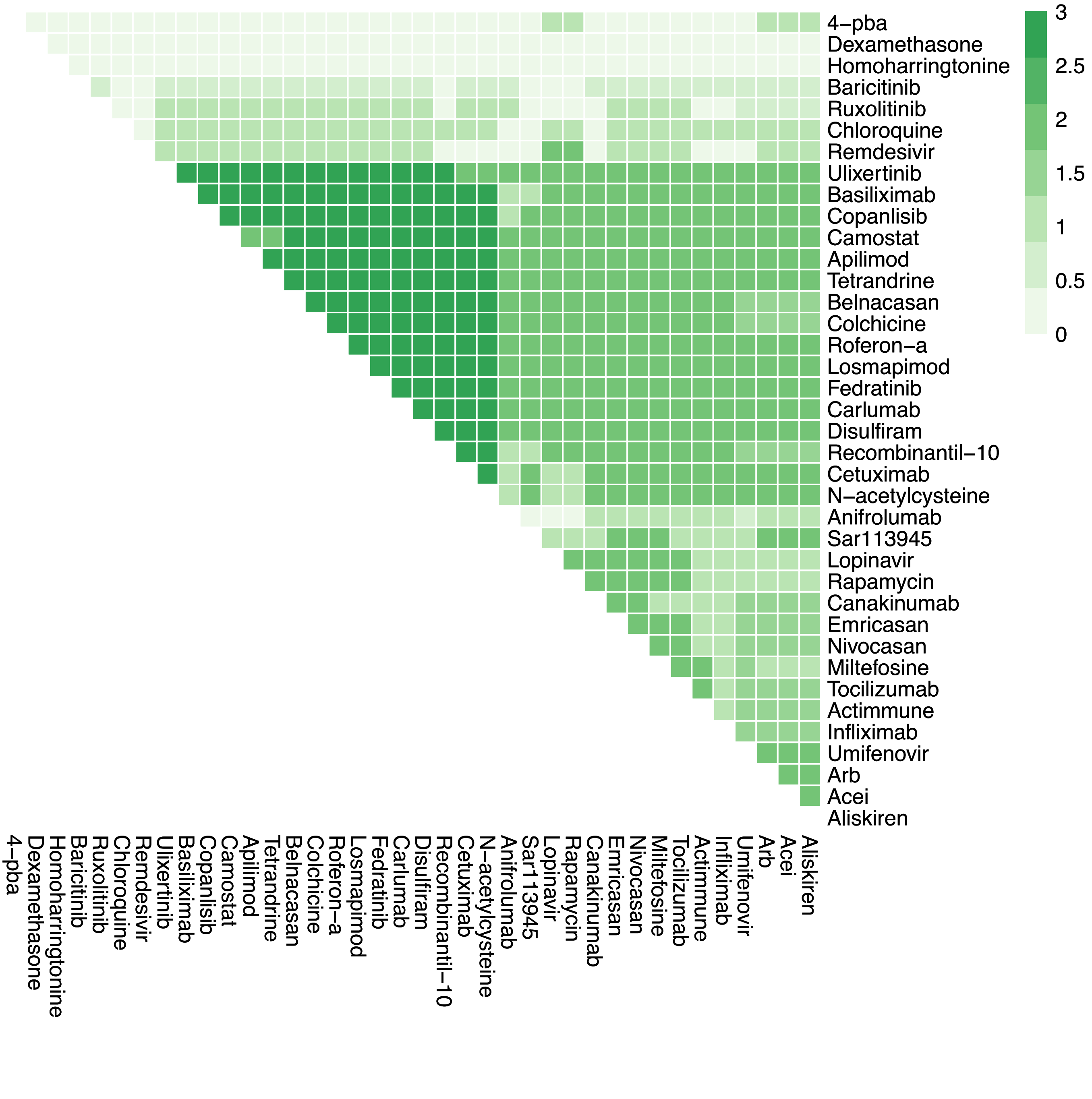

Supplementary Figure 4I. Effects of combination therapy (38 drugs that have passed phase II trials) on Inflammation in late stage, severe COVID-19. The colour of the square corresponds to the level of the *Inflammation* node at steady state under treatment by the drugs indicated on the x- and y-axis. The plot shows drugs with known safety profiles, having completed phase II clinical trials (Supplementary Table 5).

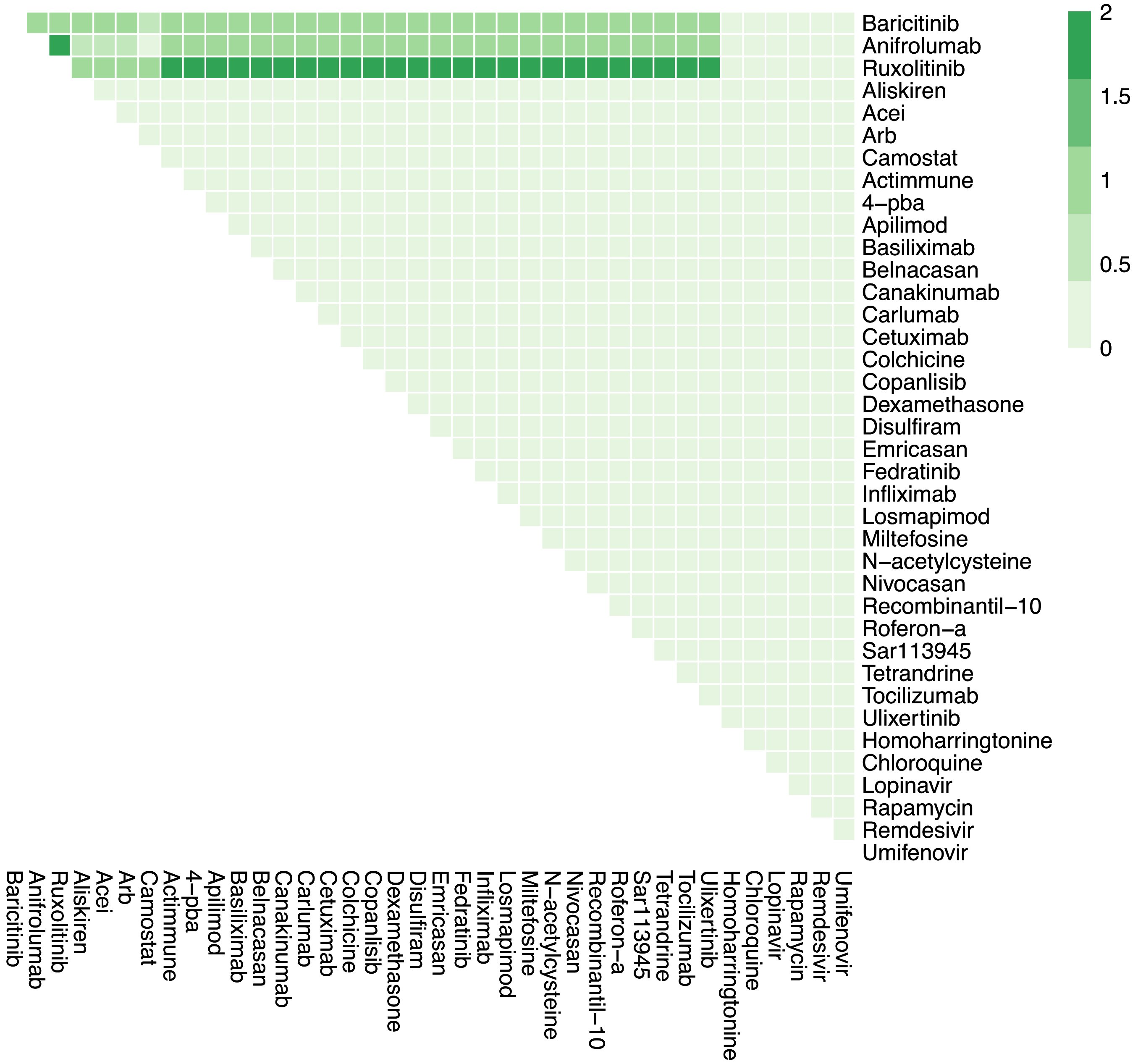

Supplementary Figure 4m. Effects of combination therapy (38 drugs that have passed phase II trials) on Syncytia Formation in late stage, severe COVID-19. The colour of the square corresponds to the level of the *SyncytiaFormation* node at steady state under treatment by the drugs indicated on the x- and y-axis. The plot shows drugs with known safety profiles, having completed phase II clinical trials (Supplementary Table 5).

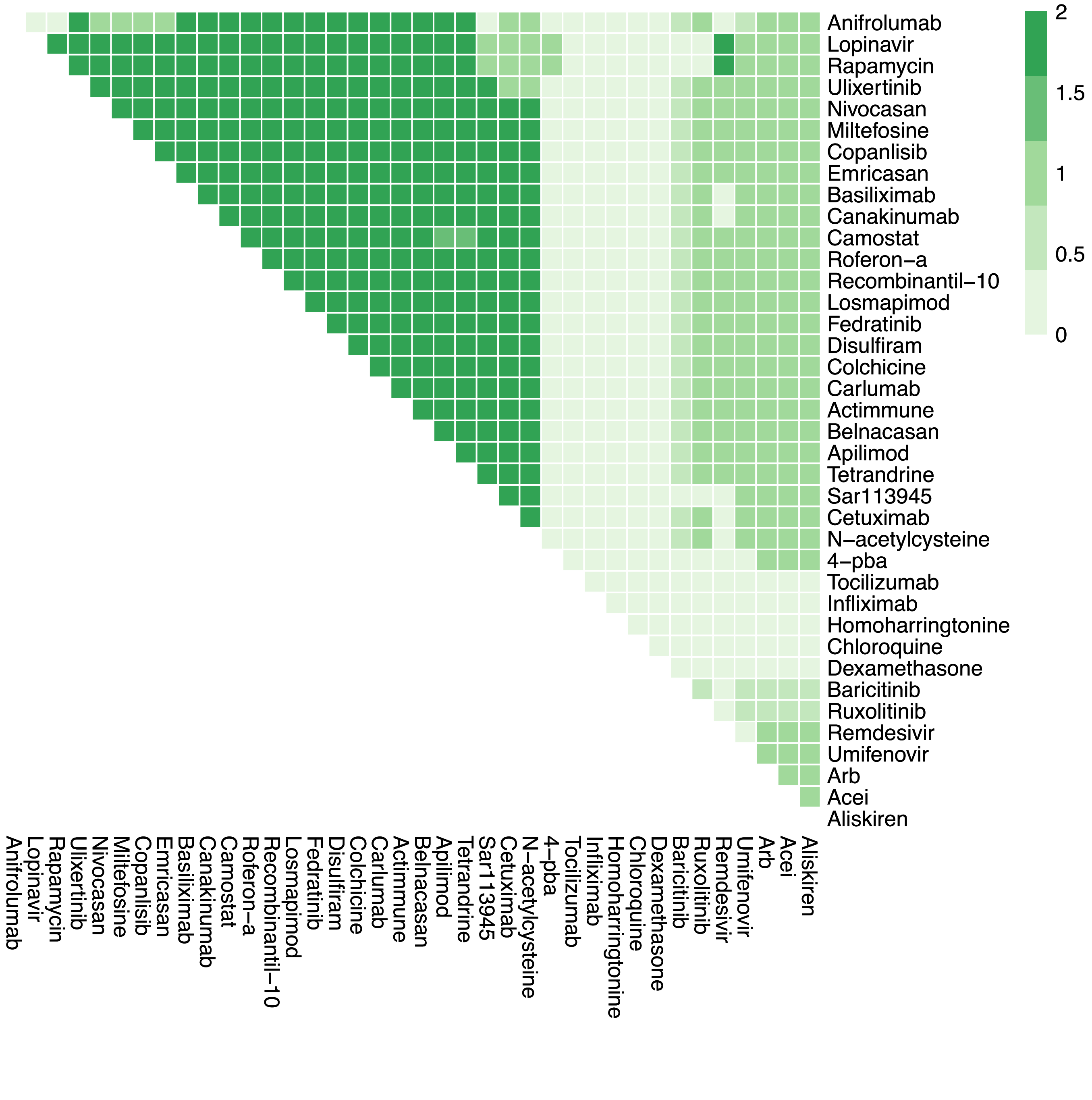

Supplementary Figure 4n. Effects of combination therapy (38 drugs that have passed phase II trials) on T-cell Infiltration in late stage, severe COVID-19. The colour of the square corresponds to the level of the *T-cellInfiltration* node at steady state under treatment by the drugs indicated on the x- and y-axis. The plot shows drugs with known safety profiles, having completed phase II clinical trials (Supplementary Table 5).

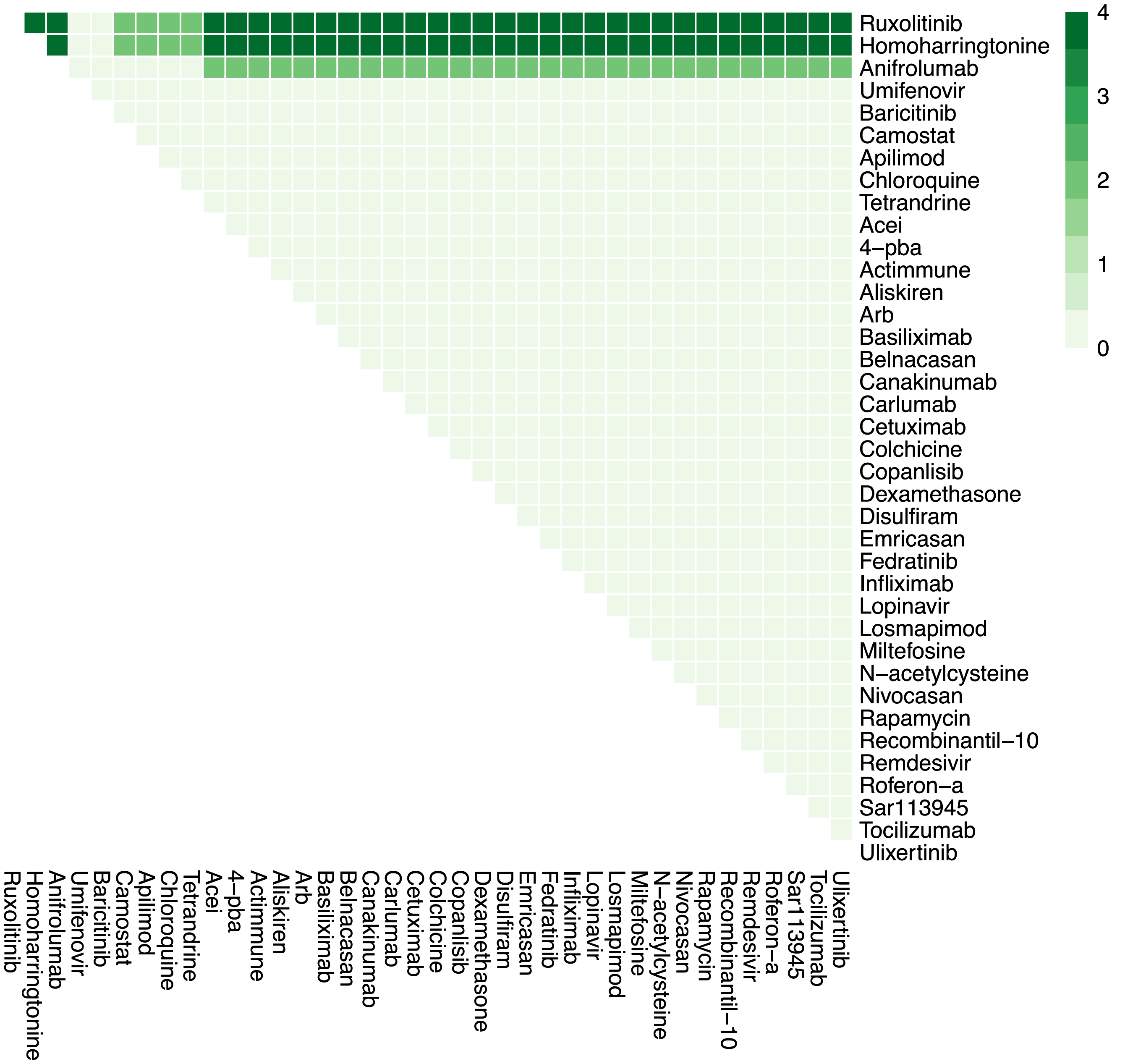

Supplementary Figure 4o. Effects of combination therapy (38 drugs that have passed phase II trials) on Viral Entry in late stage, severe COVID-19. The colour of the square corresponds to the level of the *ViralEntry* node at steady state under treatment by the drugs indicated on the x- and y-axis. The plot shows drugs with known safety profiles, having completed phase II clinical trials (Supplementary Table 5).

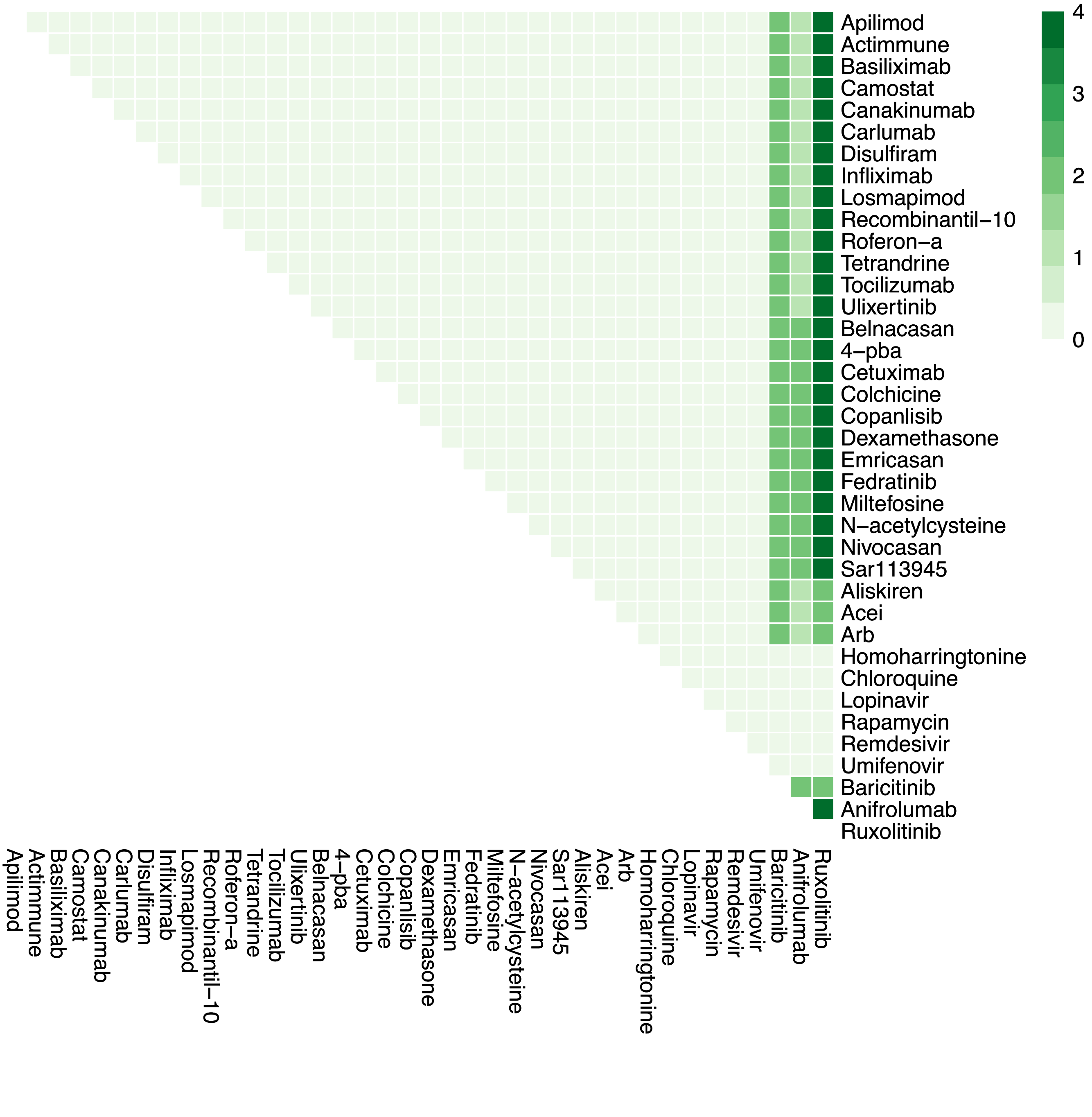

Supplementary Figure 4p. Effects of combination therapy (38 drugs that have passed phase II trials) on Viral Replication in late stage, severe COVID-19. The colour of the square corresponds to the level of the *Viral Replication* node at steady state under treatment by the drugs indicated on the x- and y-axis. The plot shows drugs with known safety profiles, having completed phase II clinical trials (Supplementary Table 5).

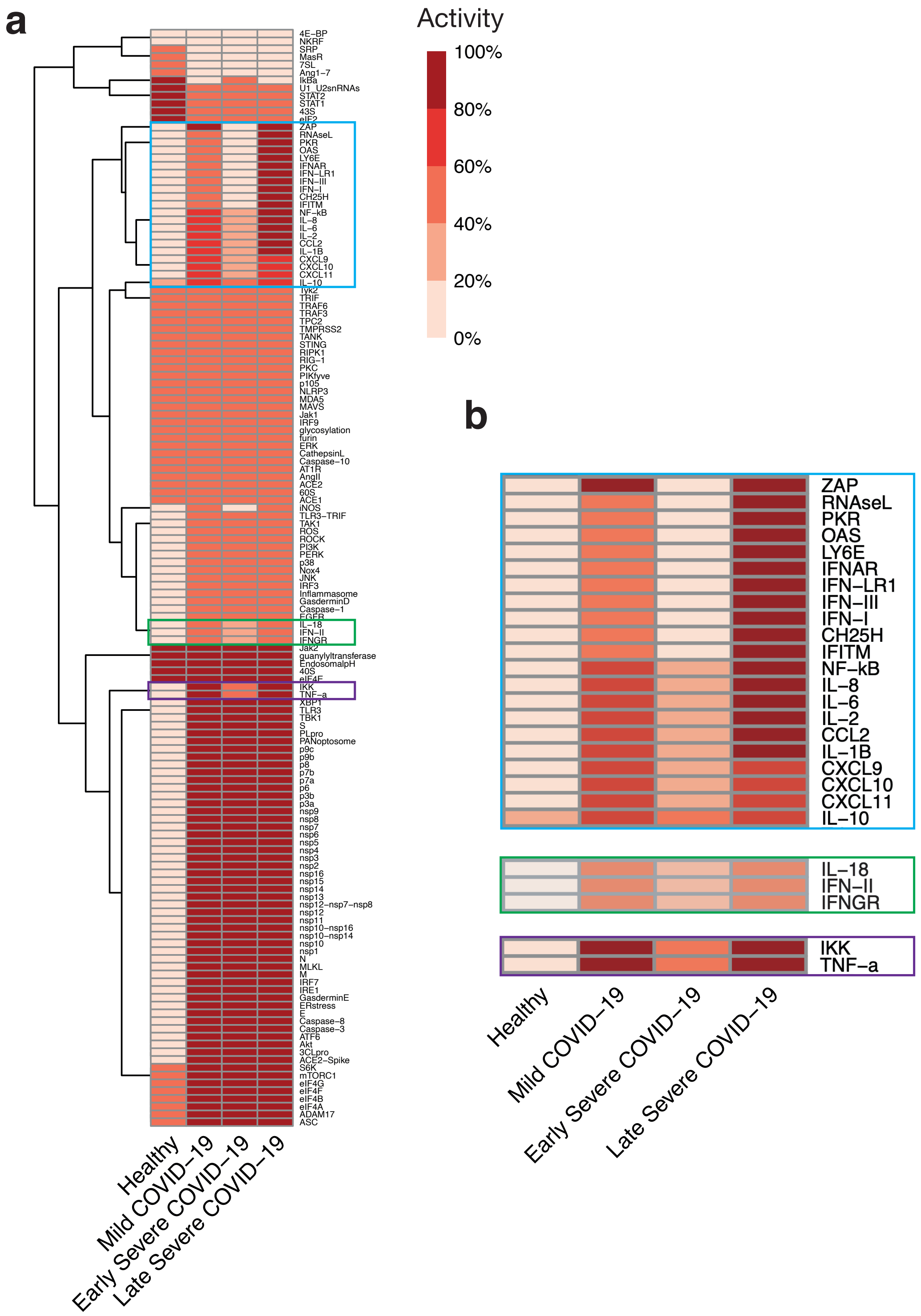

Supplementary Figure 5. Differential characteristics of Mild, Early Severe and Late Severe COVID-19 (**a**) Hierarchical clustering heatmap representing the steady state values of each node in the network model for mild disease, and early and late severe disease (healthy controls for comparison). (**b**) Zoom in plot of areas in (**a**) that vary between disease states. All nodes normalised to maximal level of respective nodes, and range between 0-100%.

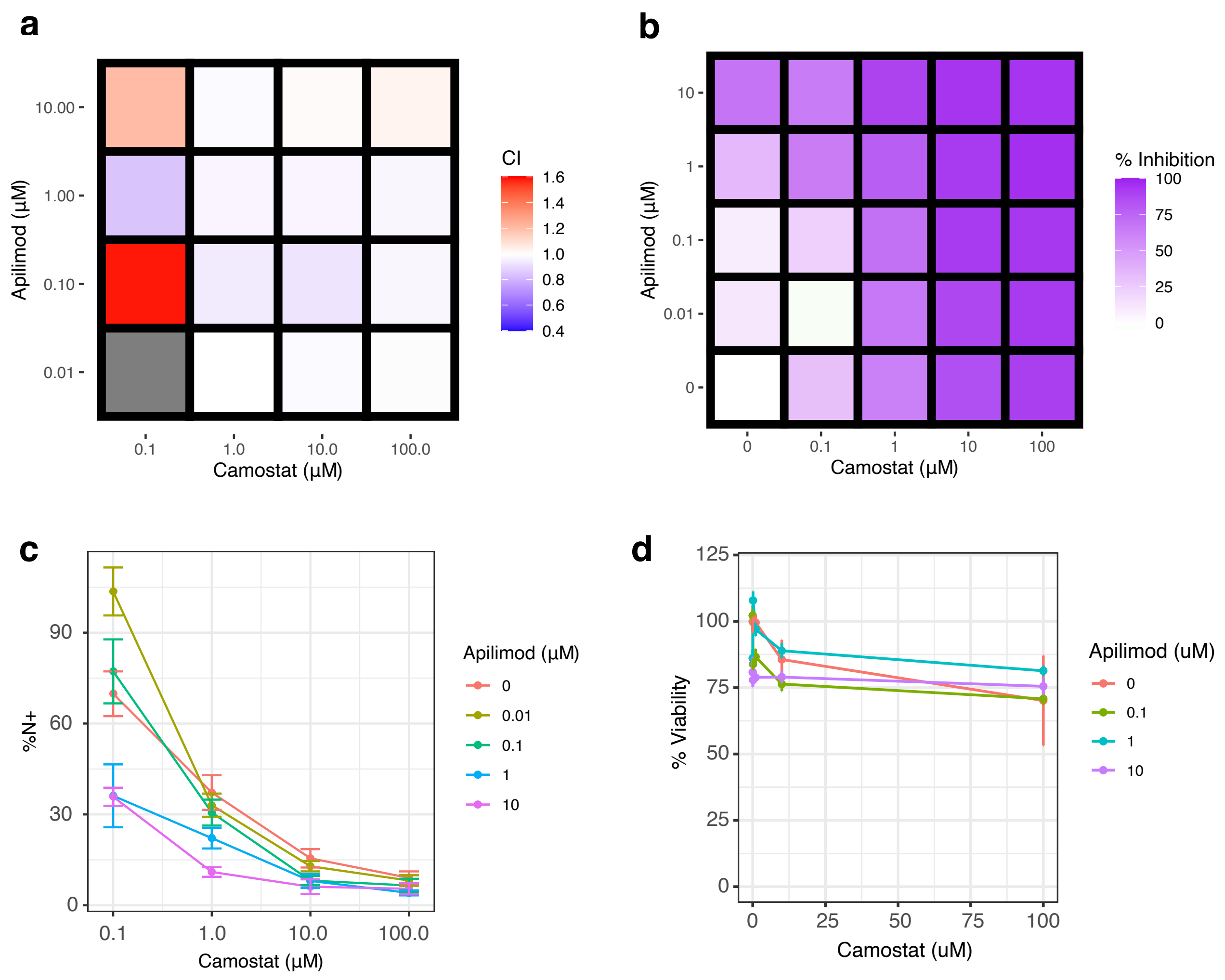

Supplementary Figure 6: Camostat and Apilimod inhibit replication of SARS-CoV-2 in Caco-2 cells. (**a**) Heatmap showing the Cooperativity Index (CI) between each dose of Apilimod and Camostat tested. The CI was calculated according to the Bliss independence model. (**b**) The percentage inhibition of cells expressing nucleocapsid protein achieved at each dose of Apilimod and Camostat tested. (**c**) Line graph showing the effect of dose of Apilimod against increasing dose of Camostat. Data show the mean and standard error, from three independent experiments. (**d**) Line graph showing the effect on cell viability, as assessed by MTT assay, of Apilimod against increasing dose of Camostat. Data show the mean and standard error of cell viability, from three independent experiments, normalized to the mean of the controls.

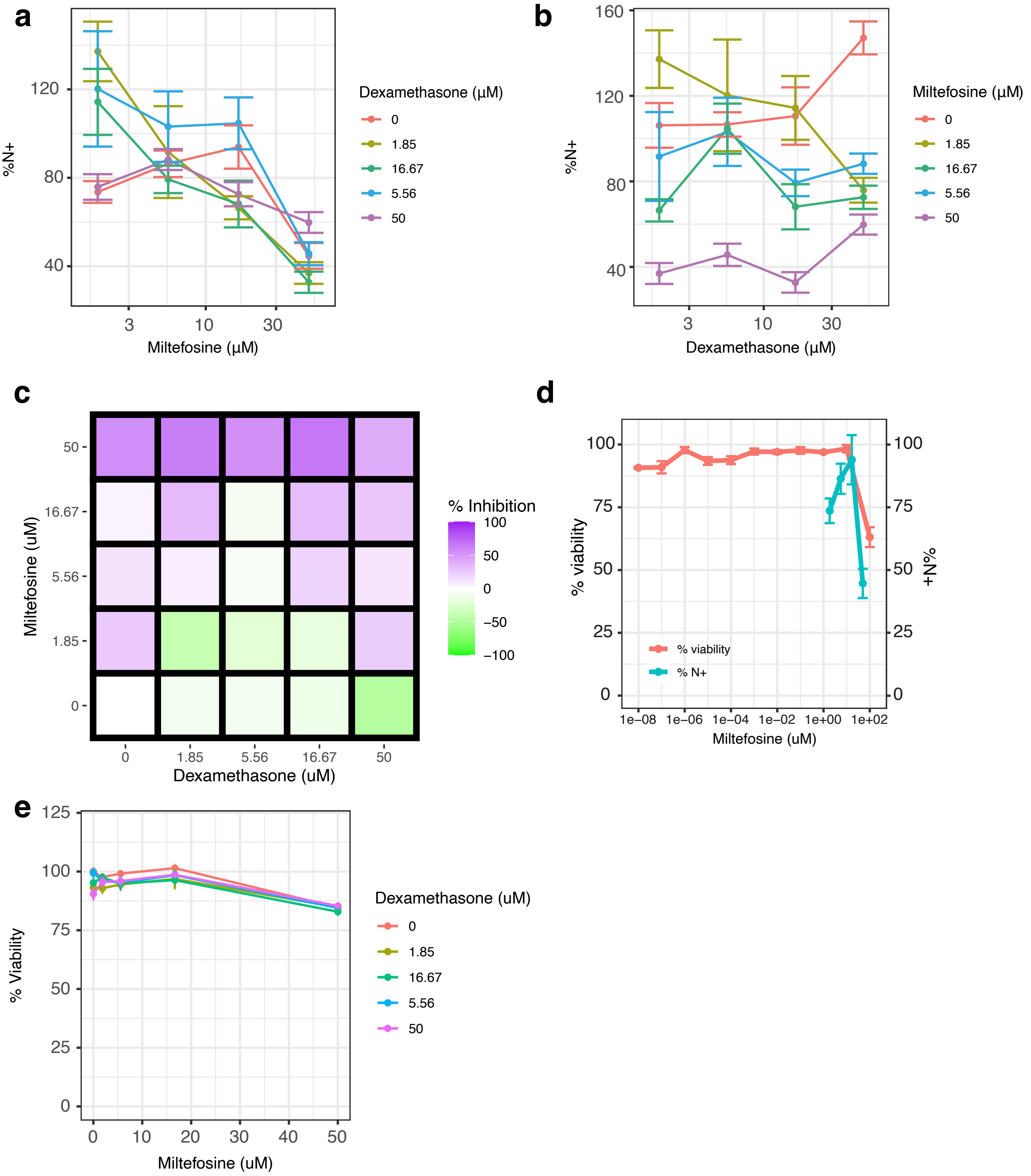

Supplementary Figure 7: The impact of Dexamethasone and Miltefosine on replication of SARS-CoV-2 in Caco-2 cells. (**a**) Line graph showing the effect of dose of Dexamethasone against increasing dose of Miltefosine. (**b**) Line graph showing the effect of dose of Miltefosine against increasing dose of Dexamethasone. (**c**) The percentage inhibition of cells expressing nucleocapsid protein achieved at each dose of Dexamethasone and Miltefosine tested. (**d**) Percentage viability as determined by MTT assay plotted alongside percentage of cells expressing nucleocapsid protein at varying doses of Miltefosine. Data show the mean and standard error, from three independent experiments. (**e**) Line graph showing the effect on cell viability, as assessed by MTT assay, of Miltefosine against increasing dose of Dexamethasone. Data show the mean and standard error of cell viability, from three independent experiments, normalized to the mean of the controls.

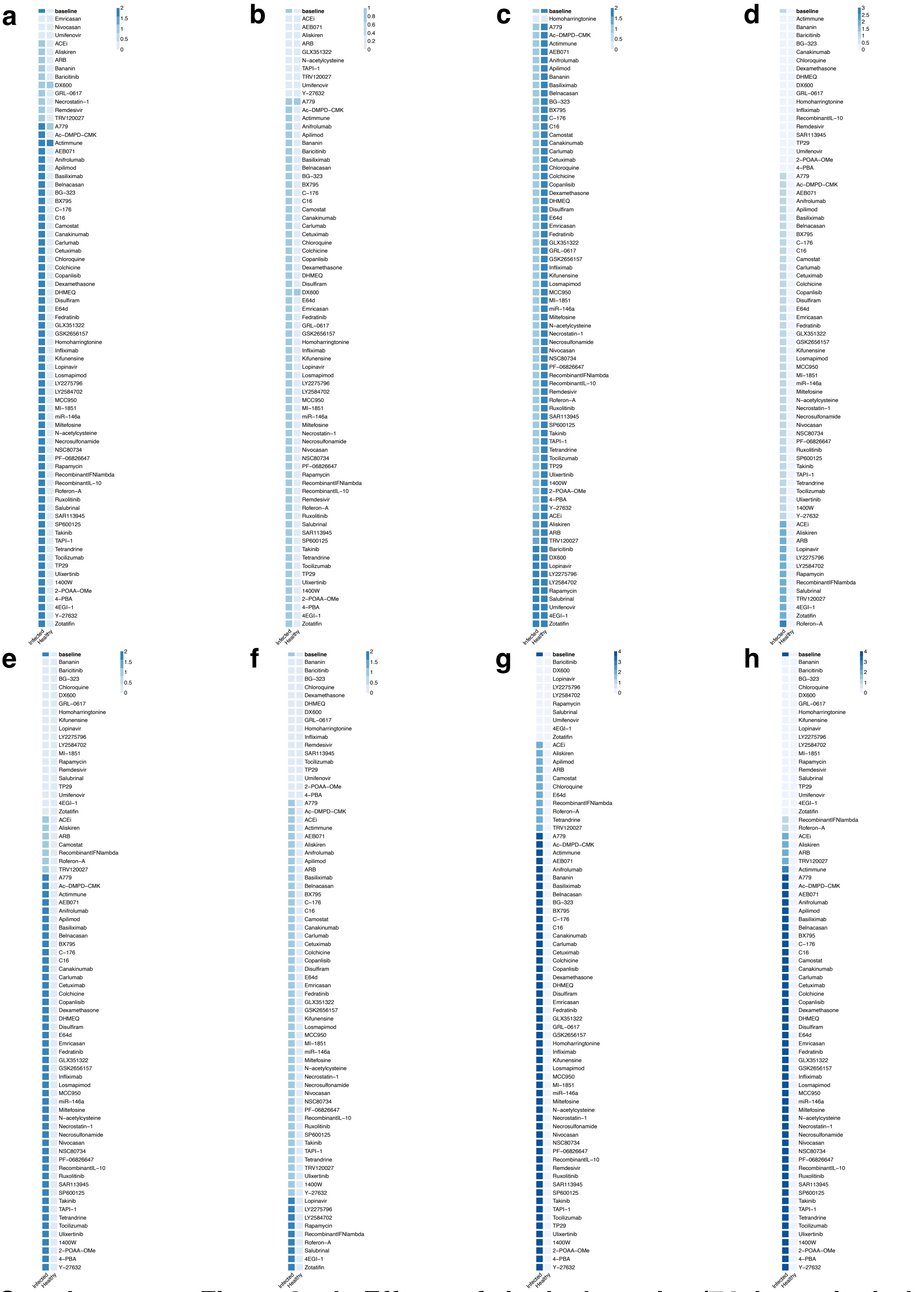

Supplementary Figure 8 a-h. Effects of single therapies (74 drugs, including those that have not passed phase II trials) in early stage, severe COVID-19. Columns represent infected and healthy conditions, rows represent drug tested including those at an experimental stage of development (Supplementary Table 5). Showing the effect on: (**a**) Cell Death. (**b**) Fibrosis. (**c**) Host Protein Synthesis. (**d**) Inflammation. (**e**) Syncytia Formation. (**f**) T-cell Infiltration. (**g**) Viral Entry. (**h**) Viral Replication.

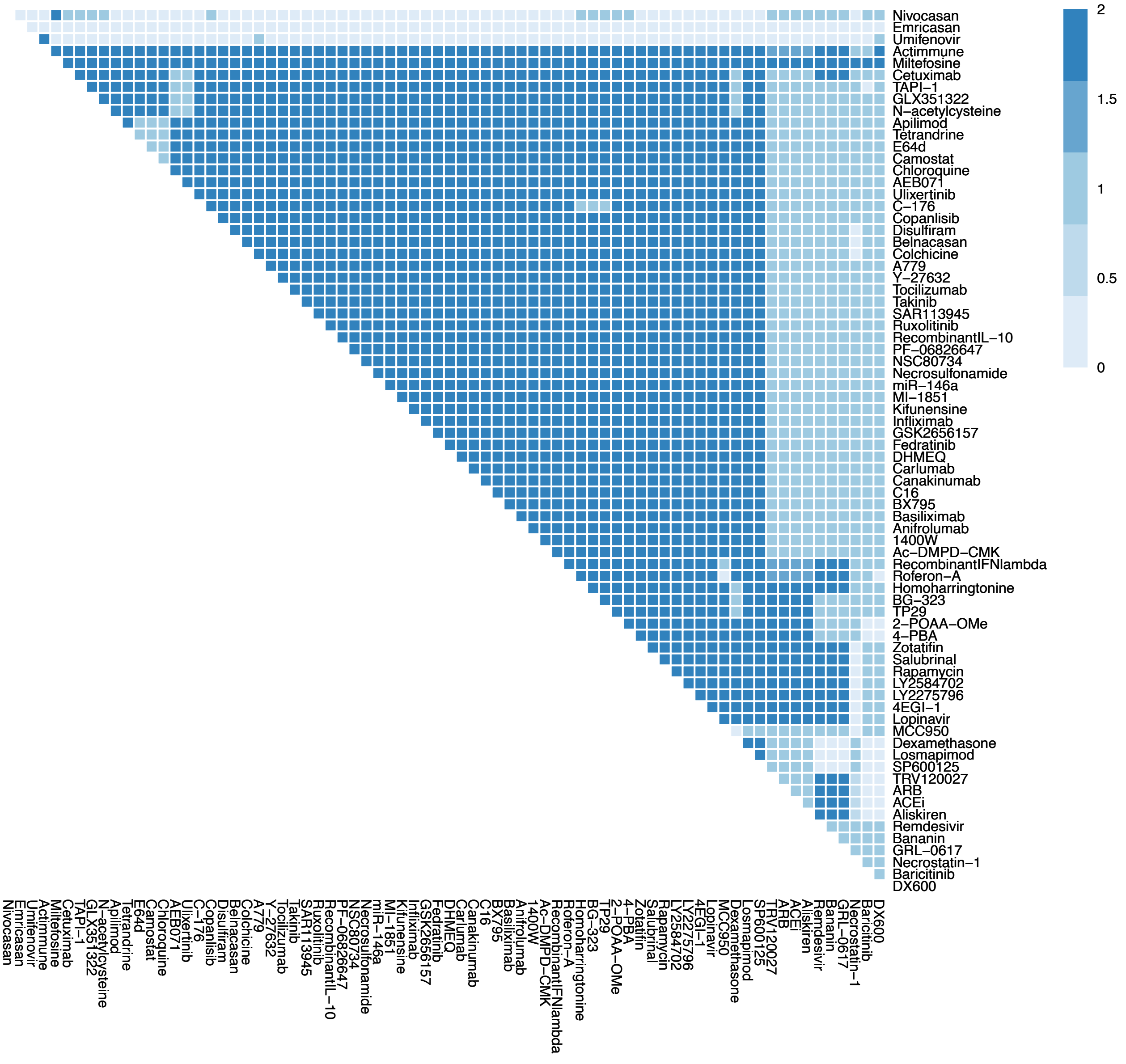

Supplementary Figure 8i Effects of combination therapy (74 drugs, including those that have not passed phase II trials) on Cell Death in early stage, severe COVID-19. The colour of the square corresponds to the level of the *CellDeath* node at steady state under treatment by the drugs indicated on the x- and y-axis. This plot shows all drugs, including those at an experimental stage of development.

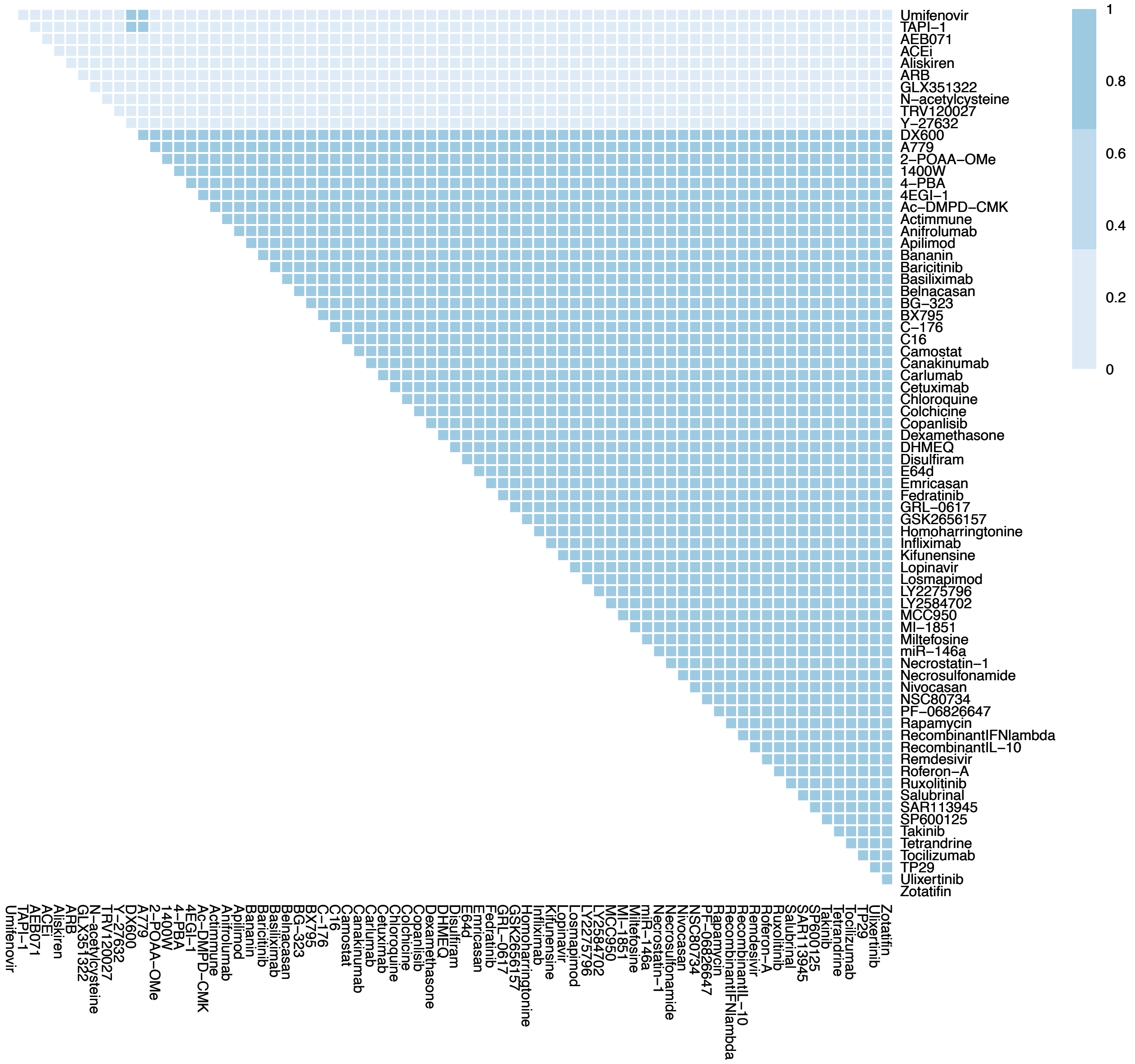

Supplementary Figure 8j. Effects of combination therapy (74 drugs, including those that have not passed phase II trials) on Fibrosis in early stage, severe COVID-19. The colour of the square corresponds to the level of the *Firbrosis* node at steady state under treatment by the drugs indicated on the x- and y-axis. This plot shows all drugs, including those at an experimental stage of development.

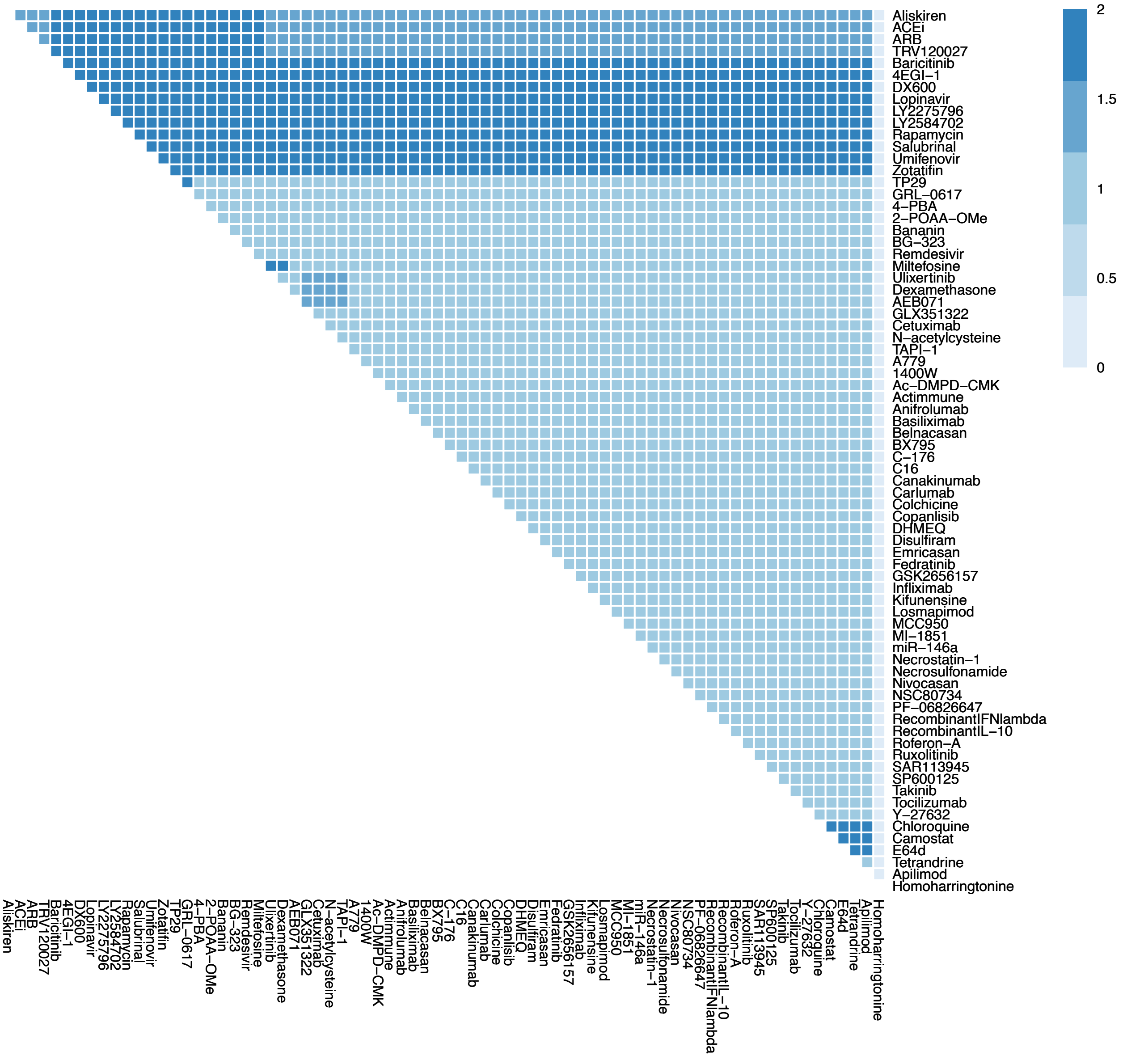

Supplementary Figure 8k. Effects of combination therapy (74 drugs, including those that have not passed phase II trials) on Host Protein Synthesis in early stage, severe COVID-19. The colour of the square corresponds to the level of the *HostProteinSynthesis* node at steady state under treatment by the drugs indicated on the x- and y-axis. This plot shows all drugs, including those at an experimental stage of development.

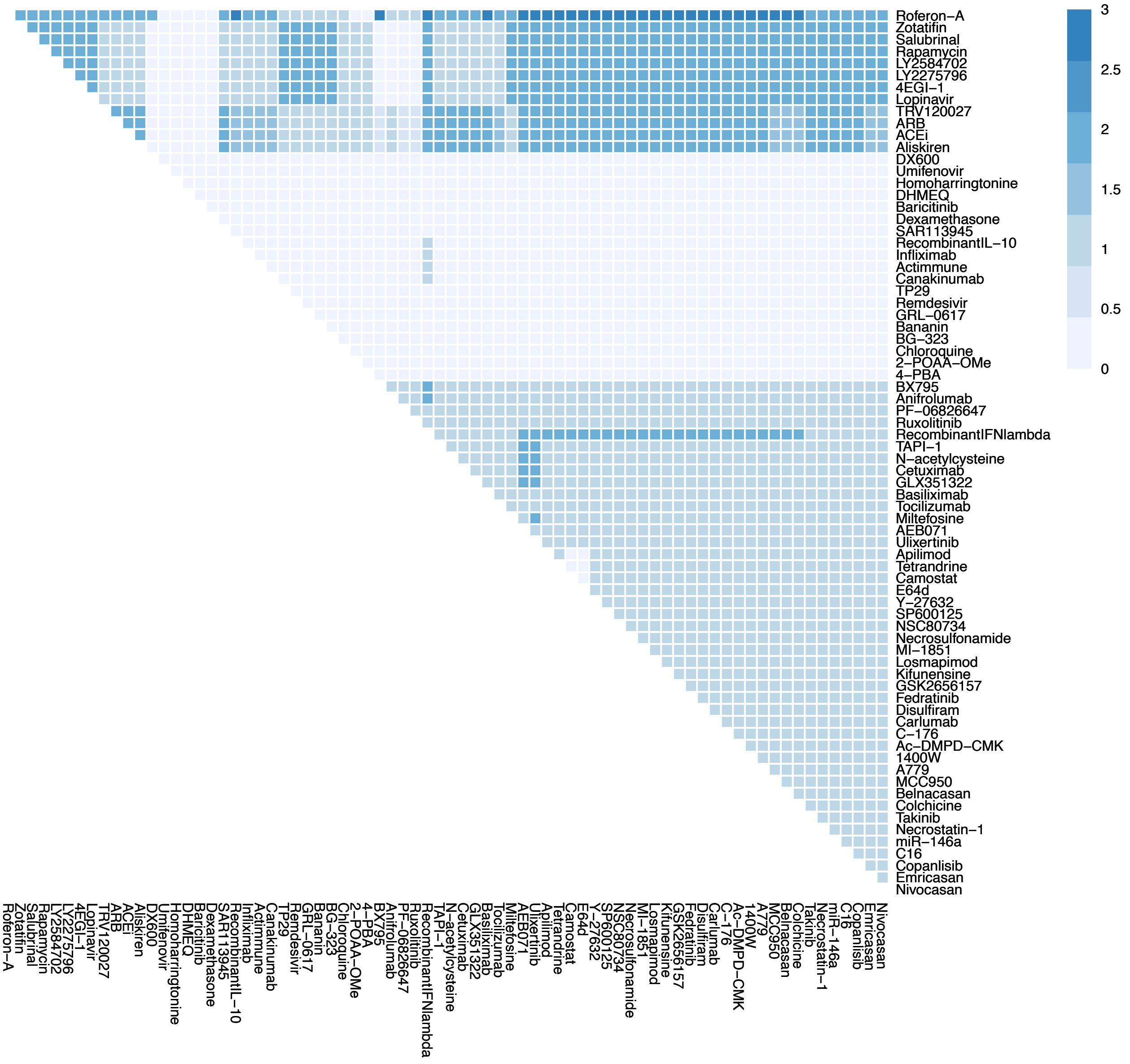

Supplementary Figure 8I. Effects of combination therapy (74 drugs, including those that have not passed phase II trials) on Inflammation in early stage, severe COVID-19. The colour of the square corresponds to the level of the *Inflammation* node at steady state under treatment by the drugs indicated on the x- and y-axis. This plot shows all drugs, including those at an experimental stage of development.

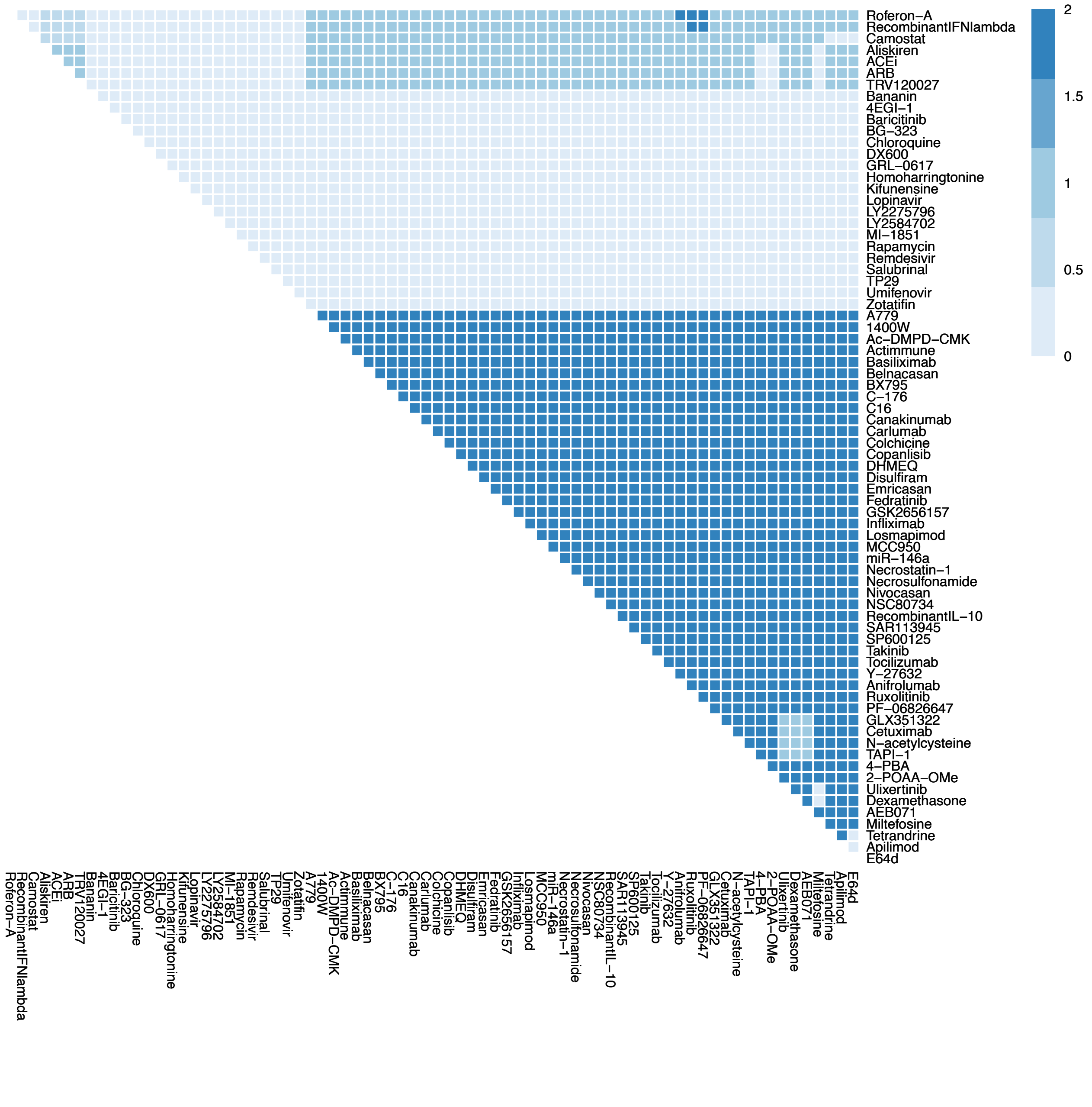

Supplementary Figure 8m. Effects of combination therapy (74 drugs, including those that have not passed phase II trials) on Syncytia Formation in early stage, severe COVID-19. The colour of the square corresponds to the level of the *SyncytiaFormation* node at steady state under treatment by the drugs indicated on the x- and y-axis. This plot shows all drugs, including those at an experimental stage of development.

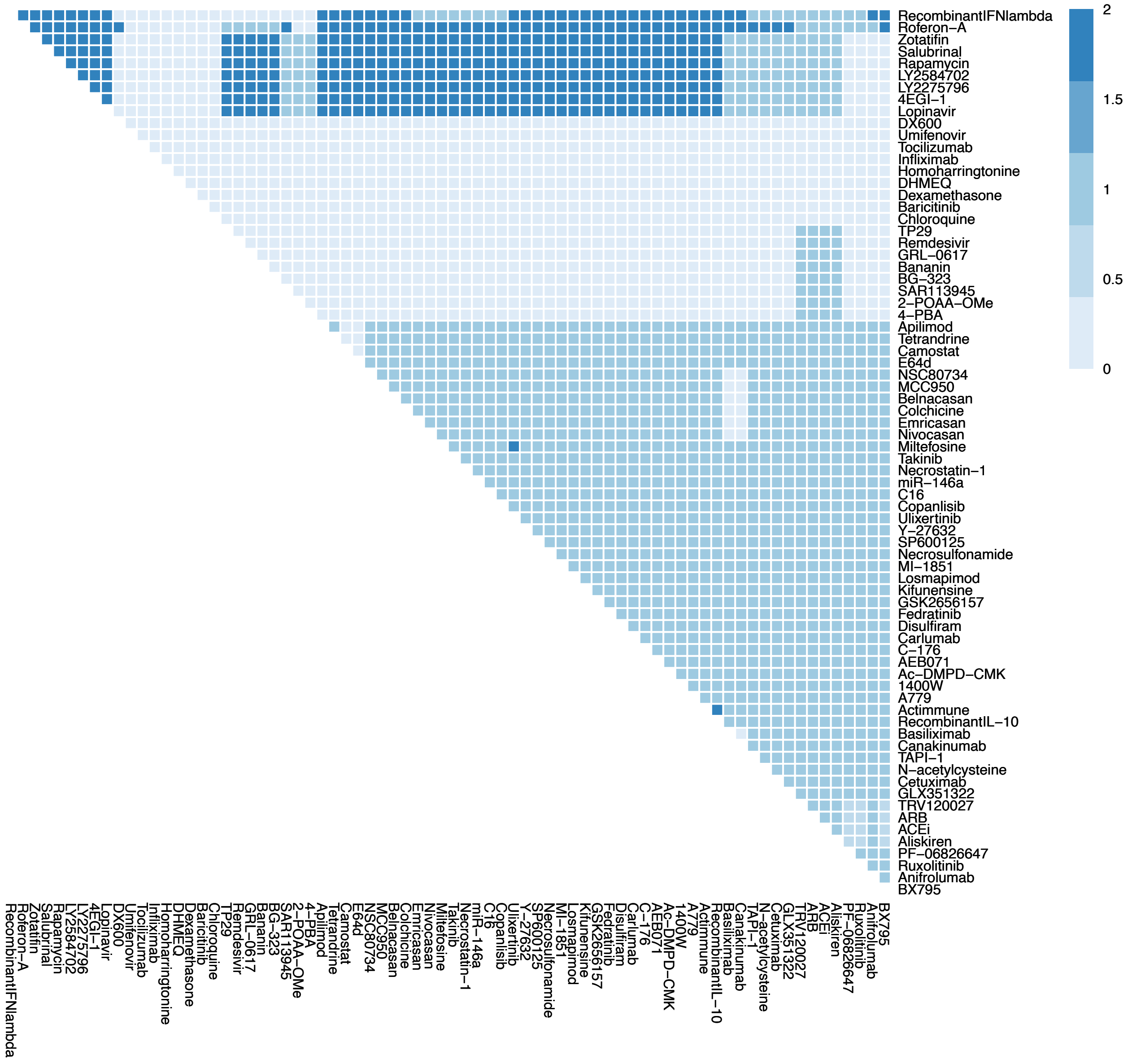

Supplementary Figure 8n. Effects of combination therapy (74 drugs, including those that have not passed phase II trials) on T-cell Infiltration in early stage, severe COVID-19. The colour of the square corresponds to the level of the *T-cellInfiltration* node at steady state under treatment by the drugs indicated on the x- and y-axis. This plot shows all drugs, including those at an experimental stage of development.

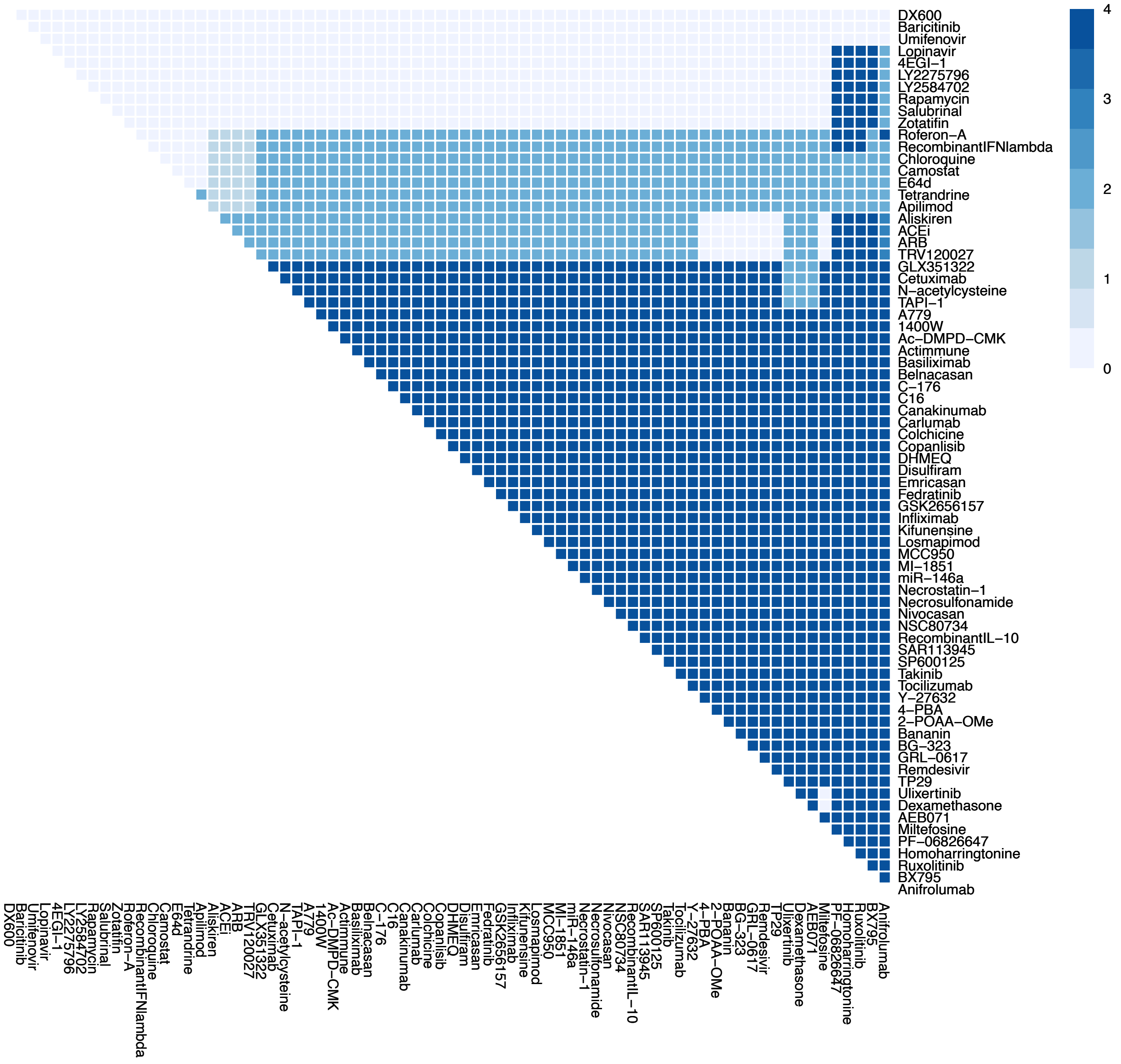

Supplementary Figure 8o. Effects of combination therapy (74 drugs, including those that have not passed phase II trials) on Viral Entry in early stage, severe COVID-19. The colour of the square corresponds to the level of the *ViralEntry* node at steady state under treatment by the drugs indicated on the x- and y-axis. This plot shows all drugs, including those at an experimental stage of development.

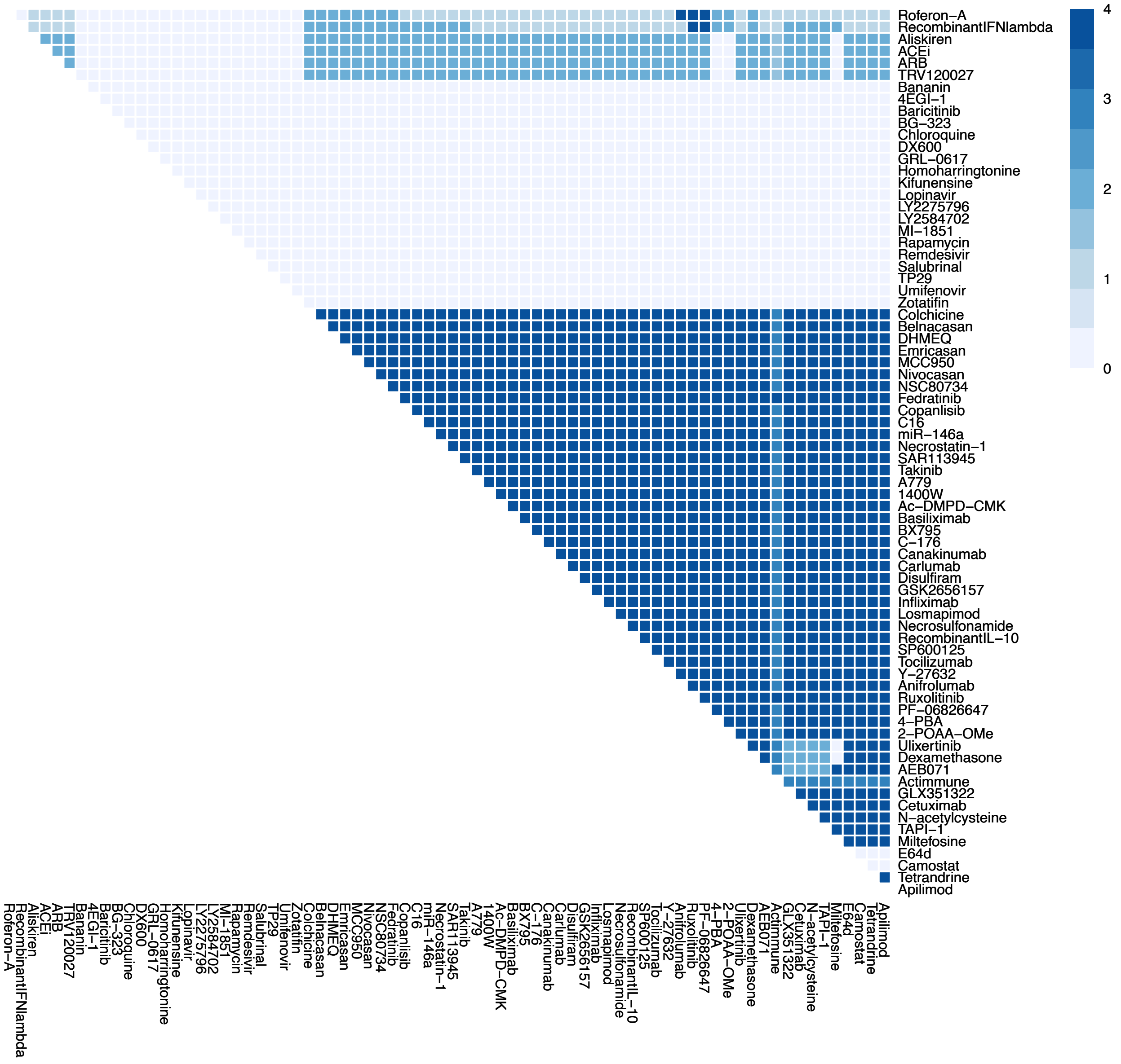

Supplementary Figure 8p. Effects of combination therapy (74 drugs, including those that have not passed phase II trials) on Viral Replication in early stage, severe COVID-19. The colour of the square corresponds to the level of the *ViralReplication* node at steady state under treatment by the drugs indicated on the x- and y-axis. This plot shows all drugs, including those at an experimental stage of development.

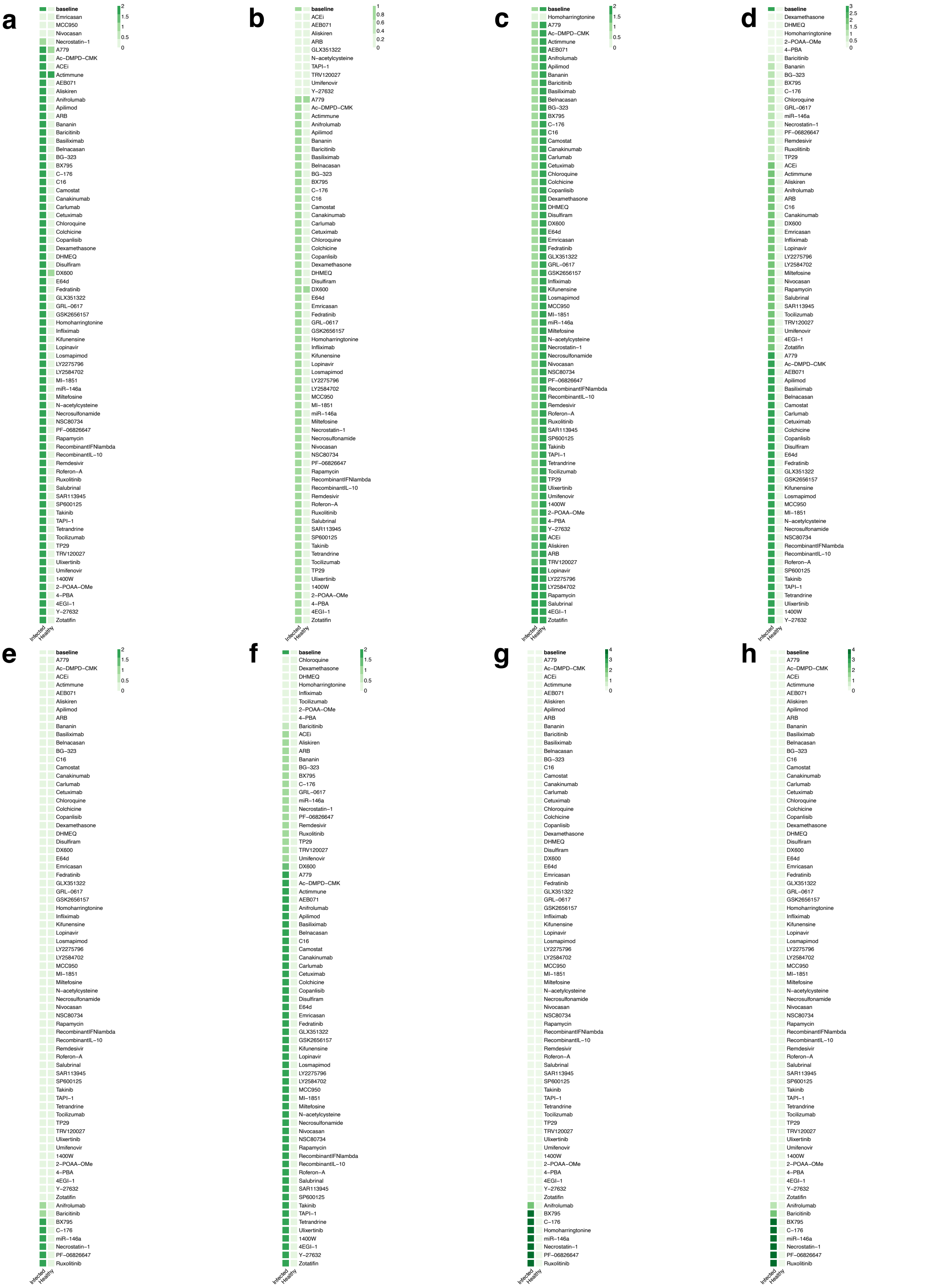

Supplementary Figure 9 a-h. Effects of single therapies (74 drugs, including those that have not passed phase II trials) in early stage, severe COVID-19. Columns represent infected and healthy conditions, rows represent drug tested including those at an experimental stage of development (Supplementary Table 5). Showing the effect on: (**a**) Cell Death. (**b**) Fibrosis. (**c**) Host Protein Synthesis. (**d**) Inflammation. (**e**) Syncytia Formation. (**f**) T-cell Infiltration. (**g**) Viral Entry. (**h**) Viral Replication.

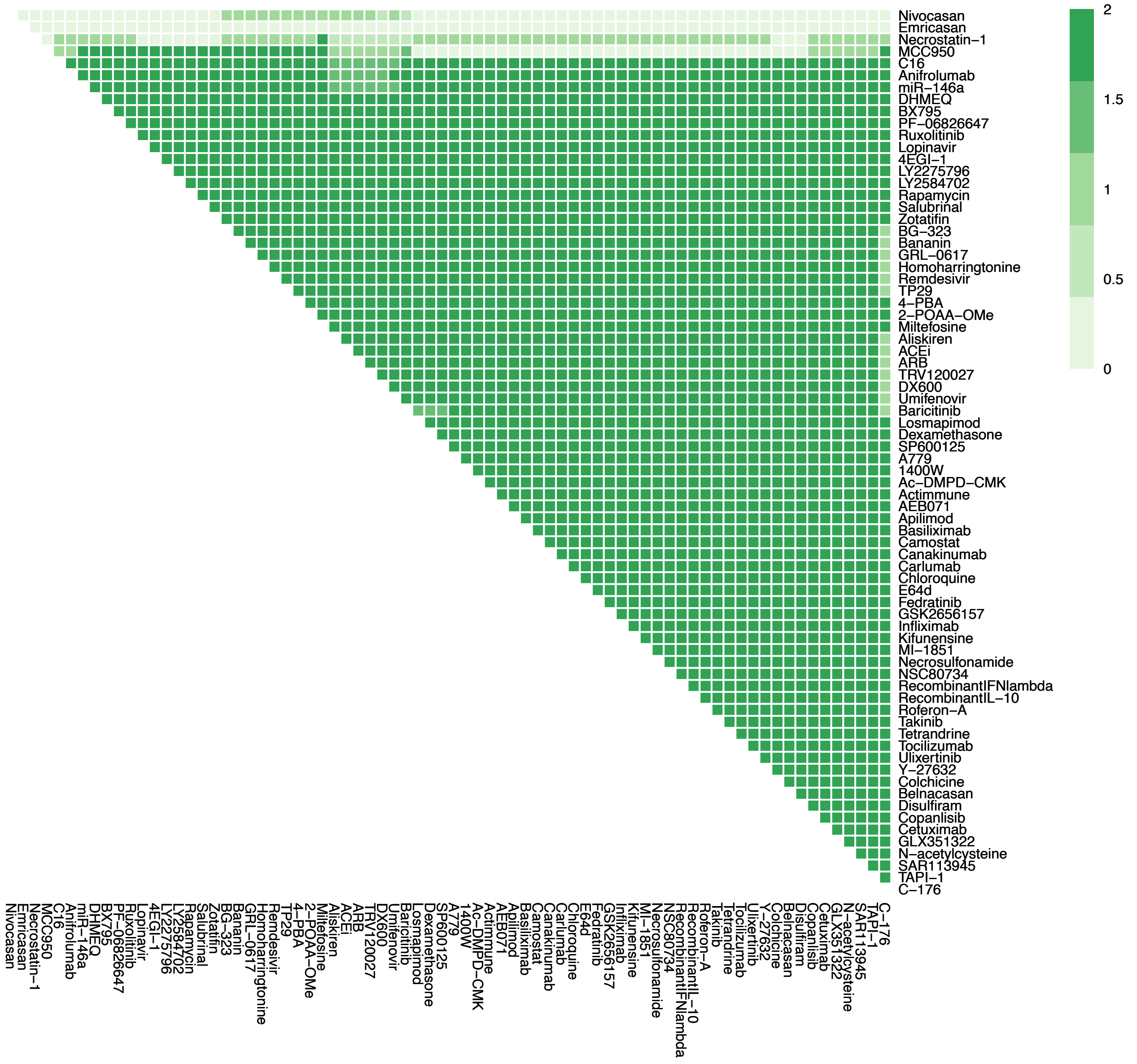

Supplementary Figure 9i. Effects of combination therapy (74 drugs, including those that have not passed phase II trials) on Cell Death in late stage, severe COVID-19. The colour of the square corresponds to the level of the *CellDeath* node at steady state under treatment by the drugs indicated on the x- and y-axis. This plot shows all drugs, including those at an experimental stage of development.

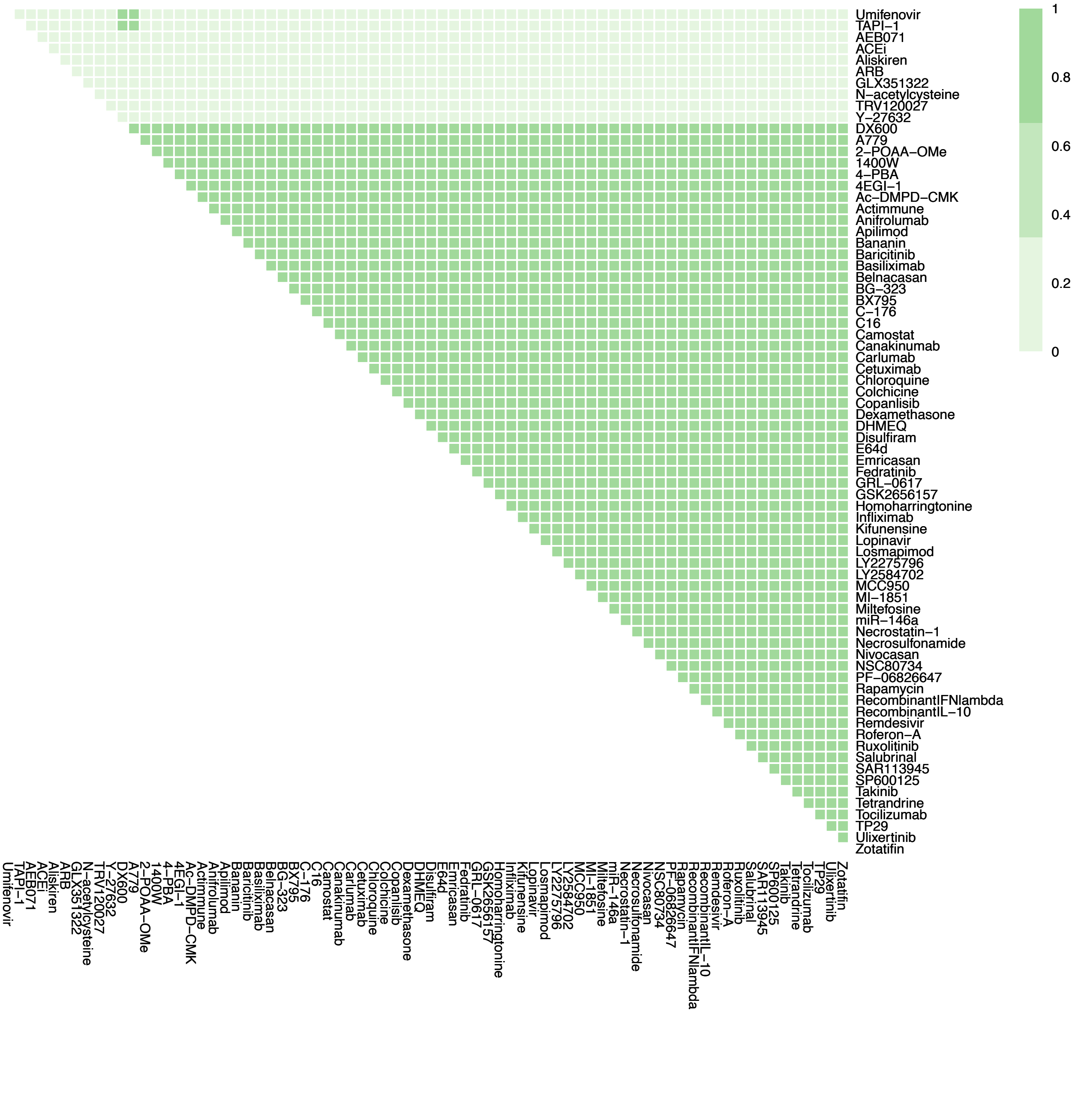

Supplementary Figure 9j. Effects of combination therapy (74 drugs, including those that have not passed phase II trials) on Fibrosis in late stage, severe COVID-19. The colour of the square corresponds to the level of the *Fibrosis* node at steady state under treatment by the drugs indicated on the x- and y-axis. This plot shows all drugs, including those at an experimental stage of development.

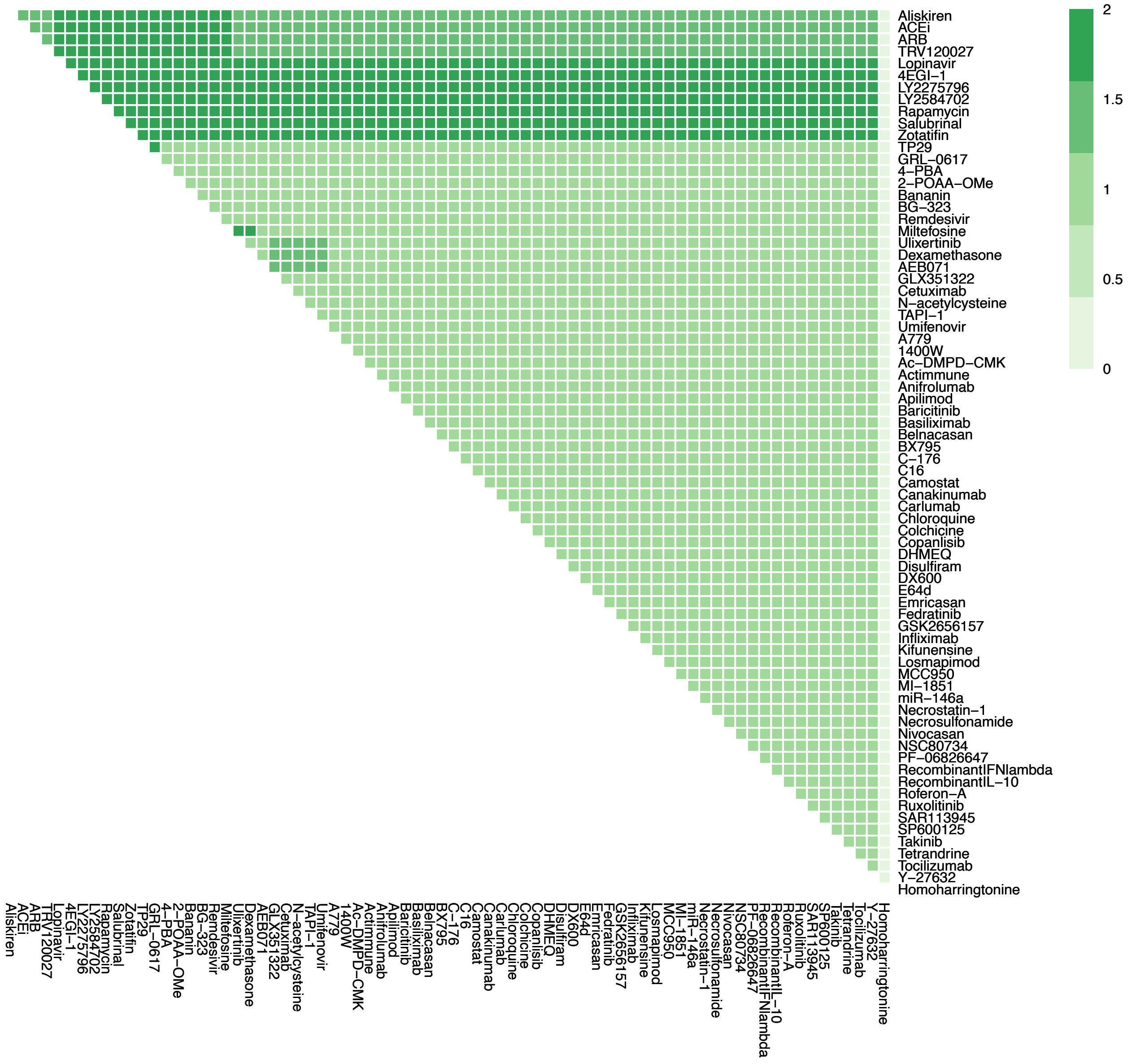

Supplementary Figure 9k. Effects of combination therapy (74 drugs, including those that have not passed phase II trials) on Host Protein Synthesis in late stage, severe COVID-19. The colour of the square corresponds to the level of the *HostProteinSynthesis* node at steady state under treatment by the drugs indicated on the x- and y-axis. This plot shows all drugs, including those at an experimental stage of development.

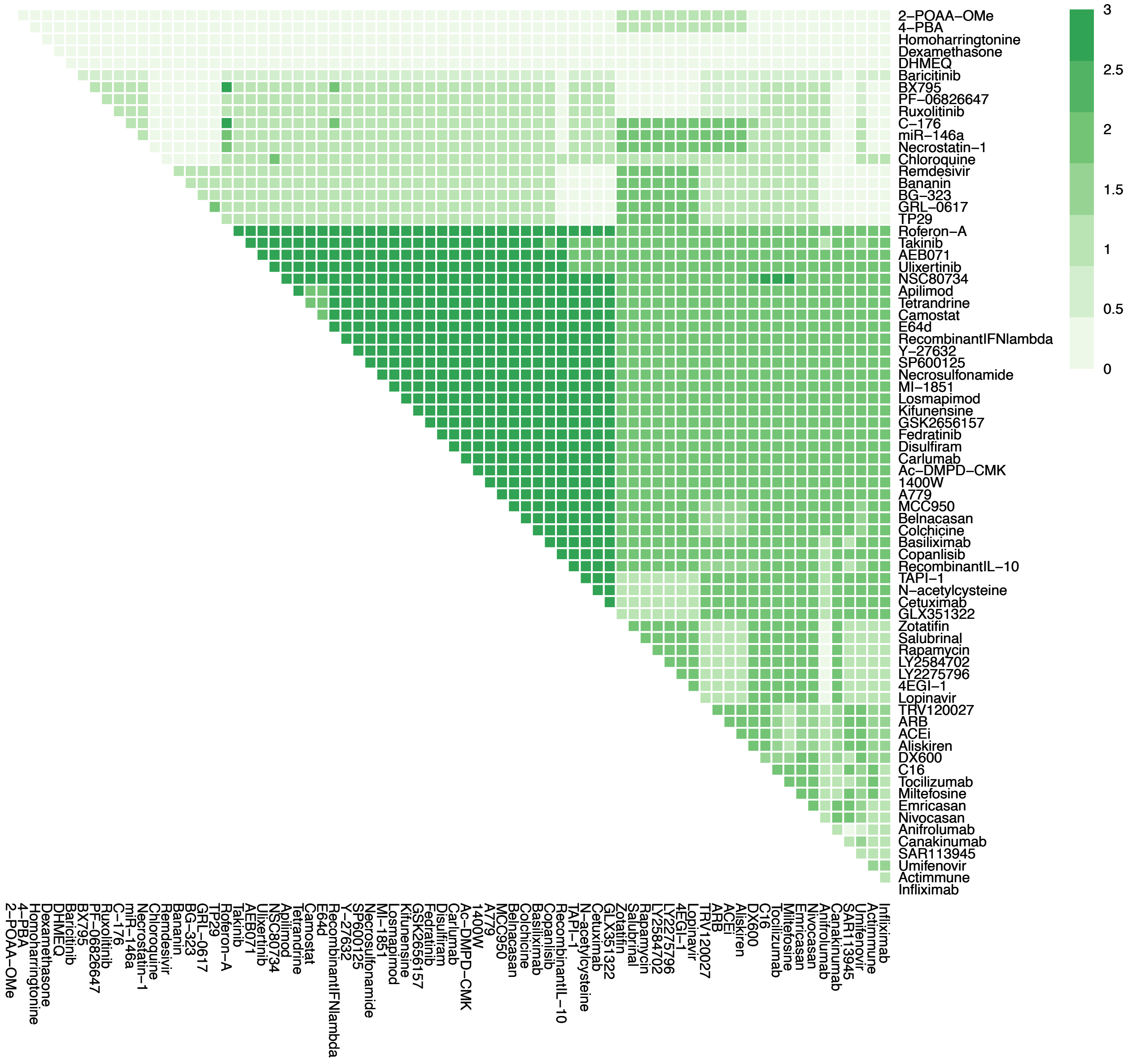

Supplementary Figure 9l. Effects of combination therapy (74 drugs, including those that have not passed phase II trials) on Inflammation in late stage, severe COVID-19. The colour of the square corresponds to the level of the *Inflammation* node at steady state under treatment by the drugs indicated on the x- and y-axis. This plot shows all drugs, including those at an experimental stage of development.

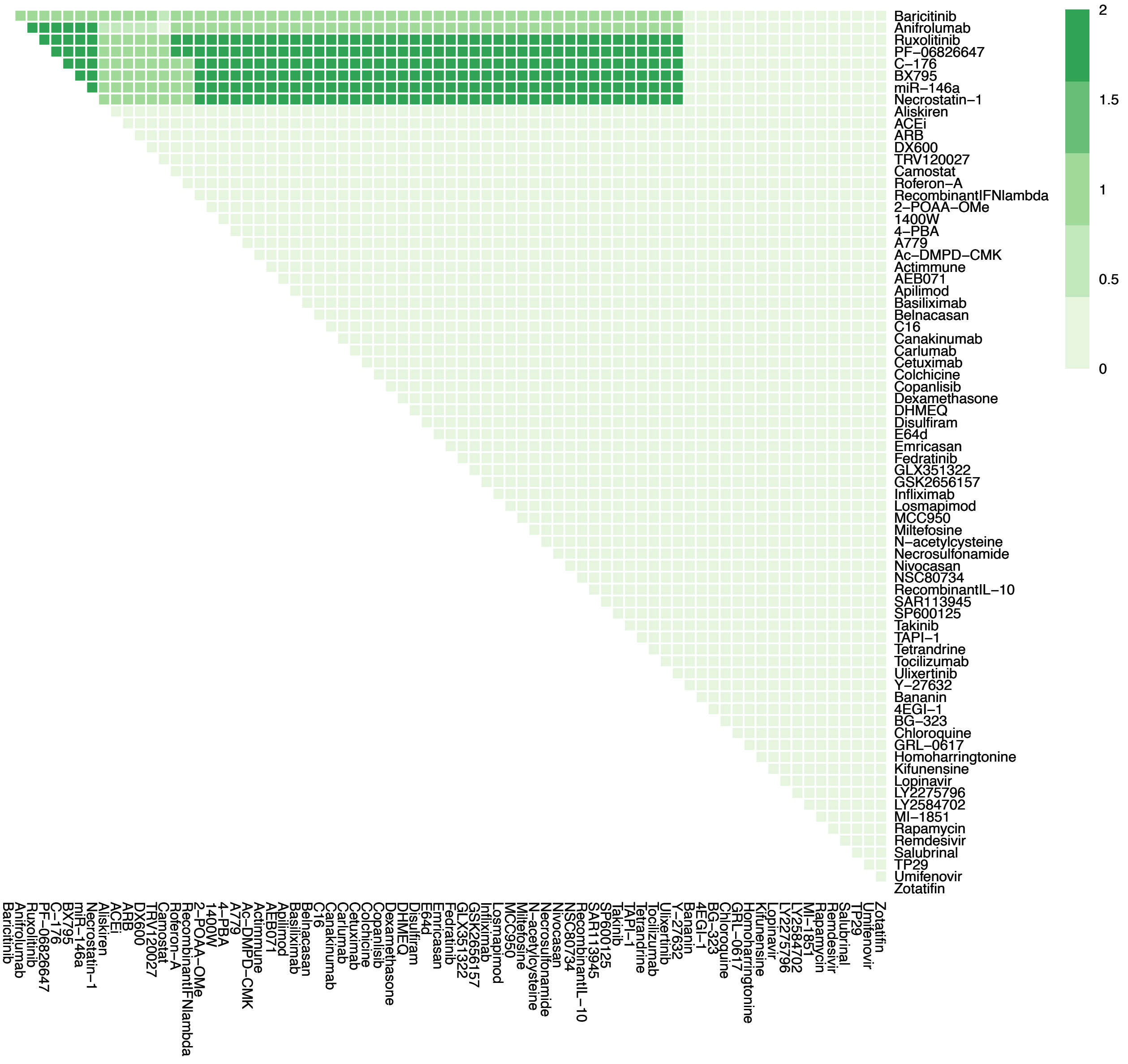

Supplementary Figure 9m. Effects of combination therapy (74 drugs, including those that have not passed phase II trials) on Syncytia Formation in late stage, severe COVID-19. The colour of the square corresponds to the level of the *SyncytiaFormation* node at steady state under treatment by the drugs indicated on the x- and y-axis. This plot shows all drugs, including those at an experimental stage of development.

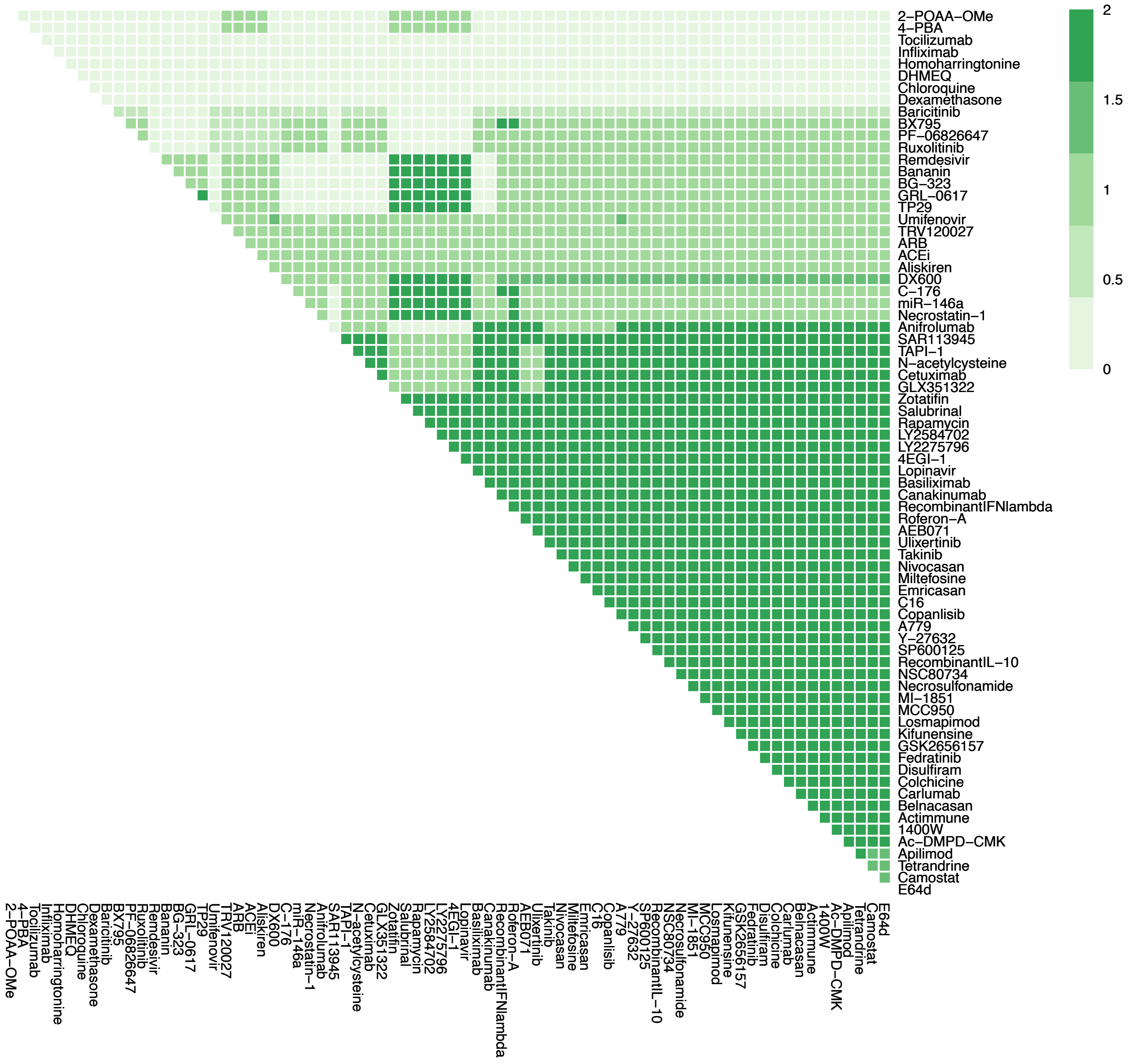

Supplementary Figure 9n. Effects of combination therapy (74 drugs, including those that have not passed phase II trials) on T-cell Infiltration in late stage, severe COVID-19. The colour of the square corresponds to the level of the *T-cellInfiltration* node at steady state under treatment by the drugs indicated on the x- and y-axis. This plot shows all drugs, including those at an experimental stage of development.

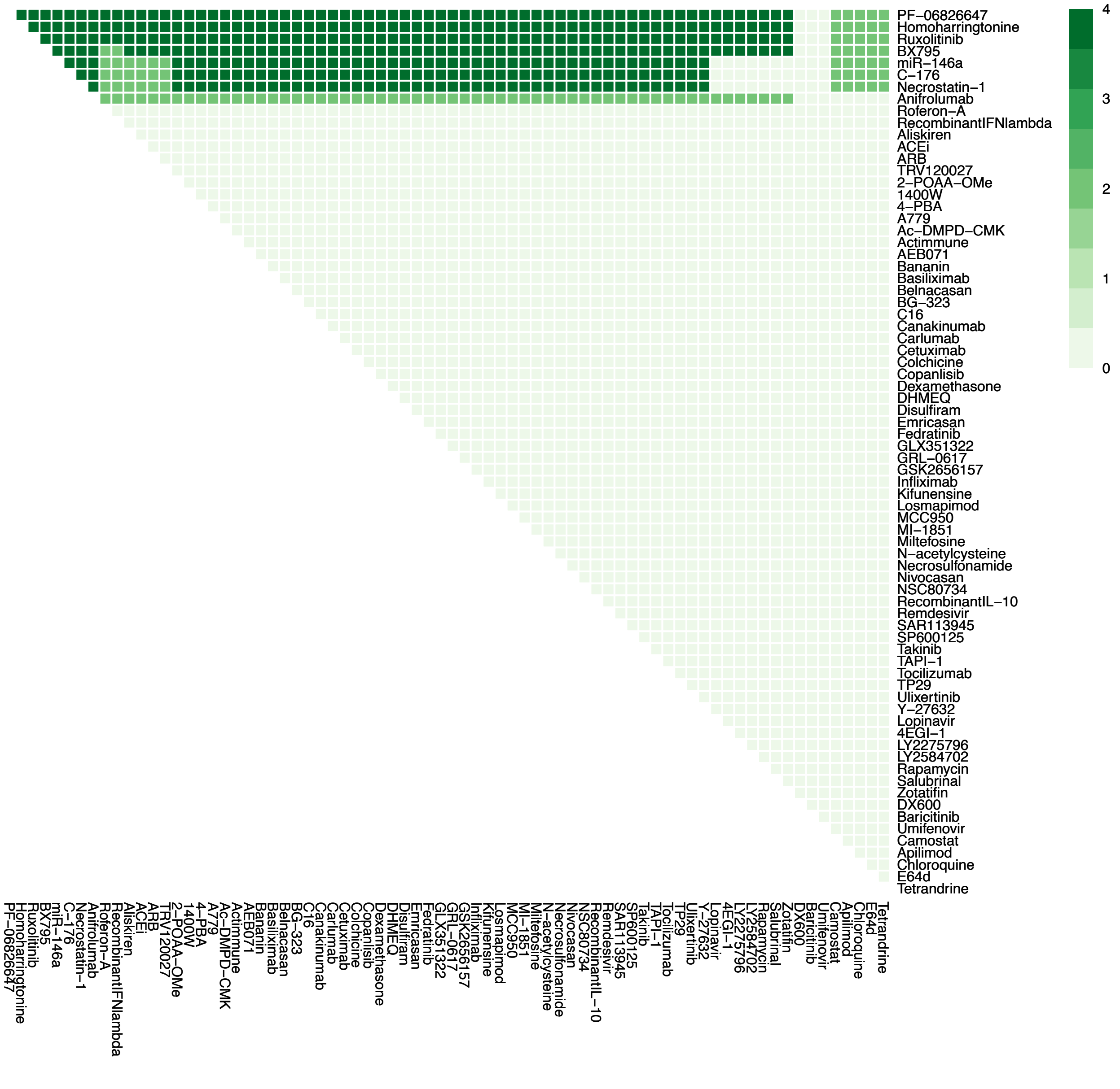

Supplementary Figure 9o. Effects of combination therapy (74 drugs, including those that have not passed phase II trials) on Viral Entry in late stage, severe COVID-19. The colour of the square corresponds to the level of the *ViralEntry* node at steady state under treatment by the drugs indicated on the x- and y-axis. This plot shows all drugs, including those at an experimental stage of development.

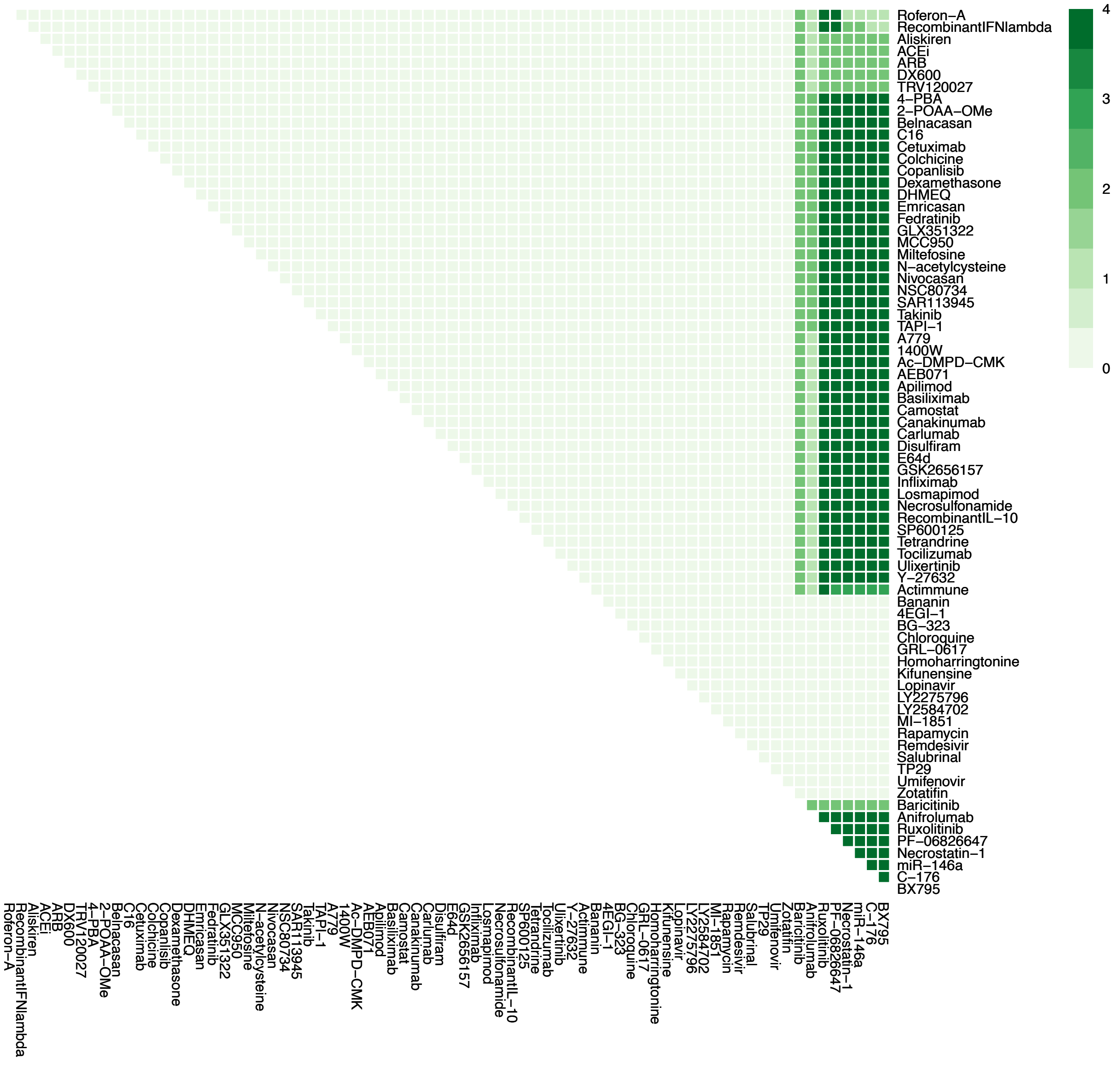

Supplementary Figure 9p. Effects of combination therapy (74 drugs, including those that have not passed Supplementary phase II trials) on Viral Replication in late stage, severe COVID-19. The colour of the square corresponds to the level of the *ViralReplication* node at steady state under treatment by the drugs indicated on the x- and y-axis. This plot shows all drugs, including those at an experimental stage of development.

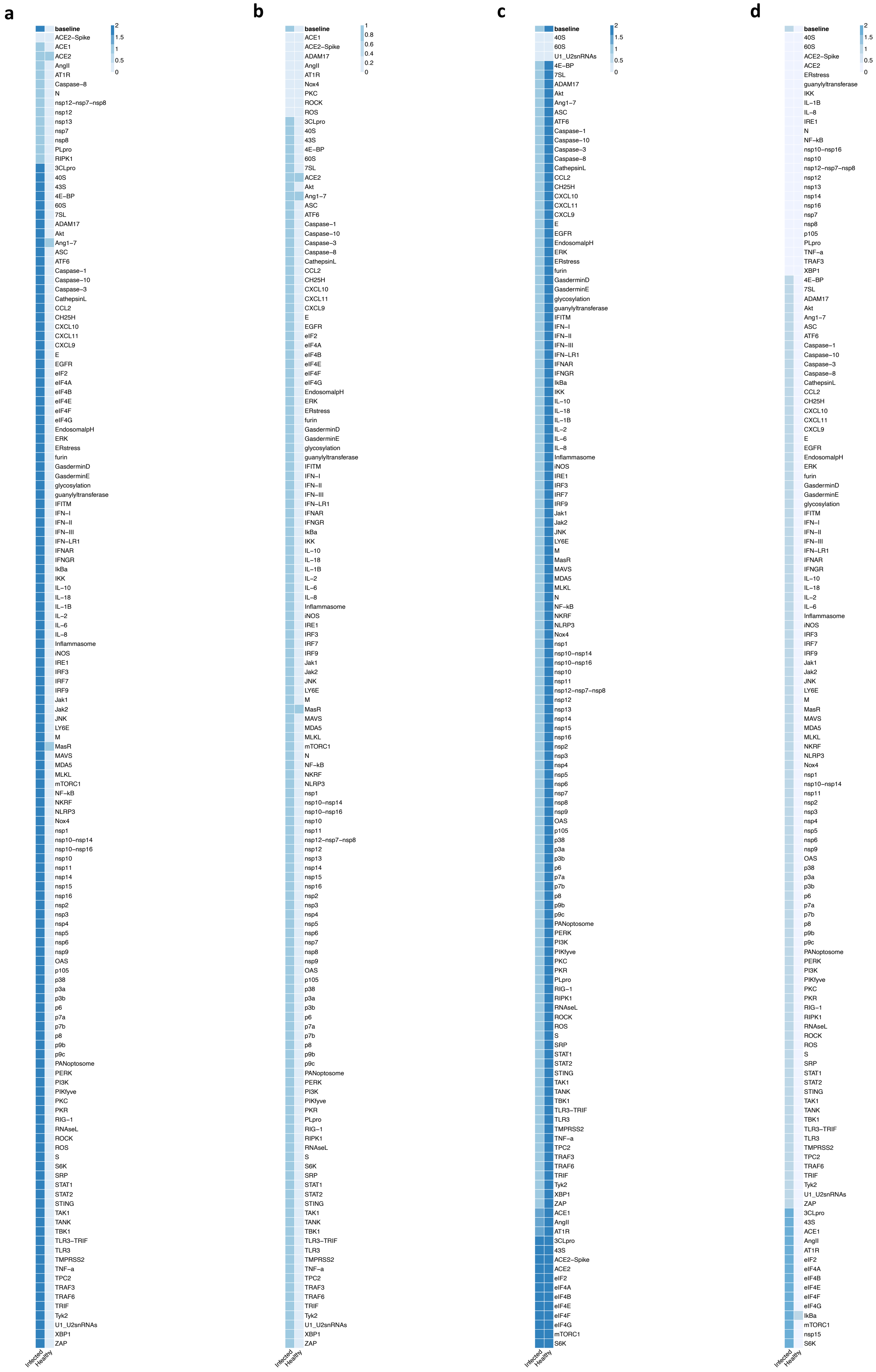

Supplementary Figure 10 a-d. Effects of single therapies (all nodes) in early stage, severe COVID-19. Columns represent infected and healthy conditions, rows represent node inhibited. Showing the effect on: (**a**) Cell Death. (**b**) Fibrosis. (**c**) Host Protein Synthesis. (**d**) Inflammation.

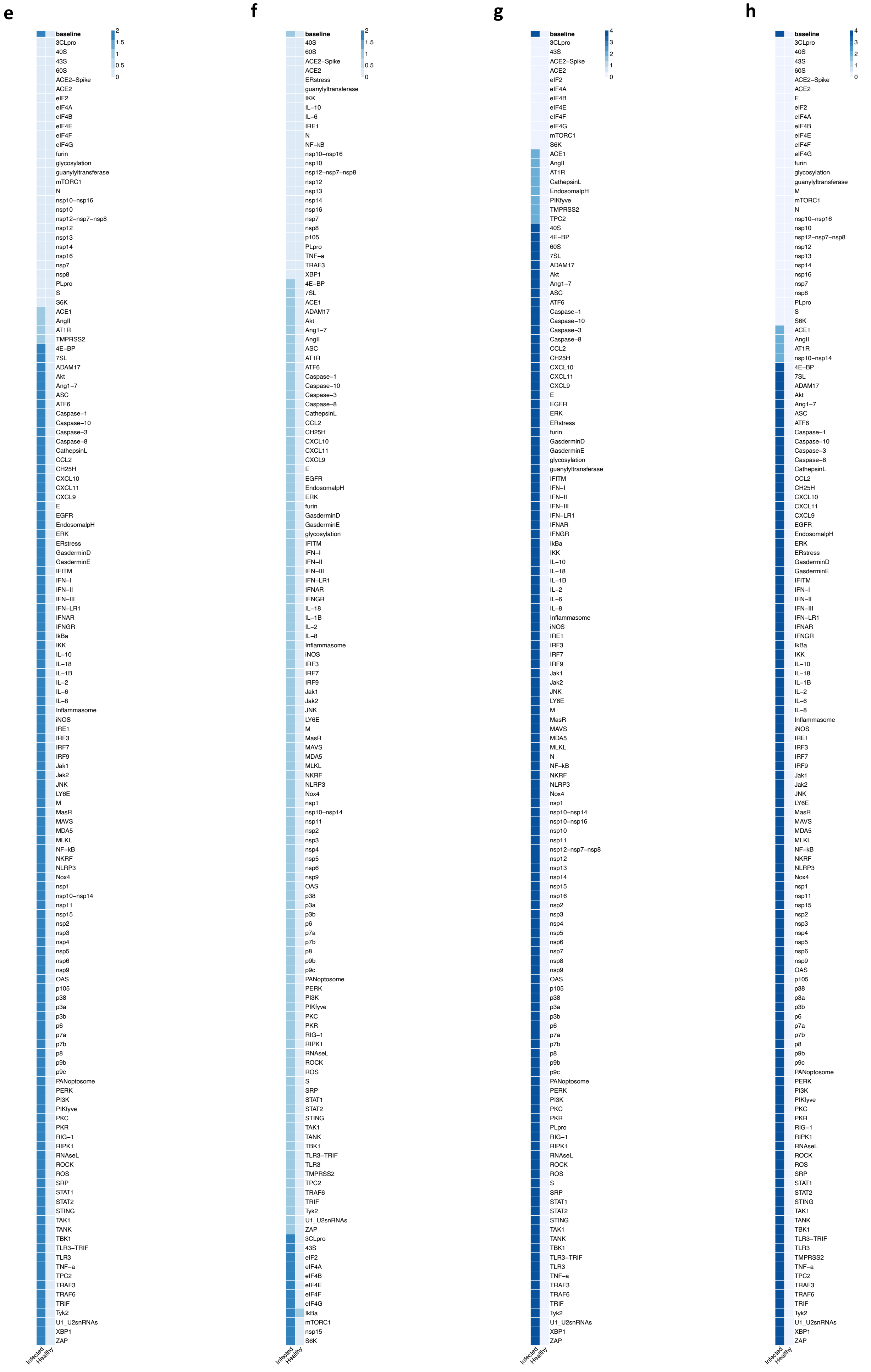

Supplementary Figure 10 e-h. Effects of single therapies (all nodes) in early stage, severe COVID-19. Columns represent infected and healthy conditions, rows represent node inhibited. Showing the effect on: (**e**) Syncytia Formation. (**f**) T-cell Infiltration. (**g**) Viral Entry. (**h**) Viral Replication.

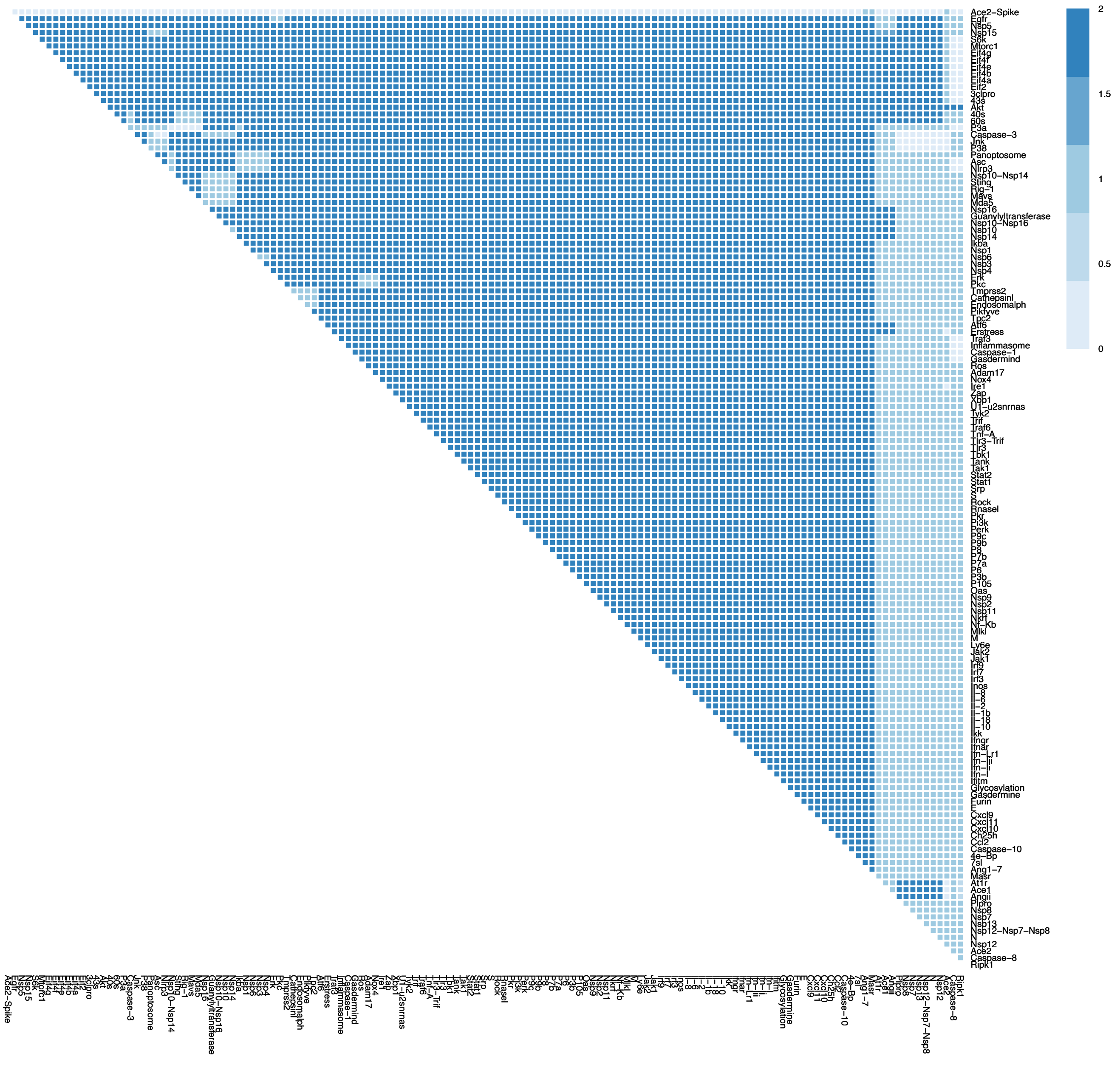

Supplementary Figure 10i. Effects of combination therapy (all nodes) on Cell Death in early stage, severe COVID-19. The colour of the square corresponds to the level of the *CellDeath* node at steady state upon inhbition of the nodes indicated on the x- and y-axis. This plot shows inhibition of all nodes representing druggable targets (proteins and complexes).

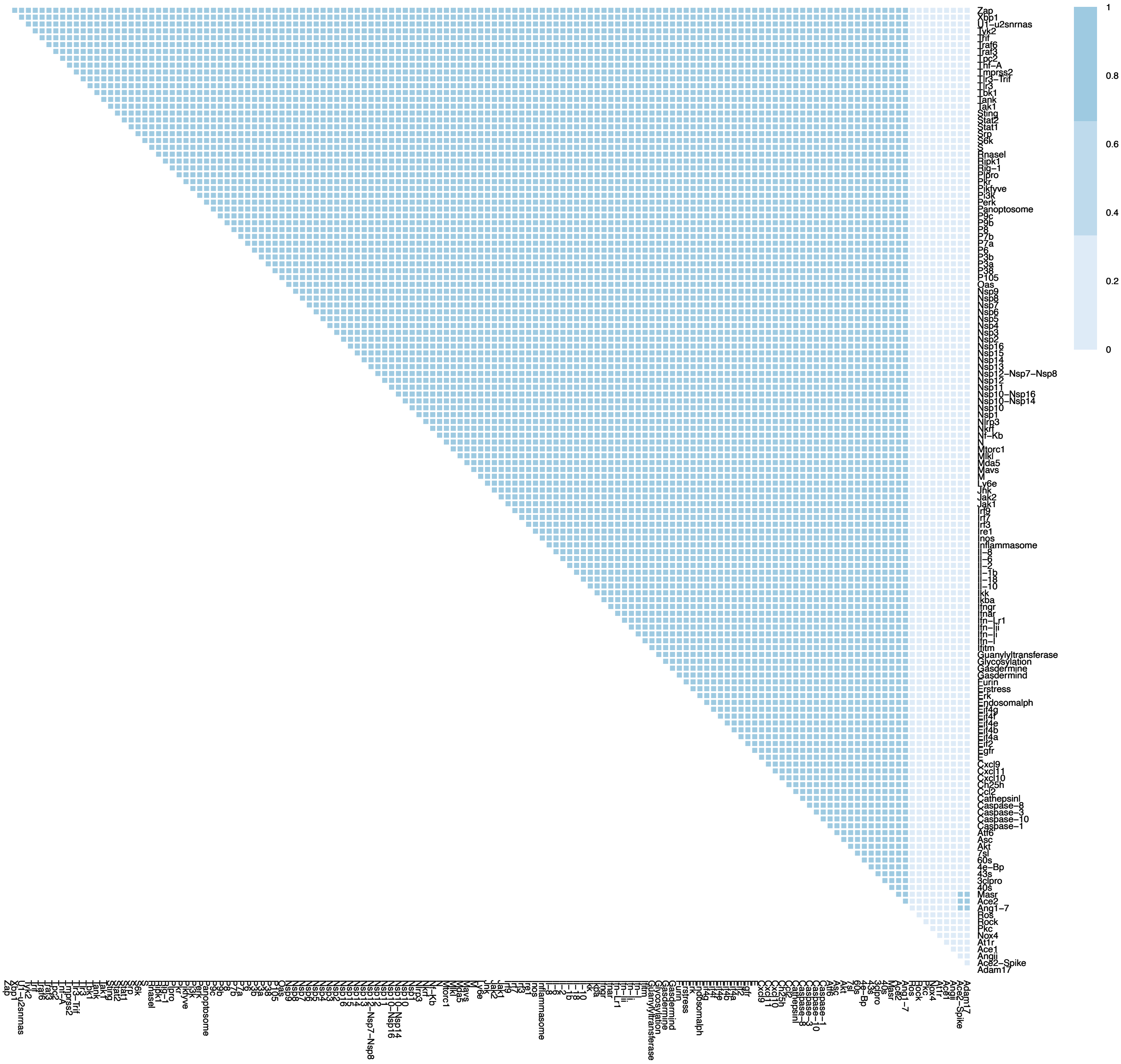

Supplementary Figure 10j. Effects of combination therapy (all nodes) on Fibrosis in early stage, severe COVID-19. The colour of the square corresponds to the level of the *Fibrosis* node at steady state upon inhbition of the nodes indicated on the x- and y-axis. This plot shows inhibition of all nodes representing druggable targets (proteins and complexes).

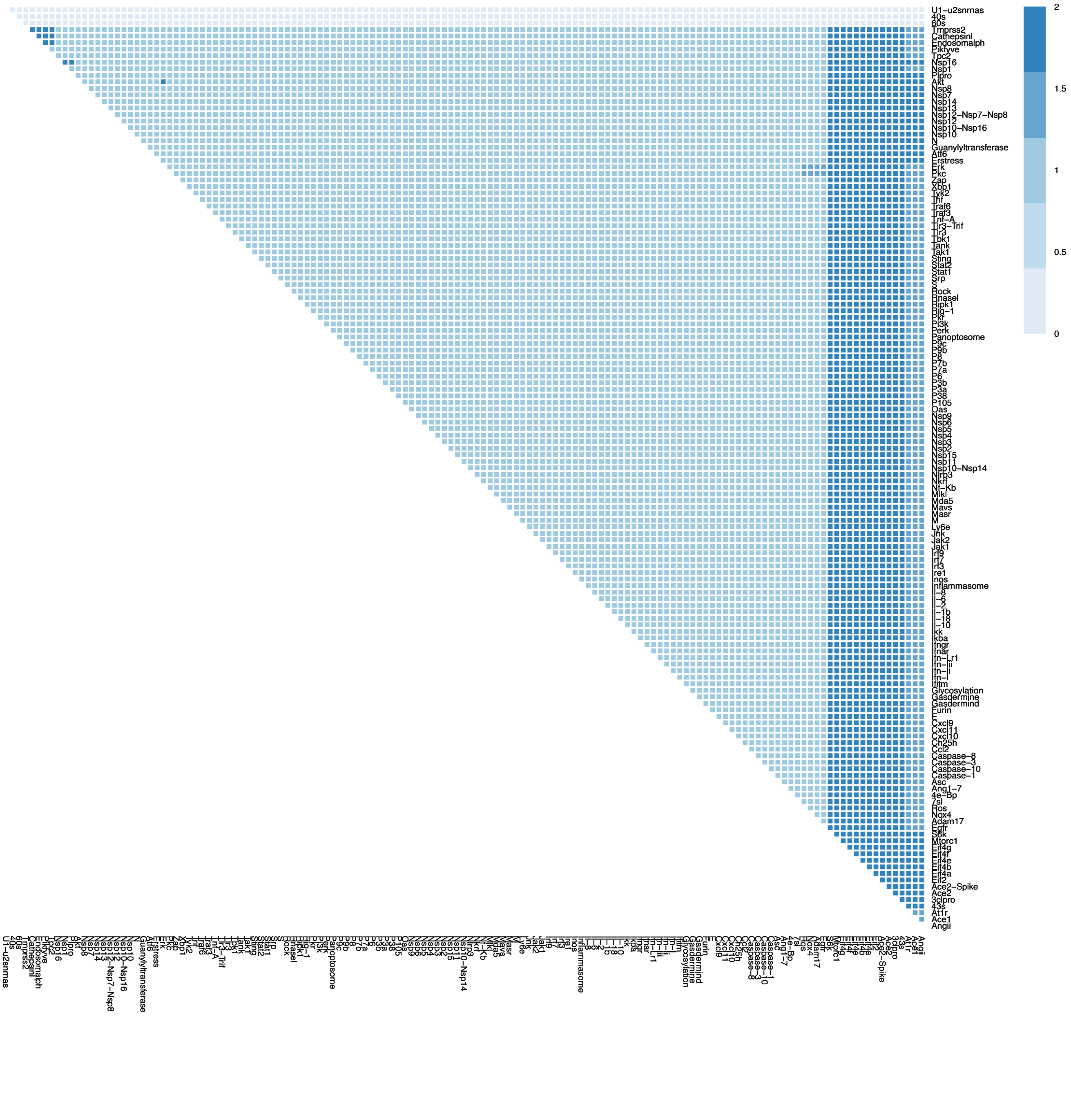

Supplementary Figure 10k. Effects of combination therapy (all nodes) on Host Protein Synthesis in early stage, severe COVID-19. The colour of the square corresponds to the level of the *HostProteinSynthesis* node at steady state upon inhbition of the nodes indicated on the x- and y-axis. This plot shows inhibition of all nodes representing druggable targets (proteins and complexes).

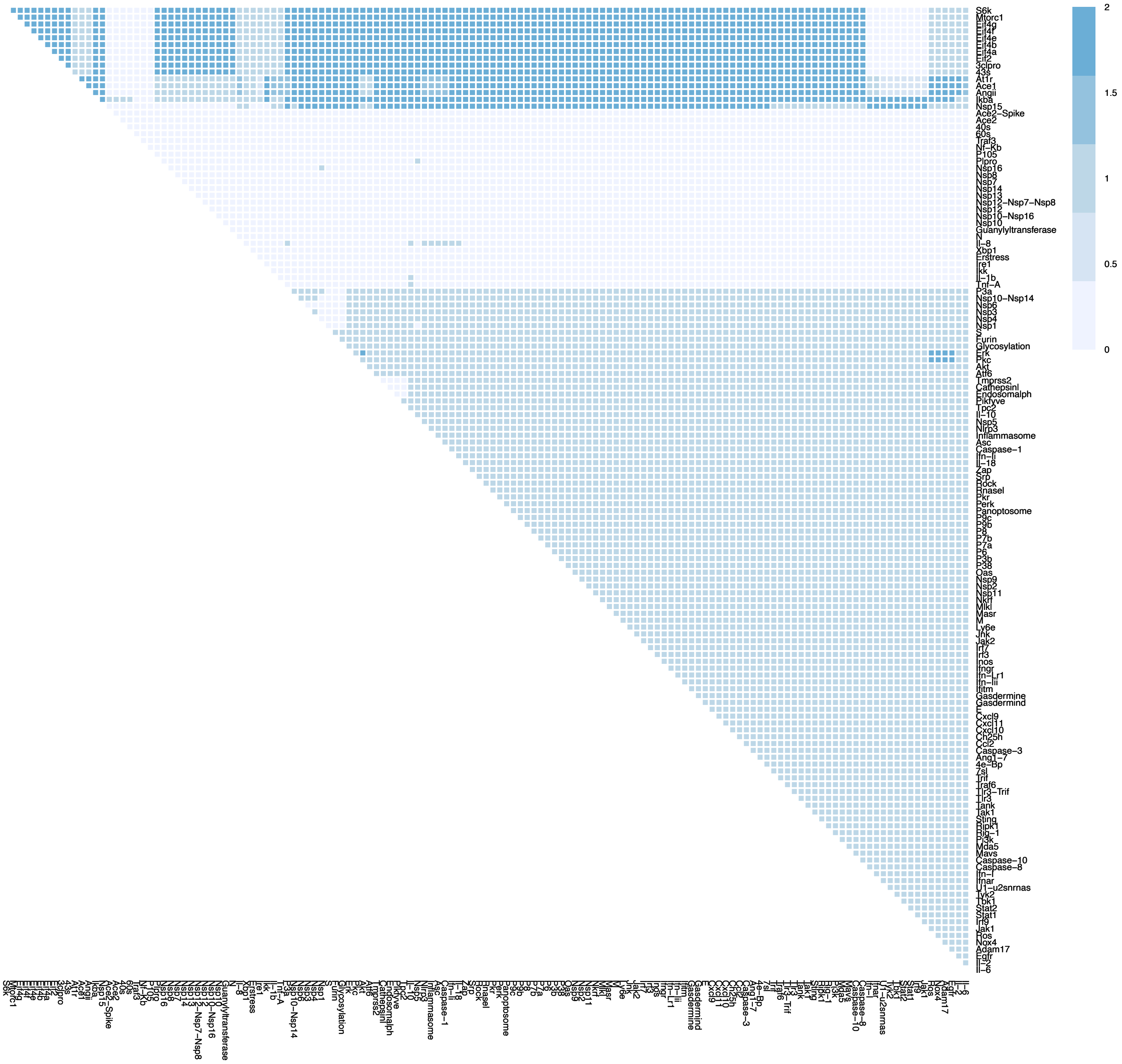

Supplementary Figure 10l. Effects of combination therapy (all nodes) on Inflammation in early stage, severe COVID-19. The colour of the square corresponds to the level of the *Inflammation* node at steady state upon inhbition of the nodes indicated on the x- and y-axis. This plot shows inhibition of all nodes representing druggable targets (proteins and complexes).

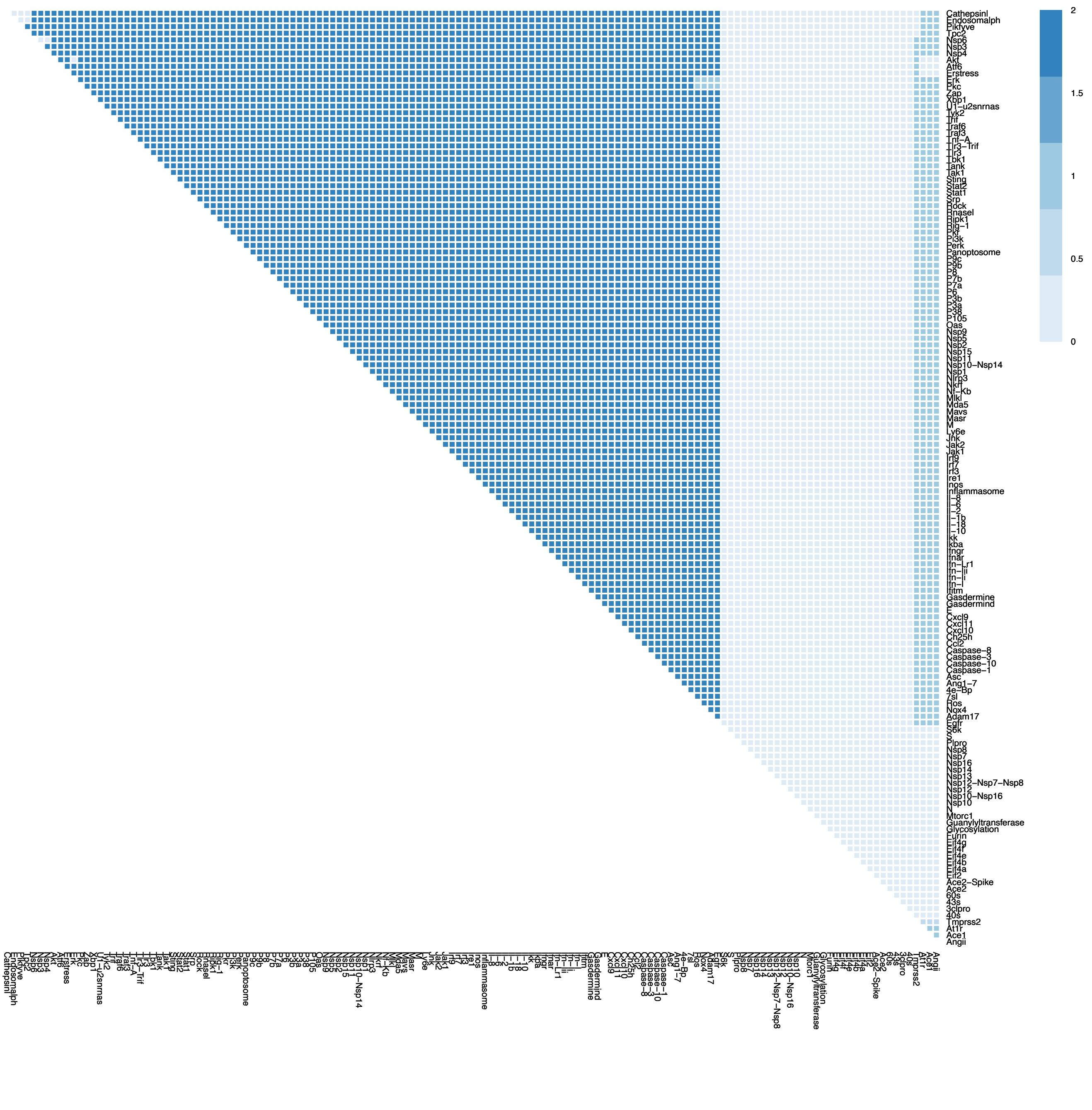

Supplementary Figure 10m. Effects of combination therapy (all nodes) on Syncytia Formation in early stage, severe COVID-19. The colour of the square corresponds to the level of the *SynctytiaFormation* node at steady state upon inhbition of the nodes indicated on the x- and y-axis. This plot shows inhibition of all nodes representing druggable targets (proteins and complexes).

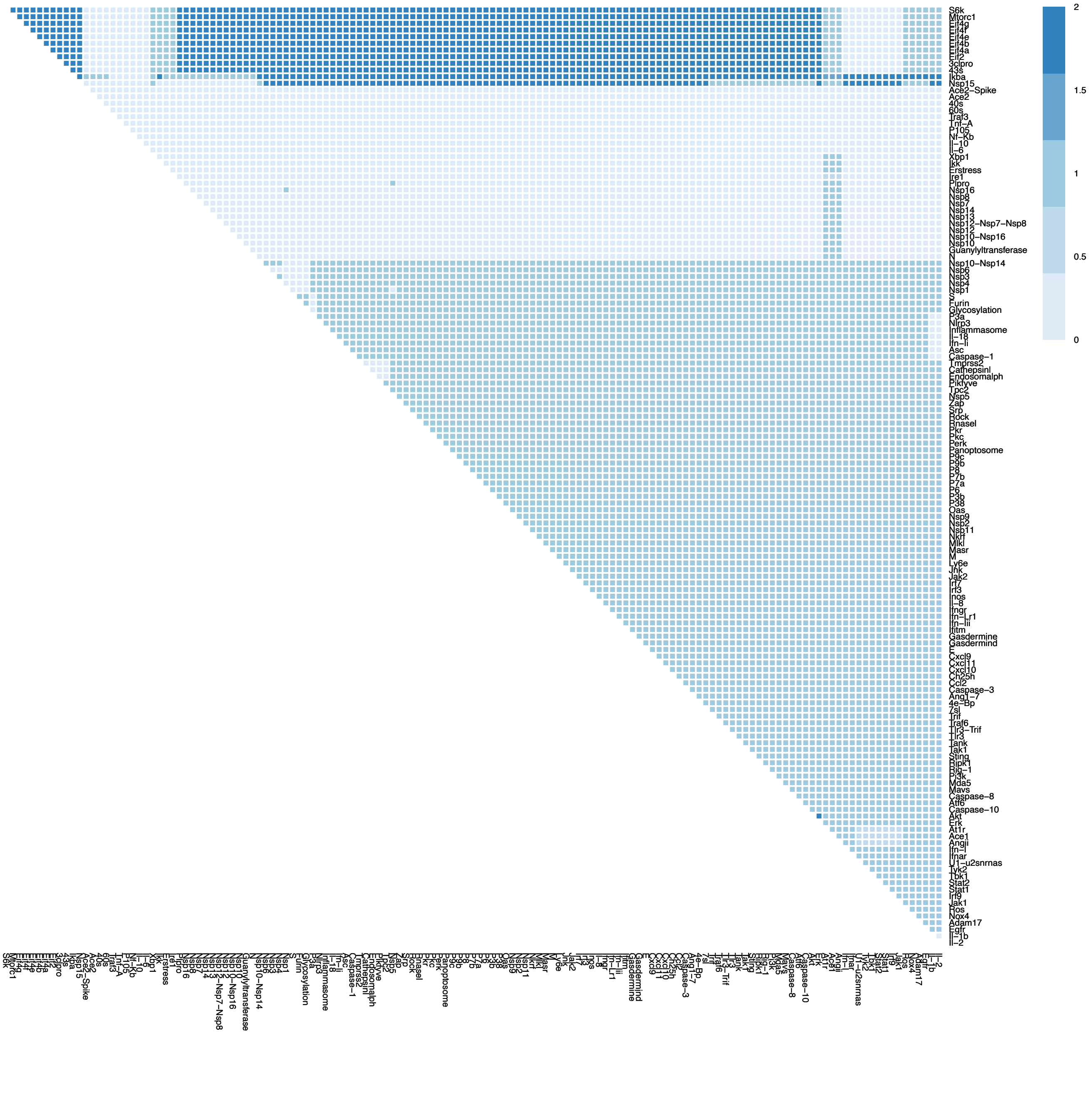

Supplementary Figure 10n. Effects of combination therapy (all nodes) on T-cell Infiltration in early stage, severe COVID-19. The colour of the square corresponds to the level of the *T-cellInfiltration* node at steady state upon inhbition of the nodes indicated on the x- and y-axis. This plot shows inhibition of all nodes representing druggable targets (proteins and complexes).

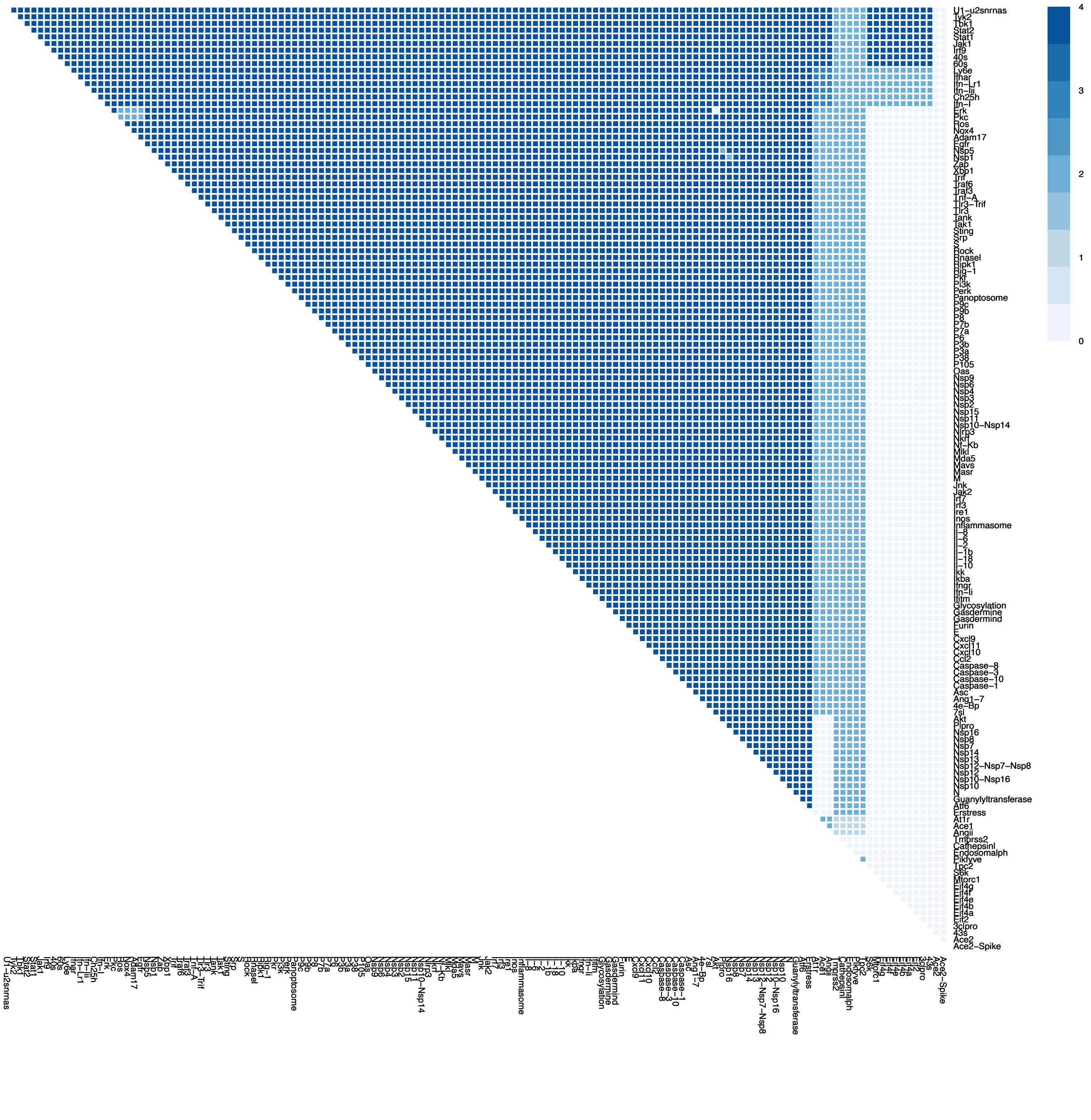

Supplementary Figure 10o. Effects of combination therapy (all nodes) on Viral Entry in early stage, severe COVID-19. The colour of the square corresponds to the level of the *ViralEntry* node at steady state upon inhbition of the nodes indicated on the x- and y-axis. This plot shows inhibition of all nodes representing druggable targets (proteins and complexes).

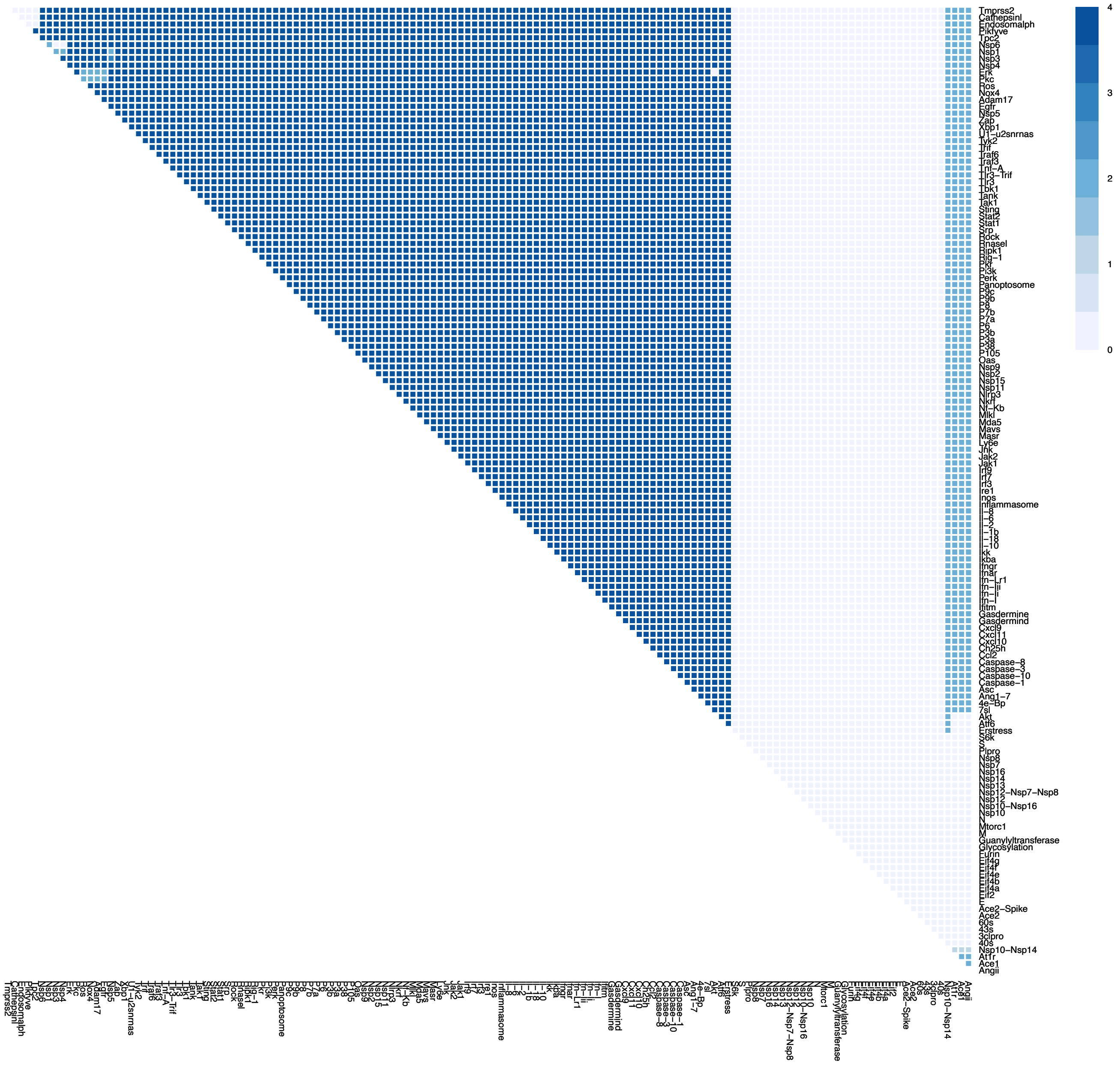

Supplementary Figure 10p. Effects of combination therapy (all nodes) on Viral Replication in early stage, severe COVID-19. The colour of the square corresponds to the level of the *ViralReplication* node at steady state upon inhbition of the nodes indicated on the x- and y-axis. This plot shows inhibition of all nodes representing druggable targets (proteins and complexes).

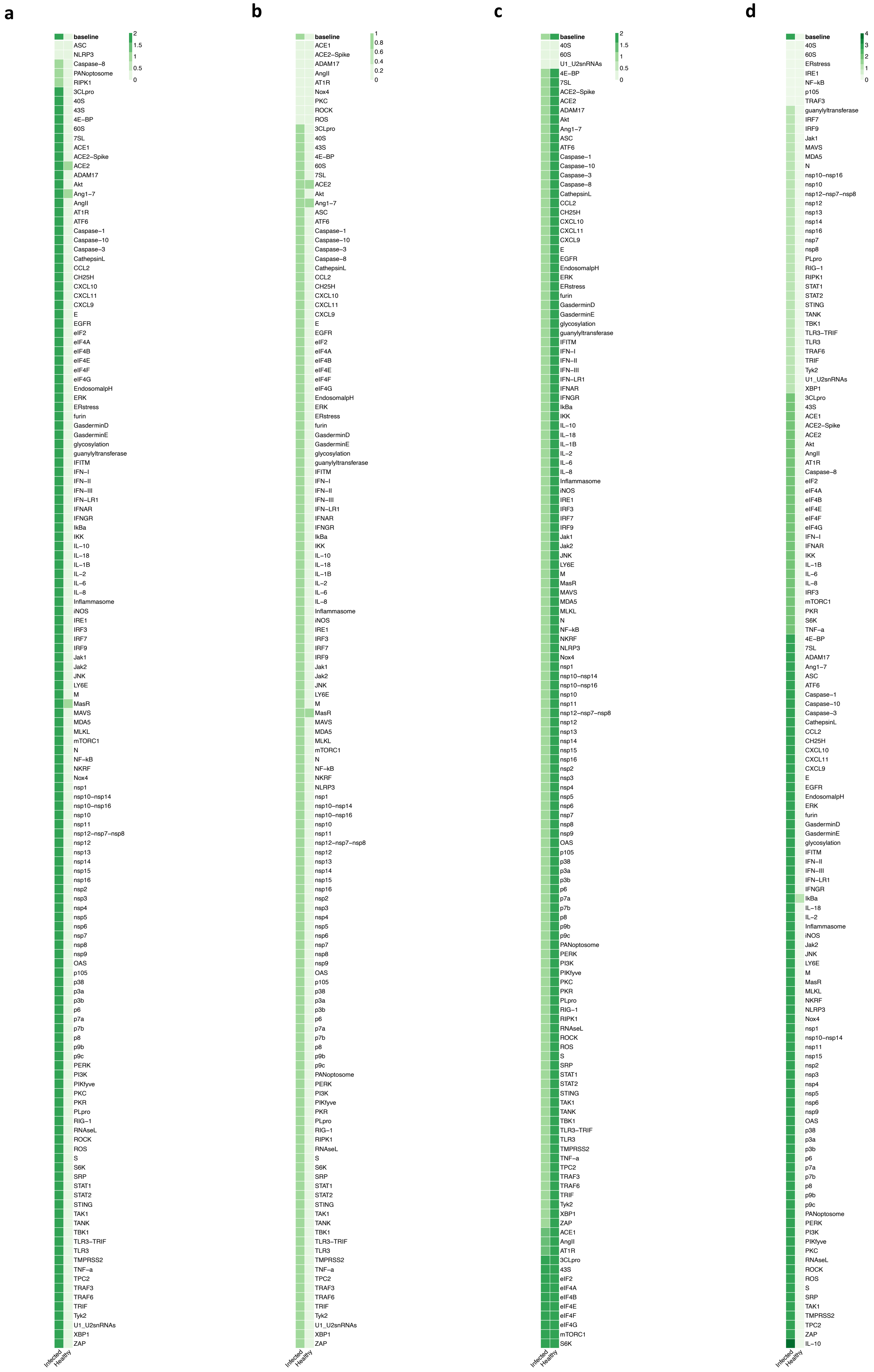

Supplementary Figure 11 a-d. Effects of single therapies (all nodes) in late stage, severe COVID-19. Columns represent infected and healthy conditions, rows represent node inhibited. Showing the effect on: (**a**) Cell Death. (**b**) Fibrosis. (**c**) Host Protein Synthesis. (**d**) Inflammation.

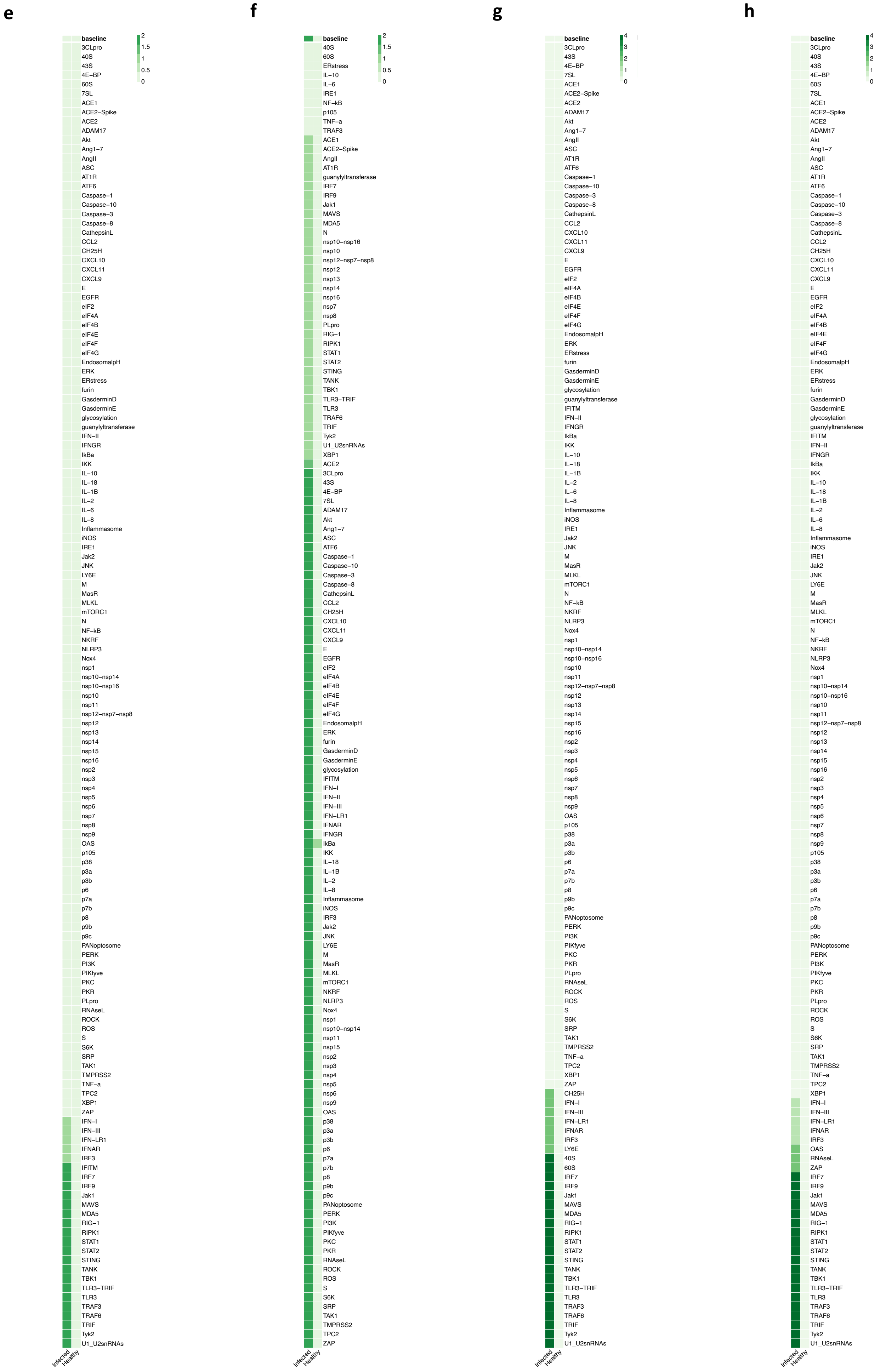

Supplementary Figure 11 e-h. Effects of single therapies (all nodes) in late stage, severe COVID-19. Columns represent infected and healthy conditions, rows represent node inhibited. Showing the effect on: (**e**) Syncytia Formation. (**f**) T-cell Infiltration. (**g**) Viral Entry. (**h**) Viral Replication.

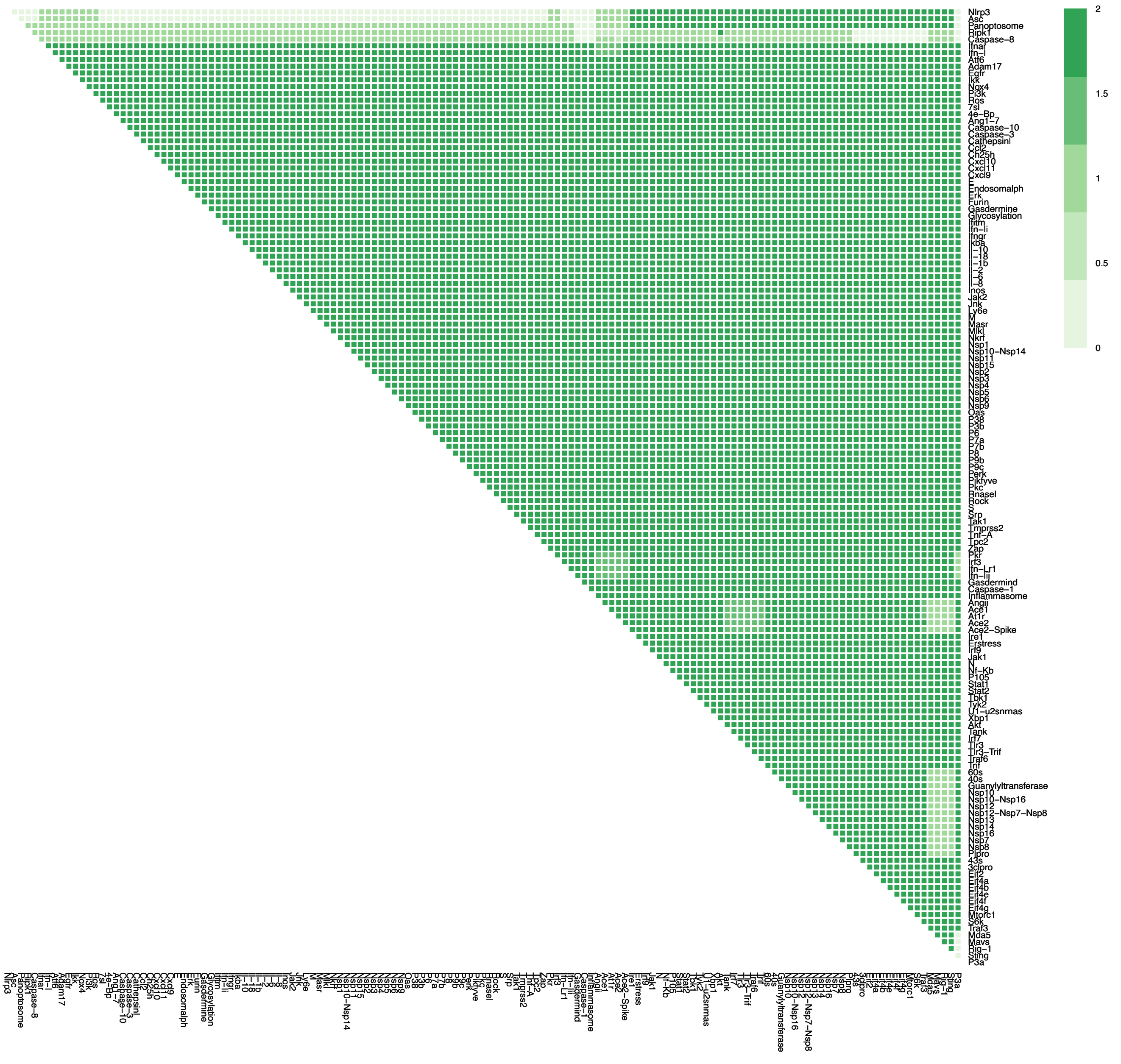

Supplementary Figure 11i. Effects of combination therapy (all nodes) on Cell Death in late stage, severe COVID-19. The colour of the square corresponds to the level of the C*ellDeath* node at steady state upon inhbition of the nodes indicated on the x- and y-axis. This plot shows inhibition of all nodes representing druggable targets (proteins and complexes).

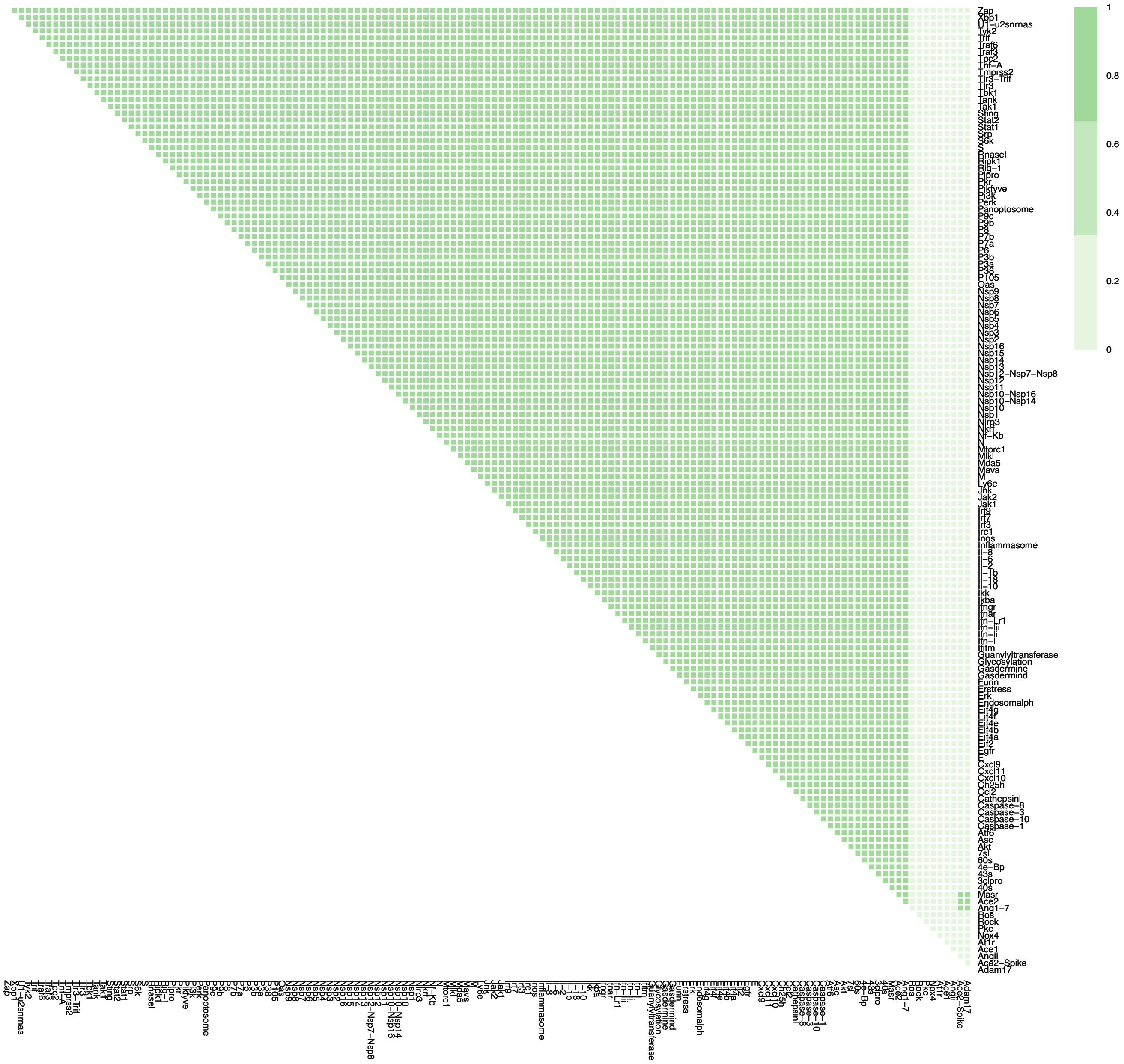

Supplementary Figure 11j. Effects of combination therapy (all nodes) on Fibrosis in late stage, severe COVID-19. The colour of the square corresponds to the level of the *Fibrosis* node at steady state upon inhbition of the nodes indicated on the x- and y-axis. This plot shows inhibition of all nodes representing druggable targets (proteins and complexes).

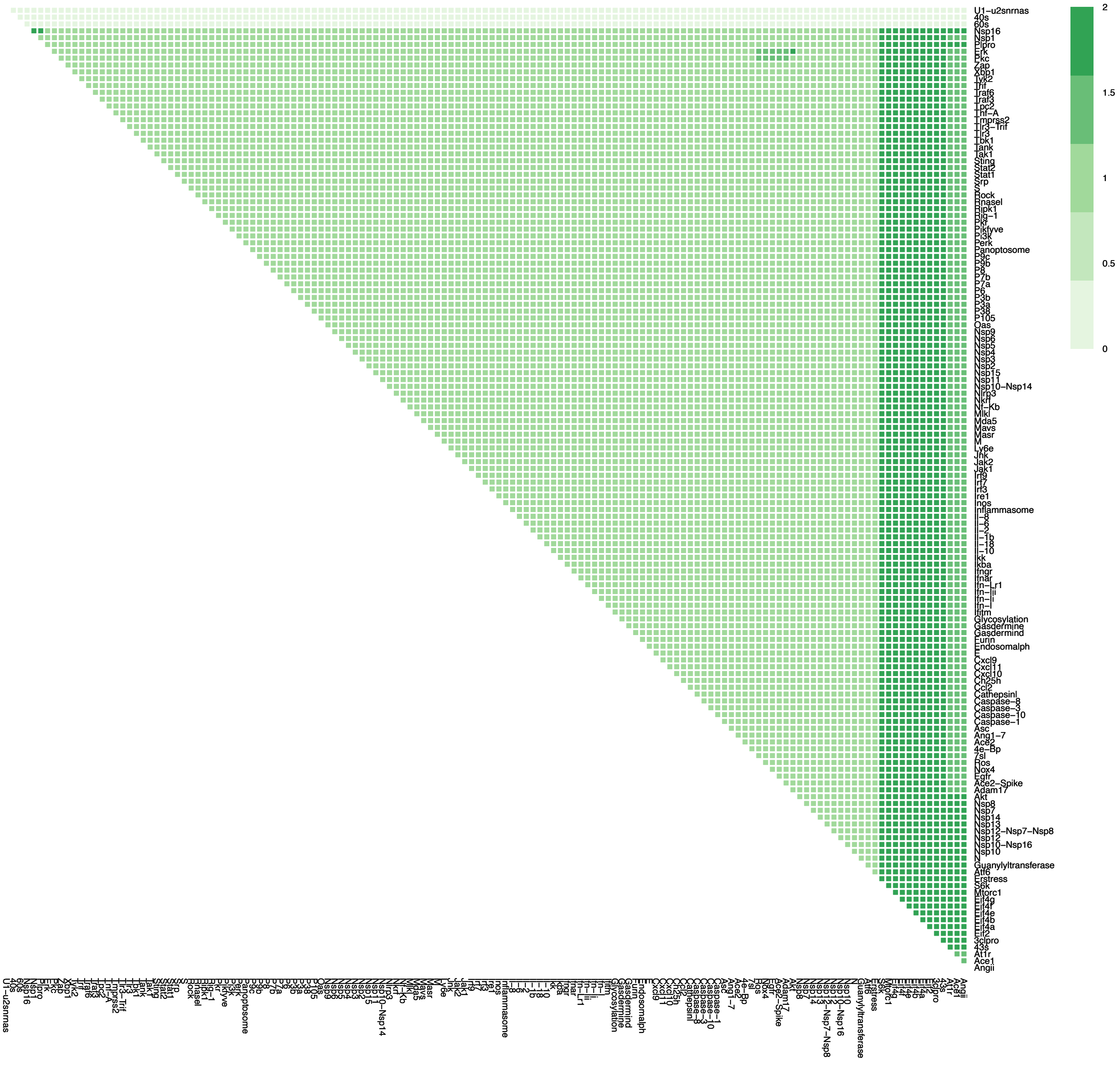

Supplementary Figure 11k. Effects of combination therapy (all nodes) on Host Protein Synthesis in late stage, severe COVID-19. The colour of the square corresponds to the level of the *HostProteinSynthesis* node at steady state upon inhbition of the nodes indicated on the x- and y-axis. This plot shows inhibition of all nodes representing druggable targets (proteins and complexes).

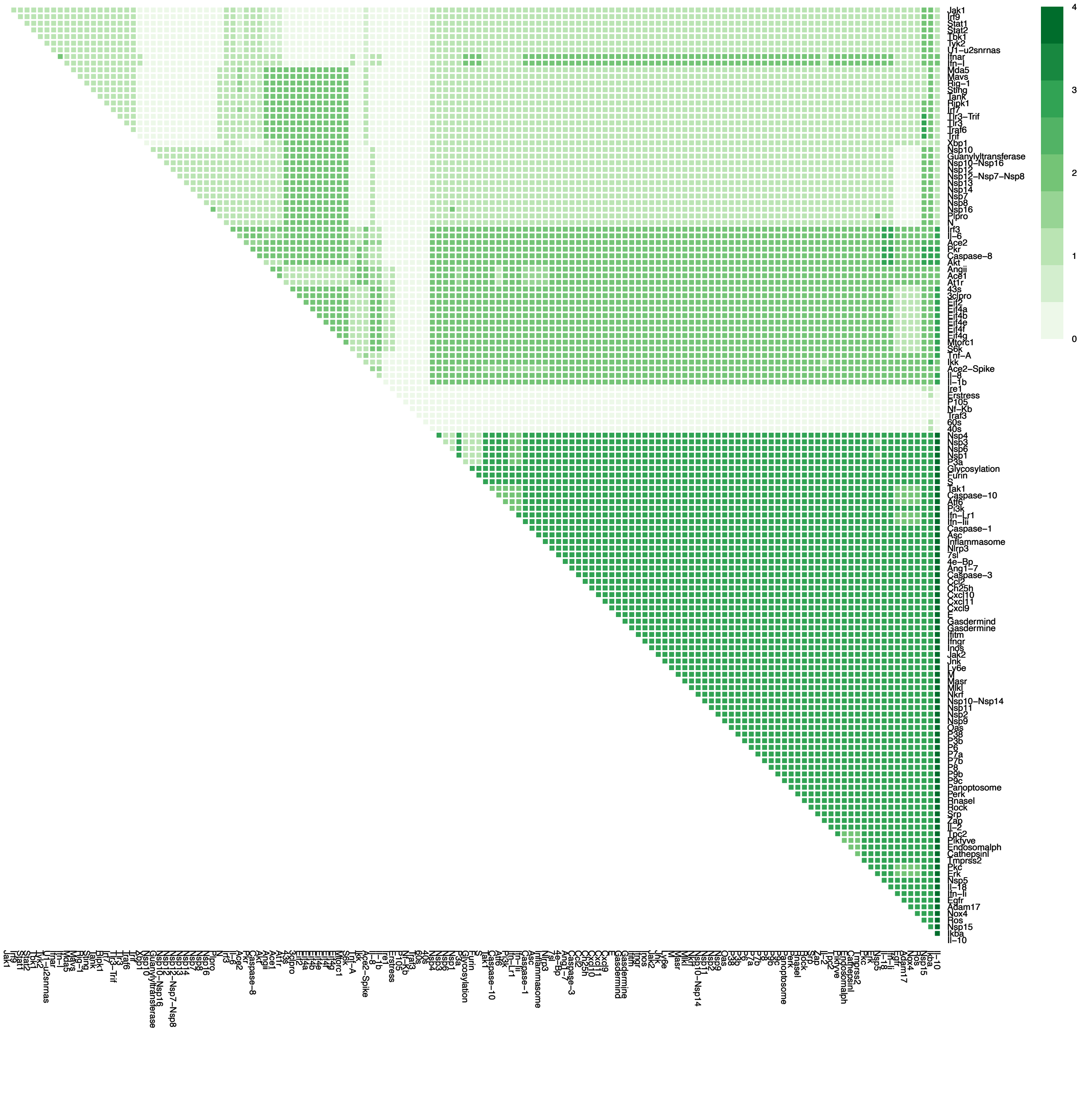

Supplementary Figure 11l. Effects of combination therapy (all nodes) on Inflammation in late stage, severe COVID-19. The colour of the square corresponds to the level of the *Inflammation* node at steady state upon inhbition of the nodes indicated on the x- and y-axis. This plot shows inhibition of all nodes representing druggable targets (proteins and complexes).

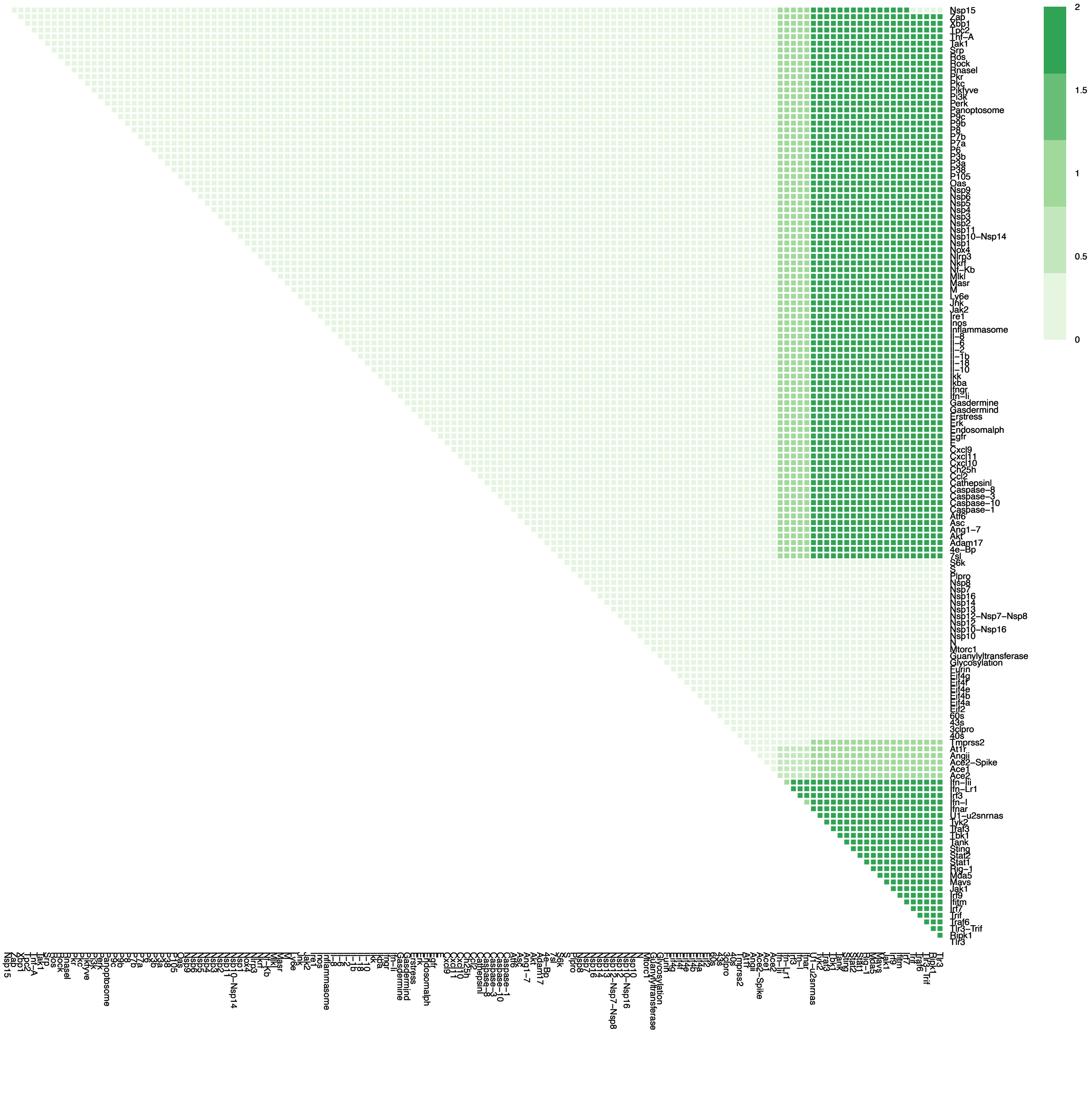

Supplementary Figure 11m. Effects of combination therapy (all nodes) on Syncytia Formation in late stage, severe COVID-19. The colour of the square corresponds to the level of the *Syncytia-Formation* node at steady state upon inhbition of the nodes indicated on the x- and y-axis. This plot shows inhibition of all nodes representing druggable targets (proteins and complexes).

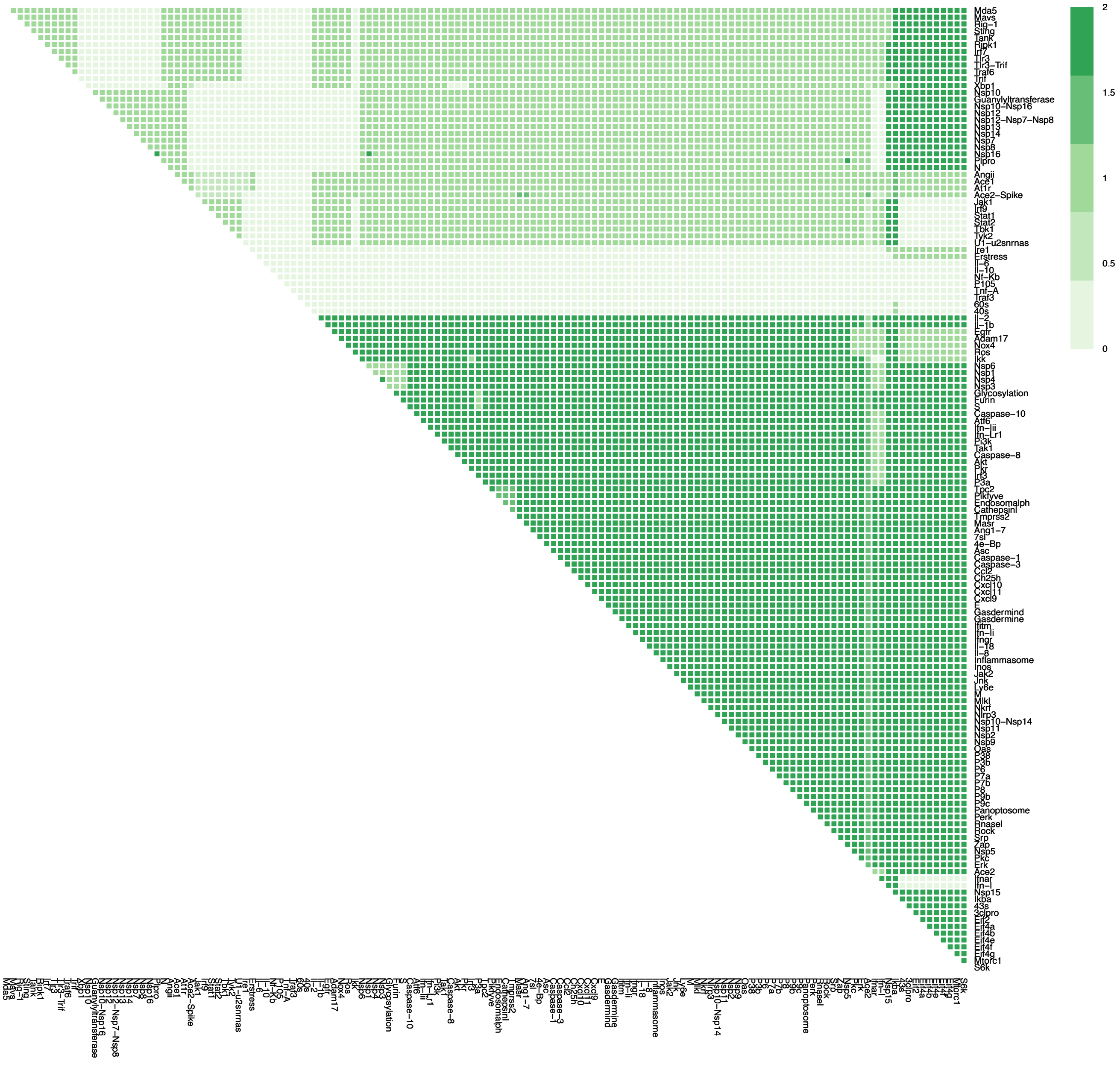

Supplementary Figure 11n. Effects of combination therapy (all nodes) on T-cell Infiltration in late stage, severe COVID-19. The colour of the square corresponds to the level of the *T-cellInfiltration* node at steady state upon inhbition of the nodes indicated on the x- and y-axis. This plot shows inhibition of all nodes representing druggable targets (proteins and complexes).

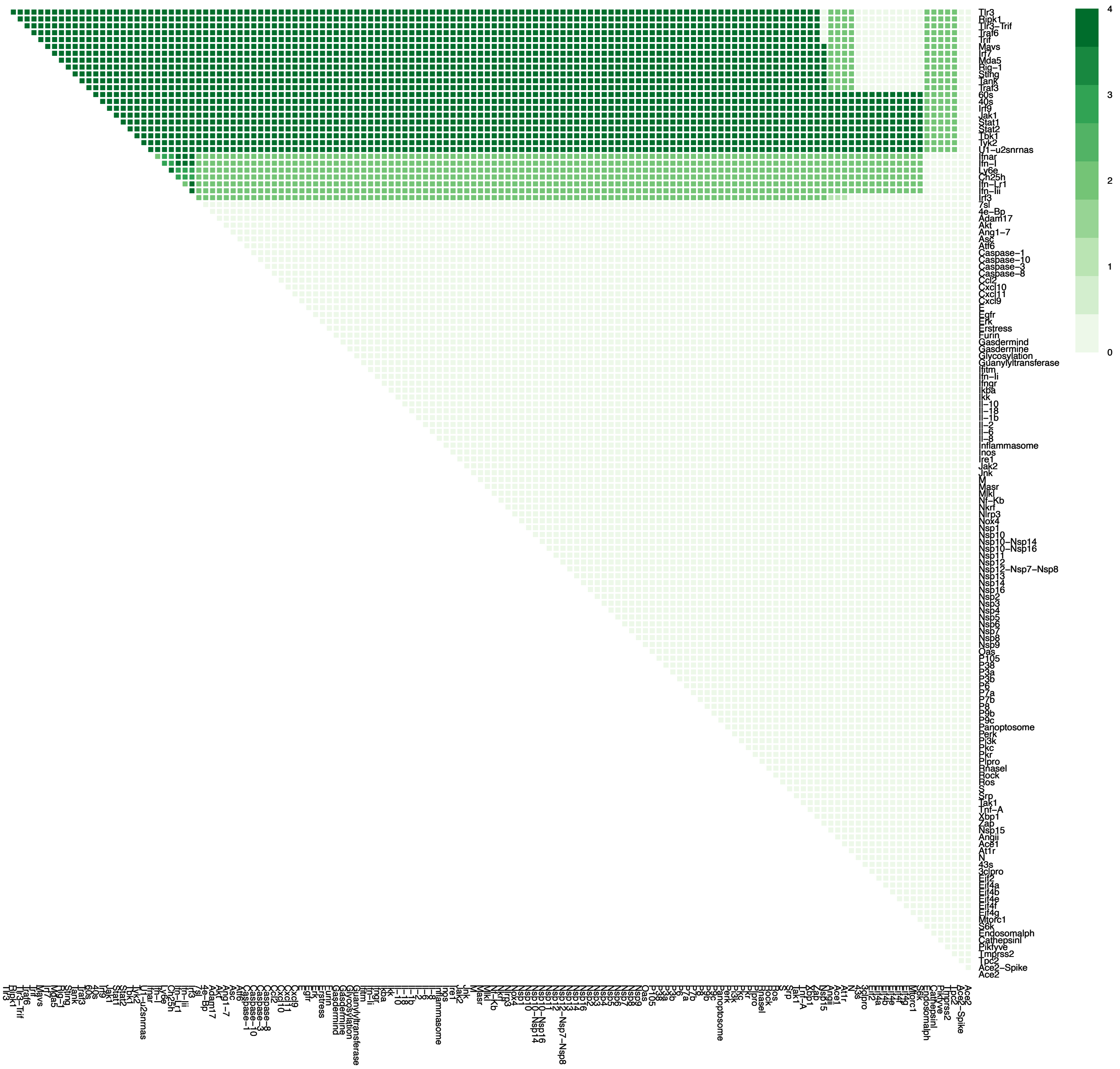

Supplementary Figure 11o. Effects of combination therapy (all nodes) on Viral Entry in late stage, severe COVID-19. The colour of the square corresponds to the level of the *ViralEntry* node at steady state upon inhbition of the nodes indicated on the x- and y-axis. This plot shows inhibition of all nodes representing druggable targets (proteins and complexes).

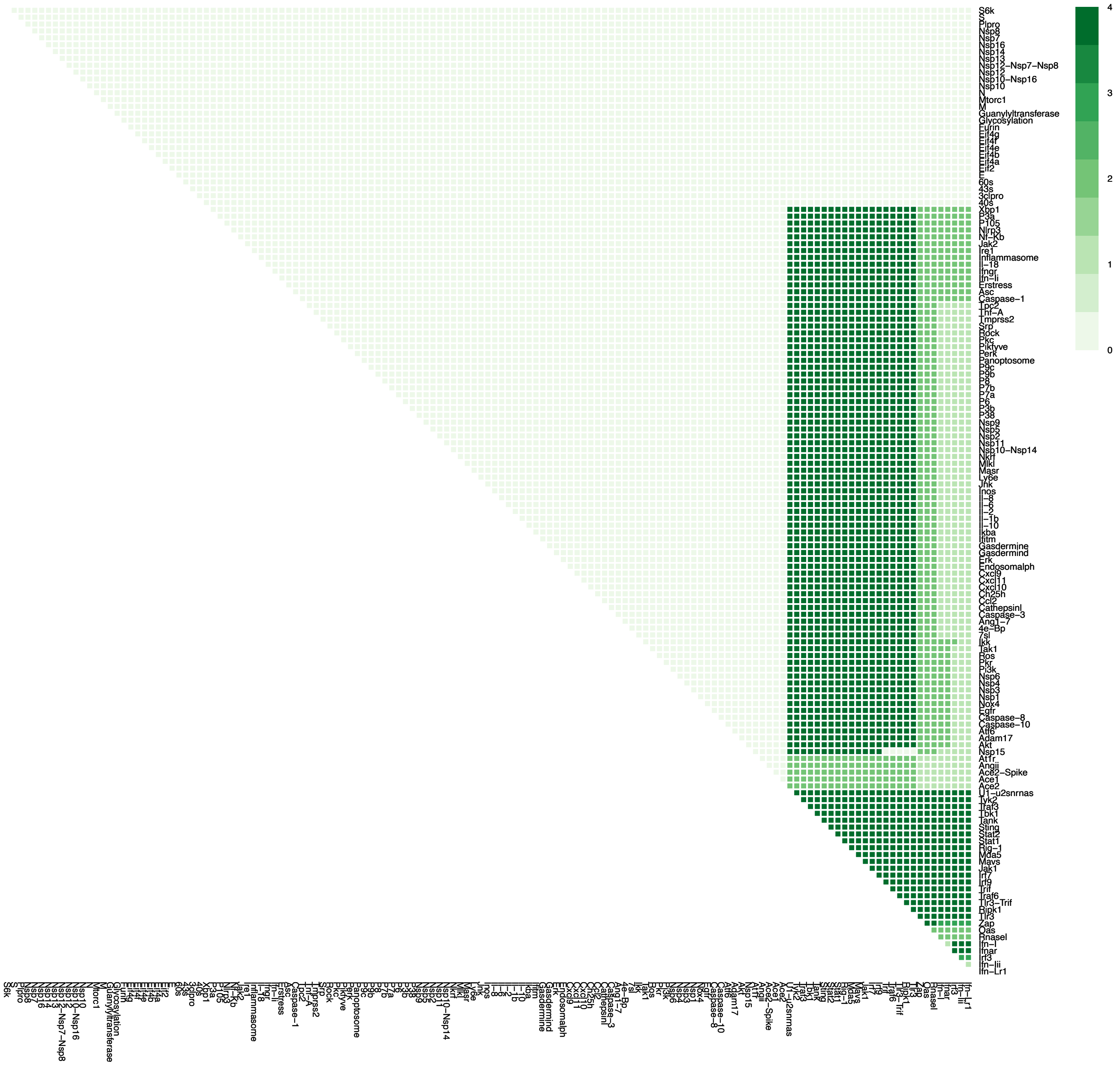

Supplementary Figure 11p. Effects of combination therapy (all nodes) on Viral Replication in late stage, severe COVID-19. The colour of the square corresponds to the level of the *ViralReplication* node at steady state upon inhbition of the nodes indicated on the x- and y-axis. This plot shows inhibition of all nodes representing druggable targets (proteins and complexes).

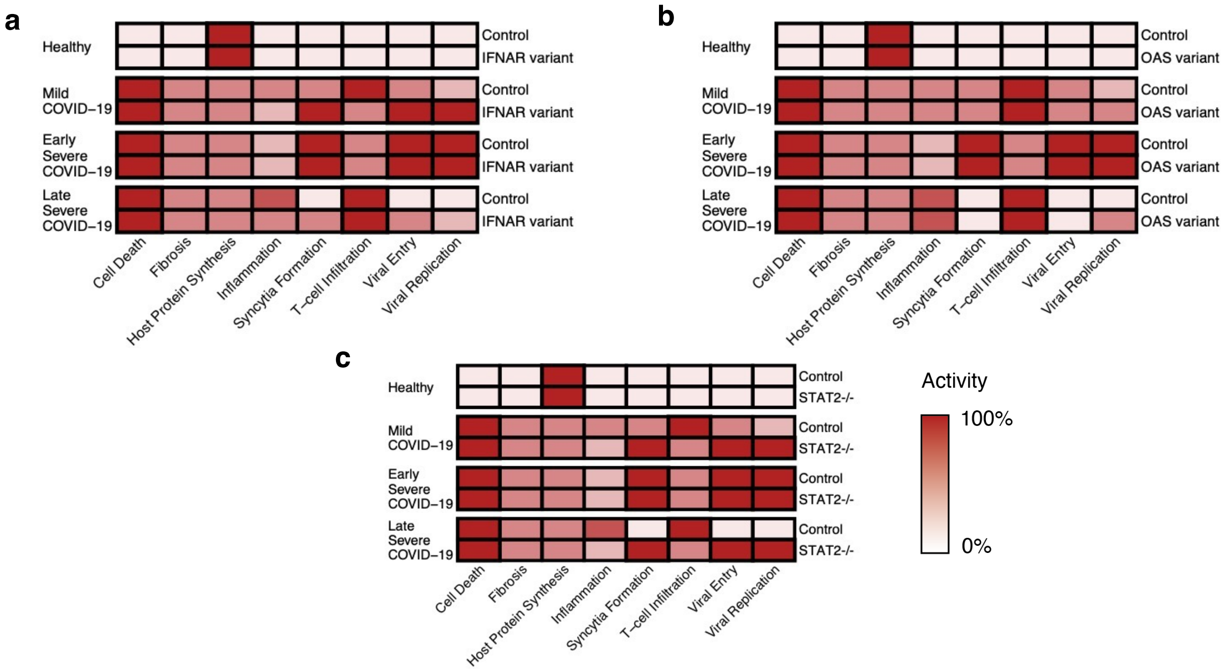

Supplementary Figure 12. Genetic risk factors associated with severe COVID-19. The behaviour of a loss-of-function mutation in IFNAR (**a**), OAS (**b**) and STAT2 (**c**) under the four conditions simulated by the model. All nodes normalized to be between 0 and 100%.

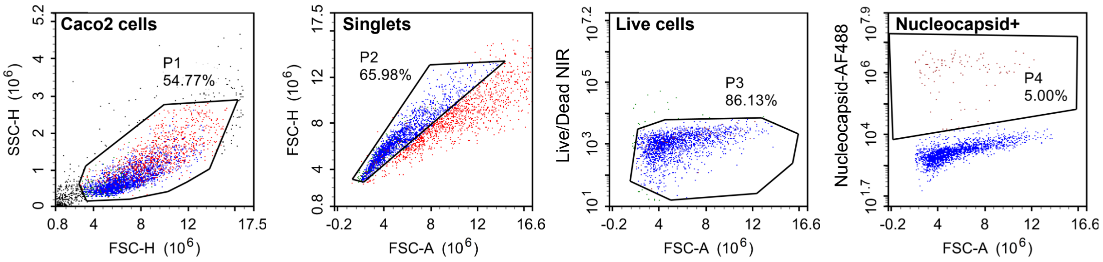

Supplementary Figure 13: Gating strategy used to identify expression of N protein and viability in Caco-2 cells. This gating strategy was utilised in Figure 6 and Supplementary Figures 6 and 7.

Supplementary Table 1. Regulatory edges in the network model

From	To	Type	Explanation	Reference
3CLpro	nsp1_nsp2_nsp3_nsp4	Activator	3CLpro cleaves pp1a and pp1ab into nsp1-4 and all nsps 5 to 10	Fehr, A. et al. (2015)
3CLpro	nsp10	Activator	3CLpro cleaves pp1a and pp1ab into nsp1-4 and all nsps 5 to 10	Fehr, A. et al. (2015)
3CLpro	nsp11	Activator	3CLpro cleaves pp1a and pp1ab into nsp1-4 and all nsps 5 to 10	Fehr, A. et al. (2015)
3CLpro	nsp12	Activator	3CLpro cleaves pp1a and pp1ab into nsp1-4 and all nsps 5 to 10	Fehr, A. et al. (2015)
3CLpro	nsp13	Activator	3CLpro cleaves pp1a and pp1ab into nsp1-4 and all nsps 5 to 10	Fehr, A. et al. (2015)
3CLpro	nsp14	Activator	3CLpro cleaves pp1a and pp1ab into nsp1-4 and all nsps 5 to 10	Fehr, A. et al. (2015)
3CLpro	nsp15	Activator	3CLpro cleaves pp1a and pp1ab into nsp1-4 and all nsps 5 to 10	Fehr, A. et al. (2015)
3CLpro	nsp16	Activator	3CLpro cleaves pp1a and pp1ab into nsp1-4 and all nsps 5 to 10	Fehr, A. et al. (2015)
3CLpro	nsp5	Activator	3CLpro cleaves pp1a and pp1ab into nsp1-4 and all nsps 5 to 10	Fehr, A. et al. (2015)
3CLpro	nsp6	Activator	3CLpro cleaves pp1a and pp1ab into nsp1-4 and all nsps 5 to 10	Fehr, A. et al. (2015)
3CLpro	nsp7	Activator	3CLpro cleaves pp1a and pp1ab into nsp1-4 and all nsps 5 to 10	Fehr, A. et al. (2015)
3CLpro	nsp8	Activator	3CLpro cleaves pp1a and pp1ab into nsp1-4 and all nsps 5 to 10	Fehr, A. et al. (2015)
3CLpro	nsp9	Activator	3CLpro cleaves pp1a and pp1ab into nsp1-4 and all nsps 5 to 10	Fehr, A. et al. (2015)
40S	43S	Activator	43S ribosomal subunit assembled from 40S subunit and multiple eIFs, including eIF2-GTP	Sonenberg, N. et al. (2009)
40S	Cap-independentTranslation	Activator	40S subunit binds mRNA directly in cap-indepdendent translation	Walters, B. et al. (2016)
43S	HostCap-dependentTranslation	Activator	43S subunit required for cap-dependent translation	Sonenberg, N. et al. (2009)
43S	ViralCap-dependentTranslation	Activator	43S subunit required for cap-dependent translation	Sonenberg, N. et al. (2009)
4E-BP	eIF4E	Inhibitor	4E-BP binds eIF4E, preventing it from associating with eIF4F	Sonenberg, N. et al. (2009)
60S	Cap-independentTranslation	Activator	60S required for protein synthesis cap-indepdendent translation	Walters, B. et al. (2016)
60S	HostCap-dependentTranslation	Activator	60S subunit required for cap-dependent translation	Sonenberg, N. et al. (2009)
60S	ViralCap-dependentTranslation	Activator	60S subunit required for cap-dependent translation	Sonenberg, N. et al. (2009)
7SL	SRP	Activator	7SL RNA binds to SRP	Akopian, D. et al. (2013)
ACE1	AngII	Activator	ACE1 produces AngII	Alexandre, J. et al. (2020)
ACE2	ACE2-Spike	Activator	The virus enters the cell through interaction of the spike protein with ACE2	Zhou, P. (2020); Shang, J. et al. (2020)
ACE2	Ang1-7	Activator	ACE2 converts AngII to Ang1-7	Alexandre, J. et al. (2020)
ACE2-Spike	Ang1-7	Inhibitor	Interaction with spike causes shedding of ACE2 and may compete for binding of substrates, preventing conversion into Ang1-7	Ingraham, N. (2020); Wang, S. et al. (2008); Haga, S. (2008)
ACE2-Spike	ViralFusion	Activator	Interaction of the Spike with ACE2 allows entry of the viral genome	Hoffmann, M. (2020); Lan, J. (2020); Ou, X. (2020)
ADAM17	ACE2	Inhibitor	ADAM17 contributes to ACE2 shedding	Ingraham, N. (2020); Haga, S. (2008)
ADAM17	EGFR	Activator	ADAM17 required for EGFR transactivation, cleaves HB-EGF	Forrester, S. (2018)
Akt	Caspase-3	Inhibitor	Akt phosphorylates BAD and caspase-9, inhibiting apoptosis	Song, G. et al. (2005)
Akt	IKK	Activator	Akt promotes NF-kB activity through IKK	Song, G. et al. (2005)
Akt	mTORC1	Activator		Magnuson, B. et al. (2012)
Ang1-7	MasR	Activator	Ang1-7 binds to the MasR receptor	Alexandre, J. et al. (2020)
AngII	Ang1-7	Activator	ACE2 converts AngII to Ang1-7	Alexandre, J. et al. (2020)
AngII	AT1R	Activator	AngII binds to the AT1R receptor	Alexandre, J. et al. (2020)
Apoptosis	CellDeath	Activator	Cell death may occur via apoptosis, necroptosis or pyroptosis	
ASC	Inflammasome	Activator	ASC, NLRP3 and pro-caspase-1 form inflammasome leading to activation of Caspase-1	Lee, S. et al. (2020)
ASC	PANoptosome	Activator	PANoptosome formed from Caspase-8, NLRP3, ASC, RIPK1 as well as RIPK3 and FADD	Christgen, S. et al. (2020)
AT1R	Nox4	Activator	AngII stimulates Nox activity through AT1R	Forrester, S. (2018)
AT1R	p38	Activator	AT1R activity induces p38 activity	Forrester, S. (2018)
AT1R	PKC	Activator	PKCdelta activity is induced by active AT1R	Forrester, S. (2018)
ATF6	Akt	Activator	ATF6 activity causes Akt phosphorylation	Pathinayake, P. et al. (2018)
Cap-independentTranslation	HostProteinSynthesis	Activator	Host proteins can be produced by both cap-dependent and -independent mechanisms (less efficient and not all proteins)	Walters, B. et al. (2016)
Caspase-1	GasderminD	Activator	Caspase-1 drives pyroptosis through cleavage of Gasdermin D	Man, S. et al. (2017)
Caspase-1	IL-18	Activator	Caspase-1 cleaves pro-IL-18	Man, S. et al. (2017)
Caspase-1	IL-1B	Activator	Caspase-1 cleaves pro-IL-1B	Man, S. et al. (2017)
Caspase-10	IKK	Activator	Cytosolic PRRs can induce NF-kB through caspase8/10 using RIP1	Mogensen, T. (2009)
Caspase-3	Apoptosis	Activator	Caspase-3 drives apoptosis	Lee, S. et al. (2020)
Caspase-8	Caspase-3	Activator	Caspase-8 cleaves pro-caspase-3 to initiate cell death	Lee, S. et al. (2020)
Caspase-8	IKK	Activator	Cytosolic PRRs can induce NF-kB through caspase8/10 using RIP1	Mogensen, T. (2009)
Caspase-8	PANoptosome	Activator	PANoptosome formed from Caspase-8, NLRP3, ASC, RIPK1 as well as RIPK3 and FADD	Christgen, S. et al. (2020)
CathepsinL	VIralFusion	Activator	Fusion of viral and cellular membranes requires proteolytic cleavage of the spike.	Fehr, A. et al. (2015); Shang, J. et al. (2020); Hoffmann, M. (2020)
CH25H	ViralEntry	Inhibitor	ISG CH25H interferes with S protein membrane fusion by production of CH25 lipid	Zang, R. et al. (2020)
CH25H	ViralEntry1	Inhibitor	ISG CH25H interferes with S protein membrane fusion by production of CH25 lipid	Zang, R. et al. (2020)
CytosolicPRRs	Caspase-10	Activator	Cytosolic PRRs can induce NF-kB through caspase8/10 using RIP1	Mogensen, T. (2009)
CytosolicPRRs	Caspase-8	Activator	Cytosolic PRRs can induce NF-kB through caspase8/10 using RIP1	Mogensen, T. (2009)
CytosolicPRRs	TBK1	Activator	Cytosolic PRRs together with TRAF3 and TANK1 can induce IFN through TBK1	Mogensen, T. (2009)
DMVformation	ViralGenome	Activator	RNA replication occurs in DMVs	Snijder, E. (2020)
DMVformation	ViralsgRNAs	Activator	RNA replication occurs in DMVs	Snijder, E. (2020)
E	VirionERGIC	Activator	Structural proteins form virion in endoplasmic reticulum–Golgi intermediate compartment (ERGIC)	Fehr, A. et al. (2015)
EGFR	ERK	Activator	ERK1/2 activated by AT1R through PKC and EGFR	Forrester, S. (2018)
EGFR	ERstress	Activator	EGFR/ADAM17 transactivation contributes to AngII/AT1R induced ER stress.	Forrester, S. (2018)
EGFR	PI3K	Activator	PI3K induced by EGFR/ADAM17 transactivation by AngII/AT1R.	Forrester, S. (2018)
eIF2	43S	Activator	43S ribosomal subunit assembled from 40S subunit and multiple eIFs, including eIF2-GTP	Sonenberg, N. et al. (2009)
eIF4A	eIF4F	Activator	eIF4F complex fromed of eIF4A, E, G and others	Sonenberg, N. et al. (2009)
eIF4B	eIF4A	Activator	eIF4B activates eIF4A helicase activity	Magnuson, B. et al. (2012)
eIF4E	eIF4F	Activator	eIF4F complex fromed of eIF4A, E, G and others	Sonenberg, N. et al. (2009)
eIF4F	HostCap-dependentTranslation	Activator	eIF4F complex subunit required for cap-dependent translation	Sonenberg, N. et al. (2009)
eIF4F	ViralCap-dependentTranslation	Activator	eIF4F complex subunit required for cap-dependent translation	Sonenberg, N. et al. (2009)
eIF4G	eIF4F	Activator	eIF4F complex fromed of eIF4A, E, G and others	Sonenberg, N. et al. (2009)
EndosomalpH	ViralFusion	Activator	Low endosomal pH is required for viral fusion	Vincent, M. et al. (2005); Wang, M. et al. (2020)
ERK	mTORC1	Activator	ERK promotes mTORC1 activation through ihbition of Tsc2, inhibitor of mTORC1	Magnuson, B. et al. (2012)
ERstress	ATF6	Activator	ATF6 cleaved and moves to nucleus in repsonse to ER stress	Pathinayake, P. et al. (2018)
ERstress	IRE1	Activator	ER stress causes IRE1 self-activation	Pathinayake, P. et al. (2018)
ERstress	PERK	Activator	AngII/AT1R induces PERK activity through ER stress	Forrester, S. (2018)
furin	S	Activator	furin cleaves S protein to activate	Fehr, A. et al. (2015); Shang, J. et al. (2020)
GasderminD	Pyroptosis	Activator	Caspase-1 drives pyroptosis through cleavage of Gasdermin D	Man, S. et al. (2017)
GasderminE	Pyroptosis	Activator	PANoptosome induces pyroptosis through cleavage of Gasdermin E	Man, S. et al. (2017)
glycosylation	S	Activator	Spike protein is highly glycosylated, if this is blocked can no longer enter cells efficiently	Yang, Q. et al. (2020); Walls, A. et al. (2020)
guanylyltransferase	ViralsgRNAs	Activator	Unknown guanylyltransferase required for sgRNA capping, suspected to be host protein	Nakagawa, K. et al. (2016)
HostCap-dependentTranslation	HostProteinSynthesis	Activator	Host proteins can be produced by both cap-dependent and -independent mechanisms (less efficient and not all proteins)	Walters, B. et al. (2016)
HostmRNAs	HostProteinSynthesis	Activator	Host proteins can be produced by both cap-dependent and -independent mechanisms (less efficient and not all proteins)	Walters, B. et al. (2016)
HostProteinSynthesis	IFN-I	Activator	IRF7 and IRF3 drive synthesis of type I IFNs	Sa Ribero, M. et al. (2020)
HostProteinSynthesis	IFN-I_SG	Activator	IIFN-I_SG leads to synthesis of ISG proteins	Sa Ribero, M. et al. (2020)
HostProteinSynthesis	IFN-II_SG	Activator	IFN-II_SG leads to synthesis of ISG proteins	
HostProteinSynthesis	IFN-III	Activator	IRF7 and IRF3 drive synthesis of type III IFNs	Wack, A. et al. (2015)
HostProteinSynthesis	IFN-III_SG	Activator	IFN-III_SG leads to synthesis of ISG proteins	Wack, A. et al. (2015)
HostProteinSynthesis	iNOS	Activator	TNF-a and IFN-II induce production of iNOS (NOS2)	Karki, R. et al. (2021)
IFITM	SyncytiaFormation	Inhibitor	S protein induces syncytia formation, IFITM (mainly IFITM1) prevents formation but only in absence of TMPRSS2	Buchrieser, J. et al. (2020)
IFN-I	IFNAR	Activator	Secreted IFN-I bind to heterodimeric IFNAR1/2 receptor	Sa Ribero, M. et al. (2020)
IFN-I_SG	CH25H	Activator	CH25H is an ISG	Zang, R. et al. (2020)
IFN-I_SG	IFITM	Activator	IFITM1-3 are ISGs.	Ribero, M. et al. (2020)

From	To	Type	Explanation	Reference
IFN-I_SG	LY6E	Activator	LY6E is an ISG	Sa Ribero, M. et al. (2020)
IFN-I_SG	NF-kB	Activator	Cytokines and other NF-kB targets are downstream of IFNAR signalling in a poorly understood mechanism	Makris, S. et al. (2017); Goritzka, M. et al. (2014)
IFN-I_SG	OAS	Activator	OASs are ISGs	Schoggins, J. et al. (2011)
IFN-I_SG	PKR	Activator	PKR is an ISG	Sa Ribero, M. et al. (2020)
IFN-I_SG	ZAP	Activator	ISG ZAP restricts viral RNA	Nchioua, R. et al. (2020)
IFN-II	CCL2	Activator	CXCL9, CXCL10, CXCL11 and CCL2 are induced in lung cells by TNF-a, IL-1B and IFN-g	Pechkovsky, D. et al. (2005)
IFN-II	CXCL10	Activator	CXCL9, CXCL10, CXCL11 and CCL2 are induced in lung cells by TNF-a, IL-1B and IFN-g	Pechkovsky, D. et al. (2005)
IFN-II	CXCL11	Activator	CXCL9, CXCL10, CXCL11 and CCL2 are induced in lung cells by TNF-a, IL-1B and IFN-g	Pechkovsky, D. et al. (2005)
IFN-II	CXCL9	Activator	CXCL9, CXCL10, CXCL11 and CCL2 are induced in lung cells by TNF-a, IL-1B and IFN-g	Pechkovsky, D. et al. (2005)
IFN-II	IFNGR	Activator	IFN-II activates IFNGR1/2	Ivashkiv, L. (2018)
IFN-II	Inflammation	Inhibitor	anti-inflammatory cytokine	Hamid, Q. et al. (2009)
IFN-II	TNF-a	Activator	TNF-a produced by a range of immune cells in response to many stimuli including IFN-II, IL-1 and IL-2 and NF-kB	Falvo, J. et al. (2010)
IFN-II_SG	iNOS	Activator	TNF-a and IFN-II induce production of iNOS (NOS2)	Karki, R. et al. (2021)
IFN-II_SG	ZAP	Activator	ISG ZAP restricts viral RNA	Nchioua, R. et al. (2020)
IFN-III	IFN-LR1	Activator	IFN-III interacts with heterodimeric IFN-lambdaR1, IL-10R2 receptor	Wack, A. et al. (2015)
IFN-III_SG	CH25H	Activator	CH25H is an ISG	Zang, R. et al. (2020)
IFN-III_SG	IFITM	Activator	IFITM1-3 are ISGs.	Ribero, M. et al. (2020)
IFN-III_SG	LY6E	Activator	LY6E is an ISG	Sa Ribero, M. et al. (2020)
IFN-III_SG	OAS	Activator	OASs are ISGs	Schoggins, J. et al. (2011)
IFN-III_SG	PKR	Activator	PKR is an ISG	Sa Ribero, M. et al. (2020)
IFN-III_SG	ZAP	Activator	ISG ZAP restricts viral RNA	Nchioua, R. et al. (2020)
IFN-LR1	IFN-III_SG	Activator	IFN-III response requires IFN-LR1, Tyk2, Jak1, STAT1/2 and IRF9	Wack, A. et al. (2015)
IFNAR	IFN-I_SG	Activator	IFN-I response requires IFNAR, Tyk2, Jak1, STAT1/2 and IRF9	Barrat, F. et al. (2019)
IFNGR	IFN-II_SG	Activator	IFN-II response requires IFNGR, Jak2, Jak1, STAT1	Barrat, F. et al. (2019)
IkBa	NF-kB	Inhibitor	Phosphorylation of IkBa reduces its inhibitory activity towards NF-kB	Liu, T. et al. (2017)
IKK	IkBa	Inhibitor	IKK inhibits IkBa through phosphorylation	Liu, T. et al. (2017)
IL-10	Inflammation	Inhibitor	anti-inflammatory cytokine	Hamid, Q. et al. (2009)
IL-10	T-cellInfiltration	Activator	Speculative interaction based on inverse correlation	Chen, Z. et al. (2020), Diao, B. (2020), Liu, J. (2020)
IL-18	IFN-II	Activator	IFN-gamma produced by various immune cells in response to IL-12 or IL-18	Ivashkiv, L. (2018)
IL-1B	CCL2	Activator	CXCL9, CXCL10, CXCL11 and CCL2 are induced in lung cells by TNF-a, IL-1B and IFN-g	Pechkovsky, D. et al. (2005)
IL-1B	CXCL10	Activator	CXCL9, CXCL10, CXCL11 and CCL2 are induced in lung cells by TNF-a, IL-1B and IFN-g	Pechkovsky, D. et al. (2005)
IL-1B	CXCL11	Activator	CXCL9, CXCL10, CXCL11 and CCL2 are induced in lung cells by TNF-a, IL-1B and IFN-g	Pechkovsky, D. et al. (2005)
IL-1B	CXCL9	Activator	CXCL9, CXCL10, CXCL11 and CCL2 are induced in lung cells by TNF-a, IL-1B and IFN-g	Pechkovsky, D. et al. (2005)
IL-1B	Inflammation	Activator	pro-inflammatory cytokine	Hamid, Q. et al. (2009)
IL-1B	TNF-a	Activator	TNF-a produced by a range of immune cells in response to many stimuli including IFN-II, IL-1 and IL-2 and NF-kB	Falvo, J. et al. (2010)
IL-2	TNF-a	Activator	TNF-a produced by a range of immune cells in response to many stimuli including IFN-II, IL-1 and IL-2 and NF-kB	Falvo, J. et al. (2010)
IL-6	CCL2	Activator	Inhibition of IL-6 causes decrease in IFN-_, IL-8, IL-10 and CCL2 levels in Cytokine release syndrome	Tanaka, T. et al. (2016)
IL-6	IL-10	Activator	Inhibition of IL-6 causes decrease in IFN-_, IL-8, IL-10 and CCL2 levels in Cytokine release syndrome	Tanaka, T. et al. (2016)
IL-6	Inflammation	Activator	pro-inflammatory cytokine	Hamid, Q. et al. (2009)
IL-6	T-cellInfiltration	Activator	Speculative interaction based on inverse correlation	Chen, Z. et al. (2020), Diao, B. (2020)
IL-8	Inflammation	Activator	pro-inflammatory cytokine	Hamid, Q. et al. (2009)
ImmuneCells	IFN-II	Activator	IFN-gamma produced by various immune cells in response to IL-12 or IL-18	Ivashkiv, L. (2018)
ImmuneCells	IL-10	Activator	IL-10 produced by nearly all leukocytes	Kany, S. et al. (2019)
ImmuneCells	IL-2	Activator	IL-2 primarily produced by T cells	Liao, W. et al. (2013)
ImmuneCells	TNF-a	Activator	TNF-a produced by a range of immune cells in response to many stimuli including IFN-II, IL-1 and IL-2 and NF-kB	Falvo, J. et al. (2010)
Inflammasome	Caspase-1	Activator	ASC, NLRP3 and pro-caspase-1 form inflammasome leading to activation of Caspase-1	Lee, S. et al. (2020)
iNOS	NO	Activator	iNOS catalyzes production of NO	Karki, R. et al. (2021)
IRE1	IKK	Activator	IRE1 interacts with TRAF2, leading to JNK activation and IkBa inhibition	Pathinayake, P. et al. (2018)
IRE1	JNK	Activator	IRE1 interacts with TRAF2, leading to JNK acgivation and Ikk inhibition	Pathinayake, P. et al. (2018)
IRE1	XBP1	Activator	IRE1 splices XBP1 mRNA	Pathinayake, P. et al. (2018)
IRF3	IFN-I	Activator	IRF7 and IRF3 drive synthesis of type I IFNs	Sa Ribero, M. et al. (2020)
IRF3	IFN-III	Activator	IRF7 and IRF3 drive synthesis of type III IFNs	Wack, A. et al. (2015)
IRF7	IFN-I	Activator	IRF7 and IRF3 drive synthesis of type I IFNs	Sa Ribero, M. et al. (2020)
IRF7	IFN-III	Activator	IRF7 and IRF3 drive synthesis of type III IFNs	Wack, A. et al. (2015)
IRF9	IFN-I_SG	Activator	IFN-I response requires IFNAR, Tyk2, Jak1, STAT1/2 and IRF9	Barrat, F. et al. (2019)
IRF9	IFN-III_SG	Activator	IFN-III response requires IFN-LR1, Tyk2, Jak1, STAT1/2 and IRF9	Wack, A. et al. (2015)
Jak1	IFN-I_SG	Activator	IFN-I response requires IFNAR, Tyk2, Jak1, STAT1/2 and IRF9	Barrat, F. et al. (2019)
Jak1	IFN-II_SG	Activator	IFN-II response requires IFNGR, Jak2, Jak1, STAT1	Barrat, F. et al. (2019)
Jak1	IFN-III_SG	Activator	IFN-III response requires IFN-LR1, Tyk2, Jak1, STAT1/2 and IRF9	Wack, A. et al. (2015)
Jak2	IFN-II_SG	Activator	IFN-II response requires IFNGR, Jak2, Jak1, STAT1	Barrat, F. et al. (2019)
JNK	Caspase-3	Activator	Ire1 activation as a result of ER stress can cause apoptosis in a p38 and JNK mediated way	Redza-Dutordoir, M. et al. (2016)
LY6E	ViralEntry	Inhibitor	ISG LY6E interferes with S protein membrane fusion	Vabret, N. (2020); Pfaender, S. (2020); Zhao, X. et al. (2020)
LY6E	ViralEntry1	Inhibitor	ISG LY6E interferes with S protein membrane fusion	Vabret, N. (2020); Pfaender, S. (2020); Zhao, X. et al. (2020)
M	STAT1	Inhibitor	nsp1, nsp6, nsp13, p3a, M p7b inhibit STAT1 phosphorylation	Xia, H. et al. (2020)
M	VirionERGIC	Activator	Structural proteins form virion in endoplasmic reticulum–Golgi intermediate compartment (ERGIC)	Fehr, A. et al. (2015)
MasR	Nox4	Inhibitor	ACE2 and Ang1-7 protect against PF by reduction of Nox4 induction	Meng, Y. et al. (2015)
MasR	p38	Inhibitor	MasR opposed AT1R activity	Forrester, S. et al. (2018)
MAVS	CytosolicPRRs	Activator	Cytosolic PRR signalling requires PRRs MDA5 or RIG-1 and adaptors MAVS and STING	Mogensen, T. (2009)
MDA5	CytosolicPRRs	Activator	Cytosolic PRR signalling requires PRRs MDA5 or RIG-1 and adaptors MAVS and STING	Mogensen, T. (2009)
MLKL	Necroptosis	Activator	PANoptosome induces necroptosis through MLKL	Man, S. et al. (2017)
mTORC1	4E-BP	Inhibitor	phosphorylation of 4E-BP by mTOR prevents association with eIF4E	Sonenberg, N. et al. (2009)
mTORC1	eIF4G	Activator	mTOR responsible (possibly indirectly) for activatory eIF4G phosphorylation	Sonenberg, N. et al. (2009)
mTORC1	S6K	Activator	mTOR responsible (possibly indirectly) for activatory S6K phosphorylation	Sonenberg, N. et al. (2009)
N	IFN-I	Inhibitor	p6, p8 and N inhibit IFN-beta production and NF-kB responsive promoter	Li, J. et al. (2021)
N	IFN-III	Inhibitor	p6, p8 and N inhibit IFN-beta production and NF-kB responsive promoter, IFN-III has similar promoter to IFN-I	Wack, A. et al. (2015); Li, J. et al. (2021)
N	ViralGenome	Activator	N protein interacts with nsp3, this interaction required for RNA transcription.	Cong, Y. (2020)
N	ViralsgRNAs	Activator	N protein interacts with nsp3, this interaction required for RNA transcription.	Cong, Y. (2020)
N	VirionERGIC	Activator	Structural proteins form virion in endoplasmic reticulum–Golgi intermediate compartment (ERGIC)	Fehr, A. et al. (2015)
Necroptosis	CellDeath	Activator	Cell death may occur via apoptosis, necroptosis or pyroptosis	
NF-kB	Caspase-3	Inhibitor	NF-kB induces transcirption of pro-survival genes	Song, G. et al. (2005)
NF-kB	IL-18	Activator	NF-kB induces transcription of NLRP3, pro-IL-18 and pro-IL-1B	Liu, T. et al. (2017)
NF-kB	IL-1B	Activator	NF-kB induces transcription of NLRP3, pro-IL-18 and pro-IL-1B	Liu, T. et al. (2017)
NF-kB	IL-2	Activator	NF-kB induces production of many cytokines including TNF-a, IL-2, IL-6, IL-8, IL-12, CCL2	Liu, T. et al. (2017)
NF-kB	IL-6	Activator	NF-kB induces production of many cytokines including TNF-a, IL-2, IL-6, IL-8, IL-12, CCL2	Liu, T. et al. (2017)

From	To	Type	Explanation	Reference
NF-kB	IL-8	Activator	NF-kB induces production of many cytokines including TNF-a, IL-2, IL-6, IL-8, IL-12, CCL2	Liu, T. et al. (2017)
NF-kB	TNF-a	Activator	NF-kB induces production of many cytokines including TNF-a, IL-2, IL-6, IL-8, IL-12, CCL2	Liu, T. et al. (2017)
NKRF	IL-6	Inhibitor	NKRF modulates NF-kB activity towards IL-6 and IL-8 specifically	Vabret, N. (2020); Li, J. et al. (2021)
NKRF	IL-8	Inhibitor	NKRF modulates NF-kB activity towards IL-6 and IL-8 specifically	Vabret, N. (2020); Li, J. et al. (2021)
NLRP3	Inflammasome	Activator	ASC, NLRP3 and pro-caspase-1 form inflammasome leading to activation of Caspase-1	Lee, S. et al. (2020)
NLRP3	PANoptosome	Activator	PANoptosome formed from Caspase-8, NLRP3, ASC, RIPK1 as well as RIPK3 and FADD	Christgen, S. et al. (2020)
NO	Caspase-8	Activator	NO thought to act through caspase-8	Karki, R. et al. (2021)
Nox4	ROS	Activator	Nox4 is the Nox family protein primarily responsible for ROS production in PF	Hecker, L. (2009)
nsp1	Cap-independentTranslation	Inhibitor	nsp1 inhibits host translation and promotes viral translation through binding of the 40S ribosome	Banerjee, A. (2020); Thoms, M. (2020); Schubert, K. et al. (2020)
nsp1	HostCap-dependentTranslation	Inhibitor	nsp1 inhibits host translation and promotes viral translation through binding of the 40S ribosome	Banerjee, A. (2020); Thoms, M. (2020); Schubert, K. et al. (2020)
nsp1	STAT1	Inhibitor	nsp1, nsp6, nsp13, p3a, M p7b inhibit STAT1 phosphorylation	Xia, H. et al. (2020)
nsp1	ViralCap-dependentTranslation	Activator	nsp1 inhibits host translation and promotes viral translation through binding of the 40S ribosome	Banerjee, A. (2020); Thoms, M. (2020); Schubert, K. et al. (2020)
nsp1_nsp2_nsp3_nsp4	nsp1	Activator	nsp1-4 is cleaved into individual proteins by PLpro	Fehr, A. et al. (2015)
nsp1_nsp2_nsp3_nsp4	nsp2	Activator	nsp1-4 is cleaved into individual proteins by PLpro	Fehr, A. et al. (2015)
nsp1_nsp2_nsp3_nsp4	nsp3	Activator	nsp1-4 is cleaved into individual proteins by PLpro	Fehr, A. et al. (2015)
nsp1_nsp2_nsp3_nsp4	nsp4	Activator	nsp1-4 is cleaved into individual proteins by PLpro	Fehr, A. et al. (2015)
nsp10	NKRF	Inhibitor	Nsp9/10 stimulate IL6/8 production through inhibition of Nsp9/10	Li, J. et al. (2021)
nsp10	nsp10-nsp14	Activator	Interaction of nsp10 with nsp14 requirted for ExoN (proof-reading) activity but not N7-MTase activity.	Bouvet, M. et al. (2010)
nsp10	nsp10-nsp16	Activator	nsp16 2'O-MTase activity depends on interaction with nsp10.	Bouvet, M. et al. (2010)
nsp10-nsp14	ViralGenome	Activator	nsp10-nsp14 acts as a ExoN, proofreading the viral genome	Bouvet, M. et al. (2012)
nsp10-nsp16	ViralsgRNAs	Activator	nsp10-nsp16 functions as 2'O-MTase, ensuring viral mRNAs are correctly capped	Bouvet, M. et al. (2010)
nsp12	nsp12-nsp7-nsp8	Activator	Complex formed of nsp12-nsp7-nsp8 required for RdRp activity.	Subissi, L. (2014); Kirchdoerfer, R. et al. (2019); Chen, J. (2020)
nsp12-nsp7-nsp8	ViralGenome	Activator	Complex formed of nsp12-nsp7-nsp8 required for RdRp activity. Note nsp13 has been observed binding to this complex.	Subissi, L. (2014); Chen, J. (2020)
nsp12-nsp7-nsp8	ViralsgRNAs	Activator	Complex formed of nsp12-nsp7-nsp8 required for RdRp activity. Note nsp13 has been observed binding to this complex.	Subissi, L. (2014); Chen, J. (2020)
nsp13	IRF3	Inhibitor	nsp13, nsp14 and nsp15 found to inhibit nuclear localization of IRF3	Yuen, C. et al. (2020)
nsp13	STAT1	Inhibitor	nsp1, nsp6, nsp13, p3a, M p7b inhibit STAT1 phosphorylation	Xia, H. et al. (2020)
nsp13	STAT2	Inhibitor	nsp6, nsp13, p7a, p7b inhibit STAT2 phosphorylation	Xia, H. et al. (2020)
nsp13	ViralGenome	Activator	nsp13 helicase has been observed in complex with nsp12-nsp7-nsp8	Fehr, A. et al. (2015); Chen, J. (2020)
nsp13	ViralsgRNAs	Activator	nsp13 helicase has been observed in complex with nsp12-nsp7-nsp8	Fehr, A. et al. (2015); Chen, J. (2020)
nsp14	IRF3	Inhibitor	nsp13, nsp14 and nsp15 found to inhibit nuclear localization of IRF3	Yuen, C. et al. (2020)
nsp14	nsp10-nsp14	Activator	Interaction of nsp10 with nsp14 requirted for ExoN (proof-reading) activity but not N7-MTase activity.	Bouvet, M. et al. (2010); Bouvet, M. et al. (2012)
nsp14	ViralsgRNAs	Activator	nsp14 functions as N7-MTase, ensuring viral mRNAs are correctly capped	Bouvet, M. et al. (2010); Ogando, N. et al. (2020)
nsp15	CytosolicPRRs	Inhibitor	nsp15 EndoU activity cleaves polyUs from viral sgRNAs, preventing their recognition by MDA5.	Hackbart, M. et al. (2020)
nsp15	IRF3	Inhibitor	nsp13, nsp14 and nsp15 found to inhibit nuclear localization of IRF3	Yuen, C. et al. (2020)
nsp16	nsp10-nsp16	Activator	nsp16 2'O-MTase activity depends on interaction with nsp10.	Bouvet, M. et al. (2010); Viswanathan, T. (2020)
nsp16	U1_U2snRNAs	Inhibitor	nsp16 binds U1/U2 snRNAs inhibiting splicing	Banerjee, A. (2020)
nsp3	DMVformation	Activator	Double Membrane Vesicles are formed by nsp3, nsp4 and nsp6, these DMVs contain a pore consiting of a hexameric nsp3 complex	Angelini, M. et al. (2013); Wolff, G. (2020)
nsp3	PLpro	Activator	PL protease activity lies in nsp3, uncleaved pp1a and pp1ab also contain this domain	Fehr, A. et al. (2015)
nsp4	DMVformation	Activator	Double Membrane Vesicles are formed by nsp3, nsp4 and nsp6	Angelini, M. et al. (2013)
nsp5	3CLpro	Activator	3CL protease activity lies in nsp5, uncleaved pp1a and pp1ab also contain this domain	Fehr, A. et al. (2015)
nsp6	DMVformation	Activator	Double Membrane Vesicles are formed by nsp3, nsp4 and nsp6	Angelini, M. et al. (2013)
nsp6	IRF3	Inhibitor	nsp6 binds TBK1 preventing activation of IRF3	Xia, H. et al. (2020)
nsp6	STAT1	Inhibitor	nsp1, nsp6, nsp13, p3a, M p7b inhibit STAT1 phosphorylation	Xia, H. et al. (2020)
nsp6	STAT2	Inhibitor	nsp6, nsp13, p7a, p7b inhibit STAT2 phosphorylation	Xia, H. et al. (2020)
nsp7	nsp12-nsp7-nsp8	Activator	Complex formed of nsp12-nsp7-nsp8 required for RdRp activity.	Subissi, L. (2014); Kirchdoerfer, R. et al. (2019); Chen, J. (2020)
nsp8	7SL	Inhibitor	nsp8 and 9 bind and inhibit 7SL	Banerjee, A. (2020)
nsp8	nsp12-nsp7-nsp8	Activator	Complex formed of nsp12-nsp7-nsp8 required for RdRp activity.	Subissi, L. (2014); Kirchdoerfer, R. et al. (2019); Chen, J. (2020)
nsp9	7SL	Inhibitor	nsp8 and 9 bind and inhibit 7SL	Banerjee, A. (2020)
nsp9	NKRF	Inhibitor	Nsp9/10 stimulate IL6/8 production through inhibition of Nsp9/10	Li, J. et al. (2021)
OAS	RNAseL	Activator	OASs activate RNAseL	Schoggins, J. et al. (2011)
p105	NF-kB	Activator	p105 is a precursor protein of NF-kB	Hayden, M. et al. (2014)
p38	Caspase-3	Activator	Ire1 activation as a result of ER stress can cause apoptosis in a p38 and JNK mediated way	Redza-Dutordoir, M. et al. (2016)
p3a	ASC	Activator	p3a promotes TRAF3-mediated ubiquitination of p105 and ASC (TRAF3 binding domain of ORF3a conserved from SARS-COV	Siu, K. et al. (2019); Issa, E. et al. (2020)
p3a	Caspase-8	Activator	p3a induces apoptosis through caspase-8 activation	Ren, Y. et al. (2020)
p3a	ERstress	Activator	ORF3 expression induces expression of genes indicative of ER stress	Zhang, X. et al. (2020)
p3b	IRF3	Inhibitor	p3b inibits IFN expression by preventing IRF3 nuclear localization	Konno, Y. et al. (2020)
p6	IRF3	Inhibitor	p6 prevents IRF3 nuclear translocation	Xia, H. et al. (2020); Lei, X. et al. (2020); Li, J. et al. (2021)
p6	STAT1	Inhibitor	p6 binds Nup98-Rae1 preventing nuclear import of STAT1/2 to inhibit ISG expression	Miorin, L. (2020); Lei, X. et al. (2020); Li, J. et al. (2021)
p6	STAT2	Inhibitor	p6 binds Nup98-Rae1 preventing nuclear import of STAT1/2 to inhibit ISG expression	Miorin, L. (2020); Lei, X. et al. (2020); Li, J. et al. (2021)
p7a	STAT2	Inhibitor	nsp6, nsp13, p7a, p7b inhibit STAT2 phosphorylation	Xia, H. et al. (2020)
p7b	STAT1	Inhibitor	nsp1, nsp6, nsp13, p3a, M p7b inhibit STAT1 phosphorylation	Xia, H. et al. (2020)
p7b	STAT2	Inhibitor	nsp6, nsp13, p7a, p7b inhibit STAT2 phosphorylation	Xia, H. et al. (2020)
p8	IFN-I	Inhibitor	p6, p8 and N inhibit IFN-beta production and NF-kB responsive promoter	Li, J. et al. (2021)
p8	IFN-III	Inhibitor	p6, p8 and N inhibit IFN-beta production and NF-kB responsive promoter, IFN-III has similar promoter to IFN-I	Wack, A. et al. (2015); Li, J. et al. (2021)
PANoptosome	Caspase-3	Activator	PANoptosome induces apoptosis through caspase-3	Karki, R. et al. (2021)
PANoptosome	GasderminE	Activator	PANoptosome induces pyroptosis through cleavage of Gasdermin E	Karki, R. et al. (2021)
PANoptosome	MLKL	Activator	PANoptosome induces necroptosis through MLKL	Karki, R. et al. (2021)
PERK	eIF2	Inhibitor	phosphorylation of eIF2 prevents activation by its GEF eIF2B	Sonenberg, N. et al. (2009)
PI3K	Akt	Activator	PI3K phosphorylates Akt	Song, G. et al. (2005)
PIKfyve	TPC2	Activator	Production of PI(3,5)P2, mediated by TPC2, contributes to viral entry	Ou, X. (2020)
PKC	ERK	Activator	ERK1/2 activated by AT1R through PKC and EGFR	Forrester, S. (2018)
PKC	ROCK	Activator	PKC activity required for AngII/AT1R induced RhoA/ROCK activity	Forrester, S. (2018)
PKR	Caspase-8	Activator	PKR can induce apoptosis through the FADD/caspase8/caspase3 pathway	Gal-Ben-Ari, S. et al. (2018)
PKR	eIF2	Inhibitor	phosphorylation of eIF2 prevents activation by its GEF eIF2B	Sonenberg, N. et al. (2009)
PKR	IKK	Activator	PKR inhibitrs IkBa through direct phosphorylation	Gal-Ben-Ari, S. et al. (2018); Bonnet, M. et al. (2006)
PLpro	IRF3	Inhibitor	nsp3 cleaves ISG15-modified proteins, leading to lower TBK1 phosphorylation	Shin, D. (2020)
PLpro	nsp1	Activator	nsp1-4 is cleaved into individual proteins by PL pro	Fehr, A. et al. (2015)
PLpro	nsp2	Activator	nsp1-4 is cleaved into individual proteins by PL pro	Fehr, A. et al. (2015)
PLpro	nsp3	Activator	nsp1-4 is cleaved into individual proteins by PL pro	Fehr, A. et al. (2015)
PLpro	nsp4	Activator	nsp1-4 is cleaved into individual proteins by PL pro	Fehr, A. et al. (2015)
PLpro	ViralGenome	Activator	nsp3 important to mediate interaction of N with RTC	Cong, Y. et al. (2020)
PLpro	ViralsgRNAs	Activator	nsp3 important to mediate interaction of N with RTC	Cong, Y. et al. (2020)
pp1a	3CLpro	Activator	3CL protease activity lies in nsp5, uncleaved pp1a and pp1ab also contain this domain	
pp1a	nsp1_nsp2_nsp3_nsp4	Activator	pp1a is cleaved into a single nsp1-4 and then all nsps 5 to 11 by 3CLpro	Fehr, A. et al. (2015)
pp1a	nsp10	Activator	pp1a is cleaved into a single nsp1-4 and then all nsps 5 to 11 by 3CLpro	Fehr, A. et al. (2015)
pp1a	nsp11	Activator	pp1ab is cleaved into a single nsp1-4 and then all nsps 5 to 16 by 3CLpro (nsp11 becomes nsp12 in pp1ab)	Fehr, A. et al. (2015)

From	To	Type	Explanation	Reference
pp1a	nsp5	Activator	pp1a is cleaved into a single nsp1-4 and then all nsps 5 to 11 by 3CLpro	Fehr, A. et al. (2015)
pp1a	nsp6	Activator	pp1a is cleaved into a single nsp1-4 and then all nsps 5 to 11 by 3CLpro	Fehr, A. et al. (2015)
pp1a	nsp7	Activator	pp1a is cleaved into a single nsp1-4 and then all nsps 5 to 11 by 3CLpro	Fehr, A. et al. (2015)
pp1a	nsp8	Activator	pp1a is cleaved into a single nsp1-4 and then all nsps 5 to 11 by 3CLpro	Fehr, A. et al. (2015)
pp1a	nsp9	Activator	pp1a is cleaved into a single nsp1-4 and then all nsps 5 to 11 by 3CLpro	Fehr, A. et al. (2015)
pp1a	PLpro	Activator	PL protease activity lies in nsp3, uncleaved pp1a and pp1ab also contain this domain	
pp1ab	3CLpro	Activator	3CL protease activity lies in nsp5, uncleaved pp1a and pp1ab also contain this domain	
pp1ab	nsp1_nsp2_nsp3_nsp4	Activator	pp1ab is cleaved into a single nsp1-4 and then all nsps 5 to 16 by 3CLpro (nsp11 becomes nsp12 in pp1ab)	Fehr, A. et al. (2015)
pp1ab	nsp10	Activator	pp1ab is cleaved into a single nsp1-4 and then all nsps 5 to 16 by 3CLpro (nsp11 becomes nsp12 in pp1ab)	Fehr, A. et al. (2015)
pp1ab	nsp12	Activator	pp1ab is cleaved into a single nsp1-4 and then all nsps 5 to 16 by 3CLpro (nsp11 becomes nsp12 in pp1ab)	Fehr, A. et al. (2015)
pp1ab	nsp13	Activator	pp1ab is cleaved into a single nsp1-4 and then all nsps 5 to 16 by 3CLpro (nsp11 becomes nsp12 in pp1ab)	Fehr, A. et al. (2015)
pp1ab	nsp14	Activator	pp1ab is cleaved into a single nsp1-4 and then all nsps 5 to 16 by 3CLpro (nsp11 becomes nsp12 in pp1ab)	Fehr, A. et al. (2015)
pp1ab	nsp15	Activator	pp1ab is cleaved into a single nsp1-4 and then all nsps 5 to 16 by 3CLpro (nsp11 becomes nsp12 in pp1ab)	Fehr, A. et al. (2015)
pp1ab	nsp16	Activator	pp1ab is cleaved into a single nsp1-4 and then all nsps 5 to 16 by 3CLpro (nsp11 becomes nsp12 in pp1ab)	Fehr, A. et al. (2015)
pp1ab	nsp5	Activator	pp1ab is cleaved into a single nsp1-4 and then all nsps 5 to 16 by 3CLpro (nsp11 becomes nsp12 in pp1ab)	Fehr, A. et al. (2015)
pp1ab	nsp6	Activator	pp1ab is cleaved into a single nsp1-4 and then all nsps 5 to 16 by 3CLpro (nsp11 becomes nsp12 in pp1ab)	Fehr, A. et al. (2015)
pp1ab	nsp7	Activator	pp1ab is cleaved into a single nsp1-4 and then all nsps 5 to 16 by 3CLpro (nsp11 becomes nsp12 in pp1ab)	Fehr, A. et al. (2015)
pp1ab	nsp8	Activator	pp1ab is cleaved into a single nsp1-4 and then all nsps 5 to 16 by 3CLpro (nsp11 becomes nsp12 in pp1ab)	Fehr, A. et al. (2015)
pp1ab	nsp9	Activator	pp1ab is cleaved into a single nsp1-4 and then all nsps 5 to 16 by 3CLpro (nsp11 becomes nsp12 in pp1ab)	Fehr, A. et al. (2015)
pp1ab	PLpro	Activator	PL protease activity lies in nsp3, uncleaved pp1a and pp1ab also contain this domain	
Pyroptosis	CellDeath	Activator	Cell death may occur via apoptosis, necroptosis or pyroptosis	
RIG-1	CytosolicPRRs	Activator	Cytosolic PRR signalling requires PRRs MDA5 or RIG-1 and adaptors MAVS and STING	Mogensen, T. (2009)
RIPK1	Caspase-10	Activator	Cytosolic PRRs can induce NF-kB through caspase8/10 using RIP1	Mogensen, T. (2009)
RIPK1	Caspase-8	Activator	Cytosolic PRRs can induce NF-kB through caspase8/10 using RIP1	Mogensen, T. (2009)
RIPK1	PANoptosome	Activator	PANoptosome formed from Caspase-8, NLRP3, ASC, RIPK1 as well as RIPK3 and FADD	Christgen, S. et al. (2020)
RIPK1	TLR3-TRIF	Activator	TLR3/4 signalling using TRIF adaptor requires TRAF3,6 E3 ligases and adaptors TANK and RIP1	Mogensen, T. (2009)
RNAseL	ViralGenomeDegradation	Inhibitor	RNAseL destroys RNA	Schoggins, J. et al. (2011)
RNAseL	ViralsgRNAs	Inhibitor	RNAseL destroys RNA	Schoggins, J. et al. (2011)
ROCK	Fibrosis	Activator	AngII induced PF mediated by ROS and ROCK	Kondrikov, D. et al. (2011)
ROS	ADAM17	Activator	ADAM17 activity induced by Nox4-dependent ROS production (possibly phosphorylated)	Forrester, S. et al. (2016)
ROS	EGFR	Activator	AngII/AT1R induce EGFR transactivation through ROS	Forrester, S. (2018)
ROS	JNK	Activator	JNK activity induced by ROS	Redza-Dutordoir, M. et al. (2016)
ROS	ROCK	Activator	AngII induced fibrosis mediated by ROS and ROCK	Kondrikov, D. et al. (2011)
S	ERstress	Activator	Spike protein expression causes UPR ER stess	Chan, C. et al. (2006)
S	SyncytiaFormation	Activator	S protein induces syncytia formation, IFITM (mainly IFITM1) prevents formation but only in absence of TMPRSS2	Buchrieser, J. et al. (2020)
S	VirionERGIC	Activator	Structural proteins form virion in endoplasmic reticulum–Golgi intermediate compartment (ERGIC)	Fehr, A. et al. (2015)
S6K	eIF4B	Activator	eIF4B activated by phosphorylation by S6K1	Magnuson, B. et al. (2012)
SensitivityOfIFNResponse	IFN-I	Activator	Sensitivity of IFN response	
SensitivityOfIFNResponse	IFN-III	Activator	Sensitivity of IFN response	
Splicing	HostmRNAs	Activator	Improperly spliced mRNAs are destroyed by nonsense-mediated mRNA decay, and may not be able to be translated into proper protein.	Kurosaki, T. et al. (2019)
SRP	IFN-I	Activator	SRP required to export IFNs	Banerjee, A. (2020)
SRP	IFN-III	Activator	SRP required to export IFNs	Banerjee, A. (2020)
STAT1	IFN-I_SG	Activator	IFN-I response requires IFNAR, Tyk2, Jak1, STAT1/2 and IRF9	Barrat, F. et al. (2019)
STAT1	IFN-II_SG	Activator	IFN-II response requires IFNGR, Jak2, Jak1, STAT1	Barrat, F. et al. (2019)
STAT1	IFN-III_SG	Activator	IFN-III response requires IFN-LR1, Tyk2, Jak1, STAT1/2 and IRF9	Wack, A. et al. (2015)
STAT2	IFN-I_SG	Activator	IFN-I response requires IFNAR, Tyk2, Jak1, STAT1/2 and IRF9	Barrat, F. et al. (2019)
STAT2	IFN-III_SG	Activator	IFN-III response requires IFN-LR1, Tyk2, Jak1, STAT1/2 and IRF9	Wack, A. et al. (2015)
STING	CytosolicPRRs	Activator	Cytosolic PRR signalling requires PRRs MDA5 or RIG-1 and adaptors MAVS and STING	Mogensen, T. (2009)
TAK1	IKK	Activator	TAK1 activates the IKK complex	Mogensen, T. (2009)
TAK1	JNK	Activator	TAK1 promotes MAPK activity through phosphorylation of MKK proteins	Mogensen, T. (2009)
TAK1	p38	Activator	TAK1 promotes MAPK activity through phosphorylation of MKK proteins	Mogensen, T. (2009)
TANK	TBK1	Activator	Cytosolic PRRs together with TRAF3 and TANK1 can induce IFN through TBK1	Mogensen, T. (2009)
TANK	TLR3-TRIF	Activator	TLR3/4 signalling using TRIF adaptor requires TRAF3,6 E3 ligases and adaptors TANK and RIP1	Mogensen, T. (2009)
TBK1	IRF3	Activator	TBK1 (and redundantly IKKepsilon) phosphorylate IRF3/7	Mogensen, T. (2009)
TBK1	IRF7	Activator	TBK1 (and redundantly IKKepsilon) phosphorylate IRF3/7	Mogensen, T. (2009)
TLR3	TLR3-TRIF	Activator	TLR3/4 signalling using TRIF adaptor requires TRAF3,6 E3 ligases and adaptors TANK and RIP1	Mogensen, T. (2009)
TLR3-TRIF	TAK1	Activator	TLR3/4-TRIF can induce NF-kB through TAK1	Mogensen, T. (2009)
TLR3-TRIF	TBK1	Activator	TLR3/4-TRIF can induce IFN signalling through TBK1	Mogensen, T. (2009)
TMPRSS2	ViralFusion	Activator	Fusion of viral and cellular membranes requires proteolytic cleavage of the spike.	Fehr, A. et al. (2015); Shang, J. et al. (2020)
TMPRSS2	SyncytiaFormation	Activator	S protein induces syncytia formation, IFITM (mainly IFITM1) prevents formation but only in absence of TMPRSS2	Buchrieser, J. et al. (2020)
TNF-a	CCL2	Activator	CXCL9, CXCL10, CXCL11 and CCL2 are induced in lung cells by TNF-a, IL-1B and IFN-g	Pechkovsky, D. et al. (2005)
TNF-a	CXCL10	Activator	CXCL9, CXCL10, CXCL11 and CCL2 are induced in lung cells by TNF-a, IL-1B and IFN-g	Pechkovsky, D. et al. (2005)
TNF-a	CXCL11	Activator	CXCL9, CXCL10, CXCL11 and CCL2 are induced in lung cells by TNF-a, IL-1B and IFN-g	Pechkovsky, D. et al. (2005)
TNF-a	CXCL9	Activator	CXCL9, CXCL10, CXCL11 and CCL2 are induced in lung cells by TNF-a, IL-1B and IFN-g	Pechkovsky, D. et al. (2005)
TNF-a	Inflammation	Activator	pro-inflammatory cytokine	Hamid, Q. et al. (2009)
TNF-a	iNOS	Activator	TNF-a and IFN-II induce production of iNOS (NOS2)	Karki, R. et al. (2021)
TNF-a	T-cellInfiltration	Activator	Speculative interaction based on inverse correlation	Chen, Z. et al. (2020); Diao, B. (2020); Liu, J. (2020)
TPC2	ViralFusion	Activator	TPC2 contributes to viral entry	Ou, X. (2020)
TRAF3	ASC	Activator	p3a promotes TRAF3-mediated ubiquitination of p105 and ASC (TRAF3 binding domain of ORF3a conserved from SARS-COV	Siu, K. et al. (2019); Issa, E. et al. (2020)
TRAF3	p105	Activator	p3a promotes TRAF3-mediated ubiquitination of p105 and ASC (TRAF3 binding domain of ORF3a conserved from SARS-COV	Siu, K. et al. (2019); Issa, E. et al. (2020)
TRAF3	TBK1	Activator	Cytosolic PRRs together with TRAF3 and TANK1 can induce IFN through TBK1	Mogensen, T. (2009)
TRAF3	TLR3-TRIF	Activator	TLR3/4 signalling using TRIF adaptor requires TRAF3,6 E3 ligases and adaptors TANK and RIP1	Mogensen, T. (2009)
TRAF6	TLR3-TRIF	Activator	TLR3/4 signalling using TRIF adaptor requires TRAF3,6 E3 ligases and adaptors TANK and RIP1	Mogensen, T. (2009)
TRIF	TLR3-TRIF	Activator	TLR3/4 signalling using TRIF adaptor requires TRAF3,6 E3 ligases and adaptors TANK and RIP1	Mogensen, T. (2009)
Tyk2	IFN-I_SG	Activator	IFN-I response requires IFNAR, Tyk2, Jak1, STAT1/2 and IRF9	Barrat, F. et al. (2019)

From	To	Type	Explanation	Reference
Tyk2	IFN-III_SG	Activator	IFN-III response requires IFN-LR1, Tyk2, Jak1, STAT1/2 and IRF9	Wack, A. et al. (2015)
U1_U2snRNAs	Splicing	Activator	U1, U2 snRNAs are constituent parts of spliceosome.	Matera, A. et al. (2014)
ViralCap-dependentTranslation	E	Activator	structural proteins translated from viral sgRNAs via cap-dependent translation	Nakagawa, K. et al. (2016)
ViralCap-dependentTranslation	M	Activator	structural proteins translated from viral sgRNAs via cap-dependent translation	Nakagawa, K. et al. (2016)
ViralCap-dependentTranslation	N	Activator	structural proteins translated from viral sgRNAs via cap-dependent translation	Nakagawa, K. et al. (2016)
ViralCap-dependentTranslation	p3a	Activator	protein detected	Davidson, A. (2020); Bouhaddou, M. (2020); Kim, D. et al. (2020)
ViralCap-dependentTranslation	p3b	Activator	protein detected	Davidson, A. (2020)
ViralCap-dependentTranslation	p6	Activator	protein detected	Davidson, A. (2020); Kim, D. et al. (2020)
ViralCap-dependentTranslation	p7a	Activator	protein detected	Davidson, A. (2020); Kim, D. et al. (2020)
ViralCap-dependentTranslation	p7b	Activator	protein detected	Davidson, A. (2020)
ViralCap-dependentTranslation	p8	Activator	protein detected	Davidson, A. (2020); Bouhaddou, M. (2020); Kim, D. et al. (2020)
ViralCap-dependentTranslation	p9b	Activator	protein detected	Bouhaddou, M. (2020); Davidson, A. (2020)
ViralCap-dependentTranslation	p9c	Activator	protein detected	Bouhaddou, M. (2020)
ViralCap-dependentTranslation	pp1a	Activator	pp1a and pp1ab translated directly from viral genome via cap-dependent translation	Nakagawa, K. et al. (2016)
ViralCap-dependentTranslation	pp1ab	Activator	pp1a and pp1ab translated directly from viral genome via cap-dependent translation	Nakagawa, K. et al. (2016)
ViralCap-dependentTranslation	S	Activator	structural proteins translated from viral sgRNAs via cap-dependent translation	Nakagawa, K. et al. (2016)
ViralEntry1	N	Activator	Viral genome enters cell packaged in N protein	
ViralEntry1	ViralGenome	Activator	Fusion of viral and cellular membranes allows entry of viral proteins and RNA into the cell.	Fehr, A. et al. (2015)
ViralFusion	ViralEntry	Activator	Fusion of viral and cellular membranes allows entry of viral proteins and RNA into the cell.	Fehr, A. et al. (2015); Ou, X. (2020)
ViralFusion	ViralEntry1	Activator	Fusion of viral and cellular membranes allows entry of viral proteins and RNA into the cell.	Fehr, A. et al. (2015); Ou, X. (2020)
ViralGenome	CytosolicPRRs	Activator	MDA5/RIG-1 act as PRRs that detects dsRNA eg from viral RNA replication	Lee, S. et al. (2020)
ViralGenome	OAS	Activator	OASs recognise dsRNA	Schoggins, J. et al. (2011)
ViralGenome	PKR	Activator	PKR is activated by dsRNA	Sa Ribero, M. et al. (2020)
ViralGenome	pp1a	Activator	pp1a and pp1ab translated directly from viral genome via cap-dependent translation	Nakagawa, K. et al. (2016)
ViralGenome	pp1ab	Activator	pp1a and pp1ab translated directly from viral genome via cap-dependent translation	Nakagawa, K. et al. (2016)
ViralGenome	TLR3	Activator	TLR3 is activated by dsRNA (in endosome)	Mogensen, T. (2009)
ViralGenome	ViralGenome	Activator	Viral Genome replicated	Nakagawa, K. et al. (2016)
ViralGenome	ViralsgRNAs	Activator	Viral sgRNAs transcribed by RTC from genome	Romano, M. et al. (2020); Kim, D. et al. (2020)
ViralGenome	VirionERGIC	Activator	Structural proteins form virion in endoplasmic reticulum–Golgi intermediate compartment (ERGIC)	Fehr, A. et al. (2015)
ViralGenomeDegradation	VirionERGIC	Inhibitor	Structural proteins form virion in endoplasmic reticulum–Golgi intermediate compartment (ERGIC)	Fehr, A. et al. (2015)
ViralsgRNAs	CytosolicPRRs	Activator	nsp15 EndoU activity cleaves polyUs from viral sgRNAs, preventing their recognition by MDA5.	Hackbart, M. et al. (2020)
ViralsgRNAs	E	Activator	structural proteins translated from viral sgRNAs via cap-dependent translation	Nakagawa, K. et al. (2016)
ViralsgRNAs	M	Activator	structural proteins translated from viral sgRNAs via cap-dependent translation	Nakagawa, K. et al. (2016)
ViralsgRNAs	N	Activator	structural proteins translated from viral sgRNAs via cap-dependent translation	Nakagawa, K. et al. (2016)
ViralsgRNAs	p3a	Activator	protein detected	Davidson, A. (2020); Bouhaddou, M. (2020); Kim, D. et al. (2020)
ViralsgRNAs	p3b	Activator	protein detected	Davidson, A. (2020)
ViralsgRNAs	p6	Activator	protein detected	Davidson, A. (2020); Kim, D. et al. (2020)
ViralsgRNAs	p7a	Activator	protein detected	Davidson, A. (2020); Kim, D. et al. (2020)
ViralsgRNAs	p7b	Activator	protein detected	Davidson, A. (2020)
ViralsgRNAs	p8	Activator	protein detected	Davidson, A. (2020); Bouhaddou, M. (2020); Kim, D. et al. (2020)
ViralsgRNAs	p9b	Activator	protein detected	Bouhaddou, M. (2020); Davidson, A. (2020)
ViralsgRNAs	p9c	Activator	protein detected	Bouhaddou, M. (2020)
ViralsgRNAs	S	Activator	structural proteins translated from viral sgRNAs via cap-dependent translation	Nakagawa, K. et al. (2016)
Virion	ACE2-Spike	Activator	The virus enters the cell through interaction of the spike protein with ACE2, spike complexes coat the outside of the virion.	Chen, Y. et al. (2020); Yao, H. (2020)
VirionERGIC	ViralReplication	Activator	Virions in the ERGIC are tranpsorted to the cell surface and released through exocytosis	Fehr, A. et al. (2015)
XBP1	NF-kB	Activator	Xbp1 induces NF-kB expression	Pathinayake, P. et al. (2018)
ZAP	ViralGenomeDegradation	Activator	ISG ZAP restricts viral RNA	Nchioua, R. et al. (2020)
ZAP	ViralsgRNAs	Inhibitor	ISG ZAP restricts viral RNA	Nchioua, R. et al. (2020)

Supplementary Table 1. Regulatory edges in the network model. Each row shows a single edge, originating from the node in the “from” column and directed to the node in the “to” column. The table also contains details of the type of edge, a brief explanation of the mechanism of this regulation and a reference to the literature where required.

Supplementary Table 2. Target functions used in the network model

Node	Target Function	Explanation	Reference
3CLpro	max(var(pp1a),var(pp1ab)) + var(nsp5)	3CL protease activity lies in nsp5, uncleaved pp1a and pp1ab also contain this domain	Fehr, A. et al. (2015)
40S	2		
43S	min(var(40S),var(eIF2))	43S ribosomal subunit assembled from 40S subunit and multiple eIFs, including eIF2-GTP	Sonenberg, N. et al. (2009)
4E-BP	generic		
60S	1		
7SL	1-(max(var(nsp8),var(nsp9)))	7SL bound by nsp8 and nsp9	Banerjee, A. (2020)
ACE1	1		
ACE2	2-min(1,var(ADAM17))	ACE2 shedding caused by ADAM17	Ingraham, N. (2020); Haga, S. (2008)
ACE2-Spike	var(ACE2)*var(Virion)	Extracellular virions bind to ACE2 forming an ACE2-spike complex.	
ADAM17	1+var(ROS)	ADAM17 activity increased by Nox4-dependent ROS production	Forrester, S. et al. (2016)
Akt	generic		
Ang1-7	(1+var(ACE2)-max(1,var(ACE2-Spike)))*min(var(AngII),1)	Interaction of ACE2 with spike prevents ACE2 enzymatic activity, converting AngII into Ang1-7	Alexandre, J. et al. (2020); Wang, S. et al. (2008)
AngII	generic		
Apoptosis	generic		
ASC	1+min(var(p3a),var(TRAF3))	p3a promotes TRAF3-mediated ubiquitination of ASC (TRAF3 binding domain of ORF3a conserved from SARS-COV)	Siu, K. et al. (2019); Issa, E. et al. (2020)
AT1R	generic		
ATF6	generic		
Cap-independentTranslation	var(40S)*var(60S)-min(1,var(nsp1))	Cap-independent translation relies on 40S and 60S ribosome subunits, nsp1 can inhibit host translation through interaction with 40S	Walters, B. et al. (2016); Banerjee, A. (2020); Thoms, M. (2020); Schubert, K. et al. (2020)
Caspase-1	generic		
Caspase-10	generic		
Caspase-3	var(PANoptosome)+var(Caspase-8)+var(JNK)+var(p38) -var(NF-kB)-var(Akt)	Caspase-3 is the effector of apoptosis and integrates many signals	Redza-Dutordoir, M. et al. (2016); Song, G. et al. (2005); Lee, S. et al. (2020); Karki, R. et al. (2021)
Caspase-8	max(var(CytosolicPRRs), max(var(NO), max(var(p3a),var(PKR))))*var(RIPK1)	Caspase-8 is involved with multiple forms of cell death and can respond to multiple signals, dependent on RIP1	Mogensen, T. (2009); Gal-Ben-Ari, S. et al. (2018); Ren, Y. et al. (2020); Karki, R. et al. (2021)
CathepsinL	1		
CCL2	generic		
CellDeath	max(var(Apoptosis),max(var(Necroptosis),var(Pyroptosis)))	Cell death can occur via apoptosis, pyroptosis or necroptosis	
CH25H	floor((1+2*var(IFN-I_SG))*(1+2*var(IFN-III_SG))/3)	CH25H is an type-I/III ISG	Zang, R. et al. (2020)
CXCL10	generic		
CXCL11	generic		
CXCL9	generic		
CytosolicPRRs	min(var(MAVS), min(var(MDA5), min(var(RIG-1), var(STING))))*(max(var(ViralGenome),var(ViralsgRNAs))-var(nsp15)/2)	MDA5/RIG-1 act as PRRs that detects dsRNA eg from viral RNA replication, nsp15 endonuclease helps viral sgRNAs to evade detection	Lee, S. et al. (2020); Mogensen, T. (2009); Hackbart, M. et al. (2020)
DMVformation	(min(var(nsp3),var(nsp4))+var(nsp6))/2	Double Membrane Vesicles are formed by nsp3, nsp4 and nsp6, these DMVs contain a pore consiting of a hexameric nsp3 complex. Precise requirements determined by Angelini et al.	Angelini, M. et al. (2013); Wolff, G. (2020)
E	var(ViralsgRNAs)*var(ViralCap-dependentTranslation)	structural proteins translated from viral sgRNAs via cap-dependent translation	Nakagawa, K. et al. (2016)
EGFR	min(var(ADAM17),var(ROS))	ADAM17 required for EGFR transactivation by ROS	Forrester, S. (2018)
eIF2	2-min(1,max(var(PERK),var(PKR)))	phosphorylation of eIF2 prevents activation by its GEF eIF2B	Sonenberg, N. et al. (2009)
eIF4A	generic		
eIF4B	generic		
eIF4E	2-var(4E-BP)	4E-BP binds eIF4E, preventing it from associating with eIF4F	Sonenberg, N. et al. (2009)
eIF4F	min(var(eIF4A),min(var(eIF4E),var(eIF4G)))	eIF4F complex fromed of eIF4A, E, G and others	Sonenberg, N. et al. (2009)
eIF4G	generic		
EndosomalpH	2		
ERK	generic		
ERstress	max(var(S),max(var(EGFR),var(p3a)))	ER stress can be induced by S or p3a, or through EGFR transactivation	Forrester, S. (2018); Zhang, X. et al. (2020); Hsu, A. et al. (2020)
Fibrosis	generic		
furin	1		
GasderminD	generic		
GasderminE	generic		
glycosylation	1		
guanylyltransferase	2		
HostCap-dependentTranslation	min(var(43S),min(var(60S),var(eIF4F)))-min(1,var(nsp1))	Cap-dependent translation requires 43S, 60S and eIF4F, nsp1 causes preferential translation of viral RNA	Sonenberg, N. et al. (2009); Banerjee, A. (2020); Thoms, M. (2020); Schubert, K. et al. (2020)
HostmRNAs	generic		
HostProteinSynthesis	max(var(Cap-independentTranslation),var(HostCap-dependentTranslation))*var(HostmRNAs)/2	Host protein synthesis depends on host mRNA and can proceed via cap-dependent or independent mechanisms	Walters, B. et al. (2016)
IFITM	floor((1+2*var(IFN-I_SG))*(1+2*var(IFN-III_SG))/3)	IFITM1-3 are ISGs.	Ribero, M. et al. (2020)
IFN-I	avg(var(IRF3),var(IRF7))*(1+var(SRP))*var(HostProteinSynthesis)*(1+var(SensitivityOfIFNResponse))/5-avg(var(N),var(p8))/2	IRF3 and IRF7 can induce IFN-I synthesis, SRP required for release, p8 and N can inhibit production	Sa Ribero, M. et al. (2020); Barrat, F. et al. (2019); Li, J. et al. (2021)
IFN-I_SG	var(IRF9)*var(Jak1)*min(var(STAT1),var(STAT2))*var(Tyk2)*var(IFNAR)*var(HostProteinSynthesis)/80	IFN-I response requires IFNAR, Tyk2, Jak1, STAT1/2 and IRF9	Barrat, F. et al. (2019)
IFN-II	var(ImmuneCells)*var(IL-18)/2	IFN-gamma produced by various immune cells in response to IL-12 or IL-18	Ivashkiv, L. (2018)
IFN-II_SG	var(Jak1)*var(Jak2)*var(STAT1)*var(IFNGR)*var(HostProteinSynthesis)/80	IFN-II response requires IFNGR, Jak2, Jak1, STAT1	Barrat, F. et al. (2019)
IFN-III	avg(var(IRF3),var(IRF7))*(1+var(SRP))*var(HostProteinSynthesis)*(1+var(SensitivityOfIFNResponse))/5-avg(var(N),var(p8))/2	IRF3 and IRF7 can induce IFN-I synthesis, SRP required for release, p8 and N can inhibit production	Sa Ribero, M. et al. (2020); Barrat, F. et al. (2019); Li, J. et al. (2021)
IFN-III_SG	var(IRF9)*var(Jak1)*min(var(STAT1),var(STAT2))*var(Tyk2)*var(IFN-LR1)*var(HostProteinSynthesis)/80	IFN-III response requires IFN-LR1, Tyk2, Jak1, STAT1/2 and IRF9	Wack, A. et al. (2015)
IFN-LR1	generic		
IFNAR	generic		
IFNGR	generic		
IkBa	2-var(IKK)	IKK inhibits IkBa through phosphorylation	Liu, T. et al. (2017); Gal-Ben-Ari, S. et al. (2018)
IKK	generic		
IL-10	generic		
IL-18	min(var(Caspase-1),var(NF-kB))	Pro-IL-18 production driven by NF-kB, cleaved into IL-18 by caspase-1	Man, S. et al. (2017); Liu, T. et al. (2017)
IL-1B	max(1,var(Caspase-1))*var(NF-kB)/2	Pro-IL-1B production driven by NF-kB, cleaved into IL-18 by caspase-1, assume cleavage possible even at low levels of caspase activity	Man, S. et al. (2017); Liu, T. et al. (2017)
IL-2	var(ImmuneCells)*var(NF-kB)/2	IL-2 produced by immune cells in response to NF-kB	Liao, W. et al. (2013)
IL-6	generic		
IL-8	generic		
ImmuneCells	input		
Inflammasome	(var(ASC)*var(NLRP3))-1	ASC, NLRP3 and pro-caspase-1 form inflammasome leading to activation of Caspase-1	Lee, S. et al. (2020)
Inflammation	(var(IL-1B)+var(IL-6)+var(IL-8)+var(TNF-a))/4-(var(IL-10)+var(IFN-II))/4	Inflammation is result of integration of several pro- and anti-inflammatory cytokines	Hamid, Q. et al. (2009)
iNOS	min(var(HostProteinSynthesis),min(var(IFN-II_SG),var(TNF-a)))	TNF-a and IFN-II induce production of iNOS (NOS2)	Karki, R. et al. (2021)
IRE1	generic		
IRF3	var(TBK1)-max(var(nsp13),max(var(nsp14),max(var(nsp15),max(var(p3b),max(var(p6),max(var(PLpro),var(nsp6)))))))/2	IRF3 activity driven by TBK1 and inhibited by many viral proteins	Mogensen, T. (2009); Yuen, C. et al. (2020); Xia, H. et al. (2020); Konno, Y. et al. (2020); Shin, D. (2020); Xia, H. et al. (2020)
IRF7	generic		
IRF9	1		
Jak1	1		
Jak2	1		
JNK	generic		
LY6E	floor((1+2*var(IFN-I_SG))*(1+2*var(IFN-III_SG))/3)	LY6E is an ISG	Sa Ribero, M. et al. (2020)
M	var(ViralsgRNAs)*var(ViralCap-dependentTranslation)	structural proteins translated from viral sgRNAs via cap-dependent translation	Nakagawa, K. et al. (2016)
MasR	generic		
MAVS	1		
MDA5	1		
MLKL	generic		
mTORC1	generic		
N	var(ViralEntry1) + var(ViralsgRNAs)*var(ViralCap-dependentTranslation)	structural proteins translated from viral sgRNAs via cap-dependent translation	Nakagawa, K. et al. (2016)
Necroptosis	generic		
NF-kB	var(p105)*(max(1,var(XBP1)))*(1+var(IFN-I_SG)/2)/4-var(IkBa)/2	p105 is a constituent protein of the NF-kB complex, NF-kB activity is stimulated by XBP1 and the IFN-I pathway and is regulated by its inhibitor IkBa	Liu, T. et al. (2017); Hayden, M. et al. (2014); Pathinayake, P. et al. (2018); Goritzka, M. et al. (2014)
NKRF	generic		
NLRP3	1		
NO	generic		
Nox4	generic		
nsp1	var(nsp1_nsp2_nsp3_nsp4)*var(PLpro)	nsp1-4 is cleaved into individual proteins by PLpro	Fehr, A. et al. (2015)
nsp1_nsp2_nsp3_nsp4	max(var(pp1a),var(pp1ab))*var(3CLpro)	pp1a is cleaved into a single nsp1-4 and then all nsps 5 to 11 by 3CLpro, pp1ab is cleaved into a single nsp1-4 and then all nsps 5 to 16 by 3CLpro (nsp11 becomes nsp12 in pp1ab)	Fehr, A. et al. (2015)
nsp10	max(var(pp1a),var(pp1ab))*var(3CLpro)	pp1a is cleaved into a single nsp1-4 and then all nsps 5 to 11 by 3CLpro, pp1ab is cleaved into a single nsp1-4 and then all nsps 5 to 16 by 3CLpro (nsp11 becomes nsp12 in pp1ab)	Fehr, A. et al. (2015)
nsp10-nsp14	generic		
nsp10-nsp16	min(var(nsp10),var(nsp16))	nsp10 and nsp16 form a complex	Bouvet, M. et al. (2010); Viswanathan, T. (2020)

Node	Target Function	Explanation	Reference
nsp11	var(pp1a)*var(3CLpro)	pp1a is cleaved into a single nsp1-4 and then all nsps 5 to 11 by 3CLpro, pp1ab is cleaved into a single nsp1-4 and then all nsps 5 to 16 by 3CLpro (nsp11 becomes nsp12 in pp1ab)	Fehr, A. et al. (2015)
nsp12	var(pp1ab)*var(3CLpro)	pp1a is cleaved into a single nsp1-4 and then all nsps 5 to 11 by 3CLpro, pp1ab is cleaved into a single nsp1-4 and then all nsps 5 to 16 by 3CLpro (nsp11 becomes nsp12 in pp1ab)	Fehr, A. et al. (2015)
nsp12-nsp7-nsp8	min(var(nsp12),min(var(nsp7),var(nsp8)))	Complex formed of nsp12-nsp7-nsp8 required for RdRp activity.	Subissi, L. (2014); Kirchdoerfer, R. et al. (2019); Chen, J. (2020)
nsp13	var(pp1ab)*var(3CLpro)	pp1a is cleaved into a single nsp1-4 and then all nsps 5 to 11 by 3CLpro, pp1ab is cleaved into a single nsp1-4 and then all nsps 5 to 16 by 3CLpro (nsp11 becomes nsp12 in pp1ab)	Fehr, A. et al. (2015)
nsp14	var(pp1ab)*var(3CLpro)	pp1a is cleaved into a single nsp1-4 and then all nsps 5 to 11 by 3CLpro, pp1ab is cleaved into a single nsp1-4 and then all nsps 5 to 16 by 3CLpro (nsp11 becomes nsp12 in pp1ab)	Fehr, A. et al. (2015)
nsp15	var(pp1ab)*var(3CLpro)	pp1a is cleaved into a single nsp1-4 and then all nsps 5 to 11 by 3CLpro, pp1ab is cleaved into a single nsp1-4 and then all nsps 5 to 16 by 3CLpro (nsp11 becomes nsp12 in pp1ab)	Fehr, A. et al. (2015)
nsp16	var(pp1ab)*var(3CLpro)	pp1a is cleaved into a single nsp1-4 and then all nsps 5 to 11 by 3CLpro, pp1ab is cleaved into a single nsp1-4 and then all nsps 5 to 16 by 3CLpro (nsp11 becomes nsp12 in pp1ab)	Fehr, A. et al. (2015)
nsp2	var(nsp1_nsp2_nsp3_nsp4)*var(PLpro)	nsp1-4 is cleaved into individual proteins by PLpro	Fehr, A. et al. (2015)
nsp3	var(nsp1_nsp2_nsp3_nsp4)*var(PLpro)	nsp1-4 is cleaved into individual proteins by PLpro	Fehr, A. et al. (2015)
nsp4	var(nsp1_nsp2_nsp3_nsp4)*var(PLpro)	nsp1-4 is cleaved into individual proteins by PLpro	Fehr, A. et al. (2015)
nsp5	max(var(pp1a),var(pp1ab))*var(3CLpro)	pp1a is cleaved into a single nsp1-4 and then all nsps 5 to 11 by 3CLpro, pp1ab is cleaved into a single nsp1-4 and then all nsps 5 to 16 by 3CLpro (nsp11 becomes nsp12 in pp1ab)	Fehr, A. et al. (2015)
nsp6	max(var(pp1a),var(pp1ab))*var(3CLpro)	pp1a is cleaved into a single nsp1-4 and then all nsps 5 to 11 by 3CLpro, pp1ab is cleaved into a single nsp1-4 and then all nsps 5 to 16 by 3CLpro (nsp11 becomes nsp12 in pp1ab)	Fehr, A. et al. (2015)
nsp7	max(var(pp1a),var(pp1ab))*var(3CLpro)	pp1a is cleaved into a single nsp1-4 and then all nsps 5 to 11 by 3CLpro, pp1ab is cleaved into a single nsp1-4 and then all nsps 5 to 16 by 3CLpro (nsp11 becomes nsp12 in pp1ab)	Fehr, A. et al. (2015)
nsp8	generic		
nsp9	generic		
OAS	floor((1+2*var(IFN-I_SG))*(1+2*var(IFN-III_SG))/3)*min(var(ViralGenome),1)	OAS is an ISG, activated by dsRNA	Schoggins, J. et al. (2011)
p105	generic		
p38	max(var(TAK1),var(AT1R)-var(MasR))	AT1R and MasR have opposing effects on p38 activation, TAK1 promotes MAPK activity through phosphorylation of MKK proteins	Forrester, S. (2018); Mogensen, T. (2009)
p3a	min(var(ViralsgRNAs),var(ViralCap-dependentTranslation))	accessory proteins translated from viral sgRNAs via cap-dependent translation	Nakagawa, K. et al. (2016)
p3b	min(var(ViralsgRNAs),var(ViralCap-dependentTranslation))	accessory proteins translated from viral sgRNAs via cap-dependent translation	Nakagawa, K. et al. (2016)
p6	min(var(ViralsgRNAs),var(ViralCap-dependentTranslation))	accessory proteins translated from viral sgRNAs via cap-dependent translation	Nakagawa, K. et al. (2016)
p7a	min(var(ViralsgRNAs),var(ViralCap-dependentTranslation))	accessory proteins translated from viral sgRNAs via cap-dependent translation	Nakagawa, K. et al. (2016)
p7b	min(var(ViralsgRNAs),var(ViralCap-dependentTranslation))	accessory proteins translated from viral sgRNAs via cap-dependent translation	Nakagawa, K. et al. (2016)
p8	min(var(ViralsgRNAs),var(ViralCap-dependentTranslation))	accessory proteins translated from viral sgRNAs via cap-dependent translation	Nakagawa, K. et al. (2016)
p9b	min(var(ViralsgRNAs),var(ViralCap-dependentTranslation))	accessory proteins translated from viral sgRNAs via cap-dependent translation	Nakagawa, K. et al. (2016)
p9c	min(var(ViralsgRNAs),var(ViralCap-dependentTranslation))	accessory proteins translated from viral sgRNAs via cap-dependent translation	Nakagawa, K. et al. (2016)
PANoptosome	var(ASC)*var(Caspase-8)*var(NLRP3)*var(RIPK1)	PANoptosome formed from Caspase-8, NLRP3, ASC, RIPK1 as well as RIPK3 and FADD	Christgen, S. et al. (2020)
PERK	min(1,var(ERstress))	AngII/AT1R induces PERK activity through ER stress	Forrester, S. (2018)
PI3K	generic		
PIKfyve	1		
PKC	generic		
PKR	floor((1+2*var(IFN-I_SG))*(1+2*var(IFN-III_SG))/3)*min(var(ViralGenome),1)	PKR is an ISG and is activated by dsRNA	Sa Ribero, M. et al. (2020)
PLpro	max(var(pp1a),var(pp1ab)) + var(nsp3)	PL protease activity lies in nsp3, uncleaved pp1a and pp1ab also contain this domain	Fehr, A. et al. (2015)
pp1a	min(var(ViralGenome),var(ViralCap-dependentTranslation))	pp1a and pp1ab translated directly from viral genome via cap-dependent translation	Nakagawa, K. et al. (2016)
pp1ab	min(var(ViralGenome),var(ViralCap-dependentTranslation))	pp1a and pp1ab translated directly from viral genome via cap-dependent translation	Nakagawa, K. et al. (2016)
Pyroptosis	var(GasderminE)+var(GasderminD)	Caspase-1 drives pyroptosis through cleavage of Gasdermin D, PANoptosome induces pyroptosis through cleavage of Gasdermin E	Man, S. et al. (2017)
RIG-1	1		
RIPK1	1		
RNAseL	generic		
ROCK	min(var(PKC),var(ROS))	AngII induced fibrosis mediated by ROS and ROCK, PKC activity required for AngII/AT1R induced RhoA/ROCK activity	Forrester, S. (2018); Kondrikov, D. et al. (2011)
ROS	generic		
S	var(ViralsgRNAs)*var(ViralCap-dependentTranslation)*var(furin)*var(glycosylation)	structural proteins translated from viral sgRNAs via cap-dependent translation, S processed by furin cleavage and glycosylation	Nakagawa, K. et al. (2016); Yang, Q. et al. (2020); Shang, J. et al. (2020)
S6K	generic		
SensitivityOfIFNResponse	input		
Splicing	generic		
SRP	generic		
STAT1	2-max(var(p6),max(var(M),max(var(nsp1),max(var(nsp13),max(var(nsp6),var(p7b))))))/2	nsp1, nsp6, nsp13, p3a, M, p7b and p6 inhibit STAT1 phosphorylation or nuclear localization	Xia, H. et al. (2020); Miorin, L. (2020); Lei, X. et al. (2020); Li, J. et al. (2021)
STAT2	2-max(var(p6),max(var(nsp13),max(var(nsp6),max(var(p7a),var(p7b)))))/2	nsp6, nsp13, p7a, p7b and p6 inhibit STAT2 phosphorylation or nuclear localization	Xia, H. et al. (2020); Miorin, L. (2020); Lei, X. et al. (2020); Li, J. et al. (2021)
STING	1		
SyncytiaFormation	min(1,var(S))*(1+min(1,var(TMPRSS2))) - var(IFITM)	S protein induces syncytia formation, IFITM (mainly IFITM1) prevents formation but only in absence of TMPRSS2	Buchrieser, J. et al. (2020)
T-cellInfiltration	var(IL-10)*var(IL-6)*var(TNF-a)	These cytokines inversely correlated with T-cell numbers	Chen, Z. et al. (2020); Diao, B. (2020); Liu, J. (2020)
TAK1	generic		
TANK	1		
TBK1	avg(var(TRAF3),var(TANK))*(var(CytosolicPRRs)+var(TLR3-TRIF))	Cytosolic PRRs together with TRAF3 and TANK1 or TLR3-TRIF can induce IFN through TBK1	Mogensen, T. (2009)
TLR3	generic		
TLR3-TRIF	min(var(RIPK1), min(var(TANK), min(var(TLR3), min(var(TRAF3), min(var(TRAF6),var(TRIF))))))	TLR3 signalling using TRIF adaptor requires TRAF3,6 E3 ligases and adaptors TANK and RIP1	Mogensen, T. (2009)
TMPRSS2	1		
TNF-a	avg(var(IFN-II),var(IL-1B),var(IL-2),var(NF-kB))*var(ImmuneCells)	TNF-a produced by a range of immune cells in response to many stimuli including IFN-II, IL-1 and IL-2 and NF-kB	Falvo, J. et al. (2010)
TPC2	generic		
TRAF3	1		
TRAF6	1		
TRIF	1		
Tyk2	1		
U1_U2snRNAs	2-min(1,var(nsp16))	nsp16 binds U1/U2 snRNAs inhibiting splicing	Banerjee, A. (2020)
ViralCap-dependentTranslation	min(var(43S),min(var(60S),var(eIF4F)))*max(1,var(nsp1))	Cap-dependent translation requires 43S, 60S and eIF4F, nsp1 causes preferential translation of viral RNA	Sonenberg, N. et al. (2009); Banerjee, A. (2020); Thoms, M. (2020); Schubert, K. et al. (2020)
ViralEntry	var(ViralFusion)-(var(LY6E)+var(CH25H))/2	Viral entry inhibited by LY6E and CH25H	Vabret, N. (2020); Zang, R. et al. (2020); Pfaender, S. (2020); Zhao, X. et al. (2020)
ViralEntry1	var(ViralFusion)	Assume initial entry of virus uninhibited to allow analysis of stable state	
ViralFusion	(min(var(TMPRSS2),var(CathepsinL)) + min(var(CathepsinL),var(TPC2)) + min(var(TMPRSS2),var(TPC2)))*min(1,var(ACE2-Spike))*(1+min(1,var(EndosomalpH)))/4	Successful entry of the virion requires engagement of the virion with ACE2 and 2 of 3 proteases, low endosomal pH required for viral fusion by endocytosis.	Fehr, A. et al. (2015); Shang, J. et al. (2020); Hoffmann, M. (2020); Ou, X. (2020)
ViralGenome	min(1,var(ViralEntry1)) + min(var(ViralEntry1),min(var(DMVformation),min(var(N), min(var(PLpro), min(var(nsp10-nsp14), min(var(nsp12-nsp7-nsp8),min(var(nsp13),var(ViralGenome))))))))	Viral entry introduces viral genome, then replicated using viral RdRp machinery in DMVs (require viral entry to prevent infected states when virion =0)	Fehr, A. et al. (2015); Cong, Y. (2020); Chen, J. (2020); Snijder, E. (2020)
ViralGenomeDegradation	(var(ZAP)+var(RNAseL))/2	Degradation of the viral genome by cellular restriction factors ZAP and RNAseL will prevent spread of virus	Schoggins, J. et al. (2011); Nchioua, R. et al. (2020)
ViralReplication	generic		
ViralsgRNAs	min(var(ViralGenome), min(var(DMVformation), min(2*ceil(var(N)/2), min(var(PLpro), min(var(nsp10-nsp16), min(var(nsp12-nsp7-nsp8), min(var(nsp14), min(var(guanylyltransferase), var(nsp13)))))))))	Viral sgRNAs transcribed by RTC from genome, nsp14 functions as N7-MTase, ensuring viral mRNAs are correctly capped, sgRNAs restricted by ZAP	Romano, M. et al. (2020); Ogando, N. et al. (2020); Nchioua, R. et al. (2020)
Virion	input		
VirionERGIC	min(var(E), min(var(M), min(var(N), min(var(S),(var(ViralGenome)-var(ViralGenomeDegradation))))))	Structural proteins form virion in endoplasmic reticulum–Golgi intermediate compartment (ERGIC)	Fehr, A. et al. (2015)
XBP1	generic		
ZAP	floor((1+2*var(IFN-I_SG))*(1+2*var(IFN-III_SG))*(1+2*var(IFN-II_SG))/3)	ZAP is an ISG	Nchioua, R. et al. (2020)

Supplementary Table 2. Target functions used in the network model. The target function for each node is specified, along with a brief description of the mechanism and a reference where required.

Supplementary Table 3. Constraints for each state of the network model

Background	Node	Activity
Uninfected	Virion	0
	ImmuneCells	1
	Sensitivity of IFN Response	0
Mild COVID-19	Virion	1
	ImmuneCells	1
	Sensitivity of IFN Response	1
Early Stage, Severe COVID-19	Virion	1
	ImmuneCells	1
	Sensitivity of IFN Response	0
Late Stage, Severe COVID-19	Virion	1
	ImmuneCells	1
	Sensitivity of IFN Response	2
	ViralGenome	2

Supplementary Table 3. Constraints applied for each state of the network model. For each model state, the constrained nodes and their value are given.

Supplementary Table 4. Experiments from the literature used to validate the network model

Paper	Cell Line	Experiment	Constraints	Expected Results	Model Result
		Background for all experiments	Immune Cells OFF, Sensitvity of IFN Response Low, all other inputs ON		
Hoffman et al. (2020)	E6 Vero (TMPRSS2+)	Control	Virion OFF	Viral Entry OFF	Viral Entry OFF
		Untreated	Virion ON	Viral Entry High	Viral Entry High
		Cathepsin inhibitor (E-64d)	Virion ON, CathepsinL OFF	Viral Entry Low	Viral Entry Low
		TMPRSS2 inhibitor (Camostat mesylate)	Virion ON, TMPRSS2 OFF	Viral Entry Low	Viral Entry Low
		Camostat mesylate +E-64d	Virion ON, TMPRSS2 OFF, CathepsinL OFF	Viral Entry OFF	Viral Entry OFF
	BHK-21	No entry in ACE2- cell line	Virion ON, ACE2 OFF	Viral Entry OFF	Viral Entry OFF
Ou et al. (2020)	HEK-293T (ACE2+)	Entry in TMPRSS2- cell line	Virion ON, TMPRSS2 OFF	Viral Entry Low	Viral Entry Low
		PIKfyve inhibitor (apilimod)	Virion ON, TMPRSS2 OFF, PIKfyve OFF	Viral Entry OFF	Viral Entry OFF
		TPC2 inhibitor (Tetrandrine)	Virion ON, TMPRSS2 OFF, TPC2 OFF	Viral Entry OFF	Viral Entry OFF
Buchrieser et al. (2020)	U2-OS (ACE2+)	Syncytia assay (TMPRSS2- cells)	Virion ON, TMPRSS2 OFF	Syncytia Formation Low	Syncytia Formation Low
		Syncytia assay (TMPRSS2+ cells)	Virion ON	Syncytia Formation High	Syncytia Formation High
		Syncytia assay (TMPRSS2- cells) + S-protein	Virion OFF, TMPRSS2 OFF, S ON	Syncytia Formation Low	Syncytia Formation Low
		Syncytia assay (TMPRSS2+ cells) + S-protein	Virion OFF, TMPRSS2 ON, S ON	Syncytia Formation High	Syncytia Formation High
		Syncytia assay (TMPRSS2- cells) + S-protein + IFITM1 expression	Virion OFF, TMPRSS2 OFF, S ON, IFITM1 ON	Syncytia Formation OFF	Syncytia Formation OFF
		Syncytia assay (TMPRSS2+ cells) + S-protein + IFITM1 expression	Virion OFF, S ON, IFITM1 ON	Syncytia Formation Low	Syncytia Formation Low
Choy et al. (2020)	E6 Vero	control	Virion ON, TMPRSS2 OFF	Viral Replication High	Viral Replication High
		Redemsivir	Virion ON, TMPRSS2 OFF, nsp12 OFF	Viral Replication OFF	Viral Replication OFF
		Homoharringtonine	Virion ON, TMPRSS2 OFF, 60S OFF	Viral Replication OFF	Viral Replication OFF
		Lopinavir	Virion ON, TMPRSS2 OFF, 3CLpro OFF	Viral Replication OFF	Viral Replication OFF
Wang et al. (2020a)	E6 Vero	Arbidol	Virion ON, TMPRSS2 OFF, ACE2-Spike OFF, S OFF	Viral Replication OFF	Viral Replication OFF
Gordon et al. (2020)	E6 Vero	Zotatifin (eIF4A inhibitor)	Virion ON, TMPRSS2 OFF, eIF4A OFF	Viral Replication OFF	Viral Replication OFF
Wang et al. (2020b)	E6 Vero	Chloroquine	Virion ON, TMPRSS2 OFF, EndosomalpH OFF, TNF-a OFF, IL-6 OFF, glycosylation OFF	Viral Entry OFF	Viral Entry OFF
		Chloroquine post-entry	Virion ON, Viral Entry ON, TMPRSS2 OFF, EndosomalpH OFF, TNF-a OFF, IL-6 OFF, glycosylation OFF	Viral Replication OFF	Viral Replication OFF
Shin et al. (2020)	Caco-2	GRL-0617 (PLpro inhibitor)	Virion ON, PLpro OFF	Viral Replication OFF	Viral Replication OFF
Ren et al. (2020)	E6 Vero	Control	Virion OFF, TMPRSS2 OFF	Apoptosis OFF	Apoptosis OFF
		ORF3a expression	Virion OFF, TMPRSS2 OFF, p3a ON	Apoptosis High	Apoptosis High
Lokugamage et al. (2020)	Calu-3	Infected	Virion ON, Sensitivity of IFN Response Mid	Replication Mid	Replication Mid
		IFN-I treatment	Virion ON, Sensitivity of IFN Response Mid, IFN-I High	Replication OFF	Replication OFF
		IFN-I treatment, post-infection	Virion ON, Sensitivity of IFN Response Mid, IFN-I High, Viral Entry ON	Replication Mid	Replication OFF
	Human airway epithelial cultures	IFN-I treatment, HAEC	Virion ON, Sensitivity of IFN Response Mid, IFN-I High, Immune Cells ON	Replication OFF	Replication Low
Vanderheiden et al. (2020)	Human airway epithelial cultures	Infected	Virion ON, Immune Cells ON	Replication High	Replication High
		IFN-III treatment	Virion ON, Immune Cells ON, IFN-III High	Replication Mid	Replication Low
		IFN-III treatment, post-infection	Virion ON, Immune Cells ON, IFN-III High, Viral Entry ON	Replication Mid	Replication Low
		Transcriptomics Control	Virion OFF, Immune Cells ON	TNF-a OFF, IL-6 OFF, IL-18 OFF, IL-1B OFF, IFN-I OFF, IFN-III OFF, IFITM OFF, OAS OFF	TNF-a OFF, IL-6 OFF, IL-18 OFF, IL-1B OFF, IFN-I OFF, IFN-III OFF, IFITM OFF, OAS OFF
		Transcriptomics Infected	Virion ON, Immune Cells ON	TNF-a Mid, IL-6 Low, IL-18 Low, IL-1B Low, IFN-I OFF, IFN-III OFF, IFITM OFF, OAS OFF	TNF-a Mid, IL-6 Low, IL-18 Low, IL-1B Low, IFN-I OFF, IFN-III OFF, IFITM OFF, OAS OFF
Nchioua et al. (2020)	Calu-3	Infected	Virion ON, Sensitivity of IFN Response Low	Viral Replication Mid, Viral Entry Mid	Viral Replication Mid, Viral Entry Mid
		IFN-II treatment	Virion ON, Sensitivity of IFN Response Low, IFN-II High	Viral Replication Low, Viral Entry Mid	Viral Replication Low, Viral Entry Mid
		IFN-I treatment, ZAP siRNA	Virion ON, Sensitivity of IFN Response Low, IFN-I High, ZAP OFF	Viral Replication OFF, Viral Entry OFF	Viral Replication Mid, Viral Entry OFF
		IFN-II treatment, ZAP siRNA	Virion ON, Sensitivity of IFN Response Low, IFN-II High, ZAP OFF	Viral Replication Mid, Viral Entry Mid	Viral Replication Mid, Viral Entry Mid
Zhang et al. (2020)	A549	Control	Virion OFF	ER Stress OFF	ER Stress OFF
		Infected	Virion ON	ER Stress High	ER Stress High
		ORF3a expression	Virion OFF, p3a ON	ER Stress High, IFN-I Low, IL-6 Low, IL-1B Low, TNF-a Low	ER Stress High, **IFN-I OFF**, IL-6 Low, IL-1B Low, **TNF-a OFF**
Hsu et al. (2020)	BCi-NS1.1	S treatment	Virion OFF, S ON	ER Stress High, IL-6 Low, IL-1B Low, TNF-a Low, CCL2 Low	ER Stress High, IL-6 Low, IL-1B Low, **TNF-a OFF**, CCL2 Low
		S treatment + 4-PBA (ER stress inhibitor)	Virion OFF, S ON, ER Stress OFF	IL-6 OFF, IL-1B OFF, TNF-a OFF, CCL2 OFF	IL-6 OFF, IL-1B OFF, TNF-a OFF, CCL2 OFF
		S treatment + Ulixertinib (ERK inhibitor)	Virion OFF, S ON, ERK OFF	IL-6 OFF, IL-1B OFF, TNF-a OFF, CCL2 OFF	**IL-6 Low, IL-1B Low,** TNF-a OFF**, CCL2 Low**
Miorin et al. (2020)	HEK293T	ISG assay control	Virion OFF, IFN-I High	IFN-I_SG High	IFN-I_SG High
		ISG assay ORF6 expression	Virion OFF, IFN-I High, p6 High	IFN-I_SG Mid	IFN-I_SG Mid
Banerjee et al. (2020)	HEK293T	ISG assay, Nsp1 expression	Virion OFF, IFN-I High, Nsp1 High	IFN-I_SG Mid	IFN-I_SG Mid
		ISG assay, Nsp16 expression	Virion OFF, IFN-I High, Nsp16 High	IFN-I_SG Mid	IFN-I_SG Mid
		ISG assay, Nsp8+Nsp9 expression	Virion OFF, IFN-I High, Nsp8 High, Nsp9 High	IFN-I_SG Mid	**IFN-I_SG High**
Xia et al. (2020)	HEK293T	ISG assay, Nsp6 expression	Virion OFF, IFN-I High, Nsp6 High	IFN-I_SG Mid	IFN-I_SG Mid
		ISG assay, Nsp13 expression	Virion OFF, IFN-I High, Nsp13 High	IFN-I_SG Mid	IFN-I_SG Mid
Uhal et al. (2020)	MLE-12	AngII treatment, uninfected	Virion OFF, AngII High	Apoptosis Low	Apoptosis Low
		ACE2 inhibitor (DX600), uninfected	Virion OFF, ACE2 OFF	Apoptosis Low	Apoptosis Low
Meng et al. (2015)	Rat primary lung fibroblast	Control, collagen assay, uninfected	Virion OFF	Fibrosis OFF	Fibrosis OFF
		AngII treatment, collagen + ROS assay, uninfected	Virion OFF, AngII High	Fibrosis ON, ROS ON	Fibrosis ON, ROS ON
		AngII treatment + Nox4 siRNA, collagen + ROS assay, uninfected	Virion OFF, AngII High, Nox4 OFF	Fibrosis OFF, ROS OFF	Fibrosis OFF, ROS OFF
		AngII treatment + NAC (ROS scavenger), collagen assay, uninfected	Virion OFF, AngII High, ROS OFF	Fibrosis OFF	Fibrosis OFF
		AngII + Ang1-7 treatment, collagen assay, uninfected	Virion OFF, AngII High, Ang1-7 High	Fibrosis OFF	Fibrosis OFF
		AngII + Ang1-7 treatment + A779 (MasR antagonist), collagen assay, uninfected	Virion OFF, AngII High, Ang1-7 High, MasR OFF	Fibrosis ON	Fibrosis ON
		AngII treatment + ACE2 overexpression, collagen, uninfected	Virion OFF, AngII High, ACE2 High	Fibrosis OFF	Fibrosis OFF
Karki et al. (2021)	Bone Marrow Derived Macrophages	Control	Virion OFF	Cell Death OFF	Cell Death OFF
		TNF-a + IFN-II treatment	Virion OFF, TNF-a High, IFN-II High	Cell Death High	Cell Death High
		TNF-a treatment	Virion OFF, TNF-a High	Cell Death OFF	Cell Death OFF
		IFN-II treatment	Virion OFF, IFN-II High	Cell Death OFF	Cell Death OFF
		Cytokine cocktail (IL-6, IL-8, IL-2, IL-1B)	Virion OFF, IL-6 High, IL-8 High, IL-2 High, IL-1B High	Cell Death OFF	Cell Death OFF
		TNF-a + IFN-I treatment	Virion OFF, TNF-a High, IFN-I High	Cell Death OFF	Cell Death OFF
		TNF-a + IFN-III treatment	Virion OFF, TNF-a High, IFN-III High	Cell Death OFF	Cell Death OFF
		Control, PANoptosis assay	Virion OFF	Apoptosis OFF, Pyroptosis OFF, Necroptosis OFF	Apoptosis OFF, Pyroptosis OFF, Necroptosis OFF
		TNF-a + IFN-II treatment, PANoptosis assay	Virion OFF, TNF-a High, IFN-II High	Apoptosis High, Pyroptosis High, Necroptosis High	Apoptosis High, Pyroptosis High, Necroptosis High
		TNF-a + IFN-II treatment, stat1-/-	Virion OFF, TNF-a High, IFN-II High, STAT1 OFF	Cell Death OFF	Cell Death OFF
		TNF-a + IFN-II treatment, nos2-/-	Virion OFF, TNF-a High, IFN-II High, iNOS OFF	Cell Death OFF	Cell Death OFF
		SIN-1 (NO donor) treatment	Virion OFF, NO High	Cell Death High	Cell Death High
Thorne et al. (2021)	Calu-3	Control	Virion OFF, Sensitivity of IFN Response Low	NF-kB OFF, IL-6 OFF, IRF3 OFF, IFITM OFF	NF-kB OFF, IL-6 OFF, IRF3 OFF, IFITM OFF
		Infected	Virion ON, Sensitivity of IFN Response Low	NF-kB High, IL-6 High, IRF3 Low, IFITM Low	NF-kB High, IL-6 High, IRF3 Low, IFITM Low
		Infected, RNA assay	Virion ON, Sensitivity of IFN Response Low	CXCL10 Low, IL-6 High, IFN-I Mid, IFITM Low	CXCL10 Low, IL-6 High, IFN-I Mid, IFITM Low
		Infected + MDA5 siRNA, RNA assay	Virion ON, Sensitivity of IFN Response Low, MDA5 OFF	NF-kB OFF, IL-6 High, IRF3 OFF, IFITM OFF	NF-kB OFF, **IL-6 Low**, IRF3 OFF, IFITM OFF
		Infected + RIG-1 siRNA, RNA assay	Virion ON, Sensitivity of IFN Response Low, RIG-1 OFF	NF-kB OFF, IL-6 Low, IRF3 OFF, IFITM OFF	NF-kB OFF, IL-6 Low, IRF3 OFF, IFITM OFF
		Infected + MAVS siRNA, RNA assay	Virion ON, Sensitivity of IFN Response Low, MAVS OFF	NF-kB OFF, IL-6 Low, IRF3 OFF, IFITM OFF	NF-kB OFF, IL-6 Low, IRF3 OFF, IFITM OFF
		Infected + TPCA-1 (IKK inhibitor), RNA assay	Virion ON, Sensitivity of IFN Response Low, IKK OFF	CXCL10 OFF, IL-6 Low, IFITM OFF, Viral Replication Mid, Cell Death Low	CXCL10 OFF, IL-6 Low, **IFITM Low**, Viral Replication Mid, **Cell Death Mid**
		Infected + Ruxolitinib (JAK1/2 inhibitor), RNA assay	Virion ON, Sensitivity of IFN Response Low, Jak1 OFF, Jak2 OFF	CXCL10 OFF, IL-6 High, IFITM OFF, Viral Replication Mid, Cell Death Low	CXCL10 OFF, **IL-6 Low,** IFITM OFF, **Viral Replication High, Cell Death Mid**

Supplementary Table 4. Experiments from the literature used to validate the network model. For each experiment, the table shows the publication or pre-print, the cell line used, the constraints applied to the model and the expected outcome, based on the experimental result. The final column shows the result of the model simulations, with deviations from the expected results highlighted in bold.

Supplementary Table 5. Drugs used for in silico drug repurposing screens

Drug	Target	Passed PI	COVID-19 Trial	Reference	DrugBank ID	DrugBank Interactions	Notes
1400W	iNOS	No		Garvey, E. et al. (1997)	DB02044	Nitric oxide synthase, endothelial; Nitric oxide synthase, inducible (**iNOS**); Nitric oxide synthase, brain	
2-POAA-OMe	ATF6, IRE1	No		Zhang, H. et al. (2013)	NA	Drug not included in drugbank	
4-PBA	ERstress	Yes		Hsu, A. et al. (2020); Kolb, P. S., et al. (2015)	DB06819	Aromatic-amino-acid aminotransferase; Thermolysin	
4EGI-1	eIF4G	No		Shives, K. et al. (2016)	NA	Drug not included in drugbank	
A779	MasR	No		Meng, Y. et al. (2015)	NA	Drug not included in drugbank	
Ac-DMPD-CMK	GasderminE	No		Xu, W. et al. (2021)	NA	Drug not included in drugbank	
ACEi	ACE1	Yes	NCT04353596	Vaduganathan, M. et al. (2020)	DB00542	Angiotensin-converting enzyme (**ACE1**)	
Actimmune	IFN-II	Yes		Razaghi, A. et al. (2016)	DB00033	Interferon gamma receptor 1; Interferon gamma receptor 2	Actimmune = IFN-II, which binds to IFN-IIR
AEB071	PKC	No		Skvara, H. et al. (2008)	DB12369	No interactions reported in drugbank	
Aliskiren	AngII, Ang1-7	Yes	NCT04432350	Sriram, K. et al. (2020)	DB09026	Renin	
Anifrolumab	IFNAR	Yes		Riggs, J. et al. (2018)	DB11976	No interactions reported in drugbank	
Apilimod	PIKfyve	Yes	NCT04446377	Ou, X. et al. (2020)	DB05611	No interactions reported in drugbank	
ARB	AT1R	Yes	NCT04353596	Vaduganathan, M. et al. (2020)	DB00678	Type-1 angiotensin II receptor (**AT1R**)	
Bananin	nsp13	No		Tanner, J. et al. (2005), Habtemariam, S. et al. (2020)	NA	Drug not included in drugbank	
Baricitinib	Jak1, Jak2, ViralFusion	Yes	NCT04340232	Richardson, P. et al. (2020)	DB11817	Tyrosine-protein kinase **JAK1**; Tyrosine-protein kinase **JAK2**; Protein-tyrosine kinase 2-beta; Tyrosine-protein kinase JAK3	
Basiliximab	IL-2	Yes		Onrust, S et al. (1999)	DB00074	Interleukin-2 receptor subunit alpha; Interleukin-2 receptor subunit beta	Basiliximab is an anti-Il-2R ab but receptor is not modelled
Belnacasan	Caspase-1	Yes		Kudelova, J. et al. (2015)	DB05507	**Caspase-1**; Interleukin-1 beta (**not modelled**)	Effect of Belnacasan on IL-1B is indirect.
BG-323	guanylyltransferase	No		Stahla-Beek, H. et al. (2012)	NA	Drug not included in drugbank	
BX795	TBK1	No		Yu, T. et al. (2015)	NA	Drug not included in drugbank	
C-176	STING	No		Haag, S. et al. (2018)	NA	Drug not included in drugbank	
C16	PKR	No		Gal-Ben-Ari, S. et al. (2018)	NA	Drug not included in drugbank	
Camostat	TMPRSS2	Yes	NCT04583592	Hoffmann, M. et al. (2020)	DB13729	Trypsin-1; Transmembrane protease serine 2 (**TMPRSS2**); Suppressor of tumorigenicity 14 protein; Cholecystokinin	
Canakinumab	IL-1B	Yes	NCT04362813	Ucciferri, C. et al. (2020)	DB06168	Interleukin-1 beta (**IL-1B**)	
Carlumab	CCL2	Yes		Lim, S. et al. (2016)	DB12718	No interactions reported in drugbank	
Cetuximab	EGFR	Yes			DB00002	Epidermal growth factor receptor (EGFR); Low affinity immunoglobulin gamma Fc region receptor III-B; Complement C1q subcomponent subunit A; Complement C1q subcomponent subunit B; Complement C1q subcomponent subunit C; Low affinity immunoglobulin gamma Fc region receptor III-A; High affinity immunoglobulin gamma Fc receptor I; Low affinity immunoglobulin gamma Fc region receptor II-a	
Chloroquine	EndosomalpH, glycosylation, TNF-a, IL-6	Yes	NCT04333628	Wang, M. et al. (2020); Savarino, A. et al. (2003)	DB00608	Glutathione S-transferase A2; Tumor necrosis factor (**TNF-a**); Toll-like receptor 9; Glutathione S-transferase; High mobility group protein B1; Glutathione S-transferase Mu 1; Angiotensin-converting enzyme 2 (**not modelled**)	Chloroquine blocks glycosylation of ACE2, no direct evidence that this prevents binding by spike protein.
Colchicine	Inflammasome	Yes	NCT04355143	Martinez, G. et al. (2017)	DB01394	Tubulin beta chain	
Copanlisib	PI3K	Yes		Yang, J. et al. (2019)	DB12483	Phosphatidylinositol 4,5-bisphosphate 3-kinase catalytic subunit alpha isoform (**PI3K**); Phosphatidylinositol 4,5-bisphosphate 3-kinase catalytic subunit delta isoform (**PI3K**)	
Dexamethasone	IL-10, TNF-a. NF-kB, JNK, ERK, p38, IL-1B, IL-18	Yes	NCT04381936	Selvaraj, V., et al. 2020; Smoak, K. A., et al. 2004; RECOVERY 2021	DB01234	Glucocorticoid receptor; Nuclear receptor subfamily 0 group B member 1; Annexin A1; Nitric oxide synthase, inducible (**not modelled**); Nuclear receptor subfamily 1 group I member 2	Effect fo dexamethasone on iNOS is indirect.
DHMEQ	NF-kB	No		Herrington, F. et al. (2015)	NA	Drug not included in drugbank	
Disulfiram	GasderminD	Yes	NCT04485130	Hu, J. et al. (2020)	DB00822	Aldehyde dehydrogenase, mitochondrial; Dopamine beta-hydroxylase	
DX600	ACE2	No		Uhal, B. et al. (2011)	NA	Drug not included in drugbank	
E64d	CathepsinL	No		Hoffmann, M. et al. (2020)	NA	Drug not included in drugbank	
Emricasan	Caspase-1, Caspase-3, Caspase-8, Caspase-10	Yes		Kudelova, J. et al. (2015)	DB05408	**Caspase-1**; **Caspase-3**; Caspase-7	
Fedratinib	Jak2	Yes		Szelag, M. et al. (2016)	DB12500	Tyrosine-protein kinase **JAK2**; Receptor-type tyrosine-protein kinase FLT3; Tyrosine-protein kinase JAK1 (not modelled)	Literature cited in drugbank does not indicate direct interaction between Federatinib and JAK1.
GLX351322	Nox4	No		Anvari, E. et al. (2015)	NA	Drug not included in drugbank	
GRL-0617	PLpro	No		Shin, D. et al. (2020)	NA	Drug not included in drugbank	
GSK2656157	PERK	No		Axten, J. et al. (2013)	NA	Drug not included in drugbank	
Homoharringtonine	60S	Yes		Choy, K.-T., et al. 2020; Winer, E. S., et al. 2018	DB04865	50S ribosomal protein L2; **60S** ribosomal protein L3	
Infliximab	TNF-a	Yes	NCT04425538	Robinson, P. et al. (2020)	DB00065	Tumor necrosis factor (**TNF-a**)	
Kifunensine	glycosylation	No		Yang, Q. et al. (2020)	DB02742	Mannosyl-oligosaccharide alpha-1,2-mannosidase (**glycosylation**); Alpha-mannosidase 2 (**glycosylation**); Endoplasmic reticulum mannosyl-oligosaccharide 1,2-alpha-mannosidase (**glycosylation**)	
Lopinavir	3CLpro	Yes	NCT04372628	Choy, K.-T., et al. 2020; Ma, C., et al. 2020; Cao, B., et al. 2020	DB01601	Human immunodeficiency virus type 1 protease	
Losmapimod	p38	Yes	NCT04511819		DB12270	No interactions reported in drugbank	
LY2275796	eIF4E	No			DB05165	Eukaryotic translation initiation factor 4E (**eIF-4E**)	
LY2584702	S6K	No		Tolcher, A. et al. (2014)	DB12690	No interactions reported in drugbank	
MCC950	NLRP3	No		Coll, R. (2015)	NA	Drug not included in drugbank	
MI-1851	furin	No		Bestle, D. et al. (2020); Xiu, S. et al. (2020)	NA	Drug not included in drugbank	
Miltefosine	Akt	Yes		Nitulescu, G. et al. (2015)	DB09031	P-glycoprotein 1	
miR-146a	TRAF6	No		Gao, W. et al. (2017)	NA	Drug not included in drugbank	
N-acetylcysteine	ROS	Yes		Meng, Y. et al. (2015)	DB06151	Glutathione synthetase; Cystine/glutamate transporter; NAPQI (N-acetyl-p-benzoquinone imine); Aminoacylase-1; Inhibitor of nuclear factor kappa-B kinase subunit beta (**not modelled**); Inhibitor of nuclear factor kappa-B kinase subunit alpha (**not modelled**); Glutamate receptor ionotropic, NMDA 2B; Glutamate receptor ionotropic, NMDA 1; Glutamate receptor ionotropic, NMDA 2A; Glutamate receptor ionotropic, NMDA 2D; Glutamate receptor ionotropic, NMDA 3A	Effect of N-acetylcysteine on IKK is indirect.
Necrostatin-1	RIPK1	No		Degterev, A. et al. (2019)	NA	Drug not included in drugbank	
Necrosulfonamide	MLKL	No		Liao, D. et al. (2014)	NA	Drug not included in drugbank	
Nivocasan	Caspase-1, Caspase-8	Yes		Kudelova, J. et al. (2015)	DB12720	No interactions reported in drugbank	
NSC80734	IL-18	No		Krumm, B. et al. (2017)	NA	Drug not included in drugbank	
PF-06826647	Tyk2	No		Gerstenberger, B. (2020)	NA	Drug not included in drugbank	
Rapamycin	mTORC1	Yes	NCT04341675	Ballou, L. et al. (2008)	DB00877	Serine/threonine-protein kinase **mTOR**; Peptidyl-prolyl cis-trans isomerase FKBP1A; Fibroblast growth factor 2	
Recombinant_IL-10	IL-10	Yes		Asadullah, K. et al. (2003)	NA	Drug not included in drugbank	
RecombinantIFNlambd	IFN-III	No		Donnelly, R. et al. (2010)	DB12880	No interactions reported in drugbank	
Remdesivir	nsp12-nsp7-nsp8	Yes	NCT04280705	Wang, M., et al. 2020; Beigel, J. H., et al. 2020	DB14761	Replicase polyprotein 1ab; Replicase polyprotein 1ab; Replicase polyprotein 1ab; RNA-directed RNA polymerase L (**nsp12-nsp7-nsp8**)	
Roferon-A	IFN-I	Yes	NCT04293887	Mantlo, E. et al. (2020)	DB00034	Interferon alpha/beta receptor 1; Interferon alpha/beta receptor 2	Roferon-A = IFN-I, which binds to IFN-IR
Ruxolitinib	Jak1, Jak2	Yes	NCT04377620	Szelag, M., et al. 2016; Cao, Y., et al. 2020; Thorne, L. G., et al. 2021	DB08877	Tyrosine-protein kinase **JAK1**; Tyrosine-protein kinase **JAK2**	
Salubrinal	eIF2	No		Boyce, M. et al. (2005)	NA	Drug not included in drugbank	
SAR113945	IKK	Yes		Herrington, F. et al. (2015)	NA	Drug not included in drugbank	
SP600125	JNK	No		Wang, J. et al. (2017)	DB01782	Mitogen-activated protein kinase 10 (**JNK**); C-Jun-amino-terminal kinase-interacting protein 1; Mitogen-activated protein kinase 8 (**JNK**); Dual specificity protein kinase TTK	
Takinib	TAK1	No		Totzke, J. et al. (2017)	NA	Drug not included in drugbank	
TAPI-1	ADAM17	No		Palau, V. et al. (2020)	NA	Drug not included in drugbank	
Tetrandrine	TPC2	Yes		Ou, X. et al. (2020)	DB14066	P-glycoprotein 1	
Tocilizumab	IL-6	Yes	NCT04479358	Mihara, M., et al. 2011; RECOVERY 2021	DB06273	Interleukin-6 receptor subunit alpha (**IL-6**)	Tocilizumab is an anti-IL-6R ab but receptor is not modelled
TP29	nsp16	No		Decroly, E. et al. (2008)	NA	Drug not included in drugbank	
TRV120027	AT1R	No		Forrester, S. et al. (2018)	DB12199	No interactions reported in drugbank	
Ulixertinib	ERK	Yes		Hsu, A. et al. (2020)	DB13930	Mitogen-activated protein kinase 3 (**ERK**); Mitogen-activated protein kinase 1 (**ERK**)	
Umifenovir	ACE2-Spike, S	Yes	NCT04260594	Wang, X. et al. (2020)	DB13609	No interactions reported in drugbank	
Y-27632	ROCK	No		Meng, Y. et al. (2015)	DB08756	cAMP-dependent protein kinase catalytic subunit alpha; cAMP-dependent protein kinase inhibitor alpha; Rho-associated protein kinase 1 (**ROCK**); Rho-associated protein kinase 2 (**ROCK**)	
Zotatifin	eIF4A	No	NCT04632381	Gordon, D. et al. (2020)	DB16408	No interactions reported in drugbank	

Supplementary Table 5. Drugs used for *in silico* drug repurposing screens. For each drug, the table contains its targets, whether it has passed phase II clinical trials (1 means it has), clinical trials identifier (if in trials to treat COVID-19), a reference or drugbank ID, any targets listed in the Drugbank database and notes on how interaction of the drugs with these targets are modelled. In the drugbank targets column, the node in the model used to represent a specific target is written in bold. In some cases, drugbank includes indirect drug interactions, where the effect of the drug is mediated by other proteins or pathways, such targets were not modelled.

Supplementary Table 6. Classification of nodes in the network model

Node	Type	Pathway
3CLpro	Cellular Process	Non-Structural
40S	Host Protein or Complex	Translation
43S	Host Protein or Complex	Translation
4E-BP	Host Protein or Complex	Translation
60S	Host Protein or Complex	Translation
7SL	RNA	IFN
ACE1	Host Protein or Complex	AT1R
ACE2	Viral Protein or Complex	Entry
ACE2-Spike	Viral Protein or Complex	Entry
ADAM17	Host Protein or Complex	Entry
Akt	Host Protein or Complex	Signalling
Ang1-7	Host Protein or Complex	AT1R
AngII	Host Protein or Complex	AT1R
Apoptosis	Cellular Process	Inflammasome
ASC	Host Protein or Complex	Inflammasome
AT1R	Host Protein or Complex	AT1R
ATF6	Host Protein or Complex	AT1R
Cap-independentTranslation	Cellular Process	Translation
Caspase-1	Host Protein or Complex	Inflammasome
Caspase-10	Host Protein or Complex	Inflammasome
Caspase-3	Host Protein or Complex	Inflammasome
Caspase-8	Host Protein or Complex	Inflammasome
CathepsinL	Host Protein or Complex	Entry
CCL2	Host Protein or Complex	
CellDeath	Cellular Process	Inflammasome
CH25H	Host Protein or Complex	IFN
CXCL10	Host Protein or Complex	
CXCL11	Host Protein or Complex	
CXCL9	Host Protein or Complex	
CytosolicPRRs	Host Protein or Complex	Sensing of Viral RNA
DMVformation	Cellular Process	RTC
E	Viral Protein or Complex	Structural
EGFR	Host Protein or Complex	Signalling
eIF2	Host Protein or Complex	Translation
eIF4A	Host Protein or Complex	Translation
eIF4B	Host Protein or Complex	Translation
eIF4E	Host Protein or Complex	Translation
eIF4F	Host Protein or Complex	Translation
eIF4G	Host Protein or Complex	Translation
EndosomalpH	Cellular Process	Entry
ERK	Host Protein or Complex	Signalling
ERstress	Cellular Process	AT1R
Fibrosis	Cellular Process	Signalling
furin	Host Protein or Complex	Structural
GasderminD	Host Protein or Complex	Inflammasome
GasderminE	Host Protein or Complex	Inflammasome
glycosylation	Cellular Process	Structural
guanylyltransferase	Host Protein or Complex	Translation

Node	Type	Pathway
HostCap-dependentTranslation	Cellular Process	Translation
HostmRNAs	RNA	Translation
HostProteinSynthesis	Cellular Process	Translation
IFITM	Host Protein or Complex	IFN
IFN-I	Host Protein or Complex	
IFN-I_SG	Cellular Process	IFN
IFN-II	Host Protein or Complex	
IFN-II_SG	Cellular Process	IFN
IFN-III	Host Protein or Complex	
IFN-III_SG	Cellular Process	IFN
IFN-LR1	Host Protein or Complex	IFN
IFNAR	Host Protein or Complex	IFN
IFNGR	Host Protein or Complex	IFN
IkBa	Host Protein or Complex	Sensing of Viral RNA
IKK	Host Protein or Complex	Sensing of Viral RNA
IL-10	Host Protein or Complex	
IL-18	Host Protein or Complex	
IL-1B	Host Protein or Complex	
IL-2	Host Protein or Complex	
IL-6	Host Protein or Complex	
IL-8	Host Protein or Complex	
ImmuneCells	Cellular Process	
Inflammasome	Host Protein or Complex	Inflammasome
Inflammation	Cellular Process	
iNOS	Host Protein or Complex	Inflammasome
IRE1	Host Protein or Complex	AT1R
IRF3	Host Protein or Complex	IFN
IRF7	Host Protein or Complex	IFN
IRF9	Host Protein or Complex	IFN
Jak1	Host Protein or Complex	IFN
Jak2	Host Protein or Complex	IFN
JNK	Host Protein or Complex	Signalling
LY6E	Host Protein or Complex	IFN
M	Viral Protein or Complex	Structural
MasR	Host Protein or Complex	AT1R
MAVS	Host Protein or Complex	Sensing of Viral RNA
MDA5	Host Protein or Complex	Sensing of Viral RNA
MLKL	Host Protein or Complex	Inflammasome
mTORC1	Host Protein or Complex	Signalling
N	Viral Protein or Complex	Structural
Necroptosis	Cellular Process	Inflammasome
NF-kB	Host Protein or Complex	Inflammasome
NKRF	Host Protein or Complex	Sensing of Viral RNA
NLRP3	Host Protein or Complex	Inflammasome
NO	Cellular Process	Inflammasome
Nox4	Host Protein or Complex	AT1R
nsp1	Viral Protein or Complex	Non-Structural
nsp1_nsp2_nsp3_nsp4	Viral Protein or Complex	Non-Structural
nsp10	Viral Protein or Complex	Non-Structural
nsp10-nsp14	Viral Protein or Complex	RTC

Node	Type	Pathway
nsp10-nsp16	Viral Protein or Complex	RTC
nsp11	Viral Protein or Complex	Non-Structural
nsp12	Viral Protein or Complex	Non-Structural
nsp12-nsp7-nsp8	Viral Protein or Complex	RTC
nsp13	Viral Protein or Complex	Non-Structural
nsp14	Viral Protein or Complex	Non-Structural
nsp15	Viral Protein or Complex	Non-Structural
nsp16	Viral Protein or Complex	Non-Structural
nsp2	Viral Protein or Complex	Non-Structural
nsp3	Viral Protein or Complex	Non-Structural
nsp4	Viral Protein or Complex	Non-Structural
nsp5	Viral Protein or Complex	Non-Structural
nsp6	Viral Protein or Complex	Non-Structural
nsp7	Viral Protein or Complex	Non-Structural
nsp8	Viral Protein or Complex	Non-Structural
nsp9	Viral Protein or Complex	Non-Structural
OAS	Host Protein or Complex	IFN
p105	Host Protein or Complex	Inflammasome
p38	Host Protein or Complex	Signalling
p3a	Viral Protein or Complex	Accessory
p3b	Viral Protein or Complex	Accessory
p6	Viral Protein or Complex	Accessory
p7a	Viral Protein or Complex	Accessory
p7b	Viral Protein or Complex	Accessory
p8	Viral Protein or Complex	Accessory
p9b	Viral Protein or Complex	Accessory
p9c	Viral Protein or Complex	Accessory
PANoptosome	Host Protein or Complex	Inflammasome
PERK	Host Protein or Complex	AT1R
PI3K	Host Protein or Complex	Signalling
PIKfyve	Host Protein or Complex	Entry
PKC	Host Protein or Complex	Signalling
PKR	Host Protein or Complex	IFN
PLpro	Cellular Process	Non-Structural
pp1a	Viral Protein or Complex	Non-Structural
pp1ab	Viral Protein or Complex	Non-Structural
Pyroptosis	Cellular Process	Inflammasome
RIG-1	Host Protein or Complex	Sensing of Viral RNA
RIPK1	Host Protein or Complex	Sensing of Viral RNA
RNAseL	Host Protein or Complex	IFN
ROCK	Host Protein or Complex	Signalling
ROS	Cellular Process	AT1R
S	Viral Protein or Complex	Structural
S6K	Host Protein or Complex	Translation
SensitivityOfIFNResponse	Sensitivity of IFN response	
Splicing	Cellular Process	Translation
SRP	Host Protein or Complex	IFN
STAT1	Host Protein or Complex	IFN
STAT2	Host Protein or Complex	IFN
STING	Host Protein or Complex	Sensing of Viral RNA

Node	Type	Pathway
SyncytiaFormation	Cellular Process	
T-cellInfiltration	Cellular Process	
TAK1	Host Protein or Complex	Sensing of Viral RNA
TANK	Host Protein or Complex	Sensing of Viral RNA
TBK1	Host Protein or Complex	Sensing of Viral RNA
TLR3	Host Protein or Complex	Sensing of Viral RNA
TLR3-TRIF	Host Protein or Complex	Sensing of Viral RNA
TMPRSS2	Host Protein or Complex	Entry
TNF-a	Host Protein or Complex	
TPC2	Host Protein or Complex	Entry
TRAF3	Host Protein or Complex	Sensing of Viral RNA
TRAF6	Host Protein or Complex	Sensing of Viral RNA
TRIF	Host Protein or Complex	Sensing of Viral RNA
Tyk2	Host Protein or Complex	IFN
U1_U2snRNAs	RNA	Translation
ViralCap-dependentTranslation	Cellular Process	Translation
ViralEntry	Cellular Process	Entry
ViralEntry1	Cellular Process	Entry
ViralFusion	Cellular Process	Entry
ViralGenome	RNA	RTC
ViralGenomeDegradation	Cellular Process	Structural
ViralReplication	Cellular Process	Structural
ViralsgRNAs	RNA	Translation
Virion	Viral Protein or Complex	
VirionERGIC	Viral Protein or Complex	Structural
XBP1	Host Protein or Complex	AT1R
ZAP	Host Protein or Complex	IFN

Supplementary Table 6. Classification of nodes in the network model. For each node, the type and pathway within the model are specified.

Supplementary Table 7. *In silico* simulation of Caco-2 cell drug treatments

Condition	Treatment	Inflammation	Viral Entry	Viral Replication
Caco-2	Control	1	4	4
Caco-2	Apilimod	1	2	4
Caco-2	Camostat	1	2	4
Caco-2	Camostat + Apilimod	0	0	0
Caco-2	Miltefosine	1	4	4
Caco-2	Dexamethasone	0	4	4
Caco-2	Dexamethasone + Miltefosine	0	0	0

Supplementary Table 7. *In silico* simulation of Caco-2 cell drug treatments. The Caco-2 cell line is modelled *in silico* by setting the "ImmuneCells" and "SensitivityOfIFNResponse" nodes to zero, reflecting the fact that Caco-2 cultures consist of clonal colorectal epithelial cells and do not mount an IFN response to SARS-CoV-2 infection.

Supplementary References

Akopian, D., Shen, K., Zhang, X. & Shan, S. Signal Recognition Particle: An Essential Protein-Targeting Machine. *Annu. Rev. Biochem.* **82**, 693–721 (2013)

Alexandre, J., Cracowski, J. L., Richard, V. & Bouhanick, B. Renin-angiotensin-aldosterone system and COVID-19 infection. *Ann. Endocrinol. (Paris).* **81**, 63–67 (2020)

Angelini, M. M., Akhlaghpour, M., Neuman, B. W. & Buchmeier, M. J. Severe acute respiratory syndrome coronavirus nonstructural proteins 3, 4, and 6 induce double-membrane vesicles. *MBio* **4**, (2013)

Anvari, E., Wikström, P., Walum, E. & Welsh, N. The novel NADPH oxidase 4 inhibitor GLX351322 counteracts glucose intolerance in high-fat diet-treated C57BL/6 mice. *Free Radic. Res.* **49**, 1308–1318 (2015)

Asadullah, K., Sterry, W. & Volk, H. D. Interleukin-10 therapy - Review of a new approach. *Pharmacol. Rev.* **55**, 241–269 (2003)

Axten, J. M. *et al.* Discovery of GSK2656157: An optimized PERK inhibitor selected for preclinical development. *ACS Med. Chem. Lett.* **4**, 964–968 (2013)

Ballou, L. M. & Lin, R. Z. Rapamycin and mTOR kinase inhibitors. *J. Chem. Biol.* **1**, 27–36 (2008)

Banerjee, A. K. *et al.* SARS-CoV-2 Disrupts Splicing, Translation, and Protein Trafficking to Suppress Host Defenses. *Cell* **183**, 1325-1339.e21 (2020)

Barrat, F. J., Crow, M. K. & Ivashkiv, L. B. Interferon target-gene expression and epigenomic signatures in health and disease. *Nat. Immunol.* **20**, 1574–1583 (2019)

Beigel, J. H. *et al.* Remdesivir for the Treatment of Covid-19 — Final Report. *N. Engl. J. Med.* **383**, 1813–1826 (2020)

Bestle, D. *et al.* TMPRSS2 and furin are both essential for proteolytic activation of SARS-CoV-2 in human airway cells. *Life Sci. Alliance* **3**, 1–14 (2020)

Bonnet, M. C., Daurat, C., Ottone, C. & Meurs, E. F. The N-terminus of PKR is responsible for the activation of the NF-κB signaling pathway by interacting with the IKK complex. *Cell. Signal.* **18**, 1865–1875 (2006)

Bouhaddou, M. *et al.* The Global Phosphorylation Landscape of SARS-CoV-2 Infection. *Cell* **182**, 685-712.e19 (2020)

Bouvet, M. *et al.* In Vitro Reconstitution of SARS-Coronavirus mRNA Cap Methylation. *PLoS Pathog.* **6**, e1000863 (2010)

Bouvet, M. *et al.* RNA 3′-end mismatch excision by the severe acute respiratory syndrome coronavirus nonstructural protein nsp10/nsp14 exoribonuclease complex. *Proc. Natl. Acad. Sci. U. S. A.* **109**, 9372–9377 (2012)

Boyce, M. *et al.* A selective inhibitor of elF2α dephosphorylation protects cells from ER stress. *Science (80-.).* **307**, 935–939 (2005)

Buchrieser, J. *et al.* Syncytia formation by SARS-CoV-2-infected cells. *EMBO J.* **39**, 1–12 (2020)

Cao, B. *et al.* A Trial of Lopinavir–Ritonavir in Adults Hospitalized with Severe Covid-19. *N. Engl. J. Med.* **382**, 1787–1799 (2020)

Cao, Y. *et al.* Ruxolitinib in treatment of severe coronavirus disease 2019 (COVID-19): A multicenter, single-blind, randomized controlled trial. *J. Allergy Clin. Immunol.* **146**, 137-146.e3 (2020)

Chan, C.-P. *et al.* Modulation of the Unfolded Protein Response by the Severe Acute Respiratory Syndrome Coronavirus Spike Protein. *J. Virol.* **80**, 9279–9287 (2006)

Chen, J. *et al.* Structural Basis for Helicase-Polymerase Coupling in the SARS-CoV-2 Replication-Transcription Complex. *Cell* **182**, 1560-1573.e13 (2020)

Chen, Y., Liu, Q. & Guo, D. Emerging coronaviruses: Genome structure, replication, and pathogenesis. *J. Med. Virol.* **92**, 418–423 (2020)

Chen, Z. & John Wherry, E. T cell responses in patients with COVID-19. *Nat. Rev. Immunol.* **20**, 529–536 (2020)

Choy, K. T. *et al.* Remdesivir, lopinavir, emetine, and homoharringtonine inhibit SARS-CoV-2 replication in vitro. *Antiviral Res.* **178**, 104786 (2020)

Christgen, S. *et al.* Identification of the PANoptosome: A Molecular Platform Triggering Pyroptosis, Apoptosis, and Necroptosis (PANoptosis). *Front. Cell. Infect. Microbiol.* **10**, 1–12 (2020)

Coll, R. C. *et al.* A small-molecule inhibitor of the NLRP3 inflammasome for the treatment of inflammatory diseases. *Nat. Med.* **21**, 248–257 (2015)

Cong, Y. *et al.* Nucleocapsid Protein Recruitment to Replication-Transcription Complexes Plays a Crucial Role in Coronaviral Life Cycle. *J. Virol.* **94**, 1–21 (2020)

Davidson, A. D. *et al.* Characterisation of the transcriptome and proteome of SARS-CoV-2 reveals a cell passage induced in-frame deletion of the furin-like cleavage site from the spike glycoprotein. *Genome Med.* **12**, 15–68 (2020)

Decroly, E. *et al.* Coronavirus Nonstructural Protein 16 Is a Cap-0 Binding Enzyme Possessing (Nucleoside-2′O)-Methyltransferase Activity. *J. Virol.* **82**, 8071–8084 (2008)

Degterev, A., Ofengeim, D. & Yuan, J. Targeting RIPK1 for the treatment of human diseases. *Proc. Natl. Acad. Sci. U. S. A.* **116**, 9714–9722 (2019)

Diao, B. *et al.* Reduction and Functional Exhaustion of T Cells in Patients With Coronavirus Disease 2019 (COVID-19). *Front. Immunol.* **11**, 727–7 (2020)

Donnelly, R. P. & Kotenko, S. V. Interferon-lambda: A new addition to an old family. *J. Interf. Cytokine Res.* **30**, 555–564 (2010)

Falvo, J. V., Tsytsykova, A. V. & Goldfeld, A. E. Transcriptional control of the TNF Gene. *Curr. Dir. Autoimmun.* **11**, 27–60 (2010)

Fehr, A. R. & Perlman, S. Coronaviruses: An Overview of Their Replication and Pathogenesis. in *Coronaviruses: Methods and Protocols* vol. 1282 1–23 (2015).

Forrester, S. J. *et al.* Angiotensin II signal transduction: An update on mechanisms of physiology and pathophysiology. *Physiol. Rev.* **98**, 1627–1738 (2018)

Forrester, S. J. *et al.* Epidermal Growth Factor Receptor Transactivation: Mechanisms, Pathophysiology, and Potential Therapies in the Cardiovascular System. *Annu. Rev. Pharmacol. Toxicol.* **56**, 627–653 (2016)

Gal-Ben-Ari, S., Barrera, I., Ehrlich, M. & Rosenblum, K. PKR: A kinase to remember. *Front. Mol. Neurosci.* **11**, 1–20 (2019)

Gao, W., Xiong, Y., Li, Q. & Yang, H. Inhibition of toll-like receptor signaling as a promising therapy for inflammatory diseases: A journey from molecular to nano therapeutics. *Front. Physiol.* **8**, (2017)

Garvey, E. P. *et al.* 1400W is a slow, tight binding, and highly selective inhibitor of inducible nitric-oxide synthase in vitro and in vivo. *J. Biol. Chem.* **272**, 4959–4963 (1997)

Gerstenberger, B. S. *et al.* Discovery of Tyrosine Kinase 2 (TYK2) Inhibitor (PF-06826647) for the Treatment of Autoimmune Diseases. *J. Med. Chem.* **63**, 13561–13577 (2020)

Gordon, D. E. *et al.* A SARS-CoV-2 protein interaction map reveals targets for drug repurposing. *Nature* **583**, 459–468 (2020)

Goritzka, M. *et al.* Alpha/Beta Interferon Receptor Signaling Amplifies Early Proinflammatory Cytokine Production in the Lung during Respiratory Syncytial Virus Infection. *J. Virol.* **88**, 6128–6136 (2014)

Haag, S. M. *et al.* Targeting STING with covalent small-molecule inhibitors. *Nature* **559**, 269–273 (2018)

Habtemariam, S. *et al.* Should We Try SARS-CoV-2 Helicase Inhibitors for COVID-19 Therapy? *Arch. Med. Res.* **51**, 733–735 (2020)

Hackbart, M., Deng, X. & Baker, S. C. Coronavirus endoribonuclease targets viral polyuridine sequences to evade activating host sensors. *Proc. Natl. Acad. Sci. U. S. A.* **117**, 8094–8103 (2020)

Haga, S. *et al.* Modulation of TNF-α-converting enzyme by the spike protein of SARS-CoV and ACE2 induces TNF-α production and facilitates viral entry. *Proc. Natl. Acad. Sci. U. S. A.* **105**, 7809–7814 (2008)

Hamid, Q. & Tulic, M. Immunobiology of asthma. *Annu. Rev. Physiol.* **71**, 489–507 (2009)

Hayden, M. S. & Ghosh, S. Regulation of NF-κB by TNF family cytokines. *Semin. Immunol.* **26**, 253–266 (2014)

Hecker, L. *et al.* NADPH oxidase-4 mediates myofibroblast activation and fibrogenic responses to lung injury. *Nat. Med.* **15**, 1077–1081 (2009)

Herrington, F. D., Carmody, R. J. & Goodyear, C. S. Modulation of NF-κB Signaling as a Therapeutic Target in Autoimmunity. *J. Biomol. Screen.* **21**, 223–242 (2016)

Hoffmann, M. *et al.* SARS-CoV-2 Cell Entry Depends on ACE2 and TMPRSS2 and Is Blocked by a Clinically Proven Protease Inhibitor. *Cell* **181**, 271-280.e8 (2020)

Hsu, A. C. Y. *et al.* SARS-CoV-2 Spike protein promotes hyper-inflammatory response that can be ameliorated by Spike-antagonistic peptide and FDA-approved ER stress and MAP kinase inhibitors in vitro. *bioRxiv* (2020) doi:10.1101/2020.09.30.317818

Hu, J. J. *et al.* FDA-approved disulfiram inhibits pyroptosis by blocking gasdermin D pore formation. *Nat. Immunol.* **21**, 736–745 (2020)

Ingraham, N. E. *et al.* Understanding the renin-angiotensin-aldosterone-SARS-CoV axis: A comprehensive review. *Eur. Respir. J.* **56**, 2000912 (2020)

Issa, E., Merhi, G., Panossian, B., Salloum, T. & Tokajian, S. SARS-CoV-2 and ORF3a: Nonsynonymous Mutations, Functional Domains, and Viral Pathogenesis. *mSystems* **5**, e00266-20 (2020)

Ivashkiv, L. B. IFNγ: signalling, epigenetics and roles in immunity, metabolism, disease and cancer immunotherapy. *Nat. Rev. Immunol.* **18**, 545–558 (2018)

Kany, S., Vollrath, J. T. & Relja, B. Cytokines in inflammatory disease. *Int. J. Mol. Sci.* **20**, 6008–31 (2019)

Karki, R. *et al.* Synergism of TNF-α and IFN-γ Triggers Inflammatory Cell Death, Tissue Damage, and Mortality in SARS-CoV-2 Infection and Cytokine Shock Syndromes. *Cell* **184**, 149-168.e17 (2021)

Kim, D. *et al.* The Architecture of SARS-CoV-2 Transcriptome. *Cell* **181**, 914-921.e10 (2020)

Kim, S. Y. *et al.* Characterization of heparin and severe acute respiratory syndrome-related coronavirus 2 (SARS-CoV-2) spike glycoprotein binding interactions. *Antiviral Res.* **181**, (2020)

Kirchdoerfer, R. N. & Ward, A. B. Structure of the SARS-CoV nsp12 polymerase bound to nsp7 and nsp8 co-factors. *Nat. Commun.* **10**, 2342–9 (2019)

Kolb, P. S. *et al.* The therapeutic effects of 4-phenylbutyric acid in maintaining proteostasis. *Int. J. Biochem. Cell Biol.* **61**, 45–52 (2015)

Kondrikov, D., Caldwell, R. B., Dong, Z. & Su, Y. Reactive oxygen species-dependent RhoA activation mediates collagen synthesis in hyperoxic lung fibrosis. *Free Radic. Biol. Med.* **50**, 1689–1698 (2011)

Konno, Y. *et al.* SARS-CoV-2 ORF3b Is a Potent Interferon Antagonist Whose Activity Is Increased by a Naturally Occurring Elongation Variant. *Cell Rep.* **32**, 108185 (2020)

Krumm, B., Meng, X., Xiang, Y. & Deng, J. Identification of small molecule inhibitors of Interleukin-18. *Sci. Rep.* **7**, 483 (2017)

Kudelova, J., Fleischmannova, J., Adamova, E. & Matalova, E. Review article. *J. Physiol. Pharmacol. An Off. J. Polish Physiol. Soc.* **4**, 473–482 (2015)

Kurosaki, T., Popp, M. W. & Maquat, L. E. Quality and quantity control of gene expression by nonsense-mediated mRNA decay. *Nat. Rev. Mol. Cell Biol.* **20**, 406–420 (2019)

Lan, J. *et al.* Structure of the SARS-CoV-2 spike receptor-binding domain bound to the ACE2 receptor. *Nature* **581**, 215–220 (2020)

Lee, S. J., Channappanavar, R. & Kanneganti, T. D. Coronaviruses: Innate Immunity, Inflammasome Activation, Inflammatory Cell Death, and Cytokines. *Trends Immunol.* **41**, 1083–1099 (2020)

Lei, X. *et al.* Activation and evasion of type I interferon responses by SARS-CoV-2. *Nat. Commun.* **11**, 3810–12 (2020)

Li, J. Y. *et al.* The ORF6, ORF8 and nucleocapsid proteins of SARS-CoV-2 inhibit type I interferon signaling pathway. *Virus Res.* **286**, 198074 (2020)

Li, J. *et al.* Virus-Host Interactome and Proteomic Survey Reveal Potential Virulence Factors Influencing SARS-CoV-2 Pathogenesis. *Med* **2**, 99-112.e7 (2021)

Liao, D. *et al.* Necrosulfonamide inhibits necroptosis by selectively targeting the mixed lineage kinase domain-like protein. *Medchemcomm* **5**, 333–337 (2014)

Liao, W., Lin, J. X. & Leonard, W. J. Interleukin-2 at the Crossroads of Effector Responses, Tolerance, and Immunotherapy. *Immunity* **38**, 13–25 (2013)

Lim, S. Y., Yuzhalin, A. E., Gordon-Weeks, A. N. & Muschel, R. J. Targeting the CCL2-CCR2 signaling axis in cancer metastasis. *Oncotarget* **7**, 28697–28710 (2016)

Liu, J. *et al.* Longitudinal characteristics of lymphocyte responses and cytokine profiles in the peripheral blood of SARS-CoV-2 infected patients. *EBioMedicine* **55**, 102763 (2020)

Liu, T., Zhang, L., Joo, D. & Sun, S. C. NF-κB signaling in inflammation. *Signal Transduct. Target. Ther.* **2**, a000034-9 (2017)

Lokugamage, K. G. *et al.* Type I Interferon Susceptibility Distinguishes SARS-CoV-2 from SARS-CoV. *J. Virol.* **94**, (2020)

Ma, C. *et al.* Boceprevir, GC-376, and calpain inhibitors II, XII inhibit SARS-CoV-2 viral replication by targeting the viral main protease. *Cell Res.* **30**, 678–692 (2020)

Magnuson, B., Ekim, B. & Fingar, D. C. Regulation and function of ribosomal protein S6 kinase (S6K) within mTOR signalling networks. *Biochem. J.* **441**, 1–21 (2012)

Makris, S., Paulsen, M. & Johansson, C. Type I interferons as regulators of lung inflammation. *Front. Immunol.* **8**, 1–10 (2017)

Man, S. M., Karki, R. & Kanneganti, T. D. Molecular mechanisms and functions of pyroptosis, inflammatory caspases and inflammasomes in infectious diseases. *Immunol. Rev.* **277**, 61–75 (2017)

Mantlo, E., Bukreyeva, N., Maruyama, J., Paessler, S. & Huang, C. Antiviral activities of type I interferons to SARS-CoV-2 infection. *Antiviral Res.* **179**, 104811–5 (2020)

Martínez, G. J., Celermajer, D. S. & Patel, S. The NLRP3 inflammasome and the emerging role of colchicine to inhibit atherosclerosis-associated inflammation. *Atherosclerosis* **269**, 262–271 (2018)

Matera, A. G. & Wang, Z. A day in the life of the spliceosome. *Nat. Rev. Mol. Cell Biol.* **15**, 108–121 (2014)

Meng, Y. *et al.* The angiotensin-converting enzyme 2/angiotensin (1-7)/mas axis protects against lung fibroblast migration and lung fibrosis by inhibiting the NOX4-derived ROS-mediated RhoA/Rho kinase pathway. *Antioxidants Redox Signal.* **22**, 241–258 (2015)

Mihara, M., Ohsugi, Y. & Kishimoto, T. Tocilizumab, a humanized anti-interleukin-6 receptor antibody, for treatment of rheumatoid arthritis. *Open Access Rheumatol. Res. Rev.* **3**, 19–29 (2011)

Miorin, L. *et al.* SARS-CoV-2 Orf6 hijacks Nup98 to block STAT nuclear import and antagonize interferon signaling. *Proc. Natl. Acad. Sci. U. S. A.* **117**, 28344–28354 (2020)

Mogensen, T. H. Pathogen recognition and inflammatory signaling in innate immune defenses. *Clin. Microbiol. Rev.* **22**, 240–273 (2009)

Nakagawa, K., Lokugamage, K. G. & Makino, S. Viral and Cellular mRNA Translation in Coronavirus-Infected Cells. *Adv. Virus Res.* **96**, 165–192 (2016)

Nchioua, R. *et al.* The Zinc Finger Antiviral Protein restricts SARS-CoV-2. *MBio* **130**, 2620–40 (2020)

Nitulescu, G. M. *et al.* Akt inhibitors in cancer treatment: The long journey from drug discovery to clinical use (Review). *Int. J. Oncol.* **48**, 869–885 (2016)

Ogando, N. S. *et al.* The Enzymatic Activity of the nsp14 Exoribonuclease Is Critical for Replication of MERS-CoV and SARS-CoV-2. *J. Virol.* **94**, (2020)

Onrust, S. V & Wiseman, L. R. Basiliximab. **57**, 207–213 (1999)

Ou, X. *et al.* Characterization of spike glycoprotein of SARS-CoV-2 on virus entry and its immune cross-reactivity with SARS-CoV. *Nat. Commun.* **11**, 1620–12 (2020)

Palau, V., Riera, M. & Soler, M. J. ADAM17 inhibition may exert a protective effect on COVID-19. *Nephrol. Dial. Transplant.* **35**, 1071–1072 (2020)

Pathinayake, P. S. *et al.* Understanding the unfolded protein response in the pathogenesis of asthma. *Front. Immunol.* **9**, 621–13 (2018)

Pechkovsky, D. V. *et al.* CCR2 and CXCR3 agonistic chemokines are differently expressed and regulated in human alveolar epithelial cells type II. *Respir. Res.* **6**, 75 (2005)

Pfaender, S. *et al.* LY6E impairs coronavirus fusion and confers immune control of viral disease. *Nat. Microbiol.* **5**, 1330–1339 (2020)

Razaghi, A., Owens, L. & Heimann, K. Review of the recombinant human interferon gamma as an immunotherapeutic: Impacts of production platforms and glycosylation. *J. Biotechnol.* **240**, 48–60 (2016)

Redza-Dutordoir, M. & Averill-Bates, D. A. Activation of apoptosis signalling pathways by reactive oxygen species. *Biochim. Biophys. Acta - Mol. Cell Res.* **1863**, 2977–2992 (2016)

Ren, Y. *et al.* The ORF3a protein of SARS-CoV-2 induces apoptosis in cells. *Cell. Mol. Immunol.* **17**, 881–883 (2020)

Ribero, M. S., Jouvenet, N., Dreux, M. & Nisole, S. Interplay between SARS-CoV-2 and the type I interferon response. *PLoS Pathog.* **16**, e1008737-22 (2020)

Richardson, P. *et al.* Baricitinib as potential treatment for 2019-nCoV acute respiratory disease. *Lancet* **395**, e30–e31 (2020)

Riggs, J. M. *et al.* Characterisation of anifrolumab, a fully human anti-interferon receptor antagonist antibody for the treatment of systemic lupus erythematosus. *Lupus Sci. Med.* **5**, 1–11 (2018)

Robinson, P. C. *et al.* The Potential for Repurposing Anti-TNF as a Therapy for the Treatment of COVID-19. *Med* **1**, 90–102 (2020)

Romano, M., Ruggiero, A., Squeglia, F., Maga, G. & Berisio, R. A Structural View of SARS-CoV-2 RNA Replication Machinery: RNA Synthesis, Proofreading and Final Capping. *Cells* **9**, (2020)

Savarino, A., Boelaert, J. R., Cassone, A., Majori, G. & Cauda, R. Effects of chloroquine on viral infections: An old drug against today's diseases? *Lancet Infect. Dis.* **3**, 722–727 (2003)

Schoggins, J. W. & Rice, C. M. Interferon-stimulated genes and their antiviral effector functions. *Curr. Opin. Virol.* **1**, 519–525 (2011)

Schubert, K. *et al.* SARS-CoV-2 Nsp1 binds the ribosomal mRNA channel to inhibit translation. *Nat. Struct. Mol. Biol.* **27**, 959–966 (2020)

Selvaraj, V., Dapaah-Afriyie, K., Finn, A. & Flanigan, T. P. Short-Term Dexamethasone in Sars-CoV-2 Patients. *R. I. Med. J. (2013)* **103**, 39–43 (2020)

Shang, J. *et al.* Cell entry mechanisms of SARS-CoV-2. *Proc. Natl. Acad. Sci. U. S. A.* **117**, 11727–11734 (2020)

Shin, D. *et al.* Papain-like protease regulates SARS-CoV-2 viral spread and innate immunity. *Nature* **587**, 657–662 (2020)

Shives, K. D., Massey, A. R., May, N. A., Morrison, T. E. & Beckham, J. D. 4EBP-dependent signaling supports west nile virus growth and protein expression. *Viruses* **8**, (2016)

Siu, K. L. *et al.* Severe acute respiratory syndrome Coronavirus ORF3a protein activates the NLRP3 inflammasome by promoting TRAF3-dependent ubiquitination of ASC. *FASEB J.* **33**, 8865–8877 (2019)

Skvara, H. *et al.* The PKC inhibitor AEB071 may be a therapeutic option for psoriasis. *J. Clin. Invest.* **118**, 3151–3159 (2008)

Smoak, K. A. & Cidlowski, J. A. Mechanisms of glucocorticoid receptor signaling during inflammation. *Mech. Ageing Dev.* **125**, 697–706 (2004)

Snijder, E. J. *et al.* A unifying structural and functional model of the coronavirus replication organelle: Tracking down RNA synthesis. *PLoS Biol.* **18**, e3000715-25 (2020)

Sonenberg, N. & Hinnebusch, A. G. Regulation of Translation Initiation in Eukaryotes: Mechanisms and Biological Targets. *Cell* **136**, 731–745 (2009)

Song, G., Ouyang, G. & Bao, S. The activation of Akt/PKB signaling pathway and cell survival. *J. Cell. Mol. Med.* **9**, 59–71 (2005)

Sriram, K., Loomba, R. & Insel, P. A. Targeting the renin-angiotensin signaling pathway in COVID-19: Unanswered questions, opportunities, and challenges. *Proc. Natl. Acad. Sci. U. S. A.* **117**, 29274–29282 (2020)

Stahla-Beek, H. J. *et al.* Identification of a Novel Antiviral Inhibitor of the Flavivirus Guanylyltransferase Enzyme. *J. Virol.* **86**, 8730–8739 (2012)

Subissi, L. *et al.* One severe acute respiratory syndrome coronavirus protein complex integrates processive RNA polymerase and exonuclease activities. *Proc. Natl. Acad. Sci. U. S. A.* **111**, E3900–E3909 (2014)

Szelag, M., Piaszyk-Borychowska, A., Plens-Galaska, M., Wesoly, J. & Bluyssen, H. A. R. Targeted inhibition of STATs and IRFs as a potential treatment strategy in cardiovascular disease. *Oncotarget* **7**, 48788–48812 (2016)

Tanaka, T., Narazaki, M. & Kishimoto, T. Immunotherapeutic implications of IL-6 blockade for cytokine storm. *Immunotherapy* **8**, 959–970 (2016)

Tanner, J. A. *et al.* The adamantane-derived bananins are potent inhibitors of the helicase activities and replication of SARS coronavirus. *Chem. Biol.* **12**, 303–311 (2005)

The RECOVERY Collaborative Group. Tocilizumab in patients admitted to hospital with COVID-19 (RECOVERY): a randomised, controlled, open-label, platform trial. *Lancet* **397**, 1637–1645 (2021)

The RECOVERY Collaborative Group. Dexamethasone in Hospitalized Patients with Covid-19. *N. Engl. J. Med.* **384**, 693–704 (2021)

Thoms, M. *et al.* Structural basis for translational shutdown and immune evasion by the Nsp1 protein of SARS-CoV-2. *Science (80-.).* **369**, 1249–1256 (2020)

Thorne, L. G. *et al.* SARS-CoV-2 sensing by RIG-I and MDA5 links epithelial infection to macrophage inflammation. *EMBO J.* **40**, 2020.12.23.424169 (2021)

Tolcher, A. *et al.* A phase i trial of LY2584702 tosylate, a p70 S6 kinase inhibitor, in patients with advanced solid tumours. *Eur. J. Cancer* **50**, 867–875 (2014)

Totzke, J. *et al.* Takinib, a Selective TAK1 Inhibitor, Broadens the Therapeutic Efficacy of TNF-α Inhibition for Cancer and Autoimmune Disease. *Cell Chem. Biol.* **24**, 1029-1039.e7 (2017)

Ucciferri, C. *et al.* Canakinumab in a subgroup of patients with COVID-19. *Lancet Rheumatol.* **2**, e457-ee458 (2020)

Uhal, B. D., Li, X., Xue, A., Gao, X. & Abdul-Hafez, A. Regulation of alveolar epithelial cell survival by the ACE-2/angiotensin 1-7/ Mas axis. *Am. J. Physiol. - Lung Cell. Mol. Physiol.* **301**, 269–274 (2011)

Vabret, N. *et al.* Immunology of COVID-19: Current State of the Science. *Immunity* **52**, 910–941 (2020)

Vaduganathan, M. *et al.* Renin–Angiotensin–Aldosterone System Inhibitors in Patients with Covid-19. *N. Engl. J. Med.* **382**, 1653–1659 (2020)

Vanderheiden, A. *et al.* Type I and Type III Interferons Restrict SARS-CoV-2 Infection of Human Airway Epithelial Cultures. *J. Virol.* **94**, (2020)

Vincent, M. J. *et al.* Chloroquine is a potent inhibitor of SARS coronavirus infection and spread. *Virol. J.* **2**, 1–10 (2005)

Viswanathan, T. *et al.* Structural basis of RNA cap modification by SARS-CoV-2. *Nat. Commun.* **11**, 3718–7 (2020)

Wack, A., Terczyńska-Dyla, E. & Hartmann, R. Guarding the frontiers: The biology of type III interferons. *Nat. Immunol.* **16**, 802–809 (2015)

Walls, A. C. *et al.* Structure, Function, and Antigenicity of the SARS-CoV-2 Spike Glycoprotein. *Cell* **181**, 281-292.e6 (2020)

Walters, B. & Thompson, S. R. Cap-independent translational control of carcinogenesis. *Front. Oncol.* **6**, 128 (2016)

Wang, J. *et al.* Urban particulate matter triggers lung inflammation via the ROS-MAPK- NF-κB signaling pathway. *J. Thorac. Dis.* **9**, 4398–4412 (2017)

Wang, M. *et al.* Remdesivir and chloroquine effectively inhibit the recently emerged novel coronavirus (2019-nCoV) in vitro. *Cell Res.* **30**, 269–271 (2020)

Wang, S. *et al.* Endocytosis of the receptor-binding domain of SARS-CoV spike protein together with virus receptor ACE2. *Virus Res.* **136**, 8–15 (2008)

Wang, X. *et al.* The anti-influenza virus drug, arbidol is an efficient inhibitor of SARS-CoV-2 in vitro. *Cell Discov.* **6**, 4–8 (2020)

Winer, E. S. & DeAngelo, D. J. A Review of Omacetaxine: A Chronic Myeloid Leukemia Treatment Resurrected. *Oncol. Ther.* **6**, 9–20 (2018)

Wolff, G. *et al.* A molecular pore spans the double membrane of the coronavirus replication organelle. *Science (80-.).* **369**, 1395–1398 (2020)

Xia, H. *et al.* Evasion of Type I Interferon by SARS-CoV-2. *Cell Rep.* **33**, 108234 (2020)

Xiu, S. *et al.* Inhibitors of SARS-CoV-2 Entry: Current and Future Opportunities. *J. Med. Chem.* **63**, 12256–12274 (2020)

Xu, W. feng *et al.* Gasdermin E-derived caspase-3 inhibitors effectively protect mice from acute hepatic failure. *Acta Pharmacol. Sin.* **42**, 68–76 (2021)

Yang, J. *et al.* Targeting PI3K in cancer: mechanisms and advances in clinical trials. *Mol. Cancer* **18**, 26 (2019)

Yang, Q. *et al.* Inhibition of SARS-CoV-2 viral entry in vitro upon blocking N- and O-glycan elaboration. *bioRxiv* **9**, (2020)

Yao, H. *et al.* Molecular Architecture of the SARS-CoV-2 Virus. *Cell* **183**, 730-738.e13 (2020)

Yu, T. *et al.* TBK1 inhibitors: A review of patent literature (2011 - 2014). *Expert Opin. Ther. Pat.* **25**, 1385–1396 (2015)

Yuen, C. K. *et al.* SARS-CoV-2 nsp13, nsp14, nsp15 and orf6 function as potent interferon antagonists. *Emerg. Microbes Infect.* **9**, 1418–1428 (2020)

Zang, R. *et al.* Cholesterol 25-hydroxylase suppresses SARS-CoV-2 replication by blocking membrane fusion. *Proc. Natl. Acad. Sci. U. S. A.* **117**, 32105–32113 (2020)

Zhang, H. *et al.* Selective, potent blockade of the IRE1 and ATF6 pathways by 4-phenylbutyric acid analogues. *Br. J. Pharmacol.* **170**, 822–834 (2013)

Zhang, X. *et al.* Sequential ER stress and inflammatory responses are induced by SARS-CoV-2 ORF3 through ERphagy. *bioRxiv* (2020) doi:10.1101/2020.11.17.387902

Zhao, X. *et al.* LY6E Restricts Entry of Human Coronaviruses, Including Currently Pandemic SARS-CoV-2. *J. Virol.* **94**, (2020)

Zhou, P. *et al.* A pneumonia outbreak associated with a new coronavirus of probable bat origin. *Nature* **579**, 270–273 (2020)
