# Supplementary material for: Executable network of SARS-CoV-2-host interaction predicts drug combination treatments
Source: NPJ Digit Med. 2022 Feb 14;5:18. doi: 10.1038/s41746-022-00561-5 (PMC8844383; doi:10.1038/s41746-022-00561-5)
Supplement: Supplementary file 3 — REPORTING SUMMARY [file 41746_2022_561_MOESM3_ESM.pdf]

nature portfolio

Corresponding author(s): Jasmin Fisher and Clare Jolly

Last updated by author(s): 2021 12 20

Reporting Summary

Nature Portfolio wishes to improve the reproducibility of the work that we publish. This form provides structure for consistency and transparency in reporting. For further information on Nature Portfolio policies, see our Editorial Policies and the Editorial Policy Checklist.

Statistics

For all statistical analyses, confirm that the following items are present in the figure legend, table legend, main text, or Methods section.

n/a	Confirmed	
☐	☒	The exact sample size (n) for each experimental group/condition, given as a discrete number and unit of measurement
☐	☒	A statement on whether measurements were taken from distinct samples or whether the same sample was measured repeatedly
☐	☒	The statistical test(s) used AND whether they are one- or two-sided<br>*Only common tests should be described solely by name; describe more complex techniques in the Methods section.*
☒	☐	A description of all covariates tested
☒	☐	A description of any assumptions or corrections, such as tests of normality and adjustment for multiple comparisons
☒	☐	A full description of the statistical parameters including central tendency (e.g. means) or other basic estimates (e.g. regression coefficient) AND variation (e.g. standard deviation) or associated estimates of uncertainty (e.g. confidence intervals)
☐	☒	For null hypothesis testing, the test statistic (e.g. F, t, r) with confidence intervals, effect sizes, degrees of freedom and P value noted<br>*Give P values as exact values whenever suitable.*
☒	☐	For Bayesian analysis, information on the choice of priors and Markov chain Monte Carlo settings
☒	☐	For hierarchical and complex designs, identification of the appropriate level for tests and full reporting of outcomes
☒	☐	Estimates of effect sizes (e.g. Cohen's d, Pearson's r), indicating how they were calculated

Our web collection on statistics for biologists contains articles on many of the points above.

Software and code

Policy information about availability of computer code

Data collection	NA
Data analysis	Custom code written using the R programming language was used.

For manuscripts utilizing custom algorithms or software that are central to the research but not yet described in published literature, software must be made available to editors and reviewers. We strongly encourage code deposition in a community repository (e.g. GitHub). See the Nature Portfolio guidelines for submitting code & software for further information.

Data

Policy information about availability of data

All manuscripts must include a data availability statement. This statement should provide the following information, where applicable:

- Accession codes, unique identifiers, or web links for publicly available datasets
- A description of any restrictions on data availability
- For clinical datasets or third party data, please ensure that the statement adheres to our policy

Data Availability
Authors can confirm that all relevant data are included in the paper and/ or its supplementary information files. The model is available at https://github.com/JFisherLab/COVID19.

Field-specific reporting

Please select the one below that is the best fit for your research. If you are not sure, read the appropriate sections before making your selection.

☒ Life sciences ☐ Behavioural & social sciences ☐ Ecological, evolutionary & environmental sciences

For a reference copy of the document with all sections, see nature.com/documents/nr-reporting-summary-flat.pdf

Life sciences study design

All studies must disclose on these points even when the disclosure is negative.

Sample size	No sample size calculation was performed. Multiple independent experiments were repeated to allow for appropriate statistical analysis. N is stated for each set of data.
Data exclusions	No data relating to figures in the manuscript were excluded from the analysis due to lack of replication or reproducibility.
Replication	In vitro experiments were performed independently a minimum of 3 times (unless otherwise stated) to allow for appropriate confidence. No data relating to figures in the manuscript were excluded from the analysis due to lack of replication or reproducibility. N is stated in the figures.
Randomization	No randomisation was performed.
Blinding	Blinding was not necessary as there was no group allocation, all measurements were quantified by automated machines, and no data were excluded.

Reporting for specific materials, systems and methods

We require information from authors about some types of materials, experimental systems and methods used in many studies. Here, indicate whether each material, system or method listed is relevant to your study. If you are not sure if a list item applies to your research, read the appropriate section before selecting a response.

Materials & experimental systems

n/a	Involved in the study	
☒	☐	Antibodies
☐	☒	Eukaryotic cell lines
☒	☐	Palaeontology and archaeology
☒	☐	Animals and other organisms
☒	☐	Human research participants
☒	☐	Clinical data
☒	☐	Dual use research of concern

Methods

n/a	Involved in the study	
☒	☐	ChIP-seq
☐	☒	Flow cytometry
☒	☐	MRI-based neuroimaging

Eukaryotic cell lines

Policy information about cell lines

Cell line source(s)	Caco-2 cells were a kind gift from Dr. Dalan Bailey (Pirbright Institute, UK).
Authentication	Cacao-2 cells were authenticated by morphology and ACE2 expression.
Mycoplasma contamination	Random mycoplasma testing was conducted and cells tested negative.
Commonly misidentified lines (See ICLAC register)	None

Flow Cytometry

Plots

Confirm that:

☒ The axis labels state the marker and fluorochrome used (e.g. CD4-FITC).

☒ The axis scales are clearly visible. Include numbers along axes only for bottom left plot of group (a 'group' is an analysis of identical markers).

☐ All plots are contour plots with outliers or pseudocolor plots.

☒ A numerical value for number of cells or percentage (with statistics) is provided.

Methodology

Sample preparation	Infection levels were measured at 24h by flowcytometry. Caco-2 cells were trypsinised, stained with fixable Zombie UV Live/ Dead dye (Biolegend) and fixed with 4% PFA before intracellular staining for nucleocapsid protein. For intracellular detection of SARS-CoV-2 nucleoprotein, cells were permeabilised for 15 min with Intracellular Staining Perm Wash Buffer (BioLegend). Cells were then incubated with 1µg/ml CR3009 SARS-CoV-2 cross-reactive antibody (a kind gift from Dr. Laura McCoy) in permeabilisation buffer for 30 min at room temperature, washed once and incubated with secondary Alexa Fluor 488-Donkey-anti-Human IgG (Jackson Labs).
Instrument	All samples were acquired and analysed using a NovoCyte (Agilent).
Software	NovoExpress 1.5.0 software (Agilent).
Cell population abundance	At least 1000 live Caco-2 cells were acquired per condition.
Gating strategy	Caco-2 cell gating:<br>(1) Caco-2 cells (SSC-A/FSC-A)<br>(2) Singlets (FSC-H/FSC-A)<br>(3) Live Caco-2 cells (Live/Dead NIR/FSC-A)<br>(4) Nucleocapsid-positive cells (anti-Nucleocapsid-AF488/FSC-A)

☒ Tick this box to confirm that a figure exemplifying the gating strategy is provided in the Supplementary Information.
